# *BrCNGC* gene family in field mustard: genome-wide identification, characterization, comparative synteny, evolution and expression profiling

**DOI:** 10.1038/s41598-021-03712-y

**Published:** 2021-12-17

**Authors:** Akram Ali Baloch, Agha Muhammad Raza, Shahjahan Shabbir Ahmed Rana, Saad Ullah, Samiullah Khan, Zaib-un-Nisa, Humera Zahid, Gohram Khan Malghani, Kaleem U. Kakar

**Affiliations:** 1 Department of Biotechnology, Faculty of Life Sciences, Balochistan University of Information Technology, Engineering, and Management Sciences (BUITEMS), Quetta, 87300 Pakistan; 2 Department of Microbiology, Faculty of Life Sciences, Balochistan University of Information Technology, Engineering and Management Sciences (BUITEMS), Quetta, 87300 Pakistan; 3 Department of Botany, GC University Lahore, Lahore, Pakistan; 4 Department of Zoology, University of Balochistan, Quetta, Pakistan; 5 Department of Environmental Sciences, Faculty of Life Sciences, Balochistan University of Information Technology, Engineering and Management Sciences (BUITEMS), Quetta, 87300 Pakistan

**Keywords:** Phylogenetics, Calcium channels, Cyclic nucleotide-gated cation channels, Genomics, Plant biotechnology, Plant sciences, Plant evolution, Plant signalling, Plant stress responses

## Abstract

CNGCs are ligand-gated calcium signaling channels, which participate in important biological processes in eukaryotes. However, the *CNGC* gene family is not well-investigated in *Brassica rapa* L. (i.e., field mustard) that is economically important and evolutionary model crop. In this study, we systematically identified 29 member genes in *BrCNGC* gene family, and studied their physico-chemical properties. The *BrCNGC* family was classified into four major and two sub phylogenetic groups. These genes were randomly localized on nine chromosomes, and dispersed into three sub-genomes of *B. rapa* L. Both whole-genome triplication and gene duplication (i.e., segmental/tandem) events participated in the expansion of the *BrCNGC* family. Using *in-silico* bioinformatics approaches, we determined the gene structures, conserved motif compositions, protein interaction networks, and revealed that most *BrCNGCs* can be regulated by phosphorylation and microRNAs of diverse functionality. The differential expression patterns of *BrCNGC* genes in different plant tissues, and in response to different biotic, abiotic and hormonal stress types, suggest their strong role in plant growth, development and stress tolerance. Notably, *BrCNGC-9*, *27*, *18* and *11* exhibited highest responses in terms of fold-changes against club-root pathogen *Plasmodiophora brassicae*, *Pseudomonas syringae* pv. *maculicola*, methyl-jasmonate, and trace elements. These results provide foundation for the selection of candidate *BrCNGC* genes for future breeding of field mustard.

## Introduction

CNGCs, i.e., Cyclic nucleotide-gated ion channels, are porous cation-conducting channels and elements of the signal transduction pathways that allows the transportation of calcium, sodium and potassium cations across the cell membranes^1^. Therefore, CNGC proteins are usually found within the cytoplasmic membrane^2,3^, vacuole membrane^4^, or nuclear membrane^5^. In animals, the CNGCs have been reported to transfer the signals required by sensory processes^6^. However, in plants, the CNGCs perform more diverse functions such as absorption of the essential and toxic cations, Ca^2+^ signalling, growth, fertility of pollen, geotropism, leaf senescence, inherent immunity, and tolerance to biotic and abiotic stress^2,7–10^. The CNGC-encoded proteins in animal system have been well characterized, but, research on plant *CNGCs* has just begun and these genes have been reported from limited plant species, comprising *Arabidopsis thaliana*^1^, rice^11^, pear^12^, tomato^13^, *Physcomitrella patens*^14^ and tobacco^15^. During our latest study of *Brassica oleracea* genome, we uncovered many hidden features of plant CNGCs including conserved domains, gene structures, phylogeny and evolution, function and underlying mechanisms^16^. For example, CNGC proteins have well-preserved domain structures comprising ion transport domain at N-terminus and cNMP-binding domain at C-terminus that embodies phosphate-binding cassette (PBC) and hinge region, thus regulates the closing and opening of channel via cAMP and/or cGMP^11,17^. Additionally, a Calmodulin (CaM) binding domain controls the activity of CNGC from inside the cell by calcium, CaM and cNMP which act as secondary messengers, and a conserved isoleucine–glutamine (IQ) at C-terminus motif that upon binding to the CaM regulates the channel activity^2,17–19^. Moreover, the plant *CNGC* families can be phylogenetically classified into four groups, and the member genes function in a group-dependent manner. For example, Group IV-a *CNGCs* are reported to function in salt stress tolerance^4^, while the Group IV-b genes may be involved disease and heat resistance^20,21^. It is not clear if this correspondingly applies to other plant species^22^.

*Brassica rapa* L. is one of the notable member of the *Brassicaceae* (also known as *Cruciferae*, mustard or the cabbage family) known for its agricultural and economic importance^23,24^. *B. rapa* L. is one such important vegetable plant with medicinal properties, and is highly consumed around the world^25^. Besides, the unique genome structure of *B. rapa* L, represented by multiple sub-genomic fractions and closed syntenic relation with *B. oleracea* and Arabidopsis, makes it an important model crop for studies involving plant genomics, evolution, breeding and molecular genetics. Dietary *Brassica* crops are important for their economic, nutritional (lutein, vitamin A, folate, vitamin C, vitamin K and calcium) and antioxidant properties^26^. It is believed that a high consumption of *Brassica* vegetables reduces the threat of age -related chronic diseases^27^ and lessens the risk of several types of cancer^28,29^. The genome of this important *crucifer* crop is sequenced and deposited as in *Brassica* database (BRAD). Taking advantage of the available genomic data, we performed genome-wide identification of the *CNGC* family in *B. rapa* L. We employed multiple in silico approaches to perform genomic and functional investigates of *CNGC* genes and proteins in field mustard, including systematic characterization, classification, phylogeny, synteny, evolution, and gene expression.

## Results and discussion

### Genome-wide identification of *BrCNGC* family

**Figure 1:**
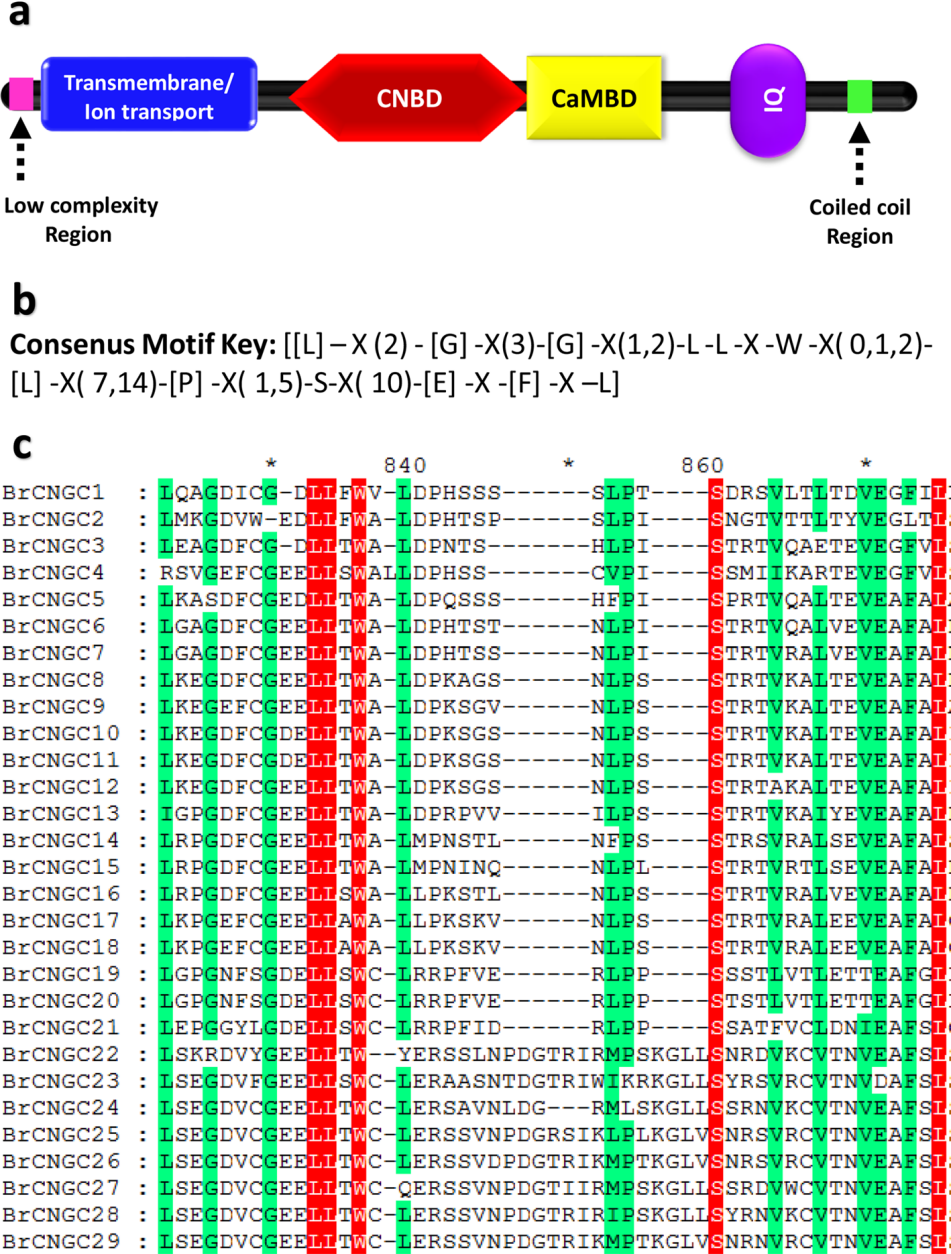
Domain architecture, consensus motif key and conserved cNMP-binding domain (CNBD) alignment of *BrCNGC* family proteins. (**a**) Each protein of the *BrCNGC* family comprised a fully conserved CNBD and IT domains, with overlapped CaMBD and adjacent IQ domains. (**b**) Plant and *Brassica*-specific CNGC-recognizing specific consensus motif key deduced after multiple sequence alignment at > 90% conservation. (**c**) Multiple sequence alignment of BrCNGC proteins using CNBD domain.

The *CNGC* genes play vital roles in development, ion transport, signaling and stress responses^11–13^, and the *CNGC* gene families have been studied in limited yet important crops^30–33^. However, the systematic identification and annotation of this family has not been performed in crucifer plants except for Chinese cabbage by our group recently^16^. The genomic sequence of *B. rapa* L., one of the most significant species of *Brassica* genus, was released in 2011^24^. Therefore, proper annotation and identification of the *CNGC* genes in *B. rapa* L. was performed in this study. All non-redundant putative gene sequences were retrieved from BRAD database, and analysed for the presence of plant CNGC-specific conserved domains and motifs. Consequently, accessions either having truncated sequences or missing CNGC-specific domains were discarded from further analysis. For instance, accession *Bra022235,* was a short truncated sequence lacking essential plant CNGC-specific domains such as CNBD^33^. Finally, twenty-nine genes with full length amino acid sequences (> 500 aa) were identified and confirmed as members of the *BrCNGC* family (Table 1). Each protein of the *BrCNGC* family comprised a fully conserved CNBD and IT domains, with overlapped CaMBD and adjacent IQ domains (Fig. 1a,c). Within the CNBD, the two most conserved regions were identified: a PBC motif, which binds the sugar and phosphate moieties of the cNMP ligand, and a “hinge” region adjacent to the PBC, which is believed to contribute to ligand binding efficacy and selectivity^17^. Moreover, the latest criterion for identification of *CNGC* genes is the validation of CNGC-specific motif key, which upon failing can mislead both the readers and researchers regarding the plant *CNGCs* including their classification and overall structure as a family. Using multiple sequence alignment at > 90% conservation, we deduced the BrCNGC-specific consensus motif key [[L] – X (2)—[G] –X (3)-[G] –X (1,2)-L -L -X -W –X (0,1,2)-[L] –X (7,14)-[P] –X (1,5)-S-X (10)-[E] -X -[F] -X –L] (Fig. 1b). The key spanning the PBC and hinge region within the CNBD domain, recognizes all 29 BrCNGCs identified in this study.

**Table 1:** Summary of 29 *BrCNGC* genes identified in the genome of *B. rapa*.

Gene	Accession	Chr	Start	Stop	Strand	Primary domains		Secondary domains		Group	Corresponding AtCNGC
*BrCNGC1*	Bra034281	A04	11980216	11982791	+	cNMP	IT	CaMBD	IQ	G1	12
*BrCNGC2*	Bra003323	A07	15879616	15883454	–	cNMP	IT	–	IQ		12
*BrCNGC3*	Bra004537	A05	687357	690331	–	cNMP	IT	–	IQ		3
*BrCNGC4*	Bra031515	A01	16651616	16656087	+	cNMP	IT	–	IQ		3
*BrCNGC5*	Bra000937	A03	14054247	14058116	–	cNMP	IT	CaMBD	IQ		13
*BrCNGC6*	Bra003081	A10	5414086	5416746	–	cNMP	IT	CaMBD	IQ		1
*BrCNGC7*	Bra022632	A02	7390572	7393211	+	cNMP	IT	CaMBD	IQ		1
*BrCNGC8*	Bra026086	A06	5904523	5907153	–	cNMP	IT	CaMBD	IQ	G2	7
*BrCNGC9*	Bra020402	A02	5537255	5540170	–	cNMP	IT	CaMBD	IQ		5
*BrCNGC10*	Bra032132	A04	11074762	11077889	–	cNMP	IT	CaMBD	IQ		6
*BrCNGC11*	Bra039221	A09	32929402	32932962	+	cNMP	IT	CaMBD	IQ		6
*BrCNGC12*	Bra024067	A03	27904482	27907069	–	cNMP	IT	CaMBD	IQ		9
*BrCNGC13*	Bra011963	A07	13141306	13144346	+	cNMP	IT	CaMBD	IQ	G3	15
*BrCNGC14*	Bra008733	A10	12426314	12429518	+	cNMP	IT	CaMBD	IQ		18
*BrCNGC15*	Bra018089	A06	9846882	9849809	+	cNMP	IT	CaMBD	IQ		16
*BrCNGC16*	Bra011186	A01	3422819	3426535	+	cNMP	IT	CaMBD	IQ		17
*BrCNGC17*	Bra007839	A09	32710513	32713938	–	cNMP	IT	CaMBD	IQ		14
*BrCNGC18*	Bra032081	A04	11383157	11386389	+	cNMP	IT	CaMBD	IQ		14
*BrCNGC19*	Bra022702	A02	6903420	6907955	+	cNMP	IT	CaMBD	IQ	G4a	4
*BrCNGC20*	Bra003001	A10	6203509	6208673	–	cNMP	IT	CaMBD	IQ		4
*BrCNGC21*	Bra008699	A10	12252329	12255245	+	cNMP	IT	CaMBD	IQ		2
*BrCNGC22*	Bra001678	A03	17843897	17852047	+	cNMP	IT	–		G4b	20
*BrCNGC23*	Bra031529	A01	16542495	16546796	–	cNMP	IT	–			20
*BrCNGC24*	Bra029958	A01	14741686	14746906	+	cNMP	IT	CaMBD	IQ		20
*BrCNGC25*	Bra021265	A01	22108307	22111729	–	cNMP	IT	CaMBD	IQ		19
*BrCNGC26*	Bra022233	A05	19633895	19637029	–	cNMP	IT	CaMBD	IQ		19
*BrCNGC27*	Bra001676	A03	17833555	17836622	+	cNMP	IT	–			20
*BrCNGC28*	Bra021266	A01	22102703	22106050	+	cNMP	IT	CaMBD	IQ		20
*BrCNGC29*	Bra022232	A05	19638792	19642102	–	cNMP	IT	CaMBD	IQ		19

### Phylogenetic analysis and classification of BrCNGCs

**Figure 2:**
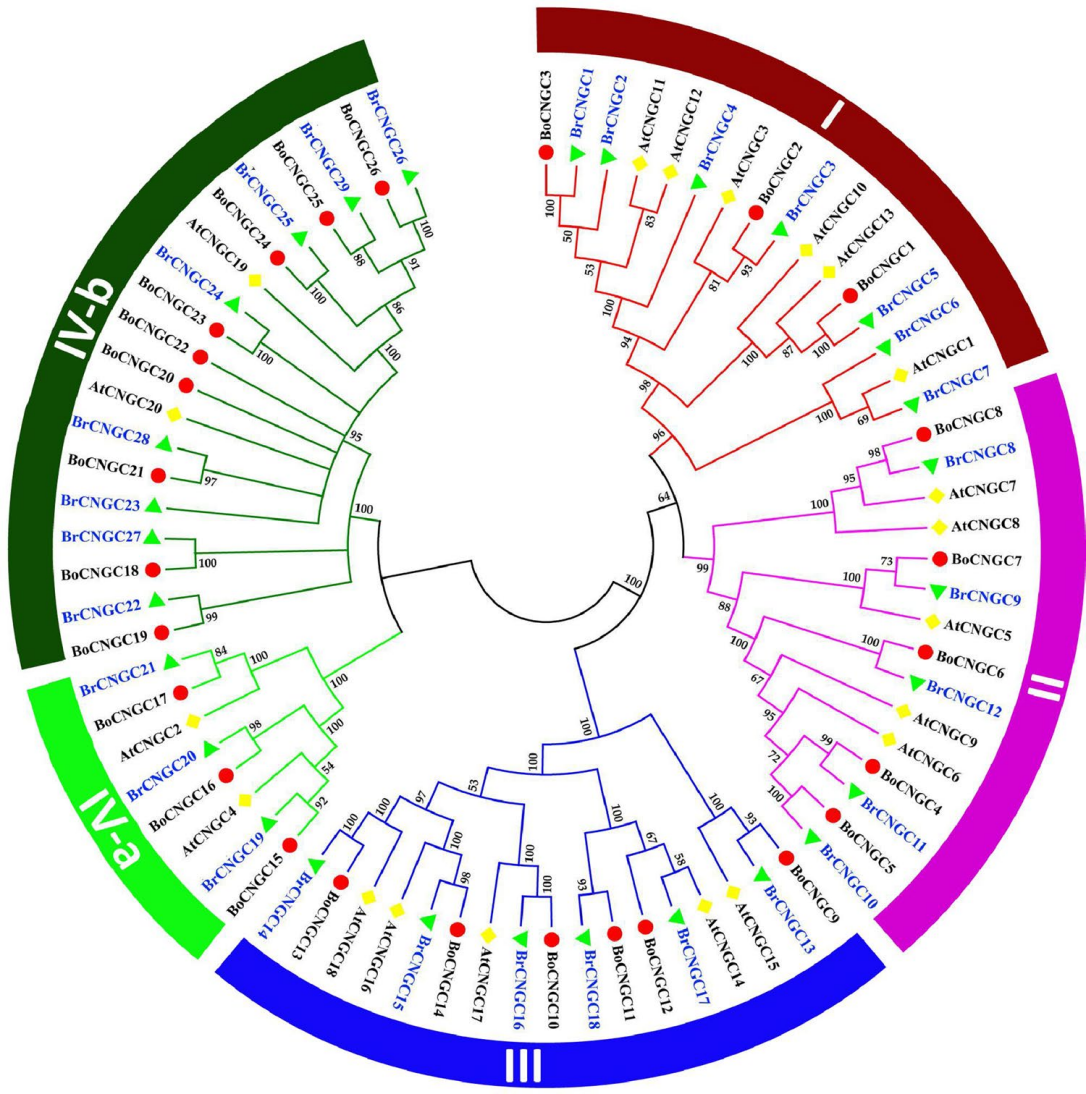
Phylogeny of CNGC proteins from *Brassica rapa* L. and *A. thaliana*. A maximum likelihood phylogenetic tree was created with MEGA 6.0, using the Jones–Taylor–Thornton model. The bootstrap values from 1000 replications are provided at each node. The BoCNGC proteins identified in this study are indicated with maroon diamonds, while the AtCNGCs are indicated with blue squares. Each group is highlighted in different color.

It is anticipated that homologs belonging to the similar taxonomic clade probably also resemble in structural, functional and evolutionary properties^34^. Such information can be used in clarifying the role(s) of the newly identified BrCNGCs. The multiple sequence alignment using full length amino acid sequences and conserved domains showed > 90% resemblance of the representative BrCNGCs among themselves, and with their respective orthologs in *A. thaliana* (i.e., AtCNGCs) and *B. oleracea* (BoCNGCs) (Fig. 2; Supplementary Figs. S1–S4 and Tables S1−S2)^16^. Using neighbor-joining method, the *BrCNGC* gene family was classified into four main clades based on the classification of *AtCNGCs,* tree topology and bootstrap values (Fig. 2; Supplementary Fig. S4). The member *BrCNGC* genes were named based on their positions in phylogenetic tree. Among these, seven *BrCNGC* genes clustered in clade-I, five in clade-II, and six in clade-III. Clade-IV that was additionally separated into two sub-clade (IV-a and IV-b), contained highest number of *BrCNGCs* genes (i.e., 11). These findings were in covenant to the previous investigations^1,15,16^*.*

### Chromosomal mapping and distribution on three sub-genomes

**Figure 3:**
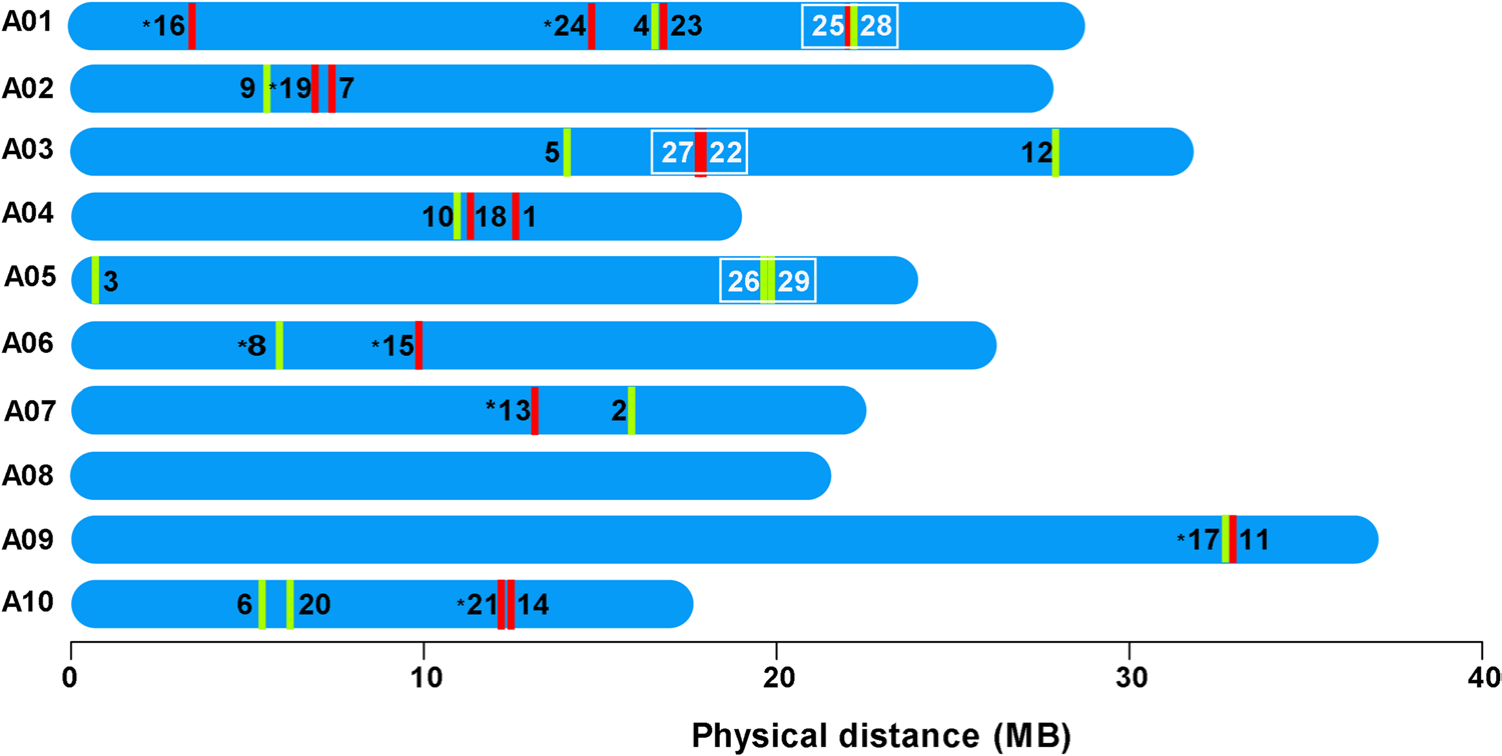
Chromosomal localization and duplication of *BrCNGC* family genes. Physical location and distance of *BrCNGC* genes across the 9 chromosomes of *B. rapa*. *BrCNGC* genes are shown as numbers on chromosomes, tandemly duplicated gene pairs by white color, while segmental duplications are indicated with asterisks. Red and yellow lines show forward and reverse orientations of each loci respectively.

The 29 *BrCNGC* genes were unsystematically dispersed across the *B. rapa* L. genome and localized on nine of ten chromosomes (i.e., A01–07 and A09–A10). The distribution of *BrCNGC* genes on chromosomes was uneven, for example, chromosome A01 carried six genes, while others had 2–4 genes. None of *BrCNGC* genes was localized on chromosome A08. Among 29 *BrCNGC* genes, 15 loci were located on forward strand, while 14 loci were positioned on reverse strand of the chromosomes (Fig. 3). Similar to *B. oleracea*, the genome of *B. rapa* L. is currently fractioned into three sub-genomes: i.e., least fractionated (LF), medium fractionated (MF-I) and most fractionated (MF-II)^24^. The LF sub-genome of *B. rapa* L. contained maximum numbers of *BrCNGC* genes (i.e., 14 genes), while MF-II carried only 3 *BrCNCG* genes (Table 2). These findings are agreement to our previous findings of *BoCNGC* sub-genomes^16^.

**Table 2:** Syntenic ancestral block structure between *A. thaliana* and three sub-genomes of *B. oleracea* and *B. rapa* L.

*A. thaliana*		*B. rapa*			*B. oleracea*			
START A	Original Block	LF	MF-I	MF-II	LF	MF-I	MF-II	Potential overlap/tandem repeats
AtCNGC13	O	–	BrCNGC05	–	–	BoCNGC01	–	–
AtCNGC03	J	BrCNGC03	–	–	–	BoCNGC02	–	–
–	–	BrCNGC01	–	–	BoCNGC03			
AtCNGC06	I	BrCNGC10	BrCNGC11	–	BoCNGC05	BoCNGC04	–	–
AtCNGC09	U	–	BrCNGC12	–	–	BoCNGC06	–	–
AtCNGC05	W	–	BrCNGC09	–	–	BoCNGC07	–	–
AtCNGC07	A	BrCNGC08	–	–	BoCNGC08	–	–	–
AtCNGC15	I	–	BrCNGC13	–	–	BoCNGC09	–	–
AtCNGC17	U	BrCNGC16	–	–	BoCNGC10	–	–	–
AtCNGC14	I	BrCNGC18	BrCNGC17	–	BoCNGC11	BoCNGC12	–	–
AtCNGC18	R	BrCNGC14	–	–	BoCNGC13	–	–	–
AtCNGC16	M	BrCNGC15	–	–	BoCNGC14	–	–	
AtCNGC04	W	BrCNGC20		BrCNGC19	BoCNGC16	BoCNGC15	–	–
AtCNGC02	R	BrCNGC21	–	–	BoCNGC17	–	–	–
AtCNGC19	F	BrCNGC29 BrCNGC26	BrCNGC25	BrCNGC27 BrCNGC22	BoCNGC25	BoCNGC18	BoCNGC24	BoCNGC22
AtCNGC20			BrCNGC28		BoCNGC26	BoCNGC19	BoCNGC21	BoCNGC20
–	–	BrCNGC24	–	–	BoCNGC23	–	–	–

### Evolution of *BrCNGC* family

#### Origin and comparative synteny analysis of *BrCNGC* family genes

*B. rapa* L. is an ancient polyploid, whose genome has undergone whole genome triplication (WGT) event ~ 13–17 million years ago (MYA), after divergence from *A. thaliana*, followed by large-scale re-diploidization (chromosomal re-arrangements)^35^. Being a member of the conventional triangle of U^36^, the assembled genome of *B. rapa* L. (312 Mb) is smaller than sister specie *B. oleracea* (540 Mb)^37^, which diverged from a common ancestor ~ 4 MYA^38^. Currently, the genomes of *B. oleracea* and *B. rapa* L. are categorised as LF, MFI and MF-II^37^. Because of a *Brassica*-lineage specific WGT, each *A. thaliana CNGC* gene was expected to generate three *Brassica* copies. However, there were 20 *AtCNGC* genes, 29 *BrCNGC* genes, and 26 *BoCNGC* genes. The LF, MF-I and MF-II sub-genomes, respectively retained 65%, 40% and 15% of the *CNGC* genes found in *A. thaliana*. To detect the retention or loss of *CNGC* genes after a WGT, the syntenic map of *BrCNGC* genes with the model *A. thaliana* and *B. oleracea CNGC* genes provided markers for defining the regions of conserved synteny among the three genomes (Supplementary Fig. S5) (Table 2). We found that more than > 80% of *BrCNGC* genes are located in well-conserved syntenic blocks, with deletion and gain of some genes, which coincides with the previous findings^39^. Compared with the ancestral *Brassicaceae* blocks (A to X) in *A. thaliana*, the synteny of 75% of the *CNGC* gene family was preserved in *Brassica* species, based on the number of *corresponding* genes. Ten of the 20 *AtCNGC* genes were retained as single copy in the equivalent blocks of both *Brassica* species. Three *AtCNGC* genes (i.e., AT2G23980, AT2G24610 and AT5G54250), located on I and W syntenic blocks, were preserved as two copies in *Brassica* genomes, which were asymmetrically fractionated into three sub-genomes. Two *AtCNGC* genes (i.e., *AT3G17690* and *AT3G17700*) in F syntenic block were retained as three copies in each species. Two *BrCNGC* genes (i.e., *BrCNGC1* and *BrCNGC24*) were respectively located on conserved syntenic block with *BoCNGC3* and *BoCNGC23*, but not with *AtCNGC* genes. An extra gene copy (*Bra022235*) was located on potential overlap/tandemly repeated regions of F block along with gene pair *BrCNGC26* and *BrCNGC29* (Table 2). Thorough examination revealed that this gene has lost its functional CNBD domain during the course of evolution. These results are agreement to the findings of Duan et al.^40^ who reported that functionally redundant gene copies are lost after genome duplication event, while functionally important some gene copies are retained. Together, these finding suggest that WGT, along with segmental duplication played important role in expansion of *BrCNGC* gene family overall, while, tandem duplication was identified to play role in expansion of group IV-b only. Moreover, conservation of *CNGC* genes after substantial genome reshuffling event suggests that these genes are crucial for plant development^41^.

#### Gene duplication events and expansion of *BrCNGC* family

Gene family expands through one of three possible mechanisms including tandem and segmental duplication, and/or whole-genome duplication^42^. The examination of gene duplication events showed that three gene pairs (i.e., *BrCNGC25/BrCNGC28, BrCNGC22/BrCNGC27* and *BrCNGC26/BrCNGC29*) are tandemly duplicated genes in *B. rapa* L. genome, as revealed by analysis in PTGBase. These tandemly duplicated genes are located on adjacent loci of chromosome 1, 3 and 5 respectively. In addition, 8 *BrCNGC* genes were likely associated with segmental duplications, which however require further elucidation (Fig. 3). These observations suggest that both tandem and segmental duplications may have donated to functional and enlargement diversity of *BrCNGC* gene family.

#### Gene structures and conserved motifs of *BrCNGC*-encoded proteins

**Figure 4:**
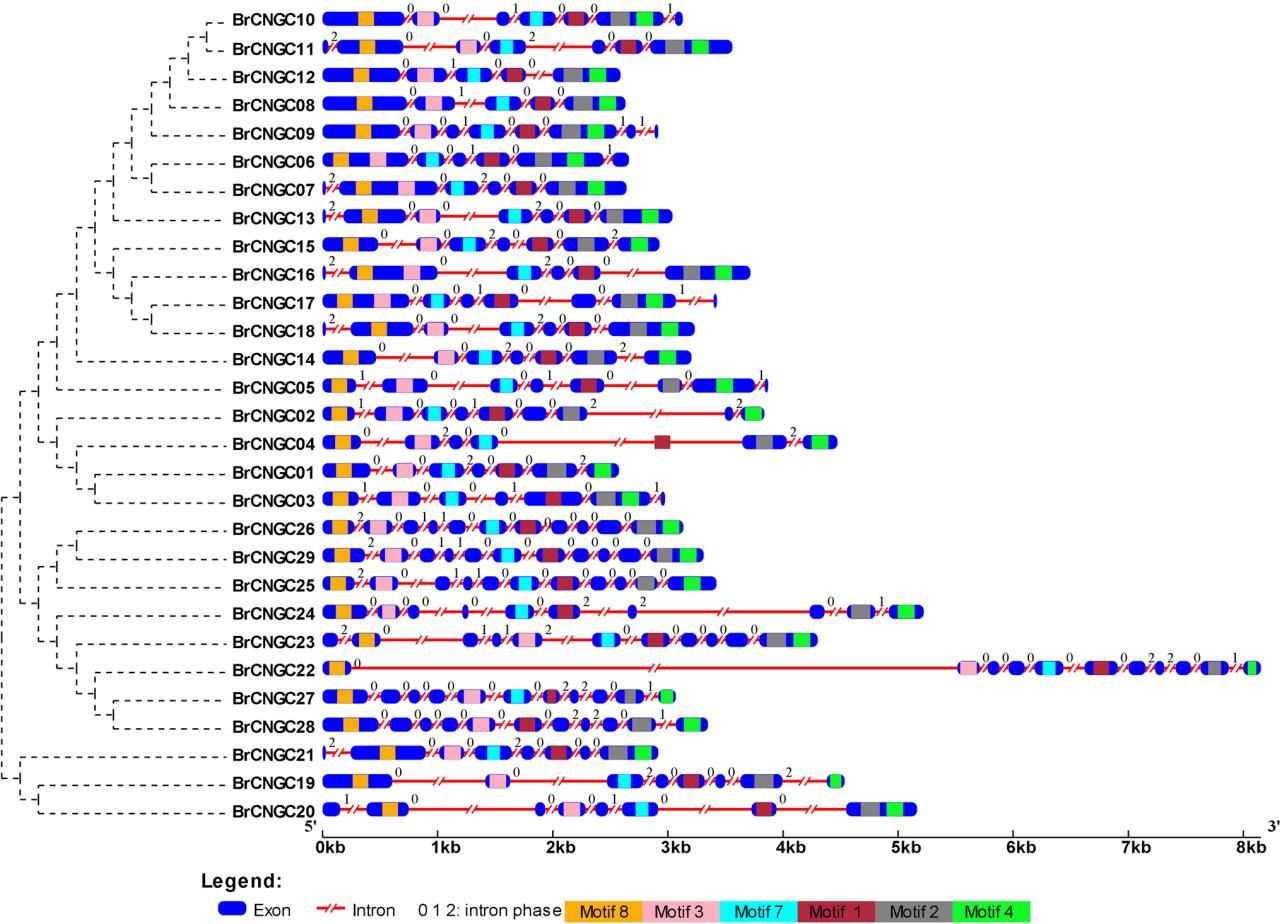
Schematic diagram showing the structures of *BrCNGC* genes and conserved motifs in their encoded proteins. Exon–intron organization and conserved motifs within the 29 BrCNGCs. The NJ phylogenetic tree of CDs is shown on the left side of the figure, exons-introns indicated as blue boxes and red lines respectively, and motifs are represented by colored boxes within the exons. Numbers [0, 1 and 2] given on gene structures represent the respective intron phases. The lengths of each exon and intron can be mapped to the scale given in the bottom. The order of motifs corresponds to the position of the motifs in protein sequence, however, the length of the boxes does not correspond to the lengths of motifs.

The diversity in exon–intron play an imperative role in gene families evolution, which provide more evidences of phylogenetic clustering^43^. Here, we analyzed the exon–intron orderliness of the individual *BrCNGC* gene, and conserved motifs in their encoded protein sequences to describe the structural variety of the *BrCNGC* family. The most of the *BrCNGC* genes from phylogenetic clade I-III included six or seven exons, while, clade IV-b contained highest number of exons, ranging between 10 and 11 (Fig. 4). Nearly grouped *BrCNGC* genes in the similar clades were alike on the subject of the number of exons-introns sizes. Maximum of the introns in *BrCNGC* genes were phase-0 introns that exist in between complete codons. Thirty-three phase-1 introns that are separated by 1st codon and thirty-five phase-2 introns that are positioned in the middle of the second and third nucleotides of a codon were detected in the *BrCNGC* family. The exceptions were *BrCNGC3*, *BrCNGC5* and *BrCNGC9*, which comprised three phase-1 introns. Comparison of exon–intron organization with the *AtCNGC* genes which clustered into similar phylogenetic groups shown numerous alterations (Supplementary Fig. S6). Utmost of the phase-1 and 2 introns were existing in *AtCNGC* genes, inferring that intron loss for the duration of evolution caused in a reduction in the number of introns in *BoCNGC* genes, principally those in clade I–III and IV-a (Supplementary Fig. S7).

Ten conserved motifs were identified in BrCNGCs during motif structure studies Multiple Expectation Maximization for Motif Elicitation suite (MEME)^44^. Rendering to Pfam codes^45^ and WebLogos, only six motifs (i.e., 1–4, 7, and 8) comprised domains with known functions (Fig. 4, Supplementary Fig. S8 and Table S3). Motif 1 was the biggest motif accompanying with product of unknown functions. Motifs 2, 3, 7, 8 and 4, which encode a CNBD, an ion transport domain, and IQ domain, correspondingly, were preserved among all BrCNGC family members. Notably, each clade members had similar arrangement of functionally annotated motifs, reveals that the directly associated proteins in each clade showed alike motif arrangements and perhaps functional resemblances too. The functionality of the leftover motifs (1, 5, 6, 9 and 10) wait for additional experimental evidence.

#### Protein sequence features and physico-chemical properties of BrCNGCs

The biochemical and physiological characteristics of the 29 BrCNGC proteins were identified (Table 3). The ProtParam tool showed that most of these proteins are localized in plasma-membrane. BrCNGC proteins varied in lengths from 556 to 786 aa with average of 711 aa, molecular weight (64.27–90.34 kDa), and residue weight (112.566–116.172 g/mol depending on the number of atoms present. Approximately, one-thirds of the BrCNGC proteins had low net charge (< 19) and relatively low isoelectric points (pI < 9). Approximately, all BrCNGC were hydrophilic, with BrCNGC21 and BrCNGC23 being somewhat hydrophobic. Based on the aliphatic index, most BrCNGC proteins were thermostable, similar to other globular proteins. Rendering to the instability index (II), none of the BrCNGC family proteins was stable in the test tube (Table 3). Additionally, the BrCNGC proteins had more positively charged residues than negatively charged residues (Supplementary Fig. S9). Hydrogen was the most abundant, followed by carbon, nitrogen and oxygen, and sulfur (Supplementary Fig. S10). Leucine was a very common amino acid among the 26 BrCNGC proteins (Supplementary Fig. S11).

**Table 3:** Physico-chemical properties and general features of *BrCNGC*-encoded proteins.

Protein	Length	MW (kDa)	pI	II	Ai	GRAVY	AW (g/mol)	Charge	Loc
BrCNGC1	647	74.76	9.52	40.4	100.8	−0.021	115.555	38.0	PM
BrCNGC2	666	75.66	8.34	41.0	93.11	−0.078	113.604	10.0	PM
BrCNGC3	702	81.03	9.14	42.2	88.89	−0.15	115.430	25.0	PM
BrCNGC4	556	64.27	8.91	41.1	92.91	−0.135	115.594	19.0	PM
BrCNGC5	705	81.34	9.3	48.3	91.3	−0.212	115.381	25.0	PM
BrCNGC6	758	88.06	9.63	50.0	83.61	−0.223	116.172	43.0	PM
BrCNGC7	739	85.76	9.25	56.4	90	−0.131	116.049	28.0	PM
BrCNGC8	712	81.81	9.1	51.8	87.4	−0.242	114.900	19.0	PM
BrCNGC9	749	85.58	9.11	50.2	89.47	−0.101	114.271	24.5	PM
BrCNGC10	746	85.43	9.5	51.8	89.44	−0.188	114.523	37.5	PM
BrCNGC11	737	84.65	9.44	50.6	92.24	−0.181	114.863	31.0	PM
BrCNGC12	712	81.03	9.34	46.5	93.3	−0.134	113.813	26.0	PM
BrCNGC13	684	79.43	9.68	46.4	91.51	−0.102	116.127	38.0	PM
BrCNGC14	714	81.34	8.51	45.0	85.78	−0.141	113.926	13.0	PM
BrCNGC15	706	81.52	8.58	52.6	86.08	−0.247	115.469	15.0	PM
BrCNGC16	728	84.28	8.95	46.5	90.41	−0.212	115.768	18.5	PM
BrCNGC17	733	84.49	9.2	48.2	93.11	−0.131	115.271	24.0	PM
BrCNGC18	728	83.82	9.06	46.9	92.94	−0.103	115.139	21.5	PM
BrCNGC19	695	80.35	8.32	52.5	90.14	−0.186	115.610	11.5	PM
BrCNGC20	698	80.57	8.52	54.3	91.16	−0.174	115.431	13.0	PM
BrCNGC21	719	82.07	9.53	56.8	94.21	0.008	114.147	36.0	PM
BrCNGC22	654	74.65	8.98	41.2	96.07	−0.049	114.138	19.0	PM
BrCNGC23	758	85.32	8.73	45.5	96.45	0.042	112.566	16.5	PM
BrCNGC24	670	75.62	9.58	44.1	90.81	−0.117	112.866	31.0	PM
BrCNGC25	743	85.29	9.61	52.9	89.22	−0.134	114.803	35.5	PM
BrCNGC26	748	86.20	9.26	51.9	88.76	−0.197	115.243	22.5	PM
BrCNGC27	680	77.60	8.94	50.7	89.46	−0.149	114.121	15.0	PM
BrCNGC28	760	86.24	9.55	48.0	91.37	−0.063	113.481	32.5	PM
BrCNGC29	786	90.34	9.71	48.3	89.66	−0.186	114.942	35.0	PM

MW = Molecular Weight, pI = Isoelectric point, PM = Plasma membrane, II = Instability Index, Ai = Aliphatic index, GRAVY = Grand average of hydropathicity, AW = Average residues weight, Loc = Localization.

#### Distribution of Post-translational modifications and microRNA target sites in BrCNGCs

Post-translational modifications (PTMs) of protein upturn the variety of their functions over and done with diverse mechanisms^46^. These mechanisms may include, protein localization, protein–protein interaction, cleavage, degradation or allosterically regulating enzyme activity^47^. We analysed BoCNGC protein sequences using ScanProsite^48^, multiple putative phosphorylation sites were identified (Table 4). These locations may act as substrates for numerous kinases, comprising tyrosine kinase, casein kinase II, cAMP/cGMP kinase, and protein kinase c. All proteins contained non-potential Glycosylphosphatidylinositol (GPI) anchor modification site in their sequences, while 16 BrCNGCs contained PEST-like sequences, which may act as a signal peptides for protein degradation^49^. Most abundant sites were casein kinase II sites, with 17 sites in BrCNGC7, followed by protein kinase C, were the maximum in clade IV members. All BrCNGC proteins had multiple N-glycosylation/ N-myristoylation motif locates are greatly preserved than rest of the PTMs. The rest of the PTM sites, such as those for amidations, leucine zipper patterns, and P-loop of the -GTP/ATP binding site motif A, were less preserved and arbitrarily dispersed, increasing diversity to function and mechanisms of CNGC-definite PTMs^47^. MicroRNAs (miRNAs) are interior non-coding RNAs that direct gene expression, particularly post-transcriptional gene silencing^50^. Recognising the targets of the expected miRNAs could facilitate the understandings of the genetic functions of miRNAs prompting signal transduction, stress adaptations, and plant development^51^. Herein, we investigated for possible miRNA targets in the set of recognized *BrCNGC* transcripts^52^. We recognized 92 miRNAs comprising target sites in 28 *BrCNGC* transcripts using a cut-off threshold of 5 for the search parameters (Supplementary Table S4). Small RNA/target site paired with an expectation score and cut-off threshold of 4 were included to reduce the number of false positive predictions. Consequently, seventeen miRNAs with target sites in fourteen *BrCNGC* genes were recognized, among which, four miRNAs with an expectation score < 3.5 can be considered more reliable (< 3.5) (Supplementary Table S5). Most of the *BrCNGC* genes included target site for single miRNA, except for *BrCNGC14*, *BrCNGC20* and *BrCNGC21*, which contained target sites for 2 miRNAs. The convenience of the target site wide-ranging from 8.828 (bra-miR9552b-5p) to 20.9 (bra-miR160a-3p), where minor values resemble to a grander likelihood of interaction between the target site and miRNA^53^. Eleven miRNAs were found to be participated in cleavage of the target transcript, although six miRNAs supposedly inhibit the translation of target genes. These miRNAs were previously identified as novel or conserved miRNAs by Yu et al.^54^ and Jiang et al.^55^ in *B. rapa* L.and *B. comparstis* ssp. *chinensis*, respectively*.* Former research has shown that some of these miRNA families are greatly preserved in *Brassicaceae* or other plant species, located and expressed in leaves, pollen, roots or flower, with ancient functions in heat stress response (bra-miR5726, bra-miR5712 and bra-miR5716)^54,56^, regulation of target genes related to plant development (i.e., bra-miR156a/b/d-3p, bra-miR824, and bra-miR391-5p)^55^, somatic embryogenesis in *Dimocarpus longan*^57^, *Brassica*-specific hormone signal transduction pathway (i.e., bra-miR162-3p), drought stress tolerance in tomato (i.e., miR160a and miR9552b)^58^ and response to Turnip mosaic virus (i.e., bra-miR1885a and bra-miR5717)^57^. The function of the remaining novel and conserved miRNAs is not known yet, which requires further experimental elucidation.

**Table 4:** Post-translation modification and phosphorylation sites within the 29 BrCNGC encoded protein sequences.

Protein	1	2	3	4	5	6	7	8	9	10	11	12	13
BrCNGC1	621	–	–		2	7	10	3	1	4	–	–	–
BrCNGC2	641	405–422	Y	–	–	14	10	6	1	8	–	1	–
BrCNGC3	673	691–702	–	–	2	7	10	4	2	7	–	–	–
BrCNGC4	530	–	–	–	3	12	10	4	2	6	–	–	–
BrCNGC5	674	694–705	–	–	–	9	8	6	1	4	3	–	–
BrCNGC6	742	–	–	–	–	10	14	5	2	8	–	–	–
BrCNGC7	711	–	–	–	–	7	17	3	2	7	–	–	–
BrCNGC8	681	–	–	–	3	6	16	3	1	7	–	–	1
BrCNGC9	715	737–749	–	–	1	6	11	5	2	8	1	1	–
BrCNGC10	722	–	–	–	3	4	16	3	1	9	–	–	–
BrCNGC11	698	–	–	–	4	3	16	6	1	8	–	–	–
BrCNGC12	677	701–712	Y		2	8	13	6	1	12	–	–	–
BrCNGC13	656	–	–	–	1	5	12	4	2	8	–	–	–
BrCNGC14	683	–	–	–	2	8	10	8		9	1	–	–
BrCNGC15	688	619–666	Y	–	1	8	13	5	1	8	–	–	–
BrCNGC16	701	665–679 / 716–728		–	1	6	13	4	2	7	–	–	–
BrCNGC17	716	722–733	–	–	2	8	16	2	1	5	–	3	–
BrCNGC18	710	717–728	–	–	1	7	15	2	1	5	–	3	–
BrCNGC19	667	–	–	1	1	12	8	3	–	8	1	1	–
BrCNGC20	670	–	–	1	2	13	10	5	–	8	–	–	–
BrCNGC21	691	21–61	–	–	2	8	6	3	1	8	–	–	–
BrCNGC22	634	–	Y	–	3	12	12	8	1	3	1	–	–
BrCNGC23	726	63–107	Y	–	–	11	13	5	–	6	–	–	–
BrCNGC24	651	22–37	Y	–	1	11	12	3	–	6	–	–	–
BrCNGC25	721	1–18 /75–92		–	–	15	14	5	–	7	–	–	–
BrCNGC26	727	–	Y	–	1	15	7	7	–	4	–	–	–
BrCNGC27	646	1–23	Y	–	2	11	11	10	1	5	1	1	–
BrCNGC28	738	75–99	Y	–	–	12	8	3	–	7	–	–	–
BrCNGC29	758	81–113	Y	–	–	13	13	5	–	6	–	–	–

1 = non-potential GPI modification site, 2 = PEST motifs, 3 = cTP-containing sequence, 4 = GLU-RICH, 5 = cAMP- and cGMP-dependent protein kinase phosphorylation sit, 6 = Casein kinase II phosphorylation site, 7 = Protein kinase C phosphorylation site, 8 = N-glycosylation site, 9 = Tyrosine phosphorylation site, 10 = Myristoylation, 11 = Amidation, 12 = Leucine zipper, 13 = ATP/GTP-binding site motif A (P-loop).

#### In-silico functional relationship network of BrCNGC proteins

A theoretical protein–protein interaction was constructed with the STRING program to recognise the relations among unlike BoCNGC proteins^59^. The interaction network of first shell of interactors presented that thirteen BrCNGCs were part of various protein–protein interaction networks (Supplementary Fig. S12). Among these, seven proteins, namely BrCNGC2, 14–18 and interact with ubiquitin3 protein (Bra009542), detected by Affinity Capture-MS assay. It is reported that Polyubiquitin chain upon covalent binding to target protein governs proteolysis, DNA damage tolerance and other processes^60^. In another association, BrCNGC29 interact with Constitutive Photomorphogenic 1, experimentally determined by biochemical data from psi-mi (fluorescent resonance energy transfer) assay and two-hybrid assay during former research on *Arabidopsis* CNGCs. The functional annotation showed that COP1 serve as a negative regulator of photomorphogenesis in *Arabidopsis*^61^. Similarly, BrCNGC2 interacted with multiple proteins including BrCNGC18 and Bra00322 (a truncated *CNGC* gene), whose genes probably have correlated expression.

### Functional analyses of *BrCNGCs* by transcriptome-based expression profiling

#### Expression patterns in different plant parts and wounding stress

**Figure 5:**
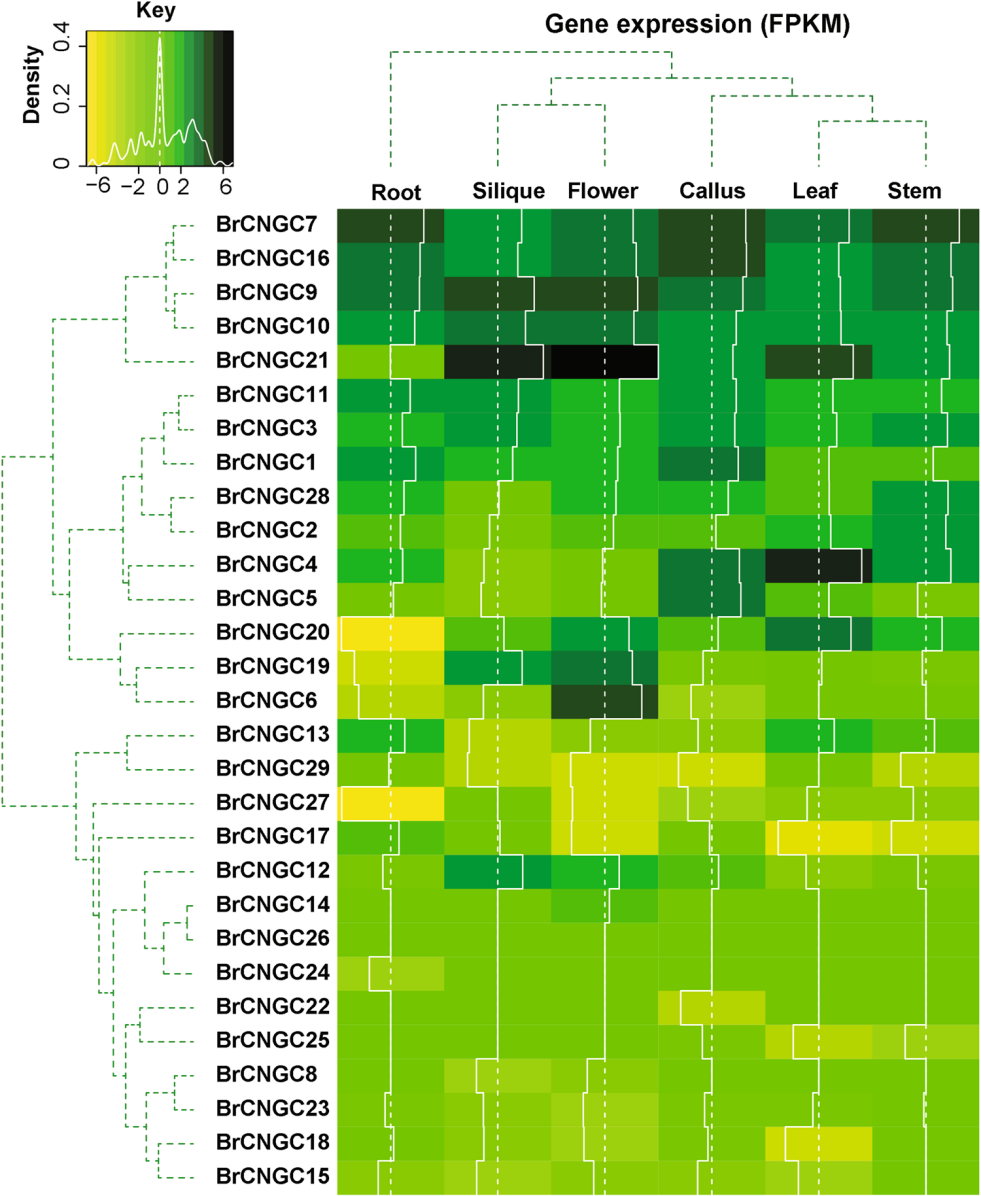
Heatmap showing the transcript abundance of *BrCNGC* genes in different development tissues of *Brassica rapa* L. The gene names and cluster tree are indicated on the left side of the figure. Normalized gene expression (FPKM) is expressed in log2 ratio, with yellow colors indicating lower accumulation of transcripts, and green colors indicating higher accumulation of the gene transcripts. The intensity of transcript abundance is indicated as white histograms within the heatmap.

Scrutinising the steady-state expression patterns of *BrCNGC* genes in six tissues (i.e., root, stem, flower, silique, leaf, and callus) was performed via Illumina RNA-sequencing data from the Gene Expression Omnibus (GEO) database database. Out of the 29 *BrCNGCs*, fifteen were expressed at moderately high levels (fragments per kilobase of exon model per million mapped reads value > 1) in at least one tissue, including ten in silique, eleven in calli, twelve in the roots and stem, and fourteen in leaves and flowers. The remaining genes either displayed lowest transcript accumulation or did not express in any tissue (Fig. 5; Supplementary Table S6). An additional investigation revealed that *BrCNGC21* was the highest expressed genes, particularly in flowers and silique, suggesting they may be vital for *Brassica* species development. Amongst the other genes, *BrCNGC4* was greatly expressed in leaves, *BrCNGC7* in stem and roots, although *BrCNGC16* was greatly expressed in calli. Greater expression in silique and calli suggest the expression of these genes is induced by wounding.

Our data suggest that *BrCNGC* genes in different tissues expressed differently, and that several genes are induced by wounding^22^. Highly expressed genes in certain tissues indicated some functional preservation, while others showing functional dissimilarities^62,63^.

#### Expression patterns in response to hormonal stress

**Figure 6:**
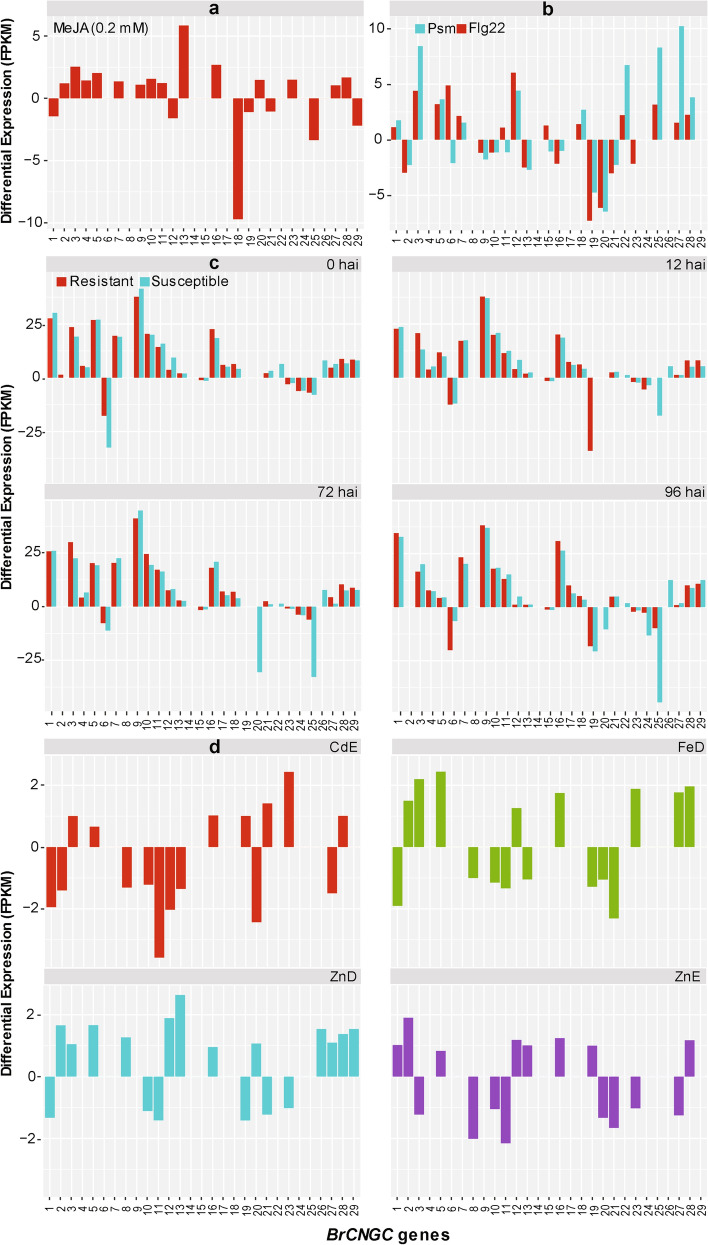
Dynamic expression profiles of *BrCNGC* genes in *Brassica rapa* L. plants, subjected to different stress types. (**a**) Exogenous hormone (0.2 mM of MeJA). (**b**) Bacterial pathogen (*Psm*) and elicitor flagellin (Flg22). (**c**) *P. brassicae* in clubroot resistant and susceptible Cabbage lines at 0, 12 72 and 96 h after inoculation. (**d**) Trace element stress represented by cadmium excess (CdE), iron deficiency (FeD), zinc excess (ZnE) and deficiency (ZnD), respectively. The final relative expression level of each transcript shown in this figure is calculated as fold change compared to controls/mocks, where threshold > 0 indicate up-regulation and threshold < 0 show down-regulation.

RNA-Seq technology allows a better understanding of the regulation of the important genes in the secondary metabolite biosynthetic pathways in plants, including *Brassica*^64^. Methyl jasmonate (MeJA) is one such plant hormone that is used in diverse developmental pathways and defense in plants^65^. We determined the expression profiles of 29 *BrCNGCs* in the leaves of *B. rapa*, exposed to 0.2 mM of MeJA (Supplementary Table S7). The calculated fold-change data showed that fourteen genes were up-regulated at 8–10th leaf stage, seven genes were down-regulated, while the remaining genes didn’t show low transcript abundance compared to control (Fig. 6a). Among these, *BrCNGC13* showed maxim level of expression, which was up-regulated > 5.8-fold compared to unstressed control. On other hand, *BrCNGC18* showed maximum negative response, which was—ninefold down-regulated compared to control. This pattern was followed by *BrCNGC25* and *BrCNGC29* respectively. These results indicated that the transcriptional responses of *CNGCs* along with other signal transduction pathway genes are regulated by MeJA^66,67^.

#### Expression patterns in response to bacterial pathogen and elicitor stress

Phytoalexins are antimicrobial substances produced by plants to elicit resistance against pathogen infection^68^. Most of the phyloalexin biosynthesis pathways are reported to be conserved across the *B. rapa* L. cultivars, Chiifu and Rapid Cycling (*RCBr*). Using illumina RNA-sequencing, Klein et al.^69^ observed that some of phyloalexin biosynthesis pathways are activated by infiltration with the *Pseudomonas syringae* pv. *maculicola* (*Psm*) and oligopeptide epitope of bacterial flagellin (flg22). Our search of the transcriptome data revealed the expression profiles of *BrCNGC* genes in the leaves of 15 days old *RCBr* plants, infiltrated by *Psm* and flg22. The FPKM values of 29 *BrCNGC* genes are shown in Supplementary Table S8. Most of the *BrCNGC* genes were expressed at higher levels after 9 h post-infiltration, including twenty-two genes in response to flg22, and twenty-one in response to *Psm* (Fig. 6b). Among these, > 18 *BrCNGC* genes were mutually expressed under both treatments, four expressed differentially, while seven genes didn’t show any expression compared to uninfected controls. Compared with their mock treatments, the expression of ten genes was increased and eleven decreased in response to *Psm*. The maximum responses were noted for *BrCNGC27* (> tenfold up-regulation) and *BrCNGC20* (– sixfold down-regulation), respectively*.* On other hand, the expression of thirteen genes was increased and nine decreased in response to flg22, with notable responses shown by *BrCNGC12* (> sixfold up-regulation) and *BrCNGC19* (i.e.,—7.2-fold down-regulation) respectively. The results showed that three duplicated gene pairs (i.e., *BrCNGC-22*/*27*, *25*/*28* and *26*/*29*) has similar expression trend (Fig. 6b). These results indicate that various *CNGCs* may be involved plant defense against bacterial pathogens^34^.

#### Expression patterns in response to clubroot pathogen *Plasmodiophora brassicae*

*Plasmodiophora brassicae* is among the most common pathogens worldwide, which cause clubroot disease in *Brassica* crops^70^. In a latest study, Chen et al.^71^ profiled the transcriptomes of the roots from two near-isogenic lines (NILs) of *B. rapa* L., namely clubroot-resistant and clubroot-susceptible. This RNA-seq library (i.e., GSE74044) contained the expressions of 26 *BrCNGCs* in 30-days old *B. rapa* L. NILs inoculated with *P. brassicae*, and the data collected after 0, 12, 72 and 96 h after inoculation (Supplementary Table S9). The missing profiles of the remaining three genes (i.e., *BrCNGC2*, *8*, and *14*), might be due to no expression at all, or these genes had spatial and temporal expression patterns^35^. As shown in fig.ure6c, almost similar expression trends were observed between two NILs, where 17 to 19 genes were up-regulated, and five or six genes were down-regulated at one or other time point. Five genes, including *BrCNGC19*, *20*, *22*, *25* and *26*, showed irregular expression between two cultivars at different time points. Comparatively, maximum level of expression was noted for *BrCNGC9*, which peaked in both NILs at all-time points (~ 37 to 44-folds), while, maximum negative responses was shown by *BrCNGC25*, which was—44-fold down-regulated in clubroot-susceptible at 96 hai. Among others, the transcripts of all genes, except *BrCNGC-6*, *15*, *20*, and *23–25*, were up-regulated, showing that some of *BrCNGCs* can be further explored to understand their mechanism to facilitate resistance to *P. brassicae*.

#### Expression patterns in response to trace elements stress

Trace elements are essential for human nutrients to fulfill their metabolic requirements^72^. Among these trace elements, iron (Fe) and zinc (Zn) are mainly significant, because their deficiency cause serious health and nutritional problems in human population^73^. On the other hand, Cadmium (Cd) is a toxic element found in the soil, which cause severe toxicity in plants, animals and humans^74^. It is documented fact that the excess of zinc intake also cause toxicity, which can be more harmful to the plants, compared to Zn deficiency^75^. Taking advantage of recently published transcriptome data^76^, we investigated the expression patterns of *BrCNGC* genes in leaves of *B. rapa* L. plants cultivated under Cd excess (CdE), Fe deficiency (FeD), and Zn deficiency (ZnD) and excess (ZnE) conditions (Supplementary Table S10). Compared to control, seven genes were up-regulated under CdE, eight under FeD, twelve under ZnD, and eight genes were up-regulated under ZnE condition, respectively (Fig. 6d). On the contrary, nine genes were down-regulated under CdE, eight under FeD, six under ZnD, and eight genes were down-regulated under ZnE, respectively. Some of the multi-copy genes, such as *BrCNGC26* and *BrCNGC29*, showed similar trend under ZnD stress, while other gene pairs exhibited differential patterns. These observations are agreement to the findings of Li et al.^76^. The data showed that some of *BrCNGC* family genes are definitely involved in trace elements response, and further experiments will clarify their individual roles and help in improving environmental adaptability in *B. rapa* L.

## Methods

### Genome-wide identification of CNGC proteins

The identify *CNGC* gene family in *B. rapa* L., the protein sequences of twenty *Arabidopsis CNGCs* were collected from TAIR10^77^ and BLAST searched against target proteomes in BRAD database^78^, using built-in BLASTP search. The matching protein sequences of target species were retrieved and analyzed in SMART^79^, Pfam^80^ and Motif search service on GenomeNet for domain analysis. Finally, the target protein sequences comprising cNMP-binding (IPR000595) and ion transport (PF00520) domains were recognized as candidate CNGCs and manually checked for the presence plant CNGC-specific consensus motif within the cNMP-binding region^14^. The newly identified *CNGC* genes were named according to standard nomenclature (i.e., taxonomic initials such Br for *B. rapa* L.) and phylogenetic positions.

### Multiple sequence alignment and phylogenetic analysis

ClustalX 2.0 program was performed for Multiple sequence alignments of the BrCNGC proteins^81^ and were observed by GeneDoc^82^. MEGA software version 6.0, was used for phylogenetic tree construction^83^. For identification purposes, the BrCNGC proteins were individually aligned with AtCNGCs and phylogeny performed. Multiple sequence alignments based on the CNGC proteins from both species were used for combined rooted tree by using *Amborella trichopoda* CNGC (AMTR_s00210p00019190) as outgroup.

### Characterization and properties of BrCNGCs

The data about gene and protein lengths, their chromosome locations and positional information of the *CNGCs* were obtained from BRAD database. The ProtParam tool was used to study the amino acid properties BrCNGC proteins^84^. The ScanProsite tool was used search the post-translational modifications sites^48^.

### Chromosomal mapping, gene duplication and syntenic analysis

The positional information from BRAD database was used for genomic mapping of *CNGC* genes on *B. rapa* L. chromosomes by using R script. The tandem and segmental duplications were analyzed by PGDD^85^ and PTGBase^39^. The synteny relationship between *BrCNGCs*, *AtCNGCs* and *BoCNGCs* were assessed in Bolbase^86^, and mapped in a circos plot using R studio^87^.

### Conserved motif composition and Gene structure

To predict the gene structures, we used Gene Structure Display Server (GSDS 2.0)^88^. To find conserved motifs in the CNGC protein sequences, we used the MEME and MAST motif discovery tools with default parameters^44^. The annotation of the motifs were performed in Pfam program^45^.

### The miRNA target sites and protein–protein interaction

The microRNA sequences of *B. rapa* L. were collected from miRBase database^89^ and submitted to psRNATarget server^52^ for miRNA’s target sites prediction within the *BrCNGC* genes. Each of these miRNAs were searched online to find their experimental proof, function and related literature. The protein–protein interaction of BrCNGC proteins was constructed in STRING v10^59^, by using the CNGC protein sequences as reference.

### Data sources and expression of *BrCNGC* genes

For expression profiling of *BrCNGC* genes in different plant tissues, the RNA-seq data placed in GEO database (GSE43245) was used^38,86^. For gene expression against different stress treatments, the expression data (GSE69785) of 15 days old *RCBr* plants infiltrated with *Psm* and flg22^69^, GSE74044 for expression in the roots of 30-day old NILs at 0, 12 , 72 and 96 h after inoculation of *P. brassicae*^71^, GSE51363 for expression in the leaves of *B. rapa* L. subsp. *pekinensis*, exposed to 0.2 mM MeJA at 8–10 leaf stage, and GSE55264 for expression in the leaves of 14 days plants exposed to Fe deficiency (0.05 µM; Normal = 3 µM), Zn deficiency (0.005 µM; Normal = 2 µM), Zn excess (50 µM; Normal = 2 µM) and Cd excess (1 µM; Normal = without Cd) for 7 days was used. Transcript abundance was calculated as FPKM and the values were log2 transformed. Data were plotted in heat maps generated in R studio^90^. For abiotic and biotic stress, we used a fold-change method, where the threshold of ≥ 0 defines a gene as “positively expressed/up-regulated” and threshold ≤ 0 as “negatively expressed/down-regulated”, compared to FPKM values in control treatments.

## Conclusion

This work is the first wide-ranging and systematic study of *CNGC* gene family in *B. rapa* L. This work identifies and fills the remaining gaps in literature, and present a clearer picture about plant *CNGCs* in general, and crucifers in particular. Here, we have tried to explore each and every aspect of *BrCNGC* gene family, from genes to protein, including gene structure, motif composition, miRNA target sites, post-translational modification sites, protein interaction network, GO-term prediction and orthologous relationship etc. The phylogenetic and synteny analyses will help in understanding the evolutionary patterns, and diversification and/or expansion of *CNGC* family genes in complex ancient polyploids (e.g., *B. rapa/B. oleracea*), whose genomes have undergone multiple duplication and reshuffling events. Additionally, this work will contribute to further clarify the functions of differentially expressed candidate *BrCNGC* genes through cloning, and to investigate their roles in the regulation of cascade pathways, plant development and stress tolerance in *B. rapa* L.

## Supplementary Information

Supplementary Information 1.

Supplementary Information 2.

## Data Availability

The raw sequence datasets generated and analyzed during the current study are available through BRAD database (http://brassicadb.org/brad/). The gene expression data analyzed during the current study are available at GEO database with accession numbers: GSE43245, GSE69785, GSE74044, GSE51363 and GSE55264.

## Abbreviations

*B. rapa* L.: *Brassica rapa* L
CNGC: Cyclic nucleotide-gated ion channel
cNMPs: Cyclic nucleotide monophosphates
flg22: Flagellin
*Psm*: *Pseudomonas syringae* Pv. *Maculicola*
NILs: Near-isogenic lines
CaM: Calmodulin
