# Supplementary material for: BrCNGC gene family in field mustard: genome-wide identification, characterization, comparative synteny, evolution and expression profiling
Source: Sci Rep. 2021 Dec 17;11:24203. doi: 10.1038/s41598-021-03712-y (PMC8683401; doi:10.1038/s41598-021-03712-y)

BrCNGC gene family in Field mustard: Genome-wide identification, characterization, comparative synteny, evolution and expression profiling

Akram Ali Baloch[1], Agha Muhammad Raza[2], Shahjahan Shabbir Ahmed Rana[1], Saadullah[2], Samiullah Khan[1], Zaib-un-Nisa[3], Humera Zahid[4], Muhammad Gohram Khan Malghani[5] and Kaleem U. Kakar[2*]

[1] Department of Biotechnology, Faculty of Life Sciences, Balochistan University of Information Technology, Engineering, and Management Sciences (BUITEMS), Quetta, 87300, Pakistan

[2] Department of Microbiology, Faculty of Life Sciences, Balochistan University of Information Technology, Engineering and Management Sciences (BUITEMS), Quetta, 87300, Pakistan

[3] Department of Botany, GC University Lahore

[4] Department of Zoology, University of Balochistan, Quetta

[5] Department of Environmental Sciences, Faculty of Life Sciences, Balochistan University of Information Technology, Engineering and Management Sciences (BUITEMS), Quetta, 87300, Pakistan

***Correspondence:** Kaleem.ullah3@buitms.edu.pk

Figure S1. Multiple sequence alignment of *BrCNGC* family proteins using full length amino acid sequences.

```
                    *        20         *        40         *
BrCNGC1  : -------------------------------------------------- :  -
BrCNGC2  : -------------------------------------------------- :  -
BrCNGC3  : -----------------------------------MEMMNLKRNTFVKFT : 15
BrCNGC4  : -------------------------------------------------- :  -
BrCNGC5  : --------------------------------------MGFGRDSRVRFK : 12
BrCNGC6  : MKFRLTSLTLSSCGSRVLHSSHKEGYTTISCASTNSPTMNFRPDKSVRFH : 50
BrCNGC7  : --------------------------------------MNFRQEKFVRFQ : 12
BrCNGC8  : --------------------------------------MMTKRNCFGFPV : 12
BrCNGC9  : ----------MTFASLPYHFCRASDRGLSRSSVIELAIMAGKPQTFVSVD : 40
BrCNGC10 : -------------------------MFDCGTNGVKSQVISGHREKFIRLE : 25
BrCNGC11 : -------------------------------MESKSQVISGHREKFIRLD : 19
BrCNGC12 : ---------------------------------------MDSRYSQGAEA : 11
BrCNGC13 : --------------------------------------MGYGNSRSVRFE : 12
BrCNGC14 : -------------------------------------------------- :  -
BrCNGC15 : -------------------------------------MSNLHLHTSARF- : 12
BrCNGC16 : --------------------------------------MELRKDKILMLY : 12
BrCNGC17 : --------------------------------------MEFKRDNTVRFY : 12
BrCNGC18 : --------------------------------------MEFKRDNTVRFY : 12
BrCNGC19 : --------------------------MATEQEFTRASRVSGASSSVGYYS : 24
BrCNGC20 : --------------------------MATEQEFTRASRVSRASSSIGYYS : 24
BrCNGC21 : ----------------------MPSHTNFLFRWIGLFSQKLRRETTEISE : 28
BrCNGC22 : ---MASSNGYDDVPMLPVSCTSSSSRTRPFTSRSRSVSLSNTSSTIDVFE : 47
BrCNGC23 : MAPPNEN--DDALKLPVLD-TSSSSRTRPFTFRSRNVSLPNTSSTSEGL- : 46
BrCNGC24 : -------------MLPISDASSSSSQTRVFTSRTRSVPLSNPTEETG--- : 34
BrCNGC25 : MTSPNEN--DQVSI----PEATSRAHTRAFNFKNRSVSLSNSTYYIDGC- : 43
BrCNGC26 : MASPNENESDEFPMLRQFP----EARSRALHSRNRSISFSDSTYSSNRVE : 46
BrCNGC27 : ---MASPMENDDVPMLPASDTSSSSRTMPFTSRSRSTSLANNSSTIDVF- : 46
BrCNGC28 : MASPKEN--DDVPMLPISD----TSRTRPFTSRSRSVSLSNTCSTIDGF- : 43
BrCNGC29 : MASPNEK--DEFPILLPVPEARSRANTRAFNSRNRSVSFSNSTYSTNRV- : 47
```

```
                   60         *        80         *       100
BrCNGC1  : -------------------------------------------------- :   -
BrCNGC2  : -------------------------------------------------- :   -
BrCNGC3  : ENEDSWNRPSVTS------------------------------------- :  28
BrCNGC4  : -------------------------------------------------- :   -
BrCNGC5  : EPSSTEFGYGRRA------------------------------------- :  25
BrCNGC6  : DWKSDKASDVEYS------------------------------------- :  63
BrCNGC7  : DWKSDKTSDVEYS------------------------------------- :  25
BrCNGC8  : KNR----------------------------------------------- :  15
BrCNGC9  : DLDFKLPSSSSLTRQHNY-------------------------------- :  58
BrCNGC10 : SMDSRYSQSSDNTGLNKC-------------------------------- :  43
BrCNGC11 : SMDPRSPEAGLNR------------------------------------- :  32
BrCNGC12 : GLNKCTLNLQGPS------------------------------------- :  24
BrCNGC13 : EDSEVTKPQAVHE------------------------------------- :  25
BrCNGC14 : MNKIRSLRFLLPE------------------------------------- :  13
BrCNGC15 : -------------------------------------------------- :   -
BrCNGC16 : SDKKEPKEAIWAV------------------------------------- :  25
BrCNGC17 : GEEKQTLEATEKR------------------------------------- :  25
BrCNGC18 : GDEKQTLEVTEKR------------------------------------- :  25
BrCNGC19 : DEDYKDEEEEEEE------------------------------------- :  37
BrCNGC20 : DEDYTTDEEENEE------------------------------------- :  37
BrCNGC21 : NNGGESSSSSDDT------------------------------------- :  41
BrCNGC22 : NSSTVVLGYTDPL------------------------------------- :  60
BrCNGC23 : DSSTVVLRYTDPHRTQRP-PPSVQMNGPLFSTSSPEPLILLPPPSTGGSS :  95
BrCNGC24 : NSKAATLGYAGSLPSQR--PPLFPMTGPLSSSTRRSSGYF---------- :  72
BrCNGC25 : DNSKVALGYTVPIRTQRR-PP-----GPLYSTPRPE-SHFPPS------- :  79
BrCNGC26 : NSSGPRRTQSRPS-------PSVHMSGPLYDTRRPDQSFFPPSP------ :  83
BrCNGC27 : NSSTVVLGYTDPLGTQRR-PPLVQMSYPLSSTRSPEPRFALPPP------ :  89
BrCNGC28 : DSSTVVLGYTGPLRAQRR-PPLVQMSGPLSSTRTPEPLFLLPPP-----S :  87
BrCNGC29 : DNSSVVLGYTGPLRTQRRLPPSVQMSGPLYSTRRPDQSFFPPSP------ :  91
```

```
                     *       120         *       140         *
BrCNGC1  : -------------------------------------------------- :   -
BrCNGC2  : ----------------------------------------------MTIF :   4
BrCNGC3  : -----------------------------------------------VIK :  31
BrCNGC4  : -------------------------------------------------- :   -
BrCNGC5  : ------------------------------------------RPSLNAVL :  33
BrCNGC6  : ---------------------------------------EVPDGLYRRAI :  74
BrCNGC7  : ------------------------------------GRNEPPNGIFRRTI :  39
BrCNGC8  : ------------------------------------------GSEKKRAS :  23
BrCNGC9  : --------------------------------SSSISGPLHPIQGSHNTS :  76
BrCNGC10 : --------------------------------TLNIQAPKRFAQGSKTSS :  61
BrCNGC11 : -------------------------------CTINIQRPKRFTQANKTSS :  51
BrCNGC12 : -----------------------------------RANGAGQGNNNNASS :  39
BrCNGC13 : ----------------------------------------ETAVKLKFKI :  35
BrCNGC14 : -------------------------------------------------- :   -
BrCNGC15 : -------------------------------------------------- :   -
BrCNGC16 : -----------------------------------------NDPMSKSYK :  34
BrCNGC17 : ----------------------------------------QPLPMFKPST :  35
BrCNGC18 : --------------------------------------------LPLPMF :  31
BrCNGC19 : ------------------------------------------------EE :  39
BrCNGC20 : ----------------------------------------EEMEELEEAE :  47
BrCNGC21 : -------------------------------------PVLSSGECYACTQ :  54
BrCNGC22 : -------------------------------------------------G :  61
BrCNGC23 : DPVGVSSSQPERYPSFAALEH-DNSDDNSVL-NPHLLRSEKFGVCNDPYC : 143
BrCNGC24 : ----------------GDLEEVNSSDNDELLKHAHRLRSGKLGMCNDPYC : 106
BrCNGC25 : ------IEPPDSS---STVDVRSE--DESVLENANILKSGQLGMCNEPYC : 118
BrCNGC26 : ------VQPPASSLSSSTVDIPSEEVVEALLKNANLLKSGQLGMCNDPYC : 127
BrCNGC27 : ----------------------------------STGASYDSVGASSSQP : 105
BrCNGC28 : DSVGISSSQPERYPSFATLEH-KKSDDEFVLKHANLLRSGQLGMCNDPYC : 136
BrCNGC29 : ------VQPPDSS---STVDVPSEE-DEVVLKNANLLKSGQLGMCNDPYC : 131
```

```
                          160         *         180         *         200
BrCNGC1  : MERASTIQSVHENIKSVRGQL----------------------------- :  21
BrCNGC2  : SVQSTLFTRASVALLSSNGLK--RFSF-----ASSFSSA----------- :  36
BrCNGC3  : KTVRRSFEKGSEKIRTFKQQP---LTF-----HSQKKNENKKKII----- :  68
BrCNGC4  : -------------------------------------------------- :   -
BrCNGC5  : DNVRRGFEKGSDKIRTFKRPL-SFNSH-----KNEEKRNATGTQKK---- :  73
BrCNGC6  : SSISDKFHRSSLRIKMLRTSY----SF-----KETVSKGIVSTH------ : 109
BrCNGC7  : TSISDKFHRSSARIKTFRRTY-KSYSF-----KEAVSKGIDSSH------ :  77
BrCNGC8  : KSFREGVKIGSEGLFSIGKSV-----T-----RAVFPEDLRISEK----- :  58
BrCNGC9  : GSFKKRFQKGSKGLKSIGRSL-GFGVY-----RAVFPEDLKVSEK----- : 115
BrCNGC10 : GSFKKGFRKGSEGLWSIGRSI-GLGVS-----RAVFPEDLKVSEK----- : 100
BrCNGC11 : GSFKKGFRKGSEGLWSIGRSI-GLGVS-----RAVFPEDLEVSEK----- :  90
BrCNGC12 : GSFKKGFRRGSKGLWSLGRSI-GLGVS-----RAVFPEDLKVSER----- :  78
BrCNGC13 : NGAQISPRKNVKKMTRGKSFK--DKVL-----SRVFTEDLGRVKN----- :  73
BrCNGC14 : TITSAASNRGSVAVRYGSQVL--------------------PWRH----- :  38
BrCNGC15 : RNFPTAFSRRHHNNNDLQNQR-GRSVF-----SELGD------------- :  43
BrCNGC16 : LSLPSALKPPDNNLLSGNRIS--RYTD-----NNKSKSSKPSWYK----- :  72
BrCNGC17 : TQFLKPELVIPKKTNKTRLFKLPRFGG-----LKVFPENFEIERD----- :  75
BrCNGC18 : KSSAAPFQKQELGTSKKSIFKIPRFGR-----FKVFPENFEIERD----- :  71
BrCNGC19 : EEMEETEKDEEEEEPRVRVTCGGR--------RNGSPGSYNKWMM----- :  76
BrCNGC20 : EEEEETHVGGTCGIRR----------------RNGSSSSYNKWMV----- :  76
BrCNGC21 : VGVPAFHSTSCDQANAPEWRASAGSSL-----VPIQEGSAPSPVRARIRR :  99
BrCNGC22 : TQRQPQLVQMGDPISSTRNLE----LV-----HNARDDDAKGWARRFVTS : 102
BrCNGC23 : TTCPSYYNRKADQVPTSRVPAIFYSMF-----HSALYEDAKARARRFATS : 188
BrCNGC24 : TTCPSNYNPKASRLPNPTVSA---STF-----HNALYDDARSWARRFASS : 148
BrCNGC25 : TTCPSYYSHQSANFHTSK-VS--DSRF-----HTVLYDDARGWAKRFASC : 160
BrCNGC26 : TTCPSYYNLQAAQFHTYGVVSDSRTQVNVTHNRQALHDYDRGWAKLFASY : 177
BrCNGC27 : NERNHAYSRKAAQSRTPRVFATSDFTL-----HNALDDDAKGWAK----- : 145
BrCNGC28 : TTCPSYYNRKAAQIPSSRVSAFFDSKF-----HNALYDDAKGWARRFATT : 181
BrCNGC29 : TTCPSYYNRQAAQFHTYRVVS--DSRF-----RTALYDDARGWAKRFASS : 174
```

```
                     *        220        *        240        *
BrCNGC1  : ------KKVYKTLKTLENWRKAILLVCVVALG------------------ :  47
BrCNGC2  : ------ALYSPPLPKT--KKRRFPIVSAVDIGGVTVARNDDDPTNNVPDS :  78
BrCNGC3  : ------RVMNPNDSYLQNWNKIFLLLCVVALA------------------ :  94
BrCNGC4  : -----MKTLN--------TRKIVLLVCLVALA------------------ :  19
BrCNGC5  : ------NIINPQGSFLQNWNKIFLFASVIALA------------------ :  99
BrCNGC6  : ------EILDPQGTFLQKWNKIFVLACIIAVS------------------ : 135
BrCNGC7  : ------KILDPQGPFLQRWNKIFVLACIIAVS------------------ : 103
BrCNGC8  : ------KIFDPQDKTLLIWNRMLVISCILAVS------------------ :  84
BrCNGC9  : ------KIFDPQDKTLLFCNKLFVISCILSVF------------------ : 141
BrCNGC10 : ------KIFDPQDKFLLLCNKLFVASCILAVS------------------ : 126
BrCNGC11 : ------KIFDPQDKFLLLCNKLFVASCILAVS------------------ : 116
BrCNGC12 : ------KIFDPQDKFLLLCNKLFVTSCILAVS------------------ : 104
BrCNGC13 : ------KILDPRGQTIRKWNKLFLIACLVSLF------------------ :  99
BrCNGC14 : ------QILDPDSNIVTYWNHVFLVTSIIALF------------------ :  64
BrCNGC15 : ------TTLDPSGDLITRWNHIFLITCLLALF------------------ :  69
BrCNGC16 : ------TILDPGSEIVLKWNWVFIISCMLALF------------------ :  98
BrCNGC17 : ------KILDPGGDVVLQWNRVFLFWCLVALY------------------ : 101
BrCNGC18 : ------KILDPGGDVVLQWNRVFLFWCLVALY------------------ :  97
BrCNGC19 : ----LGRILDPRSKLVQEWNRVFLLVCATGLF------------------ : 104
BrCNGC20 : ----LGRILDPRSKLVQEWNKVFLLVCATGLF------------------ : 104
BrCNGC21 : LKGPFGEVLDPRSKRVQRWNRALLLARGMALA------------------ : 131
BrCNGC22 : VDKYLPKIMEPDSKEVGGWTIFFAFSCLLSIF------------------ : 134
BrCNGC23 : VNRHLPGIMNPHSIFIQSWTRLFSLSCLLSIF------------------ : 220
BrCNGC24 : VNRCLPGIMNPHSKFVQIWTKFFAVSSLIAIF------------------ : 180
BrCNGC25 : VRRCVPGIMNPHSKFVQVWTRFLAFSCLMAIF------------------ : 192
BrCNGC26 : VRRCVPGIINPHSKFVQMWTRFLAFSCLLSIF------------------ : 209
BrCNGC27 : ---YFSGIIYPESNYVQLWTTFFVLSCLCSIF------------------ : 174
BrCNGC28 : ANRYLPGIMNPHSKFVQSWTKFFALSCLIAIF------------------ : 213
BrCNGC29 : VRKMVPGIMNPHSKFVQMWTRFLAFSCLVAIF------------------ : 206
                       p        w       c  6
```

```
                             260         *         280         *       300
BrCNGC1  : --------------VDPLFLFIPVIDS-PN-FCFTFDKKLAAVVSAIRTF :  81
BrCNGC2  : IFESNYANCYHQATVDPLFFFIPVIDS-HK-FCFTLDKKLGVAVCVLRTL : 126
BrCNGC3  : --------------FDPLFFFIPVVDP-DR-FCLKLDKKLEAVACVFRTF : 128
BrCNGC4  : --------------IDPLFLFIPVIDS-HR-LCFTYDKKLVATACVFRTL :  53
BrCNGC5  : --------------IDPLFFYIPIVDG-KK-HCINLHSSLEIAASVLRTF : 133
BrCNGC6  : --------------VDPLFFYVPVLDK-AN-NCLDVDKKMQTTASVLRSV : 169
BrCNGC7  : --------------LDPLFFYVPVVDD-AK-KCLGLDNKMEITASVLRSF : 137
BrCNGC8  : --------------VDPLFFYLPIVDN-SGSSCIGIDTKLAVTTTTLRTI : 119
BrCNGC9  : --------------VDPFFFYLPVIDG-ES-KCLGIDRKLAITATTFRTF : 175
BrCNGC10 : --------------VDPLFLYLPFIND-KA-KCIGIDRKLAIVATTLRTV : 160
BrCNGC11 : --------------VDPLFLFLPFIND-KA-KCVGIDRKLAIVTTTLRTV : 150
BrCNGC12 : --------------VDPLFLYLPFIND-SG-KCIGIDRRLATIATTLRTF : 138
BrCNGC13 : --------------VDPLFFFLPVMRK-EA--CITIGIRLEVVLTVIRSL : 132
BrCNGC14 : --------------LDPFYFYAPYVGG-PA--CLSVDVGLAATVTFFRSV :  97
BrCNGC15 : --------------LDPLYFYPPIVQA-GT-ACMSIDIGFGILVTFFRTL : 103
BrCNGC16 : --------------IDPLYFFVPAIGGNKDYFCAKTDTNLRILVTFFRTI : 134
BrCNGC17 : --------------VDPLFFFLSSVKNTGRSSCMTTDLKLGIVITFFRTL : 137
BrCNGC18 : --------------VDPLFFFLYSVKRTGRSSCMTTDLNLGIVVTFFRTL : 133
BrCNGC19 : --------------VDPLFLYTLSVND-AC-MCLLVDGWLALTVTALRSM : 138
BrCNGC20 : --------------VDPLFLYTISVND-AC-MCLLVDGWLALSITAVRSM : 138
BrCNGC21 : --------------VDPLFFYALSIGRTTGPACLYMDGAFAAVVTVVRTC : 167
BrCNGC22 : --------------VDPLFFFPIQVSE-SG-KCIRIDEKMARVLVAIRSL : 168
BrCNGC23 : --------------LDPLFVFLTLVKQ-NN-KCIVIDWPMAKAFIIVRSV : 254
BrCNGC24 : --------------IDPLFFFIILVQK-NN-KCIVIDWPIATAFVIVRTL : 214
BrCNGC25 : --------------IDPTFVFLLLIRH-DN-KCIEIDWPKTTVLVSIRSM : 226
BrCNGC26 : --------------IDPAFVYLLLIRD-DN-KCTDIDWPKATLFLFIRSM : 243
BrCNGC27 : --------------VDPLFFYPIEIYK-EE-RCIKIDWWTTNVFVIVRTI : 208
BrCNGC28 : --------------IDPLFFFLILVKQ-ND-KCIVIDWPMAKAFVAVRSV : 247
BrCNGC29 : --------------IDPAFLFLLSIRQ-DN-KCIEFDWPKTKVFVSLRSM : 240
                             DP15 5   6       C   d          R3
```

```
                    *       320         *       340         *
BrCNGC1  : IDTFYVIHIIFNFITE----FIAPRS---QVSLRGELIVHSKAIRKRLFF : 124
BrCNGC2  : IDVFYVIHFIFHFITE----LVAPRS---QASLRG----NSKPIRKRLFF : 165
BrCNGC3  : IDAFYLVHMLFQFNTG----FIAPSS---RGFGRGELVQSSKKIAVRYLK : 171
BrCNGC4  : IDTFYGIHIIFHFITK----HIAPRS---QVSFRGETTVYSVAISERHLI :  96
BrCNGC5  : VDAFYIIHIVFQFRTA----YVSPLS---RVFGRGELVEDPKAIALKYIS : 176
BrCNGC6  : TDIFYAIHMVFQFRTG----FIAPSS---RVFGRGVLVEDRRKIAKRYLS : 212
BrCNGC7  : TDIFYVIHIIFQFRTG----FIAPSS---RVFGRGVLVEDTRQIAIRYLS : 180
BrCNGC8  : LDVFYLTRMALQFRTA----YIAPSS---RVFGRGELVIDPAKIAQRYLT : 162
BrCNGC9  : IDVFYLAHMALQLRTA----YIAPSS---RVFGRGELVIDPAQIAKRYLQ : 218
BrCNGC10 : IDSFYLFHMALRFRTA----YVAPSS---RVFGRGELVIDPKQIAKRYLR : 203
BrCNGC11 : IDSFYLFHMALRFRTA----YVAPSS---RVFGRGELVIDPAQIAKRYLQ : 193
BrCNGC12 : IDVFYLFRMALQFRTA----FVAPSS---RVFGRGELVIDPAQIAKRYLQ : 181
BrCNGC13 : ADAFYIAQIVIRFRTA----YIAPSS---RVFGRGELVIDSRKIAWRYLN : 175
BrCNGC14 : ADLFHLLHIFMKFRTA----FVARSS---RVFGRGELVRDPREIAMKYLK : 140
BrCNGC15 : ADFSFLIHILLKFKTA----FVSKSS---RVFGRGELVIDRREIAIRYLK : 146
BrCNGC16 : ADLFYLLHIFIKFRTG----FIAPNS-STRVFGRGELVMDPKAIAWRYLK : 179
BrCNGC17 : ADLFYVLHIVIKFRTA----YVSRTS---RVFGRGELVKDPKLIARRYLR : 180
BrCNGC18 : ADLFYVLHIVIKFRTA----YVSRTS---RVFGRGELVKDPKLIARRYLR : 176
BrCNGC19 : TDLLHLWNILIQFKIARWWPYRGGDSDGDINKGDGTRVR--MRGAPPYVK : 186
BrCNGC20 : TDLLHLWNIWIQFKIARRWPYPGGDSDGDTNKGDETRLRTSRRVAPPYVK : 188
BrCNGC21 : LDALHLWHVWLQFRLA----YVSRES---LVVGCGKLVWDPRAIASHYAR : 210
BrCNGC22 : TDFLYFVNTLLQCRLA----YTDPKS---TVVGSGQLVKGSVEIAKRYYR : 211
BrCNGC23 : TDALFSVNILLQFRLA----YVSPES---MVVGVTWLVDHPVKIARHYFQ : 297
BrCNGC24 : TDVIFFANMLLQFRLA----YVARES---TVVGAGQLV------------ : 245
BrCNGC25 : FDLIFFINILLQFRMA----YVAPES---RIVGAGQLVDHPRKIARNYLR : 269
BrCNGC26 : SDFIFFINILLQFRLA----YVAPES---RIVGAGQLVDHPRKIACNYLR : 286
BrCNGC27 : TDGLYALNIVLQFRLA----YVDLES---TVAGAGQLVDDPKKIASHYLR : 251
BrCNGC28 : TDILFSVNILLQFRLA----YVAPES---TVVGAGQLVAHPRKIARHYFR : 290
BrCNGC29 : SDLIFFMNILLQFRLA----YVAPES---RIVGAGQLVDHPRKIASNYLR : 283
            D          f            S      g g l       a  y
```

```
                        360         *         380         *         400
BrCNGC1  : FQ--FIVDICSVIPI----------------------------PQVVVLI : 144
BrCNGC2  : FY--FSVDIVSVLPI----------------------------PQVMVLT : 185
BrCNGC3  : SY--FIIDVLSILPI----------------------------PQVVVLA : 191
BrCNGC4  : FY--FIVDIVSVLPI----------------------------PQ----- : 111
BrCNGC5  : SY--FIIDVLSILPL----------------------------PQLVVLA : 196
BrCNGC6  : SH--FIIDILAVLPL----------------------------PQMVISI : 232
BrCNGC7  : SH--FIIDILAVLPLPQVRIEIEIEIDLVLSTCISKPLMIVCFPQVVILI : 228
BrCNGC8  : RY--FIVDFLAVLPL----------------------------PQIAVWK : 182
BrCNGC9  : RW--FIIDFLSVLPV----------------------------PQIVVWR : 238
BrCNGC10 : QY--FIIDLLSVLPL----------------------------PQITVWR : 223
BrCNGC11 : QY--FIIDLLSVLPV----------------------------PQIIVWR : 213
BrCNGC12 : QY--FIVDFLSVLPL----------------------------PQIVVWR : 201
BrCNGC13 : KS--FWIHLVAALPI----------------------------PQVLIWI : 195
BrCNGC14 : SD--FIVDVAAMLPI----------------------------PQLVIWL : 160
BrCNGC15 : SE--FIIDLAATLPI----------------------------PQIMIWF : 166
BrCNGC16 : SD--FIIDLVATLPI----------------------------PQIVIWF : 199
BrCNGC17 : SD--FIVDLIACLPI----------------------------PQIVSWF : 200
BrCNGC18 : SD--FIVDLIACLPI----------------------------PQIVSWF : 196
BrCNGC19 : KN-GFFFDLFVILPL----------------------------PQVVLWV : 207
BrCNGC20 : KKGTFFFDLFVILPL----------------------------PQVVLWV : 210
BrCNGC21 : SLTGFWFDVIVILPV----------------------------PQAVFWL : 232
BrCNGC22 : GN--FLLDLVIVIPL----------------------------PQILILW : 231
BrCNGC23 : GN--FFLDLFIVMPL----------------------------PQILISW : 317
BrCNGC24 : ----------------------------------------------LVLS : 249
BrCNGC25 : GK--FFLDLFIVLPI----------------------------PQIMTLS : 289
BrCNGC26 : GK--FFLDVVIVYPV----------------------------PQLMILS : 306
BrCNGC27 : GK--FLTDFFIVLPI----------------------------PQILILW : 271
BrCNGC28 : GK--FLLDLFIVMPL----------------------------PQILILW : 310
BrCNGC29 : GK--FLLDLLIVLPM----------------------------PQIMILS : 303
               f  d     p                            pq
```

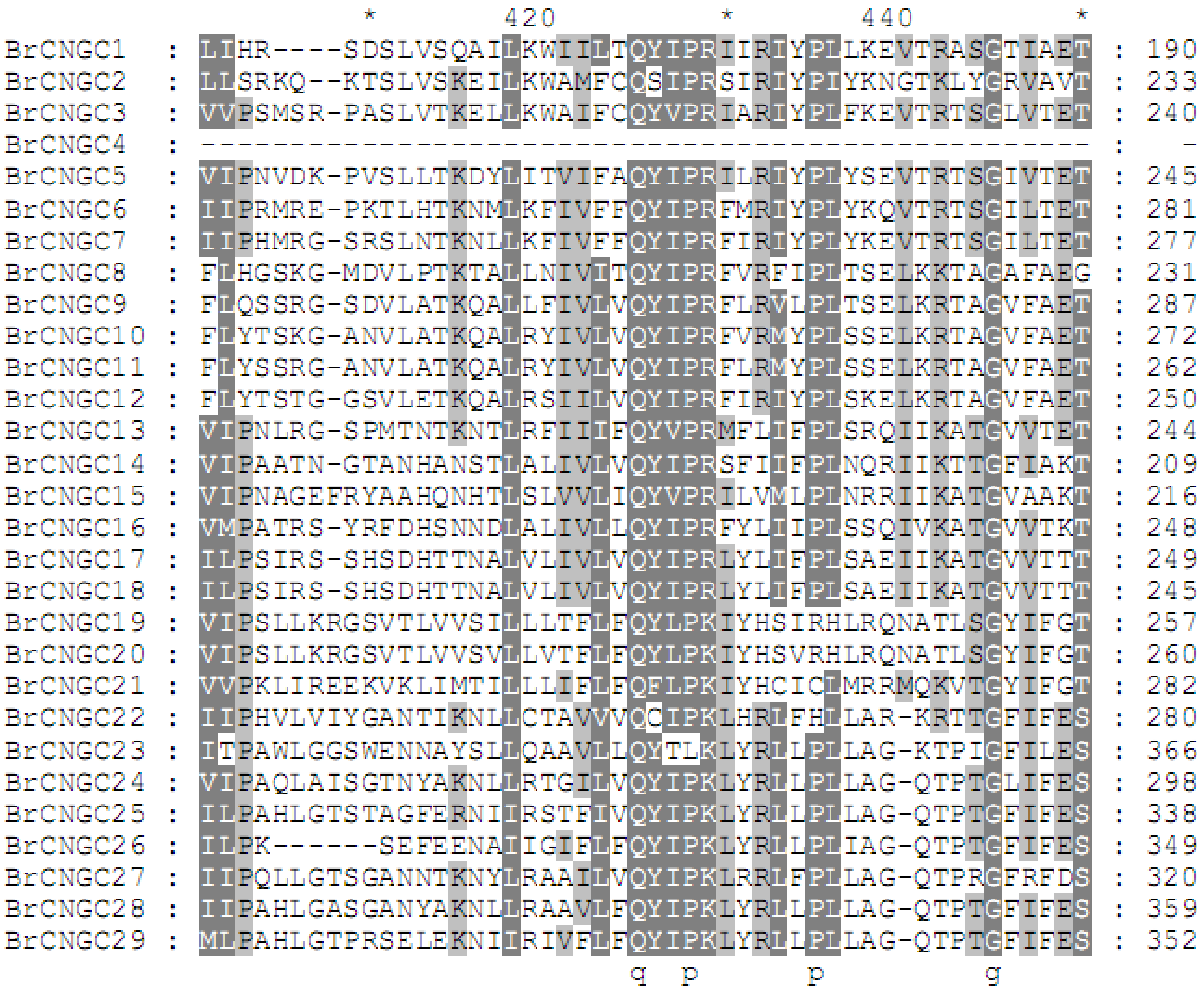

* 420 * 440 *
BrCNGC1 : LIHR----SDSLVSQAILKWIILTQYIPRIIRIYPLLKEVTRASGTIAET : 190
BrCNGC2 : LLSRKQ--KTSLVSKEILKWAMFCQSIPRSIRIYPIYKNGTKLYGRVAVT : 233
BrCNGC3 : VVPSMSR-PASLVTKELLKWAIFCQYVPRIARIYPLFKEVTRTSGLVTET : 240
BrCNGC4 : -- : -
BrCNGC5 : VIPNVDK-PVSLLTKDYLITVIFAQYIPRILRIYPLYSEVTRTSGIVTET : 245
BrCNGC6 : IIPRMRE-PKTLHTKNMLKFIVFFQYIPRFMRIYPLYKQVTRTSGILTET : 281
BrCNGC7 : IIPHMRG-SRSLNTKNLLKFIVFFQYIPRFIRIYPLYKEVTRTSGILTET : 277
BrCNGC8 : FLHGSKG-MDVLPTKTALLNIVITQYIPRFVRFIPLTSELKKTAGAFAEG : 231
BrCNGC9 : FLQSSRG-SDVLATKQALLFIVLVQYIPRFLRVLPLTSELKRTAGVFAET : 287
BrCNGC10 : FLYTSKG-ANVLATKQALRYIVLVQYIPRFVRMYPLSSELKRTAGVFAET : 272
BrCNGC11 : FLYSSRG-ANVLATKQALRYIVLVQYIPRFLRMYPLSSELKRTAGVFAET : 262
BrCNGC12 : FLYTSTG-GSVLETKQALRSIILVQYIPRFIRIYPLSKELKRTAGVFAET : 250
BrCNGC13 : VIPNLRG-SPMTNTKNTLRFIIIFQYVPRMFLIFPLSRQIIKATGVVTET : 244
BrCNGC14 : VIPAATN-GTANHANSTLALIVLVQYIPRSFIIFPLNQRIIKTTGFIAKT : 209
BrCNGC15 : VIPNAGEFRYAAHQNHTLSLVVLIQYVPRILVMLPLNRRIIKATGVAAKT : 216
BrCNGC16 : VMPATRS-YRFDHSNNDLALIVLLQYIPRFYLIIPLSSQIVKATGVVTKT : 248
BrCNGC17 : ILPSIRS-SHSDHTTNALVLIVLVQYIPRLYLIFPLSAEIIKATGVVTTT : 249
BrCNGC18 : ILPSIRS-SHSDHTTNALVLIVLVQYIPRLYLIFPLSAEIIKATGVVTTT : 245
BrCNGC19 : VIPSLLKRGSVTLVVSILLLTFLFQYLPKIYHSIRHLRQNATLSGYIFGT : 257
BrCNGC20 : VIPSLLKRGSVTLVVSVLLVTFLFQYLPKIYHSVRHLRQNATLSGYIFGT : 260
BrCNGC21 : VVPKLIREEKVKLIMTILLLIFLFQFLPKIYHCICLMRRMQKVTGYIFGT : 282
BrCNGC22 : IIPHVLVIYGANTIKNLLCTAVVVQCIPKLHRLFHLLAR-KRTTGFIFES : 280
BrCNGC23 : ITPAWLGGSWENNAYSLLQAAVLLQYTLKLYRLLPLLAG-KTPIGFILES : 366
BrCNGC24 : VIPAQLAISGTNYAKNLLRTGILVQYIPKLYRLLPLLAG-QTPTGLIFES : 298
BrCNGC25 : ILPAHLGTSTAGFERNIIRSTFIVQYIPKLYRLLPLLAG-QTPTGFIFES : 338
BrCNGC26 : ILPK------SEFEENAIIGIFLFQYIPKLYRLLPLIAG-QTPTGFIFES : 349
BrCNGC27 : IIPQLLGTSGANNTKNYLRAAILVQYIPKLRRLFPLLAG-QTPRGFRFDS : 320
BrCNGC28 : IIPAHLGASGANYAKNLLRAAVLFQYIPKLYRLLPLLAG-QTPTGFIFES : 359
BrCNGC29 : MLPAHLGTPRSELEKNIIRIVFLFQYIPKLYRLLPLLAG-QTPTGFIFES : 352
q p p g

```
                          460         *         480         *         500
BrCNGC1   : KWVGAAFNLFLYMLHSHVFGAFWYVSSVEKKNKCWRLEC-AK------IS : 233
BrCNGC2   : KWVGAALNLFFYLLPSHVIGAIWYLSAVEKKETCWREAC-AK------ID : 276
BrCNGC3   : AWAGAALNLFLYMLASHVFGSFWYLISIERKDRCWRETC-AK------IE : 283
BrCNGC4   : -----------------VFGAFWYLSAIEKKNRCWSDAC-AK------SS : 137
BrCNGC5   : AWAGAAWNLSLYMLASHVFGALWYLISVEREDRCWREAC-GK------RQ : 288
BrCNGC6   : AWAGAAFNLFLYMLASHVFGAFWYLFSIERKTVCWKQAWNRAG-----RS : 326
BrCNGC7   : AWAGAAFNLFLYMLASHVFGAFWYLFSIERETVCWKQAC-NRN-----RN : 321
BrCNGC8   : AWAGAAYYLLWYMLASHITGAFWYMLSVERNDTCWRFACKVQP----DPK : 277
BrCNGC9   : AWAGAAYYLMLYMLASHIVGAFWYLLALERNDACWQEACSDAG-----KK : 332
BrCNGC10  : AWAGAAYYLLLYMLASHIVGALWYLLALERNNDCWSKAC-VK------KD : 315
BrCNGC11  : AWAGAAYYLLLYMLASHIVGALWYLLALERNNDCWSKAC-KD------ND : 305
BrCNGC12  : AWAGAAYYLLLYMLASHIVGALWYLLALERVNGCWKKACLVD------GQ : 294
BrCNGC13  : AWAGAAYNLMLYMLASHVLGACWYLLAVERQEACWRHAC-NIE-----KP : 288
BrCNGC14  : AWAGAAYNLLLYILASHVLGAMWYLSSIGRQFSCWSKVC-EKD-HALRVL : 257
BrCNGC15  : AWSGAAYNLVLYLLVSHVLGSVWYVLSIQRQHECWRREC-IKEMNATHSP : 265
BrCNGC16  : AWAGAAYNLLLYMLASHVLGAAWYILSFDRYTSCWKTRC-NKE---HGGV : 294
BrCNGC17  : AWAGAAYNLLQYMLASHILGAAWYLLSIERQATCWKAEC-HNE---LGPI : 295
BrCNGC18  : AWAGAAYNLLQYMLASHILGAAWYLLSIQRQATCWKAEC-HKE---FAPL : 291
BrCNGC19  : VWWGFALNMVAYFVAAHAAGACWYLLGVQRSAKCLKEQC-EN------TM : 300
BrCNGC20  : VWWGIALNMIAYFVAAHAAGACWYLLGVQRSAKCLTEQC-ES------TM : 303
BrCNGC21  : IWWGFALNLIAYFIASHVAGGCWYVLAIQRVASCIRQQC-MR------TA : 325
BrCNGC22  : AWASFFINLFTYILAGHIVGSCWYLFGLQRVNRCLRETC-GDHMESKCHG : 329
BrCNGC23  : S-SKFAINFLTFMLAGHVVGSCWYLFGLQRVNQCLRDAC-GNT-----DR : 409
BrCNGC24  : AWANFVINLLTFMLAGHVVGSCWYLFGLQRVNQCLRNAC-GHS-----GR : 342
BrCNGC25  : AWASFVINLLTFMLAGHAVGSCWYFSGIERVKKCLLYAAWNNS-----VD : 383
BrCNGC26  : AWSNFIINLLTFILAGHAVGSFWYLAGLQRVNKCLLQAG---------NF : 390
BrCNGC27  : ALAKFFINLLTFMLAGHVIGSCWYLLGLQRVNQCLRDAC-GNS-----SF : 364
BrCNGC28  : AWANFVINLLTFMLAGHVVGSCWYLFGLQRVNQCLRDAC-GNS-----DH : 403
BrCNGC29  : AWANFIINLLTFILAGHAIGSFWYIAGLQRVKKCLLHAG---------NY : 393
             w     n       a h  G  WY      4   C    c
```

```
                       *        520          *        540          *
BrCNGC1   : GCNLRH--QYCA-----------R-GRENNGR------------YLNTTC : 257
BrCNGC2   : ECDLTN--LLCA-----------R-GAGGDNS---R--------FLNTSC : 301
BrCNGC3   : GCVHGN--LYCS-------------GGEDNSQ------------YLIGSC : 306
BrCNGC4   : MCNLTNLDLYCV-----------R-GGGDNSH------------FLKISC : 163
BrCNGC5   : GCELRD--LYCD-------------GNNNVIN---D--------YLTTSC : 312
BrCNGC6   : KCDMRS--LYCA-----------R-EHYGNNT------------FLNGSC : 350
BrCNGC7   : ICDITS--LYCD-----------H-KAAGGNA------------FLNASC : 345
BrCNGC8   : LCVQI---LYCG-----------T-KFVSSRE---TEWIKTVPELLKSNC : 309
BrCNGC9   : ICTTGF--LYCG-----------N-QNMDGYD---V-WNKTKEAVLESRC : 364
BrCNGC10  : NCTRNF--LFCG-----------N-QNMEGYA---A-WYTAKSSVLQEMC : 347
BrCNGC11  : NCTRNF--LFCG-----------N-QNMKGYD---A-WDDVKDPFLQLRC : 337
BrCNGC12  : NCTRNF--LFCG-----------N-ENMDGYA---A-WNTIKESVLQKSC : 326
BrCNGC13  : ICQYRF--FECR-----------R-LEDPQRN---S--------WFEWSN : 313
BrCNGC14  : DCLPSF--LDCK-----------S-LEQPERQ---Y--------WQNVTQ : 282
BrCNGC15  : SCNLLF--LDCG-----------S-LRDPGRQ---A--------WMRITR : 290
BrCNGC16  : NCYLYY--LDCD-----------SPLYDARQQ---Q--------WANVTN : 320
BrCNGC17  : RCVTDF--FDCG-----------T-VNREDRN---N--------WQNVTV : 320
BrCNGC18  : ECVTDF--FDCG-----------T-LHRPDRN---N--------WQNITV : 316
BrCNGC19  : GCDLRM--LSCK-----------EPVYYGTTE---MVLDRARLAWARNHQ : 334
BrCNGC20  : GCDLRM--LSCK-----------EPVYYGTTE---MVLDRARLAWAQNNQ : 337
BrCNGC21  : NCNLS---LSCQ-----------EEVCYQFVSPSSTIGFPCVSGNLTSVV : 361
BrCNGC22  : VCKEL---IDCGLRLKEKLIDCER-RNTTVTQ---A--------VLNWNV : 364
BrCNGC23  : ACREL---IDCG-----------R-GSSDVVL---A--------ALKYNT : 433
BrCNGC24  : ECRGL---IDCG-----------H-GNSNISASLRA--------IWRNSA : 369
BrCNGC25  : ERRNL---IDCA-----------R-GNIYASASLRA--------RWRDSD : 410
BrCNGC26  : TMDERRYLIDCT-----------S-GESYLRA------------LWRDSE : 416
BrCNGC27  : ECKQL---IDCG-----------R-ENRTEVL---H--------AWKINV : 388
BrCNGC28  : ECRNL---IDCG-----------R-GESSEAF---A--------AWKGNA : 427
BrCNGC29  : TMDERRNLIDCA-----------H-GVNYARESQIA--------LWRDSE : 423
              c        C
```

```
                   560         *       580         *       600
BrCNGC1   : PLIDPDQ-IIGSTVFNFGMYTDALRSGIVESKPRDFPRKFFYCFW----- : 301
BrCNGC2   : PLIDPEQ-ITNSTVLNFGIYTDALKSGVVET--RDFPRKLLYCFW----- : 343
BrCNGC3   : PLMDPEE-IKNSTVFNFGIFAEALQSGVVES--MNFPKKFFYCFW----- : 348
BrCNGC4   : PLIDPGE-ITNSTVFNFGMYIDALKSGVVES--RDFPRKFFYCFW----- : 205
BrCNGC5   : PFINPDD-ITNSTTFNFGIFTDALKSGIVKS--DDFWKKFFYCFW----- : 354
BrCNGC6   : PVLKPNA-----TCFEFGIFLGALESGVVES--HDFPQKFFYCFW----- : 388
BrCNGC7   : PVQTPNA-----TLFDFGIFLNALQSGVVES--QDFPQKFFYCFW----- : 383
BrCNGC8   : SAKADDA------KFNYGIYGQAISSGIVSS--TTFFSKFCYCLW----- : 346
BrCNGC9   : RADLDDP----NPPFDFGIYTQALSSGIVSS--QKFITKYCYCLW----- : 403
BrCNGC10  : PVNVTEG---EEPPFDFGIYSRALSSGIVSS--KKFVSKYFFCLW----- : 387
BrCNGC11  : PVNVTEG---EEPPFDFGIYLRALSSGIVSS--KKFVSKYFFCLW----- : 377
BrCNGC12  : PVNVTDG---DNPPFDFGIYLRALSSGIVSS--ESFVAKYFFCLW----- : 366
BrCNGC13  : ITTICKP---GTRFYEFGIYGDAVTSTVTSS---NFISKYFYCLW----- : 352
BrCNGC14  : VLSHCDA-TSSTTNFKFGMFAEAFTTQVATT---DFVSKYLYCLW----- : 323
BrCNGC15  : VLSNCDARNDDDQHFQFGMFGDAFTNDVTSS---PFFDKYFYCLW----- : 332
BrCNGC16  : VFKLCDA---RKGEFKYGMFENAITKKVVSS---NFNERYFYCLW----- : 359
BrCNGC17  : VFSNCDP--SNKIRFTFGIFADALTKNVVSS---PFLEKYLYCLW----- : 360
BrCNGC18  : VFSNCDP--SNDIKFTFGIFADALTKNVVSS---PFLEKYLYCLW----- : 356
BrCNGC19  : ARSVCLD---IDTDYTYGAYQWTIQLVSNES----RLEKVLFPIF----- : 372
BrCNGC20  : ARSICLD---INTNYTYGAYKWTIQLVSNES----RLEKILFPIF----- : 375
BrCNGC21  : KKPMCLD---SDGPFRYGIYGWALPVISSNS----LAVKILYPIF----- : 399
BrCNGC22  : TAAACFQ----ENGFPYGIYWKAVNLTHDSN----LPKKYIYSLF----- : 401
BrCNGC23  : SASACFQ----ENGFPYGIYLKAVNLTNQSS-LITIHKIHLLSFLGFSAN : 478
BrCNGC24  : SANACFQ----EDGFSYGIYLKMVNLTTHTS----LLTRYSYSLL----- : 406
BrCNGC25  : SVNACFQ----ESGFSYGIYAKAVNLTSHSS----IFTRFSYSLF----- : 447
BrCNGC26  : SVNACFQ----DGGFSFGIYLNAANLTTQRN----IFTRFSFSLF----- : 453
BrCNGC27  : SANACFQ----EDGFDYGIYLKAVNLTSNRT---RWYRRYSYSLF----- : 426
BrCNGC28  : SASACFQ----EGGFPYGIYMKAVNLTNHTS----LFTRYSYSLF----- : 464
BrCNGC29  : SVNACFQ----EGGFSFGIYLKAVNLTTQPN----IFTRFSYSLF----- : 460
                            5G 5  a
```

```
                    *        620         *         640          *
BrCNGC1  : -----WGL---------------------------RNISALGQNLKTSNS : 319
BrCNGC2  : -----WGL---------------------------RNISALGQNLKTSNS : 361
BrCNGC3  : -----WGL---------------------------RNLSAVGQNLKTSDF : 366
BrCNGC4  : -----WGL---------------------------RNLSALGQNLETSNS : 223
BrCNGC5  : -----WGL---------------------------RNLSALGQNLNTSKF : 372
BrCNGC6  : -----WGL---------------------------QNLSSLGQNLKTSTY : 406
BrCNGC7  : -----WGL---------------------------QNLSSLGQNLKTSTY : 401
BrCNGC8  : -----WGL---------------------------QNLSTLGQGLQTSTF : 364
BrCNGC9  : -----WGL---------------------------QNLSTLGQGLETSTY : 421
BrCNGC10 : -----WGL---------------------------QNLSTLGQGLETSTY : 405
BrCNGC11 : -----WGL---------------------------QNLSTLGQGLETSTY : 395
BrCNGC12 : -----WGL---------------------------QNLSTLGQGLETSTY : 384
BrCNGC13 : -----WGL---------------------------KNLSSLGQNLSTSTY : 370
BrCNGC14 : -----WGL---------------------------RNLSSYGQNITTSVY : 341
BrCNGC15 : -----WGL---------------------------RNLSSYGQSLAASTL : 350
BrCNGC16 : -----WGL---------------------------QQLSSYGQNLSTTTF : 377
BrCNGC17 : -----FGL---------------------------QQLSSYGQNLDTSTS : 378
BrCNGC18 : -----FGL---------------------------QNLSSYGQNLDTSTS : 374
BrCNGC19 : -----WGL---------------------------MTLSTFG-NLESTTE : 389
BrCNGC20 : -----WGL---------------------------MTLSTFG-NLESTTE : 392
BrCNGC21 : -----WGL---------------------------MTLSTFGNDLEPTSN : 417
BrCNGC22 : -----WGF---------------------------QQISTLAGNLVPTDF : 419
BrCNGC23 : LIRMFYGFDIKSALVGICGSLTLAYCQLSIVYYSPKQISTLAGNQVPSYF : 528
BrCNGC24 : -----WGF------------------------------------------ : 409
BrCNGC25 : -----WGF---------------------------QQISTLAGNLSPSYS : 465
BrCNGC26 : -----WGF---------------------------QQISTLAGNQTPSYS : 471
BrCNGC27 : -----WGF---------------------------QQISTLAGNQVPSYF : 444
BrCNGC28 : -----WGF---------------------------QQISTLAGNQVPSYF : 482
BrCNGC29 : -----WGF---------------------------QQISTLAGNQTPTYS : 478
                5G                                 s
```

```
                  660         *       680         *       700
BrCNGC1  : VGDIVFALIICVSGLLLFAVLIGNIQKYLQSTTIRLDEMEEKKRDTEKWM : 369
BrCNGC2  : AGEVFFAIIICVSGVLLFAGLIGNVQKYLQSTSIRVDEWEAKKRDTEQWM : 411
BrCNGC3  : EGEIIFAIIICISGLVLFALLIGNMQKYLQSTTVRIEEMRVKRRDAEQWM : 416
BrCNGC4  : VGEIFFAITICVSGLLLFSVLIGNVQKYLQSTIVRINEMEEKRRDTEKWM : 273
BrCNGC5  : VGEIIFAVLICISGLVLFALLIGNMQKYLESTTVREEEMRVRKRDAEQWM : 422
BrCNGC6  : IWENCFAVFISISGLVLFSFLIGNMQTYLQSTTTRLEEMRVKRRDAEQWM : 456
BrCNGC7  : IWEICFAVFISISGLVLFSFLIGNMQTYLQSTTTRLEEMRVKRRDAEQWM : 451
BrCNGC8  : PGEVLFSIAIAIAGLLLFALLIGNMQTYLQSLTVRLEEMRIKRRDSEQWM : 414
BrCNGC9  : PLEIMFSITLAISGLILFALLIGNMQTYLQSLTIRLEEMRVKRRDSEQWM : 471
BrCNGC10 : PGEVIFSIALAIAGLLLFALLIGNMQTYLQSLTIRLEEMRVKRRDSEQWM : 455
BrCNGC11 : PGEVIFSIVLAIAGLLLFALLIGNMQTYLQSLTIRLEEMRVKRRDSEQWM : 445
BrCNGC12 : PGEIIFSIALAVAGLLLFALLIGNMQTYLQSLTIRLEEMRVKRRDSEQWM : 434
BrCNGC13 : VGEIIFAVVMATLGLVLFALLIGNMQTYLQSTTMRLEEWRIRRTDTEQWM : 420
BrCNGC14 : LGETLFCITICIFGLILFTLLIGNMQTSLQSMSVRVEEWRIKRRDTEEWM : 391
BrCNGC15 : SSETLFSCFICVAGLVFFSHLIGNVQNYLQSTTARLDEWRVRRRDTEEWM : 400
BrCNGC16 : IGETTFAVLIAIFGLVLFAHLIGNMQTYLQSLTVRLEEWRLKKRDTEEWM : 427
BrCNGC17 : VLETMFAILVAIFGLVLFALLIGNMQTYLQSITVRLEEWRLKRRDTEEWM : 428
BrCNGC18 : VLETMFAILVAIFGLVLFALLIGNMQTYLQSITVRLEEWRLKRRDTEEWM : 424
BrCNGC19 : WSEVVFNIIVLTSGLLIVTMLIGNIKVFLHATTSKKQAMHLKMRNIEWWM : 439
BrCNGC20 : WSEVVFNIIVLTSGLLIVTMLIGNIKVFLHATTSKKQAMHLKMRNIEWWM : 442
BrCNGC21 : WLEVVFSIVMVLSGLLLFTLLIGNIQVFLHAVMAKKRKMQIRCRDMEWWM : 467
BrCNGC22 : VWEVLFTMAIIGLGIFLFALLIGNMQNFLQSLGRRNMEMTLRRRDVNNWM : 469
BrCNGC23 : IWEVLFTMGIIGLGLLLFAFLIGNMQNFLQTLGQRNLEMTLRQRDAEQWM : 578
BrCNGC24 : -------------------------QVMVYSNNVWSLEMTLRRRDVEQWM : 434
BrCNGC25 : VGEVFFTMGITGLGLLLFARLIGNMHNFLQALDRRRMEMMLRRRDVEQWM : 515
BrCNGC26 : VGEVYFTMGIIGLGLLLFARLIGNMHNFLQALDRRRTEMMLRQRDVEQWM : 521
BrCNGC27 : FGEVLFTMCIIGLGIFLFALLIGNMQNFLQSLGRRDTEMTVRRRDVEQWM : 494
BrCNGC28 : LGEVFFTMGIIGLGLLLFALLIGNMQNFLQALGRRNLEMTLRRRDVEQWM : 532
BrCNGC29 : VGEVYFTMGIIGLGLLLFARLIGNMHNFLQALDRRRMEMMLRRRDVEQWM : 528
             e  f       g  lf  lign q  6        e   4 r1 e WM
```

```
                    *         720         *         740         *
BrCNGC1  : SNRMLPEYLKERIRRYENYKWRKTRGIEEEALLHSLPKDIRLETKRHLYL : 419
BrCNGC2  : PSEDLPDDLEKCI------EWEKNSSIEREAHLRSLPKDIRVEAKRNLYL : 455
BrCNGC3  : SHRMLPDDLRKRVREYEQYKWQETRGVEEEALLSSLPKDIRKEIKRHLCL : 466
BrCNGC4  : SYRMLPESLKERIRKSEDHKWRETRGTKEESFLRGLPKNIRLEIERQIYQ : 323
BrCNGC5  : AHRMLPEDLRKRIRRYEQYKWQETRGVEEENLLRNLPKDIRRDIKRHFCL : 472
BrCNGC6  : SHRLLPDNLRKRIRRYEQYKWQETRGVNEENLLSNLPKDIRRDIKRHLCL : 506
BrCNGC7  : AHRLLPESLRKRIRRYEQYKWQETRGVDEENLLSNLPKDIRRDIKRHLCL : 501
BrCNGC8  : HHRSLPQNLRERVRRYDQYKWLETRGVDEENIVQSLPKDIRRDIKRHLCL : 464
BrCNGC9  : HHRMLPQDLRQRVRRYDQYKWLETRGVDEEYLVQNLPKDIRRDIKRHLCL : 521
BrCNGC10 : HHRMLPPELRERVRRYDQYKWLETRGVDEENIVSNLPKDIRRDIKRHLCL : 505
BrCNGC11 : HHRMLPPELRERVRRYDQYKWLETRGVDEENLVSNLPKDIRRDIKRHLCL : 495
BrCNGC12 : HHRMLPPDLRERVRRYDQYKWLETKGVDEENLVQNLPKDIRRDIKRHLCL : 484
BrCNGC13 : HHRQLPPELRQAVRKYDQYKWLATRGVDEEALLISLPLDIRRDIKRHLCF : 470
BrCNGC14 : RHRQLPPELQERVRRFVQYKWLATRGVDEESILQSLPTDIRREIQRHLCL : 441
BrCNGC15 : RHRQLPQELQERVRRFVQYKWLTTRGVDEEAILRALPLDIRRQIQRHLCL : 450
BrCNGC16 : RHRQLPEDLRDRVRRYEQYKWLAARGVDEEVLLQSLPTDIRRDIQRHLCL : 477
BrCNGC17 : GHRQLPQNLRERVRRFVQYKWLATRGVDEETILHSLPADIRRDIQRHLCL : 478
BrCNGC18 : GHRQLPQDLRERVRRFVQYKWLATRGVDEETILHSLPADIRRDIQRHLCL : 474
BrCNGC19 : KKRHLPLGFRQRVRNYERQRWAAMRGVDECEMVQNLPEGIRRDIKYHLCL : 489
BrCNGC20 : KKRQLPLGYRQRVRNYERQRWAAMRGVDECEMVQNLPEGIRRDIKYHLCL : 492
BrCNGC21 : KRRQLPSRLRQRVRRFERQRWTALGGEDELELIQDLPPGIRRDIKRYLCV : 517
BrCNGC22 : SHRQFPDDIRTRVKNAERLNWSSTRGVNEELLFENMSDDLQRDIRRHL-F : 518
BrCNGC23 : SHRRFPEGIRKRVREVERFHWAARRGVDEELLFNNMPDDLQRDIRRHL-F : 627
BrCNGC24 : SHRRLPEGIRKRVREAERFNWAATRGVNEELLFENMPDDLQGDIRRHL-F : 483
BrCNGC25 : SHRLLPEDIRKRVREAERFNWAATRGVNEEFLFENMPEDLLRDIKRHL-F : 564
BrCNGC26 : SHRLLPDDIRKRVREAERFNWAATRGVKEELIFENMPDDIQRDIKRHL-F : 570
BrCNGC27 : SHRRFPKDIRKRVREVERLNWNATRGVNEELLFENMPDDLQRDIRRHL-F : 543
BrCNGC28 : SHRRLPEGIRKRVREAERFNWAATRGVNEELLFENMPDDLQRDIRRHL-F : 581
BrCNGC29 : SHRLLPEDIRKRVREAERFNWAATRGVNEELLFENMPDDLQRDIKRHL-F : 577
            hr lP     r6       W  t g  ee     6p  6 di rh
```

```
                   760         *       780         *       800
BrCNGC1   : TLLNSVPWLNMMDDSWLLEALCDRVKSVFYSANSYIVKEGDPVAEMLIIT : 469
BrCNGC2   : YSLENVPWISFIDDDWLLNEIYDRVKPVFYSQKSYILGEGDPVKEMLIVT : 505
BrCNGC3   : NLLKKVPWFKAMDDR-LLDALCARLNTVLYTENSYIVREGEPVEDMVFIM : 515
BrCNGC4   : KLLKHMPWFEDMDDR-LLDSVCARLKTVIYTEDSYIVDEGEQIENMLFIN : 372
BrCNGC5   : DLLKKVPLFEIMDEQ-LLDAVCDKLRPVLYTENSYAIREGDPVEEMLFVM : 521
BrCNGC6   : ALVMRVPMFEQMDEQ-LLDALCDRLQPVLYTEESYIVREGDPVDEMLFIM : 555
BrCNGC7   : ALLMRVPMFEKMDEQ-LLDALCDRLQPVLYTEESYIVREGDPVDEMLFIM : 550
BrCNGC8   : NLVRRVPLFANMDER-LLDAICERLKPSLYTESTYIVREGDPVNEMLFII : 513
BrCNGC9   : ALVRRVPLFESMDDK-LLDAICMRLKPCLFTESTYLVREGDPVDEMLFII : 570
BrCNGC10  : ALVRRVPLFDNMDER-LLDAICMRLKPCLYTEKSFLVREGDPVNEMLFII : 554
BrCNGC11  : ALVRRVPLFENMDER-LLDAICERLKPCLYTEKSFLVREGDPVNEMLFII : 544
BrCNGC12  : ALVRRVPLFENMEER-LLDAICERLKPCLYTEKSYLVREGDPVNEMLFII : 533
BrCNGC13  : DLVRRVPLFDQMDER-MLDAISERLKPALFTEGTFLVREGDPVNEMLFII : 519
BrCNGC14  : ALVRRVPFFSQMDDQ-LLDAICGCLVSSLSTAGTYIFREGDPVDEMLFVI : 490
BrCNGC15  : ALVRRVPFFAQMDDQ-LIDAICERLVPSLNTKDTYVTREGDPVNEMLFII : 499
BrCNGC16  : DLVRRVPFFSQMDDQ-LLDAICERLVSSLCTEGTYLVREGDLITEMLFII : 526
BrCNGC17  : DLVRRVPLFAQMDDQ-LLDAICERLVSSLSTQGNYIVREGDPVTEMLFII : 527
BrCNGC18  : DLVRRVPLFAQMDDQ-LLDAICERLVSSLSTQGNYIVREGDPVTEMLFII : 523
BrCNGC19  : DLVRQVPLFQHMDDL-VLENICDRVKSLIFTKGETIQKEGDAVQRMLFVV : 538
BrCNGC20  : DLVRQVPLFQHMDDL-VLENICDRVKSLIFTKGETIQKEGDAVQRMLFVV : 541
BrCNGC21  : DLINNVPLFRGMDDL-ILDNICDRAKPRVYSKDEKIIREGDPVQRMIFIM : 566
BrCNGC22  : AFLKKVRIFSKMDEP-ILDAIRERLKHKTYLNGSMVIHSGDLVKKMVIIV : 567
BrCNGC23  : KFLNKVRIFTLMDES-ILNAIRERLKHITYISSSVVFSAGDVIEKIVFIV : 676
BrCNGC24  : IFLKKVRIFSLMDES-ILDSIRERLRQRTYISGSTVLHRWGPVEKIVFVV : 532
BrCNGC25  : KFLKKVRIFSLMDES-ILDSIRERLKQRTYISSSTVLHRRGLVEKMVFIV : 613
BrCNGC26  : KFLKKVWIFSLMDEL-ILDAIRERLKQRRYIRSSTVLRRKGLVEKMVFIV : 619
BrCNGC27  : AFLKKVRIFSEIDES-ILDAMRARLKQRTYLKNNKVLHRGGVVKKMVFIL : 592
BrCNGC28  : IFLKKVRIFSLMDES-ILDAIRERLKQRTYISSSTVLHRGGLVEKMVFIV : 630
BrCNGC29  : KFLKKVRIFSLMDES-ILDAIRERLKQRTYIRSSTVLHRKGLVEKMVFIV : 626
              6  6  f  6d   66  6                  g  6   66f6
```

```
                    *       820         *       840         *
BrCNGC1  : KGSLKSMIGSSDITGYYD-SSYLQAGDICG-DLLFWV-LDPHSSS----- : 511
BrCNGC2  : YGELESMTESFETSSYSDIQIRLMKGDVW-EDLLFWA-LDPHTSP----- : 548
BrCNGC3  : RGKLISTTTYGGQTGFFN-IAHLEAGDFCG-DLLTWA-LDPNTS------ : 556
BrCNGC4  : RG---TVIVTEKKTTFF-----RSVGEFCGEELLSWALLDPHSS------ : 408
BrCNGC5  : RGKLMSATTNGGRTGFFN-AVYLKASDFCGEDLLTWA-LDPQSSS----- : 564
BrCNGC6  : RGKLLTMTTNGGRTGFFN-SEHLGAGDFCGEELLTWA-LDPHTST----- : 598
BrCNGC7  : RGKLLTMTTNGGRTGFFN-SEHLGAGDFCGEELLTWA-LDPHTSS----- : 593
BrCNGC8  : RGRLESVTTDGGRSGFFN-RGLLKEGDFCGEELLTWA-LDPKAGS----- : 556
BrCNGC9  : RGRLESVTTDGGRSGFFN-YSLLKEGEFCGEELLTWA-LDPKSGV----- : 613
BrCNGC10 : RGRLECVTTDGGRSGFYK-RSLLKEGDFCGDELLTWA-LDPKSGS----- : 597
BrCNGC11 : RGRLESVTTDGGRSGFYN-RSLLKEGDFCGDELLTWA-LDPKSGS----- : 587
BrCNGC12 : RGRLESVTTDGGRSGFFN-RSLLKEGDFCGEELLTWA-LDPKSGS----- : 576
BrCNGC13 : RGHLDSYTTNGGRTGFFN-SCLIGPGDFCGEELLTWA-LDPRPVV----- : 562
BrCNGC14 : RGQIESSTTNGGRSGFFN-STTLRPGDFCGEELLTWA-LMPNSTL----- : 533
BrCNGC15 : RGQMESSTTDGGRSGFFN-SITLRPGDFCGEELLTWA-LMPNINQ----- : 542
BrCNGC16 : RGRLESSTTNGGRTGFFN-SIILRPGDFCGEELLSWA-LLPKSTL----- : 569
BrCNGC17 : RGKLESSTTNGGRTGFFN-SITLKPGEFCGEELLAWA-LLPKSKV----- : 570
BrCNGC18 : RGKLDSSTTNGGRTGFFN-SITLKPGEFCGEELLAWA-LLPKSKV----- : 566
BrCNGC19 : RGHLQS--SQLLRDGVKS-CCMLGPGNFSGDELLSWC-LRRPFVE----- : 579
BrCNGC20 : RGHLQS--SQLLRDGVRS-CCMLGPGNFSGDELLSWC-LRRPFVE----- : 582
BrCNGC21 : RGRVKR--NQSLSKGVVA-TSTLEPGGYLGDELLSWC-LRRPFID----- : 607
BrCNGC22 : RGQMESTREDGSCTR-------LSKRDVYGEELLTW--YERSSLNPDGTR : 608
BrCNGC23 : RGEMESIGKDGSVIS-------LSEGDVFGEELLSWC-LERAASNTDGTR : 718
BrCNGC24 : RGEMESTGEDGSVLP-------LSEGDVCGEELLTWC-LERSAVNLDG-- : 572
BrCNGC25 : RGEMESIGQDGSVLL-------LSEGDVCGEELLTWC-LERSSVNPDGRS : 655
BrCNGC26 : RGERESIGEDGSVLP-------LSEGDVCGEELLTWC-LERSSVDPDGTR : 661
BrCNGC27 : RGKMESIGEDGYRTL-------LSEGDVCGEELLTWC-QERSSVNPDGTI : 634
BrCNGC28 : RGEMESIGEDGSVLP-------LSEGDVCGEELLTWC-LERSSVNPDGTR : 672
BrCNGC29 : RGEMESIGEDGSVLP-------LSEGDVCGEELLTWC-LERSSVNPDGTR : 668
            G        g              g  cg eLL W  l
```

```
                  860         *       880         *       900
BrCNGC1  : -SLPT----SDRSVLTLTDVEGFILLHDDLKFVASHFNR-FHSSRLRHM- : 554
BrCNGC2  : -SLPI----SNGTVTTLTYVEGLTLSADDLK------------------- : 574
BrCNGC3  : -HLPI----STRTVQAETEVEGFVLSAEDLKFFSTQYRR-LHSKQLRHT- : 599
BrCNGC4  : -CVPI----SSMIIKARTEVEGFVLSADDLKFVAAQYCR-LHRKKARHIS : 452
BrCNGC5  : -HFPI----SPRTVQALTEVEAFALAAEDLKLVASQFRR-LHSKQLQHT- : 607
BrCNGC6  : -NLPI----STRTVQALVEVEAFALKADNLKFVASQFRR-LHSKQLRHT- : 641
BrCNGC7  : -NLPI----STRTVRALVEVEAFALKADDLKFVASQFRR-LHSKQLRHT- : 636
BrCNGC8  : -NLPS----STRTVKALTEVEAFALEAEELKFVASQFRR-LHSRQVQQT- : 599
BrCNGC9  : -NLPS----STRTVKALTEVEAFALASEELKFVASQFRR-LHSRQVQHT- : 656
BrCNGC10 : -NLPS----STRTVKALTEVEAFALIADELKFVASQFRR-LHSRQVQHT- : 640
BrCNGC11 : -NLPS----STRTVKALTEVEAFALIADELKFVASQFRR-LHSRQVQHT- : 630
BrCNGC12 : -NLPS----STRTAKALTEVEAFALIADELKFVASQFRR-LHSRQVQHT- : 619
BrCNGC13 : -ILPS----STRTVKAIYEVEAFALRADDLKFVATQFRR-LHSKQLKHK- : 605
BrCNGC14 : -NFPS----STRSVRALSEVEAFALSAEDLKFVAHQFKR-LQSKKLQHA- : 576
BrCNGC15 : -NLPL----STRTVRTLSEVEAFALRAEDLKFVANQFRR-LHSKKLQHA- : 585
BrCNGC16 : -NLPS----STRTVRALVEVEAFALRAEDLKFVANQFRR-LHSKKLQHT- : 612
BrCNGC17 : -NLPS----STRTVRALEEVEAFALQAEDLKFVANQFRR-LHSKKLQHT- : 613
BrCNGC18 : -NLPS----STRTVRALEEVEAFALQAEDLKFVANQFRR-LHSKKLQHT- : 609
BrCNGC19 : -RLPP----SSSTLVTLETTEAFGLDAQDVKYVTQHFRYTFVNEKVKRS- : 623
BrCNGC20 : -RLPP----STSTLVTLETTEAFGLDAEDVKYVTQHFRYTFVNEKVKRS- : 626
BrCNGC21 : -RLPP----SSATFVCLDNIEAFSLGSEDLRYITDHFRYKFANERLKRT- : 651
BrCNGC22 : IRMPSKGLLSNRDVKCVTNVEAFSLSVADLDDVMSLFPN----------- : 647
BrCNGC23 : IWIKRKGLLSYRSVRCVTNVDAFSLSVADLDASRDC-------------- : 754
BrCNGC24 : -RMLSKGLLSSRNVKCVTNVEAFSLSAADLEDVTSLFSRFLRSHRVQGA- : 620
BrCNGC25 : IKLPLKGLVSNRSVRCVTNVEAFSLSVADLEDVTSLFSRFLRSHRVLGA- : 704
BrCNGC26 : IKMPTKGLVSNRSVRCVTNVEAFSLSVADLEDVTSLFSRFLRSNRVQGA- : 710
BrCNGC27 : IRMPSKGLLSSRDVWCVTNVEAFSLSVADLEDITSLFPN----------- : 673
BrCNGC28 : IRIPSKGLLSYRNVKCVTNVEAFSLSVADLEDVTSLFSRFLRNPRVQGA- : 721
BrCNGC29 : IKMPTKGLVSNRNVRCVTNVEAFSLSVADLEDVTSLFSRFLRSHRVQGA- : 717
              p     S r        eaf L    6
```

```
                      *        920         *        940          *
BrCNGC1  : -----FYSAHWRLWAACFIQAAWREHYKRKLSRILHAKRDYN-------- : 591
BrCNGC2  : -----CHSKSWRSWAAFYIQAAWKAHCRRKASKILPAIKDEQ-------- : 611
BrCNGC3  : -----CYSVQWQTWAACFIQAAWKRYCRRKLSRVLREEEERL-------- : 636
BrCNGC4  : RQEFQLHSRKWRNWAACFIQAAWTEYCRRKLSKDLGEEEAKL-------- : 494
BrCNGC5  : ---FRFYSVQWRTWGASFIQAAWRRHCRRRLARSLTEEEDRF-------- : 646
BrCNGC6  : ---FRFYSQQWRTWAACFIQAAWRRHVKKRLEESLREEENRL-------- : 680
BrCNGC7  : ---FRFYSQQWRTWAACFIQAAWRRHVKKKMEESLKEEENRL-------- : 675
BrCNGC8  : ---FRFYSQQWRTWASSFIQAAWRRHSRRKNAELRRIEEEEDEMGYED-- : 644
BrCNGC9  : ---FRFYSHQWRTWAACFIQAAWRRYCKRKKMEEAEAEAVPM-------- : 695
BrCNGC10 : ---FRFYSQQWRTWAACFIQAAWRRYTKRKKLEQLRKEEEEE-------- : 679
BrCNGC11 : ---FRFYSQQWRTWAACFIQAAWRRYTKRKKLEELRKEEEME-------- : 669
BrCNGC12 : ---FRFYSQQWRTWAAIFIQAAWRRYVKKKKLEQLKKEEEEE-------- : 658
BrCNGC13 : ---FRFYSHQWRTWAACFIQAAWRRHKKRKYATELRVKEEFQ-------- : 644
BrCNGC14 : ---FRYYSHQWRAWGACFVQSAWRRYKRRKLAKELSLHESSGYYYRDETG : 623
BrCNGC15 : ---FRYYSHQWRAWGTGFIQAAWRRYMKRKLAMELARQEEGDDYYYDD-- : 630
BrCNGC16 : ---FRFYSPHGRTWAACFIQAAWRRYKRRAMENHLTAVESKQ-------- : 651
BrCNGC17 : ---FRYYSHQWRTWAACFVQVAWRRYKRRMLAKSLSLAESYSSYEEEE-- : 658
BrCNGC18 : ---FRYYSHQWRTWAACFVQVAWRRYKRRMVAKSLSLAESFSSYEEEE-- : 654
BrCNGC19 : ---ARYYSPGWRTWAAVAVQLAWRRYKHRLTLTSLSFIRPRR-------- : 662
BrCNGC20 : ---ARYYSPGWRTWAAVAVQLAWRRYKHRLTLTSLSFIRPRR-------- : 665
BrCNGC21 : ---ARYYSSNWRTWAAVNIQMSWRRYRKRTCGGVGGSMSPVS-------- : 690
BrCNGC22 : -------------------------------------------------- :   -
BrCNGC23 : -------------------------------------------------- :   -
BrCNGC24 : ---IRYESPYCRLRAATQIQVAWR-YRKRRLQKLSTAQKK---------- : 656
BrCNGC25 : ---IRYESPYWRLRAATQIQVAWR-YRRRRLHRLYTAQST---------- : 740
BrCNGC26 : ---IRYESPYWRLRAAMQIQVAWR-YRRKRLERKKQ-------------- : 742
BrCNGC27 : -------------------------------------------------- :   -
BrCNGC28 : ---IRYESPYWRLRAARQIQVAWR-YRRRRLQRLYTAQS----------- : 756
BrCNGC29 : ---IRYESPYWRLRAAMQIQVAWR-YRKKRLERLILQDNRVM-------- : 755
                  s  wr  aa   q aw
```

```
                  960         *      980          *      1000
BrCNGC1  : ------------------------HIPQGTQLNIG-----AALYVSRFVS : 612
BrCNGC2  : ------------------------QILQDTQRNLG-----ATLYASRFVS : 632
BrCNGC3  : -----------------QNTLQTTDDSGGNKLNLG-----AAIYASRFAS : 664
BrCNGC4  : ------------------HSTIQKDDSAGNTHNLG-----GTAFASRLAS : 521
BrCNGC5  : -----------------RIAVAKRERRAASSPSLV-----ATLYASRFAS : 674
BrCNGC6  : -------------------QDALAKQACGSSPSFG-----ATMYASRFAA : 706
BrCNGC7  : -------------------QDALAKEACGSSPSLG-----ATMYASRFAA : 701
BrCNGC8  : --------EYDDDDAEEEDERTPVFTRTESSSRLR-----STIFASRFAA : 681
BrCNGC9  : -------------------------STTGSSSSMG-----AAFLVTKFAA : 715
BrCNGC10 : -----------------EEESAARLIAGGSPYSIR-----ATFLASKFAA : 707
BrCNGC11 : ----------------EESSTARLIAGGSSPFSIR-----ATFLASRFAA : 698
BrCNGC12 : --------------------------GEGHVASIR-----ATFLASKFAA : 677
BrCNGC13 : ----------------------------------------CMFETASMVR : 654
BrCNGC14 : YNEEGDEENYYGSDDDDFEGERLSVDNTNNSQNLG-----ATMLASKFAA : 668
BrCNGC15 : ------DDDDQYGGEDMPESSNNVDDNSSNSQNLS-----ATILASKFAA : 669
BrCNGC16 : -SDEDDEEEEVVVRKVVEEEEEGVGSSPKTKMNIG-----VMVLASRFAA : 695
BrCNGC17 : -----ALAAAAAEEIMSQQEERQSSTPSRHHTSIGKPHFAATVLASRFAK : 703
BrCNGC18 : ------AVAVAAEEIMSQQGERQSSNPSRHSTSIGKPHFAATILASRFAK : 698
BrCNGC19 : ----------------------------------------PLSRCASLGE : 672
BrCNGC20 : ----------------------------------------PLSRCASLGE : 675
BrCNGC21 : --------------------------------------------EHSVEG : 696
BrCNGC22 : -------------------------------------------------- :   -
BrCNGC23 : -------------------------------------------------- :   -
BrCNGC24 : -------------------------------------------------- :   -
BrCNGC25 : -------------------------------------------------- :   -
BrCNGC26 : -------------------------------------------------- :   -
BrCNGC27 : -------------------------------------------------- :   -
BrCNGC28 : -------------------------------------------------- :   -
BrCNGC29 : ----------------------------------------KEMSDVRIGY : 765
```

```
                    *      1020         *      1040         *
BrCNGC1  : KALRNRQKNAANCS---------ISPHMLPPIPHKPADPEFSKN------ : 647
BrCNGC2  : KALRNRHVDSAECS------------SFPEMLPDKPADPEFSKKEA---- : 666
BrCNGC3  : HALRNVRANAAARS--------SMLPHMLSLLPQKPADPEFPMDET---- : 702
BrCNGC4  : TVLRNLRVNASRKD-----------RVRQISLPEKPVDPKFPMDEI---- : 556
BrCNGC5  : NALRNLRQHNNNTL---------------PLLPPKPSEPDFSVDDD---- : 705
BrCNGC6  : NILRTIRRSGSVRK------PRMLERMPPMLLLQKPAEPDFNSDDYMRIV : 750
BrCNGC7  : NILRTIRRSGSVRK----------PRMPERMLLQKPAEPDFNSDDYCI-- : 739
BrCNGC8  : NALKGHRLRSTESS-------------KRLLNLQKPPEPDFDAE------ : 712
BrCNGC9  : SALRTIHRNRNTRI-------------RELVKLQKPPEPDFTAEDAD--- : 749
BrCNGC10 : NALRSVHKNRIRKS------NLAPPSTKELVKFQKPPEPDFSADC----- : 746
BrCNGC11 : NALRGVRKNRTAKL------LALSQPTKELLKVQKPPEPDFSADC----- : 737
BrCNGC12 : NALRKVHQNRIAAK-----------STKELVIFQKPSEPDFSADDP---- : 712
BrCNGC13 : LNSGKFTRSGSDSG--------------MVSSIQKPVEPDFSSE------ : 684
BrCNGC14 : NTRRGT--NQKASSSTSAGKKDGSSNSLKMPQLFKPDEPDFSMDKEDV-- : 714
BrCNGC15 : NTKRGVLGNQRGSS-----RIDPDDPTLKMPKMFKPEDPGFF-------- : 706
BrCNGC16 : NTRRGVAAQRVKDV--------------EMPRFKKPEEPDFSAEPDD--- : 728
BrCNGC17 : NTRRASRKMKDVDV----------------PMLPKPEEPDFSVDAD---- : 733
BrCNGC18 : NTRKTAHKLKDVEV----------------PMLPKPDEPDFSVDGD---- : 728
BrCNGC19 : DKLRLYTAILT---------------------SPKPNPDDFDDY------ : 695
BrCNGC20 : DKLRLYTAILT---------------------SPKPNPDDFDDY------ : 698
BrCNGC21 : NSERRLLQYAA------------------MFMSIRPHDHLE--------- : 719
BrCNGC22 : --------------------------------LEISKES----------- : 654
BrCNGC23 : -----------------------------------SRDS----------- : 758
BrCNGC24 : -----------------------------------QYSSELIQKDMAKT- : 670
BrCNGC25 : -----------------------------------SRR------------ : 743
BrCNGC26 : -----------------------------------NGDIDE--------- : 748
BrCNGC27 : --------------------------------LEIPKEP----------- : 680
BrCNGC28 : -----------------------------------SYSL----------- : 760
BrCNGC29 : RLSWSGVVEGS------------------------GKQLDTRGSS----- : 786
```

```
BrCNGC1   : --------- :    -
BrCNGC2   : --------- :    -
BrCNGC3   : --------- :    -
BrCNGC4   : --------- :    -
BrCNGC5   : --------- :    -
BrCNGC6   : PIYKNRSL  :  758
BrCNGC7   : --------- :    -
BrCNGC8   : --------- :    -
BrCNGC9   : --------- :    -
BrCNGC10  : --------- :    -
BrCNGC11  : --------- :    -
BrCNGC12  : --------- :    -
BrCNGC13  : --------- :    -
BrCNGC14  : --------- :    -
BrCNGC15  : --------- :    -
BrCNGC16  : --------- :    -
BrCNGC17  : --------- :    -
BrCNGC18  : --------- :    -
BrCNGC19  : --------- :    -
BrCNGC20  : --------- :    -
BrCNGC21  : --------- :    -
BrCNGC22  : --------- :    -
BrCNGC23  : --------- :    -
BrCNGC24  : --------- :    -
BrCNGC25  : --------- :    -
BrCNGC26  : --------- :    -
BrCNGC27  : --------- :    -
BrCNGC28  : --------- :    -
BrCNGC29  : --------- :    -
```

Figure S2. Multiple sequence alignment between BrCNGCs and AtCNGCs using full length amino acid sequences.

```
                       *        20         *        40         *        60         *        80
BrCNGC1  : -------------------------------------------------------------------------------- :  -
BrCNGC2  : --------------------------------------------------------------------------MTIFSV :  6
BrCNGC3  : -----------------------------------MEMMNLKRNTFVKFTE---------NEDSWN----RPSVTSVIKK : 32
BrCNGC4  : -------------------------------------------------------------------------------- :  -
BrCNGC5  : --------------------------------------MGFGRDSRVRFKE------PSSTEFGYGR-RARPSLNAVLD- : 34
BrCNGC6  : MKFRLTSLTLSSCGSRVLHSSHKEGYTTISCASTNSPTMNFRPDKSVRFHDWKSDKAS-DVEYS---EVPDGLYRRAISS : 76
BrCNGC7  : --------------------------------------MNFRQEKFVRFQDWKSDKTS-DVEYSGRNEPPNGIFRRTITS : 41
BrCNGC8  : ---------------------------------------------------------------MMTKRNCFG-FPVKNRG : 16
BrCNGC9  : ----------MTFASLPYHFCRASDRGLSRSSVIELAIMAGKPQTFVSVDDLDFKLPSS--SSLTRQHNYSSSISGPLHP : 68
BrCNGC10 : ------------------------MFD-CGTNGVKSQVISGHREKFIRLESMDSRYSQS--SDNTGLNKCTLNIQA-PKR : 52
BrCNGC11 : -------------------------------MESKSQVISGHREKFIRLDSMDPR------SPEAGLNRCTINIQR-PKR : 42
BrCNGC12 : ---------------------------------------------------MDSRYSQ---GAEAGLNKCTLNLQGPSRA : 26
BrCNGC13 : --------------------------------------MGYGNSRSVRFEEDSEVTKPQAVHEETAVKLKFKINGAQISP : 42
BrCNGC14 : -------------------------------------------------------------------MNKIRSLRFLLPE : 13
BrCNGC15 : -----------------------------------------------------------------MSNLHLHTSARFNFP : 16
BrCNGC16 : --------------------------------------MELRKDKILMLYSDKKEPKEAIWAVN-DPMSKSYKLSLPSAL : 41
BrCNGC17 : --------------------------------------MEFKRDNTVRFYGEEKQTLEATEKRQPLPMFKPSTTQFLKPE : 42
BrCNGC18 : --------------------------------------MEFKRDNTVRFYGDEKQTLEVTEKRLPLPMFKSSAAPFQKQE : 42
BrCNGC19 : ----------------------MATEQEFTR------ASRVSGASSSVGYYS-------DEDYK-DEEEEEEEEEEEMEE : 44
BrCNGC20 : ----------------------MATEQEFTR------ASRVSRASSSIGYYS-------DEDYTTDEEENEEEEMEELEE : 45
BrCNGC21 : ----------------------MPSHTNFLFRWIGLFSQKLRRETTEISEN-----NGGESSSS-SDDTPVLSSGECYAC : 52
BrCNGC22 : --------MASSNGYD--DVPMLPVSCTSSSS-RTRPFTSRSRSVSLSNTSSTIDVFENSSTVVLGYTDPLGTQRQPQLV : 69
BrCNGC23 : --------MAPPNEND--DALKLPVLDTSSSS-RTRPFTFRSRNVSLPNTSSTSEGL-DSSTVVLRYTDPHRTQRPPPSV : 68
BrCNGC24 : ---------------------MLPISDASSSSSQTRVFTSRTRSVPLSNPTEETG---NSKAATLGYAGSLPSQR-PPLF : 55
BrCNGC25 : --------MTSPNEND------QVSIPEATSRAHTRAFNFKNRSVSLSNSTYYIDGC-DNSKVALGYTVPIRTQRRPP-- : 63
BrCNGC26 : --------MASPNENESDEFPMLRQFPEARS----RALHSRNRSISFSDSTYSSNRV-ENS------SGPRRTQSRPSPS : 61
BrCNGC27 : --------MASPMEND--DVPMLPASDTSSSS-RTMPFTSRSRSTSLANNSSTIDVF-NSSTVVLGYTDPLGTQRRPPLV : 68
BrCNGC28 : --------MASPKEND--DVPMLPISDTS----RTRPFTSRSRSVSLSNTCSTIDGF-DSSTVVLGYTGPLRAQRRPPLV : 65
BrCNGC29 : --------MASPNEK--DEFPILLPVPEARSRANTRAFNSRNRSVSFSNSTYSTNRV-DNSSVVLGYTGPLRTQRRLPPS : 69
AtCNGC1  : --------------------------------------MNFRQEKFVRFQDWKSDKTSSDVEYSGKNEIQTGIFQRTISS : 42
AtCNGC2  : ----------------------MPSHPNFIFRWIGLFSDKFRRQTTGIDENSNLQINGGDSSSSGSDETPVLSSVECYAC : 58
AtCNGC3  : -------------------------------------MMNPQRNKFVRFNG---------NDDEFSTKTTRPSVSSVMK- : 33
AtCNGC4  : ----------------------MATEQEFTR------ASRFSRDSSSVGYYS-------EEDNT-EEEDEEEEEMEEIEE : 44
AtCNGC5  : --------------------------------------MAGKRENFVRVDDLDSRLPSS--S-VAFQQNYASNFSGQLHP : 39
AtCNGC6  : ------------------------MFDTCGPKGVKSQVISGQRENFVRLDSMDSRYSQ---SSETGLNKCTLNIQGGPKR : 53
AtCNGC7  : ---------------------------------------------------------------MMMQRNCFG-FNLKNRG : 16
AtCNGC8  : --------------------------------MYKSQYISGHREKFVRLDDTDSRVSMSSNATGMKKRSCFGLFNVTSRG : 48
AtCNGC9  : -------------------------MLDCGKKAVKSQVISGRLEKFVRLDSMDSRYSQ---TSDTGLNRCTLNLQGPTRG : 52
AtCNGC10 : -------------------------------------------MILFRFKDEG---KPLSSEYGYGR-KARPSLDRVFK- : 32
AtCNGC11 : -------------------------------------------------------------------------------- :  -
AtCNGC12 : -------------------------------------------------------------------------------- :  -
AtCNGC13 : --------------------------------------MAFGRNNRVRFRDW----ISEGTEYGYGRNKARPSLNTVLK- : 37
AtCNGC14 : --------------------------------------MEFKRDNTVRFYGDEKQTIEVGEKR--VPLFKSTTAPFMKQE : 40
AtCNGC15 : --------------------------------------MGYGNSRSVRFQEDQEVVH----GGESGVKLKFKINGTQIN- : 37
AtCNGC16 : -----------------------------------------------------------------MSNLHLYTSARFNFP : 16
AtCNGC17 : --------------------------------------MELRKDKLLMFYSEGKESKEAKWAVN-DPMSKSYKLSLPSAL : 41
AtCNGC18 : -------------------------------------------------------------------MNKIRSLRCLLPE : 13
AtCNGC19 : -------------------------------MAHTRTFTSRNRSVSLSNPSFSIDGF-DNSTVTLGYTGPLRTQRIRPPL : 48
AtCNGC20 : --------MASHNEND--DIPMLPISDPSSRT-RARAFTSRSRSVSLSNPTSSIEGF-DTSTVVLGYTGPLRTQRRPPLV : 68

                       *       100         *       120         *       140         *       160
BrCNGC   : -------------------------------------------------------------------------------- :  -
BrCNGC2  : -------------------------------------------------------------------------------- :  -
BrCNGC3  : -------------------------------------------------------------------------------- :  -
BrCNGC4  : -------------------------------------------------------------------------------- :  -
BrCNGC5  : -------------------------------------------------------------------------------- :  -
BrCNGC6  : -------------------------------------------------------------------------------- :  -
BrCNGC7  : -------------------------------------------------------------------------------- :  -
```

```
BrCNGC8  : -------------------------------------------------------------------------------- :   -
BrCNGC9  : -------------------------------------------------------------------------------- :   -
BrCNGC10 : -------------------------------------------------------------------------------- :   -
BrCNGC11 : -------------------------------------------------------------------------------- :   -
BrCNGC12 : N------------------------------------------------------------------------------- :  27
BrCNGC13 : -------------------------------------------------------------------------------- :   -
BrCNGC14 : -------------------------------------------------------------------------------- :   -
BrCNGC15 : -------------------------------------------------------------------------------- :   -
BrCNGC16 : -------------------------------------------------------------------------------- :   -
BrCNGC17 : -------------------------------------------------------------------------------- :   -
BrCNGC18 : -------------------------------------------------------------------------------- :   -
BrCNGC19 : T------------------------------------------------------------------------------- :  45
BrCNGC20 : A------------------------------------------------------------------------------- :  46
BrCNGC21 : T------------------------------------------------------------------------------- :  53
BrCNGC22 : Q-MGDPISSTRNLEL----------------------------------------------------------------- :  83
BrCNGC23 : Q-MNGPLFSTSSPEPLILLPPPSTGGSSDP-------VGVSSSQPERYPSFAALEHDNSDDNSVLNPHLLRSEKFGVCND : 140
BrCNGC24 : P-MTGPLSSSTRRS---------------------SGYFGDLEEVNSSDNDELLKHAH----------RLRSGKLGMCND : 103
BrCNGC25 : ----GPLYSTPRPE-SHFPPS-IEPPDS----------SSTVDVRSEDE--SVLENAN----------ILKSGQLGMCNE : 115
BrCNGC26 : VHMSGPLYDTRRPDQSFFPPSPVQPPASSL-------SSSTVDIPSEEVVEALLKNAN----------LLKSGQLGMCND : 124
BrCNGC27 : Q-MSYPLSSTRSPEPRFALPPPSTGASYDS-------VGASSSQP----------------------------------- : 105
BrCNGC28 : Q-MSGPLSSTRTPEPLFLLPPPSDSVGISSSQPERYPSFATLEHKKS-DDEFVLKHAN----------LLRSGQLGMCND : 133
BrCNGC29 : VQMSGPLYSTRRPDQSFFPPSPVQPPDS----------SSTVDVPSEED-EVVLKNAN----------LLKSGQLGMCND : 128
AtCNGC1  : -------------------------------------------------------------------------------- :   -
AtCNGC2  : T------------------------------------------------------------------------------- :  59
AtCNGC3  : -------------------------------------------------------------------------------- :   -
AtCNGC4  : E------------------------------------------------------------------------------- :  45
AtCNGC5  : -------------------------------------------------------------------------------- :   -
AtCNGC6  : -------------------------------------------------------------------------------- :   -
AtCNGC7  : -------------------------------------------------------------------------------- :   -
AtCNGC8  : -------------------------------------------------------------------------------- :   -
AtCNGC9  : -------------------------------------------------------------------------------- :   -
AtCNGC10 : -------------------------------------------------------------------------------- :   -
AtCNGC11 : -------------------------------------------------------------------------------- :   -
AtCNGC12 : -------------------------------------------------------------------------------- :   -
AtCNGC13 : -------------------------------------------------------------------------------- :   -
AtCNGC14 : -------------------------------------------------------------------------------- :   -
AtCNGC15 : -------------------------------------------------------------------------------- :   -
AtCNGC16 : -------------------------------------------------------------------------------- :   -
AtCNGC17 : -------------------------------------------------------------------------------- :   -
AtCNGC18 : -------------------------------------------------------------------------------- :   -
AtCNGC19 : VQMSGPIHSTRRTE-PLFSPSPQESPDS----------SSTVDVPPEDD--FVFKNAN----------LLRSGQLGMCND : 105
AtCNGC20 : Q-MSGPLTSTRKHEPLFLPHPSSDSVGVSS-QPERYPSFAALEHKNSSEDEFVLKHAN----------LLRSGQLGMCND : 136


                    *       180         *       200         *       220         *       240
BrCNGC   : ------------------------------------MERASTIQSVHEN--IKSVRGQLKKVYKTLKTLENWR------- :  35
BrCNGC2  : ----------------QSTLFTRASVA---LLSSNGLKRFSFASSFSSAALYSPPLPKTKKRRFPIVSAVDIGGVTVARN :  67
BrCNGC3  : ----------------TVRRSFEKGSE---KIRTFKQQPLTFHSQKKNEN--KKKIIR--VMNPNDSYLQNWN------- :  82
BrCNGC4  : -----------------------------------------------------------------MKTLNTRK------- :   8
BrCNGC5  : ----------------NVRRGFEKGSD---KIRTFKR-PLSFNSHKNEEKRNATGTQKKNIINPQGSFLQNWN------- :  87
BrCNGC6  : ----------------ISDKFHR-------SSLRIKMLR---TSYSFKETVSKGIVSTHEILDPQGTFLQKWN------- : 123
BrCNGC7  : ----------------ISDKFHR-------SSARIKTFRRTYKSYSFKEAVSKGIDSSHKILDPQGPFLQRWN------- :  91
BrCNGC8  : -----------SEKKR-ASKSFREGVK-IGSEGLFSIGKS----VTRAVFPEDLRISEKKIFDPQDKTLLIWN------- :  72
BrCNGC9  : ---------IQGS--HNTSGSFKKRFQ-KGSKGLKSIGRSLGFGVYRAVFPEDLKVSEKKIFDPQDKTLLFCN------- : 129
BrCNGC10 : --------FAQGS--KTSSGSFKKGFR-KGSEGLWSIGRSIGLGVSRAVFPEDLKVSEKKIFDPQDKFLLLCN------- : 114
BrCNGC11 : --------FTQAN--KTSSGSFKKGFR-KGSEGLWSIGRSIGLGVSRAVFPEDLEVSEKKIFDPQDKFLLLCN------- : 104
BrCNGC12 : -------GAGQGNNNNASSGSFKKGFR-RGSKGLWSLGRSIGLGVSRAVFPEDLKVSERKIFDPQDKFLLLCN------- :  92
BrCNGC13 : -----------------RKNVKKMTR-----------GKSFKDKVLSRVFTEDLGRVKNKILDPRGQTIRKWN------- :  87
BrCNGC14 : -----------------TITSA-----------------ASNRGSVAVRYGSQVLPWRHQILDPDSNIVTYWN------- :  52
BrCNGC15 : -----------------TAFSRR---------------HHNNNDLQNQRGRSVFSELGDTTLDPSGDLITRWN------- :  57
BrCNGC16 : -----------------KPPDNNLLS-----------GNRISRYTDNNKSKSSKPSWYKTILDPGSEIVLKWN------- :  86
BrCNGC17 : -----------------LVIPKKTNKT---------RLFKLPRFGGLKVFPENFEIERDKILDPGGDVVLQWN------- :  89
BrCNGC18 : -----------------LGTSKKS-------------IFKIPRFGRFKVFPENFEIERDKILDPGGDVVLQWN------- :  85
BrCNGC19 : -------EK---------DEEEEEPRVRVTCGGRR----NGSPGSYNKWMM------LGRILDPRSKLVQEWN------- :  92
BrCNGC20 : -------E-----------EEEEETHVGGTCGIRRR---NGSSSSYNKWMV------LGRILDPRSKLVQEWN------- :  92
```

```
BrCNGC21 : -------QVGVPAFHSTSCDQANAPEWRASAGSSLVPIQEGSAPSPVRARIRRLKGPFGEVLDPRSKRVQRWN------- : 119
BrCNGC22 : ----------------------------------VHNARDDDAKGWARRFVTSVDKYLPKIMEPDSKEVGGWT------- : 122
BrCNGC23 : PYCTTCPSYYNRKADQVPTSRVPAIFYS-----MFHSALYEDAKARARRFATSVNRHLPGIMNPHSIFIQSWT------- : 208
BrCNGC24 : PYCTTCPSNYNPKASRLPNP-TVSA-------STFHNALYDDARSWARRFASSVNRCLPGIMNPHSKFVQIWT------- : 168
BrCNGC25 : PYCTTCPSYYSHQSANFHTS-KVSDSR-------FHTVLYDDARGWAKRFASCVRRCVPGIMNPHSKFVQVWT------- : 180
BrCNGC26 : PYCTTCPSYYNLQAAQFHTYGVVSDSRTQVNVTHNRQALHDYDRGWAKLFASYVRRCVPGIINPHSKFVQMWT------- : 197
BrCNGC27 : ---NERNHAYSRKAAQSRTPRVFATSDF-----TLHNALDDDAKGWA--------KYFSGIIYPESNYVQLWT------- : 162
BrCNGC28 : PYCTTCPSYYNRKAAQIPSS-RVSAFFD----SKFHNALYDDAKGWARRFATTANRYLPGIMNPHSKFVQSWT------- : 201
BrCNGC29 : PYCTTCPSYYNRQAAQFHTYRVVSDSR-------FRTALYDDARGWAKRFASSVRKMVPGIMNPHSKFVQMWT------- : 194
AtCNGC1  : ----------------ISDKFYRSFES---SSARIKLFKRSYKSYSFKEAVSKGIGSTHKILDPQGPFLQRWN------- :  96
AtCNGC2  : -------QVGVPAFHSTSCDQAHAPEWRASAGSSLVPIQEGSVPNPARTRFRRLKGPFGEVLDPRSKRVQRWN------- : 125
AtCNGC3  : ----------------TVRRSFEKGSE---KIRTFKR-PLSVHSNKNKENNKKKKILR--VMNPNDSYLQSWN------- :  84
AtCNGC4  : -------EE---------EEEEEDPRIGLTCGGRR----NGSS-NNNKWMM------LGRILDPRSKWVREWN------- :  91
AtCNGC5  : ---------IHAS--NETSRSFKKGIQ-KGSKGLKSIGRSLGFGVYRAVFPEDLKVSEKKIFDPQDKFLLYCN------- : 100
AtCNGC6  : --------FAQGS--KASSGSFKKGFR-KGSEGLWSIGRSIGLGVSRAVFPEDLEVSEKKIFDPQDKFLLLCN------- : 115
AtCNGC7  : -----------GEKKK-ASKSFREGVKKIRSEGLITIGKS----VTRAVFPEDLRITEKKIFDPQDKTLLVWN------- :  73
AtCNGC8  : -----------GGKTKNTSKSFREGVK-IGSEGLKTIGKSFTSGVTRAVFPEDLRVSEKKIFDPQDKTLLLWN------- : 109
AtCNGC9  : -------GGAQGN--NVSSGSFKKGFR-KGSKGLWSIGRSIGLGVSRAVFPEDLKVSEKKIFDPQDKFLLLCN------- : 115
AtCNGC10 : ----------------NVKWG-------------FKK-PLSFPSHKDPDHKETSSVTRKNIINPQDSFLQNWN------- :  75
AtCNGC11 : --------------------------------MNLQRRKFVRLDSTGVDGKLKSVRGRLKKVYGKMKTLENWR------- :  41
AtCNGC12 : --------------------------------MNHRRSKFARIDSMGVDGKLKSVRGRLKKVYGKMKTLENWR------- :  41
AtCNGC13 : ----------------NVRRG-------------LKK-PLSFGSHNKKRDSNSSTTTQKNIINPQGSFLQNWN------- :  80
AtCNGC14 : -----------------VLPKKSK------------TRLKIPRFGRFKVFPENFEIERDKILDPGGDAVLQWN------- :  84
AtCNGC15 : -------------------NVKMMSK-----------GKFLKAKVLSRVFSEDLERVKTKILDPRGQTIRRWN------- :  80
AtCNGC16 : -----------------TTFSLR---------------HHHN-DPNNQRRRSIFSKLRDKTLDPGGDLITRWN------- :  56
AtCNGC17 : -----------------RP--DNLLP-----------GNRL-RYTDASKSKSSKVSWYKTILDPGSEIVLKWN------- :  83
AtCNGC18 : -----------------TITSAST--------------AASNRGSDGSQFS---VLWRHQILDPDSNIVTYWN------- :  52
AtCNGC19 : PYCTTCPSYYNRQAAQLHTS-RVSASR-------FRTVLYGDARGWAKRFASSVRRCLPGIMNPHSKFVQVWT------- : 170
AtCNGC20 : PYCTTCPSYYNRKAAQIPTS-RVSALFD----STFHNALYDDAKGWARRFASSVNRYLPGIMNPHAKEVQTWT------- : 204
                                                                          p       w

                    *       260         *       280         *       300         *       320
BrCNGC   : -------------KAILLVCVVALGVDPLFLFIPVIDSP---NFCFTFDKKLAAVVSAIRTFIDTFYVIHIIFNFITE-- :  97
BrCNGC2  : DDDPTNNVPDSIFESNYANCYHQATVDPLFFFIPVIDSH---KFCFTLDKKLGVAVCVLRTLIDVFYVIHFIFHFITE-- : 142
BrCNGC3  : -------------KIFLLLCVVALAFDPLFFFIPVVDPD---RFCLKLDKKLEAVACVFRTFIDAFYLVHMLFQFNTG-- : 144
BrCNGC4  : --------------IVLLVCLVALAIDPLFLFIPVIDSH---RLCFTYDKKLVATACVFRTLIDTFYGIHIIFHFITK-- :  69
BrCNGC5  : -------------KIFLFASVIALAIDPLFFYIPIVDGK---KHCINLHSSLEIAASVLRTFVDAFYIIHIVFQFRTA-- : 149
BrCNGC6  : -------------KIFVLACIIAVSVDPLFFYVPVLDKA---NNCLDVDKKMQTTASVLRSVTDIFYAIHMVFQFRTG-- : 185
BrCNGC7  : -------------KIFVLACIIAVSLDPLFFYVPVVDDA---KKCLGLDNKMEITASVLRSFTDIFYVIHIIFQFRTG-- : 153
BrCNGC8  : -------------RMLVISCILAVSVDPLFFYLPIVDNS--GSSCIGIDTKLAVTTTTLRTILDVFYLTRMALQFRTA-- : 135
BrCNGC9  : -------------KLFVISCILSVFVDPFFFYLPVIDGE---SKCLGIDRKLAITATTFRTFIDVFYLAHMALQLRTA-- : 191
BrCNGC10 : -------------KLFVASCILAVSVDPLFLYLPFINDK---AKCIGIDRKLAIVATTLRTVIDSFYLFHMALRFRTA-- : 176
BrCNGC11 : -------------KLFVASCILAVSVDPLFLFLPFINDK---AKCVGIDRKLAIVTTTLRTVIDSFYLFHMALRFRTA-- : 166
BrCNGC12 : -------------KLFVTSCILAVSVDPLFLYLPFINDS---GKCIGIDRRLATIATTLRTFIDVFYLFRMALQFRTA-- : 154
BrCNGC13 : -------------KLFLIACLVSLFVDPLFFFLPVMR---K-EACITIGIRLEVVLTVIRSLADAFYIAQIVIRFRTA-- : 148
BrCNGC14 : -------------HVFLVTSILALFLDPFYFYAPYVG---G-PACLSVDVGLAATVTFFRSVADLFHLLHIFMKFRTA-- : 113
BrCNGC15 : -------------HIFLITCLLALFLDPLYFYPPIVQA--G-TACMSIDIGFGILVTFFRTLADFSFLIHILLKFKTA-- : 119
BrCNGC16 : -------------WVFIISCMLALFIDPLYFFVPAIGGNKD-YPCAKTDTNLRILVTFFRTIADLFYLLHIFIKFRTG-- : 150
BrCNGC17 : -------------RVFLFWCLVALYVDPLFFFLSSVKNTGR-SSCMTTDLKLGIVITFFRTLADLFYVLHIVIKFRTA-- : 153
BrCNGC18 : -------------RVFLFWCLVALYVDPLFFFLYSVKRTGR-SSCMTTDLNLGIVVTFFRTLADLFYVLHIVIKFRTA-- : 149
BrCNGC19 : -------------RVFLLVCATGLFVDPLFLYTLSVNDAC---MCLLVDGWLALTVTALRSMTDLLHLWNILIQFKIARW : 156
BrCNGC20 : -------------KVFLLVCATGLFVDPLFLYTISVNDAC---MCLLVDGWLALSITAVRSMTDLLHLWNIWIQFKIARR : 156
BrCNGC21 : -------------RALLLARGMALAVDPLFFYALSIGRTTG-PACLYMDGAFAAVVTVVRTCLDALHLWHVWLQFRLA-- : 183
BrCNGC22 : -------------IFFAFSCLLSIFVDPLFFFPIQVSES---GKCIRIDEKMARVLVALRSLTDFLYFVNTLLQCRLA-- : 184
BrCNGC23 : -------------RLFSLSCLLSIFLDPLFVFLTLVKQN---NKCIVIDWPMAKAFIIVRSVTDALFSVNILLQFRLA-- : 270
BrCNGC24 : -------------KFFAVSSLLAIFIDPLFFFIILVQKN---NKCIVIDWPIATAFVIVRTLTDVIFFANMLLQFRLA-- : 230
BrCNGC25 : -------------RFLAFSCLMAIFIDPTFVFLLLIRHD---NKCIEIDWPKTTVLVSLRSMFDLIFFINILLQFRMA-- : 242
BrCNGC26 : -------------RFLAFSCLLSIFIDPAFVYLLLIRDD---NKCTDIDWPKATLFLFLRSMSDFIFFINILLQFRLA-- : 259
BrCNGC27 : -------------TFFVLSCLCSIFVDPLFFYPIEIYKE---ERCIKIDWWTTNVFVIVRTITDGLYALNIVLQFRLA-- : 224
BrCNGC28 : -------------KFFALSCLLAIFIDPLFFFLILVKQN---DKCIVIDWPMAKAFVAVRSVTDILFSVNILLQFRLA-- : 263
BrCNGC29 : -------------RFLAFSCLVAIFIDPAFLFLLSIRQD---NKCIEFDWPKTKVFVSLRSMSDLIFFMNILLQFRLA-- : 256
AtCNGC1  : -------------KIFVLACIIAVSLDPLFFYVPIIDDA---KKCLGIDKKMEITASVLRSFTDVFYVLHIIFQFRTG-- : 158
AtCNGC2  : -------------RALLLARGMALAVDPLFFYALSIGRTTG-PACLYMDGAFAAVVTVLRTCLDAVHLWHVWLQFRLA-- : 189
AtCNGC3  : -------------KIFLLLSVVALAFDPLFFYIPYVKPE---RFCLNLDKKLQTIACVFRTFIDAFYVVHMLFQFHTG-- : 146
AtCNGC4  : -------------KVFLLVCATGLFVDPLFLYTLSVSDTC---MCLLVDGWLALTVTALRSMTDLLHLWNIWIQFKIARR : 155
```

```
AtCNGC5  : -------------KLFVASCILSVFVDPFFFYLPVINAE---SKCLGIDRKLAITASTLRTFIDVFYLAHMALQLRTA-- : 162
AtCNGC6  : -------------KLFVASCILAVSVDPLFLYLPFINDK---AKCVGIDRKLAIIVTTIRTVIDSFYLFHMALRFRTA-- : 177
AtCNGC7  : -------------RLFVISCILAVSVDPLFFYLPIVDNS--GSSCIGIDTKLAVTTTTLRTIVDVFYLTRMALQFRTA-- : 136
AtCNGC8  : -------------RMFVISCILAVSVDPLFFYLPIVDNS---KNCIGIDSKLAVTTTTLRTIIDVFYLTRMALQFRTA-- : 171
AtCNGC9  : -------------KLFVTSCILAVSVDPLFLYLPFVKDN---EKCIGIDRKLAIIATTLRTVIDAFYLFHMALRFRTA-- : 177
AtCNGC10 : -------------KIFLFACVVALAIDPLFFYIPIVDSA---RHCLTLDSKLEIAASLLRTLIDAFYIIHIVFQFRTA-- : 137
AtCNGC11 : -------------KTVLLACVVALAIDPLFLFIPLIDSQ---RFCFTFDKTLVAVVCVIRTFIDTFYVIHIIYYLITE-- : 103
AtCNGC12 : -------------KTVLLACVVALAIDPLFLFIPLIDSQ---RFCFTFDKTLVAVVCVIRTFIDTFYVIHIIYYLITE-- : 103
AtCNGC13 : -------------KIFLFASVIALAIDPLFFYIPIVDGE---RHCINLHRNLEIAASVLRTFIDAFYIIHIVFQFRTA-- : 142
AtCNGC14 : -------------RVFLFWCLVALYVDPLFFFLSSVKRIGR-SSCMTTDLKLGIVITFFRTLADLFYVLHIVIKFRTA-- : 148
AtCNGC15 : -------------KIFLIACLVSLFVDPLFFFLPVMR---N-EACITIGVRLEVVLTLIRSLADAFYIAQILIRFRTA-- : 141
AtCNGC16 : -------------HIFLITCLIALFLDPLYFYLPIVQA--G-TACMSIDVRFGIFVTCFRNLADLSFLIHILLKFKTA-- : 118
AtCNGC17 : -------------WVFIVSCMVALFIDPLYFFVPAIGGDKN-YPCARTDTSLSILVTFFRTIADLFYLLHIFIKFRTG-- : 147
AtCNGC18 : -------------HVFLITSILALFLDPFYFYVPYVG---G-PACLSIDISLAATVTFFRTVADIFHLLHIFMKFRTA-- : 113
AtCNGC19 : -------------RVLAFSSLVAIFIDPLFFFLLLIQQD---NKCIAIDWRATKVLVSLRSITDLIFFINILLQFRLA-- : 232
AtCNGC20 : -------------KFFALSCLLAIFIDPLFFFLIKVQEQ---NKCIMIDWPMTKAFVAVRSVTDVIFTMNILLQFRLA-- : 266
                               c      DPl5 5   6       C   d         R   D         f

                    *       340        *       360        *       380        *       400
BrCNGC   : --FIAPRS---QVSLRGELIVHSKAIRKRLF--FFQFIVDICSVIPIP----------------------------QVVV : 142
BrCNGC2  : --LVAPRS---QASLRG----NSKPIRKRLF--FFYFSVDIVSVLPIP----------------------------QVMV : 183
BrCNGC3  : --FIAPSS---RGFGRGELVQSSKKIAVRYL--KSYFIIDVLSILPIP----------------------------QVVV : 189
BrCNGC4  : --HIAPRS---QVSFRGETTVYSVAISERHL--IFYFIVDIVSVLPIP----------------------------Q--- : 111
BrCNGC5  : --YVSPLS---RVFGRGELVEDPKAIALKYI--SSYFIIDVLSILPLP----------------------------QLVV : 194
BrCNGC6  : --FIAPSS---RVFGRGVLVEDRRKIAKRYL--SSHFIIDILAVLPLP----------------------------QMVI : 230
BrCNGC7  : --FIAPSS---RVFGRGVLVEDTRQIAIRYL--SSHFIIDILAVLPLPQVRIEIEIEIDLVLSTCISKPLMIVCFPQVVI : 226
BrCNGC8  : --YIAPSS---RVFGRGELVIDPAKIAQRYL--TRYFIVDFLAVLPLP----------------------------QIAV : 180
BrCNGC9  : --YIAPSS---RVFGRGELVIDPAQIAKRYL--QRWFIIDFLSVLPVP----------------------------QIVV : 236
BrCNGC10 : --YVAPSS---RVFGRGELVIDPKQIAKRYL--RQYFIIDLLSVLPLP----------------------------QITV : 221
BrCNGC11 : --YVAPSS---RVFGRGELVIDPAQIAKRYL--QQYFIIDLLSVLPVP----------------------------QIIV : 211
BrCNGC12 : --FVAPSS---RVFGRGELVIDPAQIAKRYL--QQYFIVDFLSVLPLP----------------------------QIVV : 199
BrCNGC13 : --YIAPSS---RVFGRGELVIDSRKIAWRYL--NKSFWIHLVAALPLP----------------------------QVLI : 193
BrCNGC14 : --FVARSS---RVFGRGELVRDPREIAMKYL--KSDFIVDVAAMLPLP----------------------------QLVI : 158
BrCNGC15 : --FVSKSS---RVFGRGELVIDRREIAIRYL--KSEFIIDLAATLPLP----------------------------QIMI : 164
BrCNGC16 : --FIAPNSST-RVFGRGELVMDPKAIAWRYL--KSDFIIDLVATLPLP----------------------------QIVI : 197
BrCNGC17 : --YVSRTS---RVFGRGELVKDPKLIARRYL--RSDFIVDLIACLPLP----------------------------QIVS : 198
BrCNGC18 : --YVSRTS---RVFGRGELVKDPKLIARRYL--RSDFIVDLIACLPLP----------------------------QIVS : 194
BrCNGC19 : WPYRGGDSDGDINKGDGTRVRM--RGAPPYVKKNG-FFFDLFVILPLP----------------------------QVVL : 205
BrCNGC20 : WPYPGGDSDGDTNKGDETRLRTSRRVAPPYVKKKGTFFFDLFVILPLP----------------------------QVVL : 208
BrCNGC21 : --YVSRES---LVVGCGKLVWDPRAIASHYARSLTGFWFDVIVILPVP----------------------------QAVF : 230
BrCNGC22 : --YTDPKS---TVVGSGQLVKGSVEIAKRYY--RGNFLLDLVIVIPLP----------------------------QILI : 229
BrCNGC23 : --YVSPES---MVVGVTWLVDHPVKIARHYF--QGNFFLDLFIVMPLP----------------------------QILI : 315
BrCNGC24 : --YVARES---TVVGAGQLV-----------------------------------------------------------LV : 247
BrCNGC25 : --YVAPES---RIVGAGQLVDHPRKIARNYL--RGKFFLDLFIVLPIP----------------------------QIMT : 287
BrCNGC26 : --YVAPES---RIVGAGQLVDHPRKIACNYL--RGKFFLDVVIVYPVP----------------------------QLMI : 304
BrCNGC27 : --YVDLES---TVAGAGQLVDDPKKIASHYL--RGKFLTDFFIVLPIP----------------------------QILL : 269
BrCNGC28 : --YVAPES---TVVGAGQLVAHPRKIARHYF--RGKFLLDLFIVMPLP----------------------------QILI : 308
BrCNGC29 : --YVAPES---RIVGAGQLVDHPRKIASNYL--RGKFLLDLLIVLPMP----------------------------QIMI : 301
AtCNGC1  : --FIAPSS---RVFGRGVLVEDKREIAKRYL--SSHFIIDILAVLPLP----------------------------QMVI : 203
AtCNGC2  : --YVSRES---LVVGCGKLVWDPRAIASHYARSLTGFWFDVIVILPVP----------------------------QAVF : 236
AtCNGC3  : --FITPSS---SGFGRGELNEKHKDIALRYL--GSYFLIDLLSILPIP----------------------------QVVV : 191
AtCNGC4  : WPYPGGDSDGDTNKGGGTRGST--RVAPPYVKKNG-FFFDLFVILPLP----------------------------QVVL : 204
AtCNGC5  : --YIAPSS---RVFGRGELVIDPAQIAKRYL--QRWFIIDFLSVLPLP----------------------------QIVV : 207
AtCNGC6  : --YVAPSS---RVFGRGELVIDPAQIAKRYL--QQYFIIDLLSVLPVP----------------------------QIIV : 222
AtCNGC7  : --YIAPSS---RVFGRGELVIDPAKIAERYL--TRYFVVDFLAVLPLP----------------------------QIAV : 181
AtCNGC8  : --YIAPSS---RVFGRGELVIDPAKIAERYL--TRYFIVDFLAVLPLP----------------------------QIAV : 216
AtCNGC9  : --FVAPSS---RVFGRGELVIDPAQIAKRYL--QQYFIIDFLSVLPLP----------------------------QIVV : 222
AtCNGC10 : --YIAPSS---RVFGRGELVDDAKAIALKYL--SSYFIIDLLSILPLP----------------------------QIVV : 182
AtCNGC11 : --TIAPRS---QASLRGEIVVHSKATLKTRL--LFHFIVDIISVLPIP----------------------------QVVV : 148
AtCNGC12 : --TIAPRS---QASLRGEIVVHSKATLKTRL--LFHFIVDIISVLPIP----------------------------QVVV : 148
AtCNGC13 : --YISPSS---RVFGRGELVDDPKAIAIKYL--SSYFIIDLLSILPLP----------------------------QLVV : 187
AtCNGC14 : --YVSRTS---RVFGRGELVKDPKLIARRYL--RSDFIVDLIACLPLP----------------------------QIVS : 193
AtCNGC15 : --YIAPPS---RVFGRGELVIDSRKIAWRYL--HKSFWIHLVAALPLP----------------------------QVLI : 186
AtCNGC16 : --FVSKSS---RVFGRGELVMDRREIAIRYL--KSEFVIDLAATLPLP----------------------------QIMI : 163
AtCNGC17 : --FIAPNSST-RVFGRGELVMDPKAIAWRYI--KSDFIIDLIATLPLP----------------------------QIVI : 194
```

```
AtCNGC18 : --FVARSS---RVFGRGELVMDSREIAMRYL--KTDFLIDVAAMLPLP----------------------------QLVI : 158
AtCNGC19 : --YVAPES---RIVGAGQLVDHPRKIARHYF--RGKFLLDMFIVFPIP----------------------------QIMI : 277
AtCNGC20 : --YVARES---TVVGAGQLVSHPKKIALHYL--KGKFFLDLFIVMPLP----------------------------QILI : 311
                  S      g g         a  y      f  d     p p                             q

                    *        420          *        440          *        460          *        480
BrCNGC   : LILIH-RS---DSLVSQAILKWIILTQYIPRIIRIYPLLKEVTRASGTIAETKWVGAAFNLFLYMLHSHVFGAFWYVSSV : 218
BrCNGC2  : LTLLS-RKQK-TSLVSKEILKWAMFCQSIPRSIRIYPIYKNGTKLYGRVAVIKWVGAALNLFFYLLPSHVIGAIWYLSAV : 261
BrCNGC3  : LAVVP-SMSRPASLVTKELLKWAIFCQYVPRIARIYPLFKEVTRTSGLVTETAWAGAALNLFLYMLASHVFGSFWYLISI : 268
BrCNGC4  : -------------------------------------------------------------------VFGAFWYLSAI : 122
BrCNGC5  : LAVIP-NVDKPVSLLTKDYLITVIFAQYIPRILRIYPLYSEVTRTSGIVTETAWAGAAWNLSLYMLASHVFGALWYLISV : 273
BrCNGC6  : SIIIP-RMREPKTLHTKNMLKFIVFFQYIPRFMRIYPLYKQVTRTSGILTETAWAGAAFNLFLYMLASHVFGAFWYLFSI : 309
BrCNGC7  : LIIIP-HMRGSRSLNTKNLLKFIVFFQYIPRFIRIYPLYKEVTRTSGILTETAWAGAAFNLFLYMLASHVFGAFWYLFSI : 305
BrCNGC8  : WKFLH-GSKGMDVLPTKTALLNIVITQYIPRFVRFIPLTSELKKTAGAFAEGAWAGAAYYLLWYMLASHITGAFWYMLSV : 259
BrCNGC9  : WRFLQ-SSRGSDVLATKQALLFIVLVQYIPRFLRVLPLTSELKRTAGVFAETAWAGAAYYLMLYMLASHIVGAFWYLLAL : 315
BrCNGC10 : WRFLY-TSKGANVLATKQALRYIVLVQYIPRFVRMYPLSSELKRTAGVFAETAWAGAAYYLLLYMLASHIVGALWYLLAL : 300
BrCNGC11 : WRFLY-SSRGANVLATKQALRYIVLVQYIPRFLRMYPLSSELKRTAGVFAETAWAGAAYYLLLYMLASHIVGALWYLLAL : 290
BrCNGC12 : WRFLY-TSTGGSVLETKQALRSIILVQYIPRFIRIYPLSKELKRTAGVFAETAWAGAAYYLLLYMLASHIVGALWYLLAL : 278
BrCNGC13 : WIVIP-NLRGSPMTNTKNTLRFIIIFQYVPRMFLIFPLSRQIIKATGVVTETAWAGAAYNLMLYMLASHVLGACWYLLAV : 272
BrCNGC14 : WLVIP-AATNGTANHANSTLALIVLVQYIPRSFIIFPLNQRIIKTTGFIAKTAWAGAAYNLLLYILASHVLGAMWYLSSI : 237
BrCNGC15 : WFVIPNAGEFRYAAHQNHTLSLVVLIQYVPRILVMLPLNRRIIKATGVAAKTAWSGAAYNLVLYLLVSHVLGSVWYVLSI : 244
BrCNGC16 : WFVMP-ATRSYRFDHSNNDLALIVLLQYIPRFYLIIPLSSQIVKATGVVTKTAWAGAAYNLLLYMLASHVLGAAWYILSF : 276
BrCNGC17 : WFILP-SIRSSHSDHTTNALVLIVLVQYIPRLYLIFPLSAEIIKATGVVTTIAWAGAAYNLLQYMLASHILGAAWYLLSI : 277
BrCNGC18 : WFILP-SIRSSHSDHTTNALVLIVLVQYIPRLYLIFPLSAEIIKATGVVTTIAWAGAAYNLLQYMLASHILGAAWYLLSI : 273
BrCNGC19 : WVVIPSLLKRGSVTLVVSILLLTFLFQYLPKIYHSIRHLRQNATLSGYIFGTVWWGFALNMVAYFVAAHAAGACWYLLGV : 285
BrCNGC20 : WVVIPSLLKRGSVTLVVSVLLVTFLFQYLPKIYHSVRHLRQNATLSGYIFGTVWWGIALNMIAYFVAAHAAGACWYLLGV : 288
BrCNGC21 : WLVVPKLIREEKVKLIMTILLLIFLFQFLPKIYHCICLMRRMQKVTGYIFGTIWWGFALNLIAYFIASHVAGGCWYVLAI : 310
BrCNGC22 : LWIIPHVLVIYGANTIKNLLCTAVVVQCIPKLHRLFHLLAR-KRTTGFIFESAWASFFINLFTYILAGHIVGSCWYLFGL : 308
BrCNGC23 : SWITPAWLGGSWENNAYSLLQAAVLLQYTLKLYRLLPLLAG-KTPIGFILESS-SKFAINFLTFMLAGHVVGSCWYLFGL : 393
BrCNGC24 : LSVIPAQLAISGTNYAKNLLRTGIIVQYIPKLYRLLPLLAG-QTPTGLIFESAWANFVINLLTFMLAGHVVGSCWYLFGL : 326
BrCNGC25 : LSILPAHLGTSTAGFERNIIRSTFIVQYIPKLYRLLPLLAG-QTPTGFIFESAWASFVINLLTFMLAGHAVGSCWYFSGI : 366
BrCNGC26 : LSILP------KSEFEENAIIGIFLFQYIPKLYRLLPLIAG-QTPTGFIFESAWSNFIINLLTFILAGHAVGSFWYLAGL : 377
BrCNGC27 : LWIIPQLLGTSGANNTKNYLRAAILVQYIPKLRRLFPLLAG-QTPRGFRFDSALAKFFINLLTFMLAGHVIGSCWYLLGL : 348
BrCNGC28 : LWIIPAHLGASGANYAKNLLRAAVLFQYIPKLYRLLPLLAG-QTPTGFIFESAWANFVINLLTFMLAGHVVGSCWYLFGL : 387
BrCNGC29 : LSMLPAHLGTPRSELEKNIIRIVFLFQYIPKLYRLLPLLAG-QTPTGFIFESAWANFIINLLTFILAGHAIGSFWYLAGL : 380
AtCNGC1  : LIIIP-HMRGSSSLNTKNMLKFIVFFQYIPRFIRIYPLYKEVTRTSGILTETAWAGAAFNLFLYMLASHVFGAFWYLFSI : 282
AtCNGC2  : WLVVPKLIREEKVKLIMTILLLIFLFQFLPKIYHCICLMRRMQKVTGYIFGTIWWGFALNLIAYFIASHVAGGCWYVLAI : 316
AtCNGC3  : LAIVP-RMRRPASLVAKELLKWVIFCQYVPRIARIYPLFKEVTRTSGLVTETAWAGAALNLFLYMLASHVFGSFWYLISI : 270
AtCNGC4  : WVVIPSLLKRGSVTLVVSVLLVTFLFQYLPKIYHSIRHLRRNATLSGYIFGTVWWGIALNMIAYFVAAHAAGACWYLLGV : 284
AtCNGC5  : WRFLQ-SSNGSDVLATKQALLFIVLVQYIPRFLRVLPLTSELKRTAGVFAETAWAGAAYYLLLYMLASHIVGAFWYLLAL : 286
AtCNGC6  : WRFLY-TSRGANVLATKQALRYIVLVQYIPRFLRMYPLSSELKRTAGVFAETAWAGAAYYLLLYMLASHIVGALWYLLAL : 301
AtCNGC7  : WKFLH-GSKGSDVLPTKTALLNIVIVQYIPRFVRFIPLTSELKKTAGAFAEGAWAGAAYYLLWYMLASHITGAFWYMLSV : 260
AtCNGC8  : WKFLH-GSKGTDVLPTKQALLHIVITQYIPRFVRFIPLTSELKKTAGAFAEGAWAGAAYYLLWYMLASHITGAFWYMLSV : 295
AtCNGC9  : WRFLY-ISKGASVLATKRALRSIILVQYIPRFIRLYPLSSELKRTAGVFAETAWAGAAYYLLLYMLASHIVGAIWYLLAL : 301
AtCNGC10 : LAVIP-SVNQPVSLLTKDYLKFSIIAQYVPRILRMYPLYTEVTRTSGIVTETAWAGAAWNLSLYMLASHVFGALWYLISV : 261
AtCNGC11 : LTLLP-LS---ASLVSERILKWIILSQYVPRIIRMYPLYKEVTRAFGTVAESKRVGAALNFFLYMLHSYVCGAFWYLSSI : 224
AtCNGC12 : LTLLP-LS---ASLVSERILKWIILSQYVPRIIRMYPLYKEVTRAFGTVAESKWAGAALNLFLYMLHSYVFGAFWYLSSI : 224
AtCNGC13 : LAVIP-NVNKPVSLITKDYLITVIFTQYIPRILRIYPLYTEVTRTSGIVTETAWAGAAWNLSLYMLASHVFGALWYLISV : 266
AtCNGC14 : WFILP-SIRSSHSDHTTNALVLIVLVQYIPRLYLIFPLSAEIIKATGVVTTIAWAGAAYNLLQYMLASHILGSAWYLLSI : 272
AtCNGC15 : WIIIP-NLRGSPMTNTKNVLRFIIIFQYVPRMFLIFPLSRQIIKATGVVTETAWAGAAYNLMLYMLASHVLGACWYLLAV : 265
AtCNGC16 : WFVIPNAGEFRYAAHQNHTLSLIVLIQYVPRFLVMLPLNRRIIKATGVAAKTAWSGAAYNLILYLLVSHVLGSVWYVLSI : 243
AtCNGC17 : WFVIS-TTKSYRFDHNNNAIALIVLLQYIPRFYLIIPLSSQIVKATGVVTKTAWAGAAYNLLLYMLASHVLGAAWYILSV : 273
AtCNGC18 : WLVIP-AATNGTANHANSTLALIVLVQYIPRSFIIFPLNQRIIKTTGFIAKTAWAGAAYNLLLYILASHVLGAMWYLSSI : 237
AtCNGC19 : LRIIPLHLGTRREESEKQILRATVLFQYIPKLYRLLPLLAG-QTSTGFIFESAWANFVINLLTFMLAGHAVGSCWYLSAL : 356
AtCNGC20 : LWIIPAHLGASGANYAKNLLRAAVLFQYIPKLYRLLPFLAG-QTPTGFIFESAWANFVINLLTFMLAGHVVGSCWYLFGL : 390
                                             q  p       p          g      w   a         a h  G  WY

                    *        500          *        520          *        540          *        560
BrCNGC   : EKKNKCWRLECAKI------SGCNLR--HQYCAR----------GRENNGRYLNTTCPLIDPDQIIG-STVFNFGMYTDA : 279
BrCNGC2  : EKKETCWREACAKI------DECDLT--NLLCARG---------AGGDNSRFLNTSCPLIDPEQITN-STVLNFGIYTDA : 323
BrCNGC3  : ERKDRCWRETCAKI------EGCVHG--NLYCS-----------GGEDNSQYLIGSCPLMDPEEIKN-STVLNFGIFAEA : 328
BrCNGC4  : EKKNRCWSDACAKS------SMCNLTNLDLYCVR----------GGGDNSHFLKISCPLIDPGEITN-STVFNFGMYIDA : 185
BrCNGC5  : ERREDRCWREACGKR------QGCELR--DLYCDG----------NNNVINDYLTTSCPFINPDDITN-STTFNFGIFTDA : 334
BrCNGC6  : ERKTVCWKQAWNRAG----RSKCDMR--SLYCAR----------EHYGNNTFLNGSCPVLKP----N-ATCFEFGIFLGA : 368
BrCNGC7  : ERETVCWKQACNRN-----RNICDIT--SLYCDH----------KAAGGNAFLNASCPVQTP----N-ATLFDFGIFLNA : 363
```

```
BrCNGC8  : ERNDTCWRFACKVQP----DPKLCVQ--ILYCGTKFVSSRET-EWIKTVPELLKSNCSAKADD------AKFNYGIYGQA : 326
BrCNGC9  : ERNDACWQEACSDAG----KKICTTG--FLYCGNQNMDG-YD-VWNKTKEAVLESRCRADLDD---P-NPPFDFGIYTQA : 383
BrCNGC10 : ERNNDCWSKACV-KK----DN-CTRN--FLFCGNQNMEG-YA-AWYTAKSSVLQEMCPVNVTEG--E-EPPFDFGIYSRA : 367
BrCNGC11 : ERNNDCWSKACK-DN----DN-CTRN--FLFCGNQNMKG-YD-AWDDVKDPFLQLRCPVNVTEG--E-EPPFDFGIYLRA : 357
BrCNGC12 : ERVNGCWKKACLVDG----QN-CTRN--FLFCGNENMDG-YA-AWNTIKESVLQKSCPVNVTDG--D-NPPFDFGIYLRA : 346
BrCNGC13 : ERQEACWRHACNIEK-----PICQYR--FFECRR---------LEDPQRNSWFEWSNITTICKPGTR---FYEFGIYGDA : 333
BrCNGC14 : GRQFSCWSKVCEKDH-ALRVLDCLPS--FLDCKS---------LEQPERQYWQNVTQVLSHCDATSS-TTNFKFGMFAEA : 304
BrCNGC15 : QRQHECWRRECIKEMNATHSPSCNLL--FLDCGS---------LRDPGRQAWMRITRVLSNCDARNDDDQHFQFGMFGDA : 313
BrCNGC16 : DRYTSCWKTRCNKEH---GGVNCYLY--YLDCDSP--------LYDARQQQWANVTNVFKLCDARK---GEFKYGMFENA : 340
BrCNGC17 : ERQATCWKAECHNEL---GPIRCVTD--FFDCGT---------VNREDRNNWQNVTVVFSNCDPSNK--IRFTFGIFADA : 341
BrCNGC18 : QRQATCWKAECHKEF---APLECVTD--FFDCGT---------LHRPDRNNWQNITVVFSNCDPSND--IKFTFGIFADA : 337
BrCNGC19 : QRSAKCLKEQCENTMG------CDLR--MLSCKEPVYYGTTEMVLDRARLAWARNHQ---ARSVCLDIDTDYTYGAYQWT : 354
BrCNGC20 : QRSAKCLTEQCESTMG------CDLR--MLSCKEPVYYGTTEMVLDRARLAWAQNNQ---ARSICLDINTNYTYGAYKWT : 357
BrCNGC21 : QRVASCIRQQCMRTAN------CNL---SLSCQEEVCYQFVSPSSTIGFPCVSGNLTSVVKKPMCLDSDGPFRYGIYGWA : 381
BrCNGC22 : QRVNRCLRETCGDHME---SKCHGVCKELIDCGLRLKEKLID--CERRNTTVTQAVLNWNVTAAACFQENGFPYGIYWKA : 383
BrCNGC23 : QRVNQCLRDACGN--------TDRACRELIDCG-------------RGSSDVVLAALKYNTSASACFQENGFPYGIYLKA : 452
BrCNGC24 : QRVNQCLRN-ACGH-------SGRECRGLIDCGHGN----------SNISASLRAIWRNSASANACFQEDGFSYGIYLKM : 388
BrCNGC25 : ERVKKCLLYAAWNN-------SVDERRNLIDCARGN----------IYASASLRARWRDSDSVNACFQESGFSYGIYAKA : 429
BrCNGC26 : QRVNKCLLQ-AGNF-------TMDERRYLIDCTSGE----------SYLR----ALWRDSESVNACFQDGGFSFGIYLNA : 435
BrCNGC27 : QRVNQCLRDACGN--------SSFECKQLIDCG-------------RENRTEVLHAWKINVSANACFQEDGFDYGIYLKA : 407
BrCNGC28 : QRVNQCLRD-ACGN-------SDHECRNLIDCGRGE----------S--SEAF-AAWKGNASASASCFQEGGFPYGIYMKA : 446
BrCNGC29 : QRVKKCLLH-AGNY-------TMDERRNLIDCAHGV----------NYARESQIALWRDSESVNACFQEGGFSFGIYLKA : 442
AtCNGC1  : ERETVCWKQACERN-----NPPCISK--LLYCDP----------ETAGGNAFLNESCPIQTP----N-TTLFDFGIFLDA : 340
AtCNGC2  : QRVASCIRQQCMRTGN------CNL---SLACKEEVCYQFVSPTSTVGYPCLSGNLTSVVNKPMCLDSNGPFRYGIYRWA : 387
AtCNGC3  : ERKDRCWREACAKI------QNCTHA--YLYCSP----------TGEDNRLFLNGSCPLIDPEEITN-STVFNFGIFADA : 331
AtCNGC4  : QRSAKCLKEQCENTIG------CDLR--MLSCKEPVYYGTTVMVLDRARLAWAQNHQ---ARSVCLDINTNYTYGAYQWT : 353
AtCNGC5  : ERNDACWQEACIDAG----N--CSTD--FLYCGNQNMDG-YA-VWNRAKESVLKSKCRADLDD---N-NPPFDFGIYTQA : 352
AtCNGC6  : ERNNDCWSKACH-NN----QN-CTRN--FLFCGNQNMKG-YA-AWDNIKVSYLQLKCPVNVPED--E-EPPFDFGIYLRA : 368
AtCNGC7  : ERNDTCWRFACKVQP----DPRLCVQ--ILYCGTKFVSSGET-EWIKTVPELLKSNCSAKADD------SKFNYGIYGQA : 327
AtCNGC8  : ERNDTCLRSACKVQP----DPKVCVQ--ILYCGSKLMSSRET-DWIKSVPDLFKNNCSAKSDE------SKFNYGIYSQA : 362
AtCNGC9  : ERYNGCWTKVCSNSS----LD-CHRN--FLFCGNEKMDG-YA-AWTTIKDSVLQLNCPVNTTD-----NPPFDFGIYLRA : 367
AtCNGC10 : EREDRCWQEACEKT------KGCNMK--FLYCEN----------DRNVSNNFLTTSCPFLDPGDITN-STIFNFGIFTDA : 322
AtCNGC11 : ERKSTCWRAACART------SDCNLTVTDLLCKR----------AGSDNIRFLNTSCPLIDPAQITN-STDFDFGMYIDA : 287
AtCNGC12 : ERKSKCWRAACART------SDCNLTVTDLLCKR----------AGSDNIRFLNTSCPLIDPAQITN-STDFDFGMYIDA : 287
AtCNGC13 : EREDRCWREACEKIP-----EVCNFR--FLYCDG----------NSSVRNDFLTTSCPFINPDDITN-STVFNFGIFTDA : 328
AtCNGC14 : ERQATCWKAECHKES---VPLQCVTD--FFDCGT---------LHRDDRNNWQNTTVVFSNCDPSNN--IQFTFGIFADA : 336
AtCNGC15 : ERQEACWRHACNIEK-----QICQYR--FFECRR---------LEDPQRNSWFEWSNITTICKPASK---FYEFGIFGDA : 326
AtCNGC16 : QRQHECWRRECIKEMNATHSPSCSLL--FLDCGS---------LHDPGRQAWMRITRVLSNCDARNDDDQHFQFGMFGDA : 312
AtCNGC17 : DRYTSCWKSRCNGEA---GQVNCQLY--YLDCDS---------MYDNNQMTWANVTKVFKLCDARN---GEFKYGIFGNA : 336
AtCNGC18 : GRQFSCWSNVCKKDN-ALRVLDCLPS--FLDCKS---------LEQPERQYWQNVTQVLSHCDATSS-TTNFKFGMFAEA : 304
AtCNGC19 : QRVKKCMLN-AWNI-------SADERRNLIDCARG-----------SYASKSQRDLWRDNASVNACFQENGYTYGIYLKA : 417
AtCNGC20 : QRVNQCLRN-ACGN-------FGRECQDLIDCGNGN----------S--SVLVRATWKDNASANACFQEDGFPYGIYLKA : 450
            4   C    c                       C                                                5G 5   a

                      *        580         *         600         *         620         *         640
BrCNGC   : IRSGIVESKPRDFPRKFFYCF------------WWGL---------------------------RNISALGQNLKTSNSV : 320
BrCNGC2  : IKSGVVETR--DFPRKLLYCF------------WWGL---------------------------RNISALGQNLKTSNSA : 362
BrCNGC3  : IQSGVVESM--NFPKKFFYCF------------WWGL---------------------------RNLSAVGQNLKTSDFE : 367
BrCNGC4  : IKSGVVESR--DFPRKFFYCF------------WWGL---------------------------RNLSALGQNLETSNSV : 224
BrCNGC5  : IKSGIVKSD--DFWKKFFYCF------------WWGL---------------------------RNLSALGQNINTSKFV : 373
BrCNGC6  : IESGVVESH--DFPQKFFYCF------------WWGL---------------------------QNLSSLGQNLKTSTYI : 407
BrCNGC7  : IQSGVVESQ--DFPQKFFYCF------------WWGL---------------------------QNLSSLGQNLKTSTYI : 402
BrCNGC8  : ISSGIVSST--TFFSKFCYCL------------WWGL---------------------------QNLSTLGQGLQTSTFP : 365
BrCNGC9  : ISSGIVSSQ--KFITKYCYCL------------WWGL---------------------------QNLSTLGQGLETSTYP : 422
BrCNGC10 : ISSGIVSSK--KFVSKYFFCL------------WWGL---------------------------QNLSTLGQGLETSTYP : 406
BrCNGC11 : ISSGIVSSK--KFVSKYFFCL------------WWGL---------------------------QNLSTLGQGLETSTYP : 396
BrCNGC12 : ISSGIVSSE--SFVAKYFFCL------------WWGL---------------------------QNLSTLGQGLETSTYP : 385
BrCNGC13 : VTSTVTSSN---FISKYFYCL------------WWGL---------------------------KNLSSLGQNLSTSTYV : 371
BrCNGC14 : FTTQVATTD---FVSKYLYCL------------WWGL---------------------------RNLSSYGQNITTSVYL : 342
BrCNGC15 : FTNDVTSSP---FFDKYFYCL------------WWGL---------------------------RNLSSYGQSLAASTLS : 351
BrCNGC16 : ITKKVVSSN---FNERYFYCL------------WWGL---------------------------QQLSSYGQNLSTTTFI : 378
BrCNGC17 : ITKNVVSSP---FLEKYLYCL------------WFGL---------------------------QQLSSYGQNLDTSTSV : 379
BrCNGC18 : ITKNVVSSP---FLEKYLYCL------------WFGL---------------------------QNLSSYGQNLDTSTSV : 375
BrCNGC19 : IQLVSNESR----LEKVLFPI------------FWGL---------------------------MTLSTFGN-LESTTEW : 390
BrCNGC20 : IQLVSNESR----LEKILFPI------------FWGL---------------------------MTLSTFGN-LESTTEW : 393
```

```
BrCNGC21 : LPVISSNSL----AVKILYPI------------FWGL---------------------------MTLSTFGNDLEPTSNW : 418
BrCNGC22 : VNLTHDSN----LPKKYIYSL------------FWGF---------------------------QQISTLAGNLVPTDFV : 420
BrCNGC23 : VNLTNQSSL---ITIHKIHLLSFLGFSANLIRMFYGFDIKSALVGICGSLTLAYCQLSIVYYSPKQISTLAGNQVPSYFI : 529
BrCNGC24 : VNLTTHTS----LLTRYSYSL------------LWGF---------------------------QVMVYSN--------- : 416
BrCNGC25 : VNLTSHSS----IFTRFSYSL------------FWGF---------------------------QQISTLAGNLSPSYSV : 466
BrCNGC26 : ANLTTQRN----IFTRFSFSL------------FWGF---------------------------QQISTLAGNQTPSYSV : 472
BrCNGC27 : VNLTSNRTR---WYRRYSYSL------------FWGF---------------------------QQISTLAGNQVPSYFF : 445
BrCNGC28 : VNLTNHTS----LFTRYSYSL------------FWGF---------------------------QQISTLAGNQVPSYFL : 483
BrCNGC29 : VNLTTQPN----IFTRFSYSL------------FWGF---------------------------QQISTLAGNQTPTYSV : 479
AtCNGC1  : LQSGVVESQ--DFPQKFFYCF------------WWGL---------------------------QNLSSLGQNLKTSTYI : 379
AtCNGC2  : LPVISSNSL----AVKILYPI------------FWGL---------------------------MTLSTFANDLEPTSNW : 424
AtCNGC3  : LQSGVVESR--DFPKKFFYCF------------WWGL---------------------------RNLSALGQNLKTSAFE : 370
AtCNGC4  : IQLVSSESR----LEKILFPI------------FWGL---------------------------MTLSTFGN-LESTTEW : 389
AtCNGC5  : LSSGIVSSQ--NFIVKYCYCL------------WWGL---------------------------QNLSTLGQGLETSTYP : 391
AtCNGC6  : LSSGIVSSK--NFVSKYFFCL------------WWGL---------------------------QNLSTLGQGLETSTYP : 407
AtCNGC7  : ISSGIVSST--TFFSKFCYCL------------WWGL---------------------------QNLSTLGQGLQTSTFP : 366
AtCNGC8  : VSSGIVSST--TFFSKFCYCL------------WWGL---------------------------QNLSTLGQGLQTSTYP : 401
AtCNGC9  : LSSGIVSSK--SFVSKYFFCL------------WWGL---------------------------QNLSTLGQGLETSTYP : 406
AtCNGC10 : LKSGVVESH--DFWKKFFYCF------------WWGL---------------------------RNLSALGQNLQTSKFV : 361
AtCNGC11 : LKSGVLEVKPKDFPRKFVYCF------------WWGL---------------------------RNISALGQNLETSNSA : 328
AtCNGC12 : LKSGVLEVKPKDFPRKFVYCF------------WWGL---------------------------RNISALGQNLETSNSA : 328
AtCNGC13 : LKSGIVESD--DFWKKFFYCF------------WWGL---------------------------RNLSALGQNLNTSKFV : 367
AtCNGC14 : LTKNVVSSP---FLEKYLYCL------------WFGL---------------------------QNLSSYGQNLSTSTSV : 374
AtCNGC15 : VTSTVTSSK---FINKYFYCL------------WWGL---------------------------KNLSSLGQNLATSTYA : 364
AtCNGC16 : FTNDVTSSP---FFDKYFYCL------------WWGL---------------------------RNLSSYGQSLAASTLS : 350
AtCNGC17 : ITKNVVSSQ---FFERYFYCL------------WWGL---------------------------QQLSSYGQNLSTTMFM : 374
AtCNGC18 : FTTQVATTD---FVSKYLYCL------------WWGL---------------------------RNLSSYGQNITTSVYL : 342
AtCNGC19 : VNLTNESS----FFTRFSYSL------------YWGF---------------------------QQISTLAGNLSPSYSV : 454
AtCNGC20 : VNLTNHSN----LFTRYSYSL------------FWGF---------------------------QQISTLAGNQVPSYFL : 487
                                          5Gl                             6s

                      *        660         *        680         *        700         *        720
BrCNGC   : GDIVFALIICVSGLLLFAVLIGNIQKYLQSTTIRLDEMEEKKRDTEKWMSNRMLPEYLKERIRRYENYKWRKTRGIEEEA : 400
BrCNGC2  : GEVFFAIIICVSGVLLFAGLIGNVQKYLQSTSIRVDEWEAKKRDTEQWMPSEDLPDDLEKCI------EWEKNSSIEREA : 436
BrCNGC3  : GEIIFAIIICISGLVLFALLIGNMQKYLQSTTVRIEEMRVKRRDAEQWMSHRMLPDDLRKRVREYEQYKWQETRGVEEEA : 447
BrCNGC4  : GEIFFAITICVSGLLLFSVLIGNVQKYLQSTIVRINEMEEKRRDTEKWMSYRMLPESLKERIRKSEDHKWRETRGTKEES : 304
BrCNGC5  : GEIIFAVLICISGLVLFALLIGNMQKYLESTTVREEEMRVRKRDAEQWMAHRMLPEDLRKRIRRYEQYKWQETRGVEEEN : 453
BrCNGC6  : WENCFAVFISISGLVLFSFLIGNMQTYLQSTTTRLEEMRVKRRDAEQWMSHRLLPDNLRKRIRRYEQYKWQETRGVNEEN : 487
BrCNGC7  : WEICFAVFISISGLVLFSFLIGNMQTYLQSTTTRLEEMRVKRRDAEQWMAHRLLPESLRKRIRRYEQYKWQETRGVDEEN : 482
BrCNGC8  : GEVLFSIAIAIAGLLLFALLIGNMQTYLQSLTVRLEEMRIKRRDSEQWMHHRSLPQNLRERVRRYDQYKWLETRGVDEEN : 445
BrCNGC9  : LEIMFSITLAISGLILFALLIGNMQTYLQSLTIRLEEMRVKRRDSEQWMHHRMLPQDLRQRVRRYDQYKWLETRGVDEEY : 502
BrCNGC10 : GEVIFSIALAIAGLLLFALLIGNMQTYLQSLTIRLEEMRVKRRDSEQWMHHRMLPPELRERVRRYDQYKWLETRGVDEEN : 486
BrCNGC11 : GEVIFSIVLAIAGLLLFALLIGNMQTYLQSLTIRLEEMRVKRRDSEQWMHHRMLPPELRERVRRYDQYKWLETRGVDEEN : 476
BrCNGC12 : GEIIFSIALAVAGLLLFALLIGNMQTYLQSLTIRLEEMRVKRRDSEQWMHHRMLPPDLRERVRRYDQYKWLETKGVDEEN : 465
BrCNGC13 : GEIIFAVVMATLGLVLFALLIGNMQTYLQSTTMRLEEWRIRRTDTEQWMHHRQLPPELRQAVRKYDQYKWLATRGVDEEA : 451
BrCNGC14 : GETLFCITICIFGLILFTLLIGNMQTSLQSMSVRVEEWRIKRRDTEEWMRHRQLPPELQERVRRFVQYKWLATRGVDEES : 422
BrCNGC15 : SETLFSCFICVAGLVFFSHLIGNVQNYLQSTTARLDEWRVRRRDTEEWMRHRQLPQELQERVRRFVQYKWLTTRGVDEEA : 431
BrCNGC16 : GETTFAVLIAIFGLVLFAHLIGNMQTYLQSLTVRLEEWRLKKRDTEEWMRHRQLPEDLRDRVRRYEQYKWLAARGVDEEV : 458
BrCNGC17 : LETMFAILVAIFGLVLFALLIGNMQTYLQSITVRLEEWRLKRRDTEEWMGHRQLPQNLRERVRRFVQYKWLATRGVDEET : 459
BrCNGC18 : LETMFAILVAIFGLVLFALLIGNMQTYLQSITVRLEEWRLKRRDTEEWMGHRQLPQDLRERVRRFVQYKWLATRGVDEET : 455
BrCNGC19 : SEVVFNIIVLTSGLLLVTMLIGNIKVFLHATTSKKQAMHLKMRNIEWWMKKRHLPLGFRQRVRNYERQRWAAMRGVDECE : 470
BrCNGC20 : SEVVFNIIVLTSGLLLVTMLIGNIKVFLHATTSKKQAMHLKMRNIEWWMKKRQLPLGYRQRVRNYERQRWAAMRGVDECE : 473
BrCNGC21 : LEVVFSIVMVLSGLLLFTLLIGNIQVFLHAVMAKKRKMQIRCRDMEWWMKRRQLPSRLRQRVRRFERQRWTALGGEDELE : 498
BrCNGC22 : WEVLFTMAIIGLGLFLFALLIGNMQNFLQSLGRRNMEMTLRRRDVNNWMSHRQFPDDIRTRVKNAERLNWSSTRGVNEEL : 500
BrCNGC23 : WEVLFTMGIIGLGLLLFAFLIGNMQNFLQTLGQRNLEMTLRQRDAEQWMSHRRFPEGIRKRVREVERFHWAARRGVDEEL : 609
BrCNGC24 : -------------------------------NVWSLEMTLRRRDVEQWMSHRRLPEGIRKRVREAERFNWAATRGVNEEL : 465
BrCNGC25 : GEVFFTMGITGLGLLLFARLIGNMHNFLQALDRRRMEMMLRRRDVEQWMSHRLLPEDIRKRVREAERFNWAATRGVNEEF : 546
BrCNGC26 : GEVYFTMGIIGLGLLLFARLIGNMHNFLQALDRRRTEMMLRQRDVEQWMSHRLLPDDIRKRVREAERFNWAATRGVKEEL : 552
BrCNGC27 : GEVLFTMCIIGLGLFLFALLIGNMQNFLQSLGRRDTEMTVRRRDVEQWMSHRRFPKDIRKRVREVERLNWNATRGVNEEL : 525
BrCNGC28 : GEVFFTMGIIGLGLLLFALLIGNMQNFLQALGRRNLEMTLRRRDVEQWMSHRRLPEGIRKRVREAERFNWAATRGVNEEL : 563
BrCNGC29 : GEVYFTMGIIGLGLLLFARLIGNMHNFLQALDRRRMEMMLRRRDVEQWMSHRLLPEDIRKRVREAERFNWAATRGVNEEL : 559
AtCNGC1  : WEICFAVFISIAGLVLFSFLIGNMQTYLQSTTTRLEEMRVKRRDAEQWMSHRLLPENLRKRIRRYEQYKWQETRGVDEEN : 459
AtCNGC2  : LEVIFSIVMVLSGLLLFTLLIGNIQVFLHAVMAKKRKMQIRCRDMEWWMKRRQLPSRLRQRVRRFERQRWNALGGEDELE : 504
AtCNGC3  : GEIIFAIVICISGLVLFALLIGNMQKYLQSTTVRVEEMRVKRRDAEQWMSHRMLPDDLRKRIRKYEQYKWQETKGVEEEA : 450
AtCNGC4  : SEVVFNIIVLTSGLLLVTMLIGNIKVFLHATTSKKQAMHLKMRNIEWWMKKRHLPIGFRQRVRNYERQRWAAMRGVDECE : 469
```

```
AtCNGC5   : MEIIFSISLAISGLILFALLIGNMQTYLQSLTIRLEEMRVKRRDSEQWMHHRMLPQDLRERVRRYDQYKWLETRGVDEEY : 471
AtCNGC6   : GEVIFSITLAIAGLLLFALLIGNMQTYLQSLTIRLEEMRVKRRDSEQWMHHRMLPPELRERVRRYDQYKWLETRGVDEEN : 487
AtCNGC7   : GEVLFSIAIAIAGLLLFALLIGNMQTYLQSLTVRLEEMRIKRRDSEQWMHHRSLPQNLRERVRRYDQYKWLETRGVDEEN : 446
AtCNGC8   : GEVLFSIAIAVAGLLLFALLIGNMQTYLQSLTVRLEEMRIKRRDSEQWMHHRSLPQNLRERVRRYDQYKWLETRGVDEEN : 481
AtCNGC9   : GEVIFSIALAIAGLLLFALLIGNMQTYLQSLTIRLEEMRVKRRDSEQWMHHRMLPPELRERVRRYDQYKWLETRGVDEEN : 486
AtCNGC10  : GEIIFAISICISGLVLFALLIGNMQKYLESTTVREEEMRVRKRDAEQWMSHRMLPEDLRKRIRRYEQYRWQETRGVEEET : 441
AtCNGC11  : GEIFFAIIICVSGLLLFAVLIGNVQKYLQSSTTRVDEMEEKRRDTEKWMSYREIPEYLKERIRRFEDYKWRRTKGTEEEA : 408
AtCNGC12  : GEIFFAIIICVSGLLLFAVLIGNVQKYLQSSTTRVDEMEEKRRDTEKWMSYRVIPEYLKERIRRFEDYKWRETKGTEEEA : 408
AtCNGC13  : GEIIFAVSICISGLVLFALLIGNMQKYLESTTVREEEMRVRKRDAEQWMSHRMLPDDLRKRIRRYEQYKWQETRGVEEEN : 447
AtCNGC14  : LETMFAILVAIFGLVLFALLIGNMQTYLQSITVRLEEWRLKRRDTEEWMGHRLLPQNLRERVRRFVQYKWLATRGVDEET : 454
AtCNGC15  : GEILFAIIIATLGLVLFALLIGNMQTYLQSTTMRLEEWRIKRTDTEQWMHHRQLPPELRQAVRKYDQYKWLATRGVDEEA : 444
AtCNGC16  : SETIFSCFICVAGLVFFSHLIGNVQNYLQSTTARLDEWRVRRRDTEEWMRHRQLPDELQERVRRFVQYKWLTTRGVDEEA : 430
AtCNGC17  : GETTFAVLIAIFGLVLFAHLIGNMQTYLQSLTVRLEEWRLKKRDTEEWMRHRQLPEELRNRVRRYEQYKWLATRGVDEEV : 454
AtCNGC18  : GETLFCITICIFGLILFTLLIGNMQSSLQSMSVRVEEWRVKRRDTEEWMRHRQLPPELQERVRRFVQYKWLATRGVDEES : 422
AtCNGC19  : GEVFFTMGIIGLGLLLFARLIGNMHNFLQSLDRRRMEMMLRKRDVEQWMSHRRLPEDIRKRVREVERYTWAATRGVNEEL : 534
AtCNGC20  : GEVFFTMGIIGLGLLLFALLIGNMQNFLQALGKRNLEMTLRRRDVEQWMSHRRLPDGIRRRVREAERFNWAATRGVNEEL : 567
              e  f       g  lf  lign q  l         e   4 r1 e WM hr  P     r6       W  t g  ee

                       *        740          *        760          *        780          *        800
BrCNGC    : LLHSLPKDLRLETKRHLYLTLLNSVPWLNMMDDSWLLEALCDRVKSVFYSANSYIVKEGDPVAEMLIITKGSLKSMIGSS : 480
BrCNGC2   : HLRSLPKDLRVEAKRNLYLYSLENVPWISFIDDDWLLNEIYDRVKPVFYSQKSYILGEGDPVKEMLIVTYGELESMTESF : 516
BrCNGC3   : LLSSLPKDLRKEIKRHLCLNLLKKVPWFKAMDD-RLLDALCARLNTVLYTENSYIVREGEPVEDMVFIMRGKLISTTTYG : 526
BrCNGC4   : FLRGLPKNLRLEIERQIYQKLLKHMPWFEDMDD-RLLDSVCARLKTVIYTEDSYIVDEGEQIENMLFINRG---TVIVTE : 380
BrCNGC5   : LLRNLPKDLRRDIKRHFCLDLLKKVPLFEIMDE-QLLDAVCDKLRPVLYTENSYAIREGDPVEEMLFVMRGKLMSATTNG : 532
BrCNGC6   : LLSNLPKDLRRDIKRHLCLALVMRVPMFEQMDE-QLLDALCDRLQPVLYTEESYIVREGDPVDEMLFIMRGKLLTMTTNG : 566
BrCNGC7   : LLSNLPKDLRRDIKRHLCLALLMRVPMFEKMDE-QLLDALCDRLQPVLYTEESYIVREGDPVDEMLFIMRGKLLTMTTNG : 561
BrCNGC8   : IVQSLPKDLRRDIKRHLCLNLVRRVPLFANMDE-RLLDAICERLKPSLYTESTYIVREGDPVNEMLFIIRGRLESVTTDG : 524
BrCNGC9   : LVQNLPKDLRRDIKRHLCLALVRRVPLFESMDD-KLLDAICMRLKPCLFTESTYLVREGDPVDEMLFIIRGRLESVTTDG : 581
BrCNGC10  : IVSNLPKDLRRDIKRHLCLALVRRVPLFDNMDE-RLLDAICMRLKPCLYTEKSFLVREGDPVNEMLFIIRGRLECVTTDG : 565
BrCNGC11  : LVSNLPKDLRRDIKRHLCLALVRRVPLFENMDE-RLLDAICERLKPCLYTEKSFLVREGDPVNEMLFIIRGRLESVTTDG : 555
BrCNGC12  : LVQNLPKDLRRDIKRHLCLALVRRVPLFENMEE-RLLDAICERLKPCLYTEKSYLVREGDPVNEMLFIIRGRLESVTTDG : 544
BrCNGC13  : LLISLPLDLRRDIKRHLCFDLVRRVPLFDQMDE-RMLDAISERLKPALFTEGTFLVREGDPVNEMLFIIRGHLDSYTTNG : 530
BrCNGC14  : ILQSLPTDLRREIQRHLCLALVRRVPFFSQMDD-QLLDAICGCLVSSLSTAGTYIFREGDPVDEMLFVIRGQIESSTTNG : 501
BrCNGC15  : IIRALPLDLRRQIQRHLCLALVRRVPFFAQMDD-QLIDAICERLVPSLNTKDTYVTREGDPVNEMLFIIRGQMESSTTDG : 510
BrCNGC16  : LLQSLPTDLRRDIQRHLCLDLVRRVPFFSQMDD-QLLDAICERLVSSLCTEGTYLVREGDLITEMLFIIRGRLESSTTNG : 537
BrCNGC17  : ILHSLPADLRRDIQRHLCLDLVRRVPLFAQMDD-QLLDAICERLVSSLSTQGNYIVREGDPVTEMLFIIRGKLESSTTNG : 538
BrCNGC18  : ILHSLPADLRRDIQRHLCLDLVRRVPLFAQMDD-QLLDAICERLVSSLSTQGNYIVREGDPVTEMLFIIRGKLDSSTTNG : 534
BrCNGC19  : MVQNLPEGLRRDIKYHLCLDLVRQVPLFQHMDD-LVLENICDRVKSLIFTKGETIQKEGDAVQRMLFVVRGHLQSSQL-- : 547
BrCNGC20  : MVQNLPEGLRRDIKYHLCLDLVRQVPLFQHMDD-LVLENICDRVKSLIFTKGETIQKEGDAVQRMLFVVRGHLQSSQL-- : 550
BrCNGC21  : LIQDLPPGLRRDIKRYLCVDLINNVPLFRGMDD-LILDNICDRAKPRVYSKDEKIIREGDPVQRMIFIMRGRVKRNQS-- : 575
BrCNGC22  : LFENMSDDLQRDIRRHLFA-FLKKVRIFSKMDE-PILDAIRERLKHKTYLNGSMVLHSGDLVKKMVIIVRGQMESTREDG : 578
BrCNGC23  : LFNNMPDDLQRDIRRHLFK-FLNKVRIFTLMDE-SILNAIRERLKHITYISSSVVFSAGDVIEKIVFIVRGEMESIGKDG : 687
BrCNGC24  : LFENMPDDLQGDIRRHLFI-FLKKVRIFSLMDE-SILDSIRERLRQRTYISGSTVIHRWGPVEKIVFVVRGEMESTGEDG : 543
BrCNGC25  : LFENMPEDLLRDIKRHLFK-FLKKVRIFSLMDE-SILDSIRERLKQRTYISSSTVIHRRGLVEKMVFIVRGEMESIGQDG : 624
BrCNGC26  : IFENMPDDIQRDIKRHLFK-FLKKVWIFSLMDE-LILDAIRERLKQRRYIRSSTVIRRKGLVEKMVFIVRGERESIGEDG : 630
BrCNGC27  : LFENMPDDLQRDIRRHLFA-FLKKVRIFSEIDE-SILDAMRARLKQRTYLKNNKVIHRGGVVKKMVFILRGKMESIGEDG : 603
BrCNGC28  : LFENMPDDLQRDIRRHLFI-FLKKVRIFSLMDE-SILDAIRERLKQRTYISSSTVLHRGGLVEKMVFIVRGEMESIGEDG : 641
BrCNGC29  : LFENMPDDLQRDIKRHLFK-FLKKVRIFSLMDE-SILDAIRERLKQRTYIRSSTVIHRKGLVEKMVFIVRGEMESIGEDG : 637
AtCNGC1   : LLSNLPKDLRRDIKRHLCLALLMRVPMFEKMDE-QLLDALCDRLQPVLYTEESYIVREGDPVDEMLFIMRGKLLTITTNG : 538
AtCNGC2   : LIHDLPPGLRRDIKRYLCFDLINKVPLFRGMDD-LILDNICDRAKPRVFSKDEKIIREGDPVQRMIFIMRGRVKRIQS-- : 581
AtCNGC3   : LLSSLPKDLRKDIKRHLCLKLLKKVPWFQAMDD-RLLDALCARLKTVLYTEKSYIVREGEPVEDMLFIMRGNLISTTTYG : 529
AtCNGC4   : MVQNLPEGLRRDIKYHLCLDLVRQVPLFQHMDD-LVLENICDRVKSLIFTKGETIQKEGDAVQRMLFVVRGHLQSSQL-- : 546
AtCNGC5   : LVQNLPKDLRRDIKRHLCLALVRRVPLFKSMDD-KLLDAICMRLKPCLFTESTYLVREGDPVDEMLFIIRGRLESVTTDG : 550
AtCNGC6   : LVQNLPKDLRRDIKRHLCLALVRRVPLFENMDE-RLLDAICERLKPCLFTEKSYLVREGDPVNEMLFIIRGRLESVTTDG : 566
AtCNGC7   : IVQSLPKDLRRDIKRHLCLNLVRRVPLFANMDE-RLLDAICERLKPSLFTESTYIVREGDPVNEMMFIIRGRLESVTTDG : 525
AtCNGC8   : IVQSLPKDLRRDIKRHLCLNLVRRVPLFANMDE-RLLDAICERLKPSLYTESTYIVREGDPVNEMLFIIRGRLESVTTDG : 560
AtCNGC9   : LVQNLPKDLRRDIKRHLCLALVRRVPLFENMDE-RLLDAICERLKPCLYTESSYLVREGDPVNEMLFIIRGRLESVTTDG : 565
AtCNGC10  : LLRNLPKDLRRDIKRHLCLDLLKKVPLFEIMDE-QLLDAVCDRLRPVLYTENSYVIREGDPVGEMLFVMRGRLVSATTNG : 520
AtCNGC11  : LLRSLPKDLRLETKRYLFLKLLKKVPLLQAMDD-QLLDALCARLKTVHYTEKSYIVREGEPVEDMLFIMRGNLISTTTYG : 487
AtCNGC12  : LLRSLPKDLRLETKRYLYLDMLKRVPWLNIMDDGWLLEAVCDRVKSVFYLANSFIVREGHPVEEMLIVTRGKLKSTTGSH : 488
AtCNGC13  : LLRNLPKDLRRDIKRHFCLDLLKKVPLFEIMDE-QLLDAVCDKLKPVLYTENSYAIREGDPVEEMLFVMRGKLMSATTNG : 526
AtCNGC14  : IIHSLPADLRRDIQRHLCLDLVRRVPLFAQMDD-QLLDAICERLASSLSTQGNYIVREGDPVTEMLFIIRGKLESSTTNG : 533
AtCNGC15  : LLISLPLDLRRDIKRHLCFDLVRRVPLFDQMDE-RMLDAICERLKPALCTEGTFLVREGDPVNEMLFIIRGHLDSYTTNG : 523
AtCNGC16  : IIRALPLDLRRQIQRHLCLALVRRVPFFAQMDD-QLLDAICERLVPSLNTKDTYVIREGDPVNEMLFIIRGQMESSTTDG : 509
AtCNGC17  : LLQSLPTDLRRDIQRHLCLDLVRRVPFFSQMDD-QLLDAICERLVSSLCTEGTYLVREGDLISEMLFIIRGRLESSTTNG : 533
```

```
AtCNGC18 : ILHSLPTDLRREIQRHLCLSLVRRVPFFSQMDD-QLLDAICGCLVSSLSTAGTYIFREGDPVNEMLFVIRGQIESSTTNG : 501
AtCNGC19 : LFENMPDDLQRDIRRHLFK-FLKKVRIFSLMDE-SVLDSIRERLKQRTYIRSSTVIHHRGLVEKMVFIVRGEMESIGEDG : 612
AtCNGC20 : LFENMPDDLQRDIRRHLFK-FLKKVRIFSLMDE-PILDAIRERLKQRTYIGSSTVLHRCGLVEKMVFIVRGEMESIGEDG : 645
                 6p  6r di rh     6  6p f  6d   66 a6                eg  6  66f6  G        g

                      *        820         *        840         *        860         *        880
BrCNGC    : DITGYYD-SSYLQAGDIC-GDLLFWVLDPHSSSSLPTS----------DRSVLTLTDVEGEILLHDDLKFVASHFNRFHS : 548
BrCNGC2   : ETSSYSDIQIRLMKGDVW-EDLLFWALDPHTSPSLPIS----------NGTVTTLTYVEGLTLSADDLKCHS-------- : 577
BrCNGC3   : GQTGFFN-IAHLEAGDFCG-DLLTWALDPNTS-HLPIS----------TRTVQAETEVEGEVLSAEDLKFFSTQYRRLHS : 593
BrCNGC4   : KKTTFFR-SVGEFCG----EELLTWALLDPHSSCVPIS----------SMIIKARTEVEGEVLSADDLKFVAAQYCRLHR : 445
BrCNGC5   : GRTGFFN-AVYLKASDFCGEDLLTWALDPQSSSHFPIS----------PRTVQALTEVEAFALAAEDLKLVASQFRRLHS : 601
BrCNGC6   : GRTGFFN-SEHLGAGDFCGEELLTWALDPHTSTNLPIS----------TRTVQALVEVEAFALKADNLKFVASQFRRLHS : 635
BrCNGC7   : GRTGFFN-SEHLGAGDFCGEELLTWALDPHTSSNLPIS----------TRTVRALVEVEAFALKADDLKFVASQFRRLHS : 630
BrCNGC8   : GRSGFFN-RGLLKEGDFCGEELLTWALDPKAGSNLPSS----------TRTVKALTEVEAFALEAEELKFVASQFRRLHS : 593
BrCNGC9   : GRSGFFN-YSLLKEGEFCGEELLTWALDPKSGVNLPSS----------TRTVKALTEVEAFALASEELKFVASQFRRLHS : 650
BrCNGC10  : GRSGFYK-RSLLKEGDFCGDELLTWALDPKSGSNLPSS----------TRTVKALTEVEAFALIADELKFVASQFRRLHS : 634
BrCNGC11  : GRSGFYN-RSLLKEGDFCGDELLTWALDPKSGSNLPSS----------TRTVKALTEVEAFALIADELKFVASQFRRLHS : 624
BrCNGC12  : GRSGFFN-RSLLKEGDFCGEELLTWALDPKSGSNLPSS----------TRTAKALTEVEAFALIADELKFVASQFRRLHS : 613
BrCNGC13  : GRTGFFN-SCLIGPGDFCGEELLTWALDPRPVVILPSS----------TRTVKAIYEVEAFALRADDLKFVATQFRRLHS : 599
BrCNGC14  : GRSGFFN-STTLRPGDFCGEELLTWALMPNSTLNFPSS----------TRSVRALSEVEAFALSAEDLKFVAHQFKRLQS : 570
BrCNGC15  : GRSGFFN-SITLRPGDFCGEELLTWALMPNINQNLPLS----------TRTVRTLSEVEAFALRAEDLKFVANQFRRLHS : 579
BrCNGC16  : GRTGFFN-SIILRPGDFCGEELLSWALLPKSTLNLPSS----------TRTVRALVEVEAFALRAEDLKFVANQFRRLHS : 606
BrCNGC17  : GRTGFFN-SITLKPGEFCGEELLAWALLPKSKVNLPSS----------TRTVRALEEVEAFALQAEDLKFVANQFRRLHS : 607
BrCNGC18  : GRTGFFN-SITLKPGEFCGEELLAWALLPKSKVNLPSS----------TRTVRALEEVEAFALQAEDLKFVANQFRRLHS : 603
BrCNGC19  : LRDGVKS-CCMLGPGNFSGDELLSWCLRRPFVERLPPS----------SSTLVTLETTEAFGLDAQDVKYVTQHFRYTFV : 616
BrCNGC20  : LRDGVRS-CCMLGPGNFSGDELLSWCLRRPFVERLPPS----------TSTLVTLETTEAFGLDAEDVKYVTQHFRYTFV : 619
BrCNGC21  : LSKGVVA-TSTLEPGGYLGDELLSWCLRRPFIDRLPPS----------SATFVCLDNIEAFSLGSEDLRYITDHFRYKFA : 644
BrCNGC22  : SCTRLSK-------RDVYGEELLTWY-ERSSLNPDGTRIRMPSKGLLSNRDVKCVTNVEAFSLSVADLDDVMSLFPNLEI : 650
BrCNGC23  : SVISLSE-------GDVFGEELLSWCLERAASNTDGTRIWIKRKGLLSYRSVRCVTNVDAFSLSVADLDASR------DC : 754
BrCNGC24  : SVLPLSE-------GDVCGEELLTWCLERSAVNLDG---RMLSKGLLSSRNVKCVTNVEAFSLSAADLEDVTSLFSRFLR : 613
BrCNGC25  : SVLLLSE-------GDVCGEELLTWCLERSSVNPDGRSIKLPLKGLVSNRSVRCVTNVEAFSLSVADLEDVTSLFSRFLR : 697
BrCNGC26  : SVLPLSE-------GDVCGEELLTWCLERSSVDPDGTRIKMPTKGLVSNRSVRCVTNVEAFSLSVADLEDVTSLFSRFLR : 703
BrCNGC27  : YRTLLSE-------GDVCGEELLTWCQERSSVNPDGTIIRMPSKGLLSSRDVWCVTNVEAFSLSVADLEDITSLFPNLEI : 676
BrCNGC28  : SVLPLSE-------GDVCGEELLTWCLERSSVNPDGTRIRIPSKGLLSYRNVKCVTNVEAFSLSVADLEDVTSLFSRFLR : 714
BrCNGC29  : SVLPLSE-------GDVCGEELLTWCLERSSVNPDGTRIKMPTKGLVSNRNVRCVTNVEAFSLSVADLEDVTSLFSRFLR : 710
AtCNGC1   : GRTGFLN-SEYLGAGDFCGEELLTWALDPHSSSNLPIS----------TRTVRALMEVEAFALKADDLKFVASQFRRLHS : 607
AtCNGC2   : LSKGVLA-TSTLEPGGYLGDELLSWCLRRPFLDRLPPS----------SATFVCLENIEAFSLGSEDLRYITDHFRYKFA : 650
AtCNGC3   : GRTGFFN-SVDLVAGDFCG-DLLTWALDPLSS-QFPIS----------SRTVQALTEVEGELLSADDLKFVATQYRRLHS : 596
AtCNGC4   : LRDGVKS-CCMLGPGNFSGDELLSWCLRRPFVERLPPS----------SSTLVTLETTEAFGLDAEDVKYVTQHFRYTFV : 615
AtCNGC5   : GRSGFFN-RSLLKEGEFCGEELLTWALDPKSGVNLPSS----------TRTVKALTEVEAFALTSEELKFVASQFRRLHS : 619
AtCNGC6   : GRSGFYN-RSLLKEGDFCGDELLTWALDPKSGSNLPSS----------TRTVKALTEVEAFALIADELKFVASQFRRLHS : 635
AtCNGC7   : GRSGFFN-RGLLKEGDFCGEELLTWALDPKAGSNLPSS----------TRTVKALTEVEAFALEAEELKFVASQFRRLHS : 594
AtCNGC8   : GRSGFFN-RGLLKEGDFCGEELLTWALDPKAGSNLPSS----------TRTVKALTEVEAFALEAEELKFVASQFRRLHS : 629
AtCNGC9   : GRSGFFN-RSLLKEGDFCGEELLTWALDPKSGSNLPSS----------TRTAKALTEVEAFALIADELKFVASQFRRLHS : 634
AtCNGC10  : GRSGFFN-AVNLKASDFCGEDLLPWALDPQSSSHFPIS----------TRTVQALTEVEAFALTAEDLKSVASQFRRLHS : 589
AtCNGC11  : GRTGFFN-SVDLIAGDSC-GDLLTWALYSL-SSQFPIS----------SRTVQALTEVEGEVISADDLKFVATQYRRLHS : 554
AtCNGC12  : EMGVRNN-CCDLQDGDIC-GELLFN------GSRLPTS----------TRTVMTLTEVEGEILLPDDIKFIASHLNVFQR : 550
AtCNGC13  : GRTGFFN-AVYLKPSDFCGEDLLTWALDPQSSSHFPIS----------TRTVQALTEVEAFALAADDLKLVASQFRRLHS : 595
AtCNGC14  : GRTGFFN-SITLRPGDFCGEELLAWALLPKSTVNLPSS----------TRTVRALEEVEAFALQAGDLKFVANQFRRLHS : 602
AtCNGC15  : GRTGFFN-SCLIGPGDFCGEELLTWALDPRPVVILPSS----------TRTVKAICEVEAFALKAEDLQFVASQFRRLHT : 592
AtCNGC16  : GRSGFFN-SITLRPGDFCGEELLTWALVPNINHNLPLS----------TRTVRTLSEVEAFALRAEDLKFVANQFRRLHS : 578
AtCNGC17  : GRTGFFN-SIILRPGDFCGEELLSWALLPKSTLNLPSS----------TRTVRALVEVEAFALRAEDLKFVANQFRRLHS : 602
AtCNGC18  : GRSGFFN-STTLRPGDFCGEELLTWALMPNSTLNLPSS----------TRSVRALSEVEAFALSAEDLKFVAHQFKRLQS : 570
AtCNGC19  : SVLPLSE-------GDVCGEELLTWCL--SSINPDGTRIKMPPKGLVSNRNVRCVTNVEAFSLSVADLEDVTSLFSRFLR : 683
AtCNGC20  : SVLPLYE-------GDVCGEELLTWCLERSSVNPDGTRIRMPSKGLLSSRNVRCVTNVEAFSLSVADLEDVTSLFSRFLR : 718
                              g  cg eLL w l         p s          r        eaf 6     6        r

                      *        900         *        920         *        940         *        960
BrCNGC    : SRLRHM----F--YSAHWRLWAACFIQAAWREHYKRKLSRILHAKRDYNH------IP---------------------- : 594
BrCNGC2   : ----------------KSWRSWAAFYIQAAWKAHCRRKASKILPAIKDEQQ------IL---------------------- : 614
BrCNGC3   : KQLRHTC------YSVQWQTWAACFIQAAWKRYCRRKLSRVLREEERLQNTLQTTDD----------------------- : 645
BrCNGC4   : KKARHISRQEFQLHSRKWRNWAACFIQAAWTEYCRRKLSKDLGEEEAKLHSTIQKDDS---------------------- : 503
BrCNGC5   : KQLQHTFR----FYSVQWRTWGASFIQAAWRRHCRRRLARSLTEEEDRFRIAVAKRER---------------------- : 655
BrCNGC6   : KQLRHTFR----FYSQQWRTWAACFIQAAWRRHVKKRLEESLREEENRLQDALAKQAC---------------------- : 689
BrCNGC7   : KQLRHTFR----FYSQQWRTWAACFIQAAWRRHVKKKMEESLKEEENRLQDALAKEAC---------------------- : 684
```

```
BrCNGC8  : RQVQQTFR----FYSQQWRTWASSFIQAAWRRHSRRKNAELRRIEEEEDEMGYED-EYDDDDAE-------EEDERTPVF : 661
BrCNGC9  : RQVQHTFR----FYSHQWRTWAACFIQAAWRRYCKRKKMEEAEAEAVPMS--TTGSS----------------------- : 701
BrCNGC10 : RQVQHTFR----FYSQQWRTWAACFIQAAWRRYTKRKKLEQLRKEEEEEEEESAARLIAGGS------------------ : 692
BrCNGC11 : RQVQHTFR----FYSQQWRTWAACFIQAAWRRYTKRKKLEELRKEEEMEEESSTARLIAGGSS----------------- : 683
BrCNGC12 : RQVQHTFR----FYSQQWRTWAAIFIQAAWRRYVKKKKLEQLKKEEEEEGEGHVA------------------------- : 664
BrCNGC13 : KQLKHKFR----FYSHQWRTWAACFIQAAWRRHKKRKYATELRVKEEFQCMFETASM----------------------- : 652
BrCNGC14 : KKLQHAFR----YYSHQWRAWGACFVQSAWRRYKRRKLAKELSLHESSGYYYRDETGYNEEGDE--ENYYGSDDDDFEGE : 644
BrCNGC15 : KKLQHAFR----YYSHQWRAWGTGFIQAAWRRYMKRKLAMELARQEEGDDYYYDDDD---------DDQYGGEDMPESSN : 646
BrCNGC16 : KKLQHTFR----FYSPHGRTWAACFIQAAWRRYKRRAMENHLTAVESKQSDEDDEEEEVVVR----KVVEEEEEGVGSSP : 678
BrCNGC17 : KKLQHTFR----YYSHQWRTWAACFVQVAWRRYKRRMLAKSLSLAESYSSYEEEEALAAAAA----EEIMSQQEERQSST : 679
BrCNGC18 : KKLQHTFR----YYSHQWRTWAACFVQVAWRRYKRRMVAKSLSLAESFSSYEEEEAVAVAA-----EEIMSQQGERQSSN : 674
BrCNGC19 : NEKVKRSAR---YYSPGWRTWAAVAVQLAWRRYKHRLTLTSLSFIRPRRP------------------------------ : 663
BrCNGC20 : NEKVKRSAR---YYSPGWRTWAAVAVQLAWRRYKHRLTLTSLSFIRPRRP------------------------------ : 666
BrCNGC21 : NERLKRTAR---YYSSNWRTWAAVNIQMSWRRYRKRTCG-GVGGSMSPVS------------------------------ : 690
BrCNGC22 : SKES---------------------------------------------------------------------------- : 654
BrCNGC23 : SRDS---------------------------------------------------------------------------- : 758
BrCNGC24 : SHRVQGAIR---YESPYCRLRAATQIQVAWRRYRKRRLQKLSTAQKKQYSSELIQKDMAKT------------------- : 670
BrCNGC25 : SHRVLGAIR---YESPYWRLRAATQIQVAWRRYRRRRLHRLYTAQSTSRR------------------------------ : 743
BrCNGC26 : SNRVQGAIR---YESPYWRLRAAMQIQVAWRRYRRKRLERKKQNGD--IDE----------------------------- : 748
BrCNGC27 : PKEP---------------------------------------------------------------------------- : 680
BrCNGC28 : NPRVQGAIR---YESPYWRLRAARQIQVAWRRYRRRRLQRLYTAQS-SYSL----------------------------- : 760
BrCNGC29 : SHRVQGAIR---YESPYWRLRAAMQIQVAWRRYRKKRLERLLLQDNRVMKEMSDVRIGYRLSWSGVVEGSGKQLDTRGSS- : 786
AtCNGC1  : KQLRHTFR----YYSQQWKTWAACFIQAAWRRYIKKKLEESLKEEENRLQDALAKEAC---------------------- : 661
AtCNGC2  : NERLKRTAR---YYSSNWRTWAAVNIQMAWRRRRKRTRGENIGGSMSPVS------------------------------ : 697
AtCNGC3  : KQLRHMFR----FYSVQWQTWAACFIQAAWKRHCRRKLSKALREEEGKLHNTLQN-DD---------------------- : 649
AtCNGC4  : NEKVKRSAR---YYSPGWRTWAAVAVQLAWRRYKHRLTLTSLSFIRPRRP------------------------------ : 662
AtCNGC5  : RQVQHTFR----FYSHQWRTWAACFIQAAWRRYCKRKKMEEAEAEAAAVSSSTAGPS----------------------- : 672
AtCNGC6  : RQVQHTFR----FYSQQWRTWAACFMQAAWRRYIKRKKLEQLRKEEE-EEEAAAASVIAGGS------------------ : 692
AtCNGC7  : RQVQQTFR----FYSQQWRTWASCFIQAAWRRYSRRKNAELRRIEEKEEELGYED-EYDD-----------ESDKRPMVI : 658
AtCNGC8  : RQVQQTFR----FYSQQWRTWAACFIQAAWRRHLRRKIAELRRKEEEEEEMDYEDDEYYD-------------DNMGGMV : 692
AtCNGC9  : RQVQHTFR----FYSQQWRTWAAIFIQAAWRRYVKKKKLEQLRKEEEE-GEGSVT------------------------- : 684
AtCNGC10 : KQLQHTFR----FYSVQWRTWSVSFIQAAWRRYCRRKLAKSLRDEEDRLREALASQDKEHNAA----------------- : 648
AtCNGC11 : KQLQHM----FRFYSLQWQTWAACFIQAAWKRHCRRKLSKALREE----------------------------------- : 595
AtCNGC12 : QKLQRT----FRLYSQQWRSWAAFFIQAAWRKHCKRKLSKTRDNEN----------IP---------------------- : 594
AtCNGC13 : KQLQHTFR----FYSVQWRTWGASFIQAAWRRHCRRKLARSLTEEEDRFRNAITKRER---------------------- : 649
AtCNGC14 : KKLQHTFR----YYSHQWRTWAACFVQVAWRRYKRKKLAKSLSLAESFSSYDEEEAVAVAA-----TEEMSHEGEAQSGA : 673
AtCNGC15 : KQLRHKFR----FYSHQWRTWAACFIQAAWRRHRKRKYKTELRAKEEFHYRFEAATAR---------------------- : 646
AtCNGC16 : KKLQHAFR----YYSHQWRAWGTCFIQAAWRRYMKRKLAMELARQEEEDDYFYDDDG---------DYQF-EEDMPESNN : 644
AtCNGC17 : KKLQHTFR----FYSHHWRTWAACFIQAAWRRYKRRVMENNLTAIESMENEEGEVGEELVV--------VEEEECVEESP : 670
AtCNGC18 : KKLQHAFR----YYSHQWRAWGACFVQSAWRRYKRRKLAKELSLHESSGYYYPDETGYNEEDEETREYYYGSDE---EGG : 643
AtCNGC19 : SHRVQGAIR---YESPYWRLRAAMQIQVAWRRYRKRQLQRLNTAHSNSNR------------------------------ : 729
AtCNGC20 : SHRVQGAIR---YDSPYWRLRAARQIQVAWRRYRRRRLHRLCTPQS-SYSL----------------------------- : 764
                         s  w  waa   q aw

                   *       980         *      1000          *      1020          *      1040
BrCNGC   : ------QGTQLNIGAALYVSRFVSKALRNRQKNAANCSI-----SPHMLPP--IPHKPADPEFSKN-------------- : 647
BrCNGC2  : ------QDTQRNLGATLYASRFVSKALRNRHVDSAECSS-----FPEMLP-----DKPADPEFSKKEA------------ : 666
BrCNGC3  : -----SGGNKLNLGAAIYASRFASHALRNVRANAAARSSM----LPHMLSL--LPQKPADPEFPMDET------------ : 702
BrCNGC4  : ------AGNTHNLGGTAFASRLASTVLRNLRVNASRKDR-----VRQISLP----EKPVDPKFPMDEI------------ : 556
BrCNGC5  : -----RAASSPSLVATLYASRFASNALRNLRQHNN-----------NTLPL--LPPKPSEPDFSVDDD------------ : 705
BrCNGC6  : -------GSSPSFGATMYASRFAANILRTIRRSGSVRKPR----MLERMPPMLLLQKPAEPDFNSDDYMRIVPIYKNRSL : 758
BrCNGC7  : -------GSSPSLGATMYASRFAANILRTIRRSGSVRKPR----MPER----MLLQKPAEPDFNSDDYCI---------- : 739
BrCNGC8  : TRTES----SSRLRSTIFASRFAANALKG-HRLRSTESS----------KRLLNLQKPPEPDFDAE-------------- : 712
BrCNGC9  : ----------SSMGAAFLVTKFAASALRTIHRNRNTRIR-----------ELVKLQKPPEPDFTAEDAD----------- : 749
BrCNGC10 : ---------PYSIRATFLASKFAANALRSVHKNRIRKSNL----APPSTKELVKFQKPPEPDFSADC------------- : 746
BrCNGC11 : ---------PFSIRATFLASRFAANALRGVRKNRTAKLLA----LSQPTKELLKVQKPPEPDFSADC------------- : 737
BrCNGC12 : -----------SIRATFLASKFAANALRKVHQNRIAAK---------STKELVIFQKPSEPDFSADDP------------ : 712
BrCNGC13 : -----------------VRLNSGKFTRSGSDSGMVSS-----------------IQKPVEPDFSSE-------------- : 684
BrCNGC14 : RLSVDNTNNSQNLGATMLASKFAANTRRGTNQKASSSTSAGKKDGSSNSLKMPQLFKPDEPDFSMDKEDV---------- : 714
BrCNGC15 : N-VDDNSSNSQNLSATILASKFAANTKRGVLGNQRGS---SRIDPDDPTLKMPKMFKPEDPGFF---------------- : 706
BrCNGC16 : KT-------KMNIGVMVLASRFAANTRRGVAAQRVKD------------VEMPRFKKPEEPDFSAEPDD----------- : 728
BrCNGC17 : PSRHHTSIGKPHFAATVLASRFAKNTRR--ASRKMKD------------VDVPMLPKPEEPDFSVDAD------------ : 733
BrCNGC18 : PSRHSTSIGKPHFAATILASRFAKNTRK--TAHKLKD------------VEVPMLPKPDEPDFSVDGD------------ : 728
BrCNGC19 : ------------------LSRCASLGEDKLRLYTAILTS----------------PKPNPDDFDDY-------------- : 695
BrCNGC20 : ------------------LSRCASLGEDKLRLYTAILTS----------------PKPNPDDFDDY-------------- : 698
```

```
BrCNGC21 : -------------------EHSVEGNSERRLLQYAAMFMS----------------IRPH-DHLE---------------- : 719
BrCNGC22 : -------------------------------------------------------------------------------- :   -
BrCNGC23 : -------------------------------------------------------------------------------- :   -
BrCNGC24 : -------------------------------------------------------------------------------- :   -
BrCNGC25 : -------------------------------------------------------------------------------- :   -
BrCNGC26 : -------------------------------------------------------------------------------- :   -
BrCNGC27 : -------------------------------------------------------------------------------- :   -
BrCNGC28 : -------------------------------------------------------------------------------- :   -
BrCNGC29 : -------------------------------------------------------------------------------- :   -
AtCNGC1  : -------GSSPSLGATIYASRFAANILRTIRRSGSVRKPR----MPERMP-PMLLQKPAEPDFNSDD------------- : 716
AtCNGC2  : ------------------ENSIEGNSERRLLQYAAMFMS----------------IRPH-DHLE---------------- : 726
AtCNGC3  : -----SGGNKLNLGAA---------------------------------------------------------------- : 660
AtCNGC4  : ------------------LSRCASLGEDKLRLYAAILTS----------------PKPNPDDFDDY-------------- : 694
AtCNGC5  : ----------YSIGAAFLATKFAANALRTIHRNRNTKIR-----------DLVKLQKPPEPDFTAD-------------- : 717
AtCNGC6  : ---------PYSIRATFLASKFAANALRSVHKNRTAKSTL----LLSSTKELVKFQKPPEPDFSAEDH------------ : 747
AtCNGC7  : TRSES----SSRLRSTIFASRFAANALKG-HRLRSSESS----------KTLINLQKPPEPDFDAE-------------- : 709
AtCNGC8  : TRSDSSVGSSSTLRSTVFASRFAANALKG-HKLRVTESS----------KSLMNLTKPSEPDFEALDTDDLN-------- : 753
AtCNGC9  : -----------SIRATFLASKFAANALRKVHKNRIEAK---------STIELVKYQKPSEPDFSADDTS----------- : 733
AtCNGC10 : -----TVSSSLSLGGALYASRFASNALHNLRHNISNLP------PRYTLPL--LPQKPTEPDFTANHTTDP--------- : 706
AtCNGC11 : -----------------------EGKLHNTLQNDDSGGN-----KLNLGAA--IYA------------------------ : 621
AtCNGC12 : ------QGTQLNLASTLYVSRFVSKALQNRRKDTADCSS-----SPDMSPP--VPHKPADLEFAKAEA------------ : 649
AtCNGC13 : -----NAASSSSLVATLYASRFASNALRNLRTNN--------------LPL--LPPKPSEPDFSLRNPIYASRFASHALR : 708
AtCNGC14 : KARHHTSNVKPHFAATILASRFAKNTRR--TAHKLKD------------VEIPMLPKPDEPDFSVDD------------- : 726
AtCNGC15 : -----------------LAVNGGKYTRSGSDSGMMSS-----------------IQKPVEPDFSSE-------------- : 678
AtCNGC16 : NNGDENSSNNQNLSATILASKFAANTKRGVLGNQRGS---TRIDPDHPTLKMPKMFKPEDPGFF---------------- : 705
AtCNGC17 : RT-------KMNLGVMVLASRFAANTRRGVAAQRVKD------------VELPRFKKPEEPDFSAEHDD----------- : 720
AtCNGC18 : --SMDNTN----LGATILASKFAANTRRGTNQKASSS-STGKKDGSSTSLKMPQLFKPDEPDFSIDKEDV---------- : 706
AtCNGC19 : -------------------------------------------------------------------------------- :   -
AtCNGC20 : -------------------------------------------------------------------------------- :   -
```

```
                   *      1060        *
BrCNGC   : ---------------------------------- :   -
BrCNGC2  : ---------------------------------- :   -
BrCNGC3  : ---------------------------------- :   -
BrCNGC4  : ---------------------------------- :   -
BrCNGC5  : ---------------------------------- :   -
BrCNGC6  : ---------------------------------- :   -
BrCNGC7  : ---------------------------------- :   -
BrCNGC8  : ---------------------------------- :   -
BrCNGC9  : ---------------------------------- :   -
BrCNGC10 : ---------------------------------- :   -
BrCNGC11 : ---------------------------------- :   -
BrCNGC12 : ---------------------------------- :   -
BrCNGC13 : ---------------------------------- :   -
BrCNGC14 : ---------------------------------- :   -
BrCNGC15 : ---------------------------------- :   -
BrCNGC16 : ---------------------------------- :   -
BrCNGC17 : ---------------------------------- :   -
BrCNGC18 : ---------------------------------- :   -
BrCNGC19 : ---------------------------------- :   -
BrCNGC20 : ---------------------------------- :   -
BrCNGC21 : ---------------------------------- :   -
BrCNGC22 : ---------------------------------- :   -
BrCNGC23 : ---------------------------------- :   -
BrCNGC24 : ---------------------------------- :   -
BrCNGC25 : ---------------------------------- :   -
BrCNGC26 : ---------------------------------- :   -
BrCNGC27 : ---------------------------------- :   -
BrCNGC28 : ---------------------------------- :   -
BrCNGC29 : ---------------------------------- :   -
AtCNGC1  : ---------------------------------- :   -
AtCNGC2  : ---------------------------------- :   -
AtCNGC3  : ---------------------------------- :   -
AtCNGC4  : ---------------------------------- :   -
```

```
AtCNGC5  : ---------------------------------- :   -
AtCNGC6  : ---------------------------------- :   -
AtCNGC7  : ---------------------------------- :   -
AtCNGC8  : ---------------------------------- :   -
AtCNGC9  : ---------------------------------- :   -
AtCNGC10 : ---------------------------------- :   -
AtCNGC11 : ---------------------------------- :   -
AtCNGC12 : ---------------------------------- :   -
AtCNGC13 : NLRANAAARNSRFPHMLTLLPQKPADPEFPMDET : 742
AtCNGC14 : ---------------------------------- :   -
AtCNGC15 : ---------------------------------- :   -
AtCNGC16 : ---------------------------------- :   -
AtCNGC17 : ---------------------------------- :   -
AtCNGC18 : ---------------------------------- :   -
AtCNGC19 : ---------------------------------- :   -
AtCNGC20 : ---------------------------------- :   -
```

Figure S3. Multiple sequence alignment of *CNGC*-encoded proteins of *B. oleracea* and *B. rapa* L. Multiple sequence alignment was performed by clustal X2 and viewed by GeneDoc software package.

```
                    *        20         *        40         *
BrCNGC1  : -------------------------------------------------- :   -
BoCNGC3  : -------------------------------------------------- :   -
BrCNGC2  : -------------------------------------------------- :   -
BrCNGC4  : -------------------------------------------------- :   -
BrCNGC3  : --------------------------------------------MEMMNL :   6
BoCNGC2  : --------------------------------------------MEMMNL :   6
BrCNGC5  : -------------------------------------------MGFGRDS :   7
BoCNGC1  : -------------------------------------------MGFGRDN :   7
BrCNGC6  : ------MKFRLTSLTLSSCGSRVLHSSHKEGYTTISCASTNSPTMNFRPD :  44
BrCNGC7  : --------------------------------------------MNFRQE :   6
BrCNGC8  : -------------------------------------------------- :   -
BoCNGC8  : ----------------------MYKSQYISGQREKFVRLDDLDSSASHAT :  28
BrCNGC9  : MTFASLPYHFCRASDRGLSRSSVIELAIMAGKPQTFVSVDDLDFKLPSSS :  50
BoCNGC7  : ----------------------------MAGKPQTFVSVDDLDFKLPSSS :  22
BrCNGC10 : ---------------MFDCGTNGVKSQVISGHREKFIRLESMDSRYSQSS :  35
BoCNGC5  : ---------------MFDCGTNGVKSQVISGHREKFIRLESMDSRYSQSS :  35
BrCNGC11 : ---------------------MESKSQVISGHREKFIRLDSMDPR----S :  25
BoCNGC4  : ---------------------MESKSQVISGHREKFIRLDSMDPR----S :  25
BrCNGC12 : -----------------------------------------MDSRYSQ-G :   8
BoCNGC6  : ---------------MFDCVKKSVKSQVISGQREKFVRLDSMDSRYSQ-G :  34
BrCNGC13 : -----------------------------------------------MGY :   3
BoCNGC9  : -----------------------------------------------MGY :   3
BrCNGC14 : -------------------------------------------------M :   1
BoCNGC13 : -------------------------------------------------M :   1
BrCNGC15 : ----------------------------------------------MSNL :   4
BoCNGC14 : ----------------------------------------------MSNL :   4
BrCNGC16 : -----------------------------------------------MEL :   3
BoCNGC10 : -----------------------------------------------MEL :   3
BrCNGC17 : -----------------------------------------------MEF :   3
BoCNGC12 : -----------------------------------------------MEF :   3
```

```
BrCNGC18 : -----------------------------------------------MEF :   3
BoCNGC11 : -----------------------------------------------MEF :   3
BrCNGC22 : --------------------------MASSNG--YDDVPML-PVSCTSSS :  21
BoCNGC19 : --------------------------MASSNG--YDDVPML-PVSCTSSS :  21
BrCNGC27 : --------------------------MASPME--NDDVPML-PASDTSSS :  21
BoCNGC18 : --------------------------MASPME--NDDVPML-PASDTSSS :  21
BrCNGC28 : --------------------------MASPKE--NDDVPML-PISDTS-- :  19
BoCNGC21 : --------------------------MASPKE--NDDVPML-PISDTS-- :  19
BoCNGC20 : ------------------------------ME--KDDVPML-PVSDSSSL :  17
BoCNGC22 : --------------------------MAPPNE--KDDVPML-PISSSSSS :  21
BrCNGC23 : --------------------------MAPPNE--NDDALKL-PVLDTSSS :  21
BrCNGC24 : ---------------------------------------MLPISDASSSS :  11
BoCNGC23 : --------------------------MVSPNK--NDKIHILPISDASSSS :  22
BrCNGC25 : --------------------------MTSPNE--NDQV----SIPEATSR :  18
BoCNGC24 : --------------------------MISPNE--NDQV----SIPEATSR :  18
BrCNGC26 : --------------------------MASPNENESDEFPMLRQFPEARS- :  23
BoCNGC26 : --------------------------MAYPNE--SDEFPMLRQVPEARSR :  22
BrCNGC29 : --------------------------MASPNE--KDEFPILLPVPEARSR :  22
BoCNGC25 : --------------------------MASPNE--KDEFPILLPVSEARPR :  22
BrCNGC19 : ----------------------------MATEQEFTR------ASRVSGA :  16
BoCNGC15 : ----------------------------MATEQEFTR------ASRVSGA :  16
BrCNGC20 : ----------------------------MATEQEFTR------ASRVSRA :  16
BoCNGC16 : ----------------------------MATEQEFTR------ASRVSRA :  16
BrCNGC21 : ----------------------------MPSHTNFLFRWIGLFSQKLRRE :  22
BoCNGC17 : ----------------------------MPSHTNFLFRWIGLFSQKLRRE :  22


                   60         *        80         *       100
BrCNGC1  : --------------MERAS--------TIQSVHENIK--SVRGQLKKVYK :  26
BoCNGC3  : --------------MERAS--------TMQSVHENIK--SVRGQLKKVYK :  26
BrCNGC2  : ----MTIFSVQSTLFTRAS--------VALLSSNGLKRFSFASSFSSAAL :  38
BrCNGC4  : -------------------------------------------------- :   -
BrCNGC3  : KRNTFVKFTENEDSWNRPS--------VTSVIKKTVRRSFEKGSEKIRTF :  48
BoCNGC2  : KRNTFVKFTENEDSWNRPS--------VTSVIKKTVRRSFEKGSEKIRNF :  48
BrCNGC5  : RVR-FKEPSSTEFGYGRRAR------PSLNAVLDNVRRGFEKGSDKIRTF :  50
BoCNGC1  : RVRWFKEPSSTEYGYGRRAR------PSLNAVLNNVRRGFEKGSDKIRTF :  51
BrCNGC6  : KSVRFHDWKSDKASDVEYS---EVPDGLYRRAISSISDKFHRSSLRIKML :  91
BrCNGC7  : KFVRFQDWKSDKTSDVEYSGRNEPPNGIFRRTITSISDKFHRSSARIKTF :  56
BrCNGC8  : -MMTKRNCFG-FPVKNRGSE--------KKRASKSFREGVKIGSEGLFSI :  40
BoCNGC8  : GMMTKRNCFG-FPVKNRGSE--------KKRASKSFREGVKIGSEGLFSI :  69
BrCNGC9  : SLTRQHNYSSSISGPLHPIQ-------GSHNTSGSFKKRFQKGSKGLKSI :  93
BoCNGC7  : SLTRQHNYSSSISGPLHPIQ-------GSHNASGSFKKRFQKGSKGLKSI :  65
BrCNGC10 : DNTGLNKCTLNIQAPKRFAQ-------GSKTSSGSFKKGFRKGSEGLWSI :  78
BoCNGC5  : DNTGLNKCTLNIQAPKRFAQ-------GSKTSSGSFKNGFRKGSEGLWSI :  78
BrCNGC11 : PEAGLNRCTINIQRPKRFTQ-------ANKTSSGSFKKGFRKGSEGLWSI :  68
BoCNGC4  : PEAGLNRCTLNIQRPKRFTQ-------ATKASSGSFKKGFRKGSEGLWSI :  68
BrCNGC12 : AEAGLNKCTLNLQGPSRANG--AGQGNNNNASSGSFKKGFRRGSKGLWSL :  56
BoCNGC6  : SEAGLSKCTLNLQGQSRANGG-TGQGNNNNASSGSFKKGFRRGSKGLWSL :  83
BrCNGC13 : GNSRSVRFEEDSEVTKPQAVHEETAVKLKFKINGAQISPRKNVKKMTR-- :  51
```

```
BoCNGC9  : GNSRSVRFEEDSEVTKPQAVHEETAEKLKFKINGAQISPRKNVKKMTR-- :  51
BrCNGC14 : NKIRSLRFL---------------------------LPETITSAASN--- :  21
BoCNGC13 : NKIRSLRFL---------------------------LPETITSAASN--- :  21
BrCNGC15 : HLHTSARFRN--------------------------FPTAFSRRHHN--- :  25
BoCNGC14 : HLHTSARFRN--------------------------FPTAFSRRHHN--- :  25
BrCNGC16 : RKDKILMLYSDKKEPKEAIWAVN-DPMSKS--YKLSLPSALKPPDNNLLS :  50
BoCNGC10 : SKDKLLMLYSDKKEPKEAIWAVN-DPMSKS--YKLSLPSALKPPPDNLLA :  50
BrCNGC17 : KRDNTVRFYGEEKQTLEATEKRQPLPMFKPSTTQFLKPELVIPKKTNKTR :  53
BoCNGC12 : KRDNTVRFYGEEKQTLEATEKRQPLPMLKPSTTQFLKPELVIPKKTNKTR :  53
BrCNGC18 : KRDNTVRFYGDEKQTLEVTEKRLPLPMFKSSAAPFQKQELGTSKKS---- :  49
BoCNGC11 : KRDNTVRFYGDEKQTLEVTEKRLPLPMFKSSAALFQKQELGTSKKS---- :  49
BrCNGC22 : SRTRPFTSRSRSVSLSNTSSTIDVFENSSTVVLGYTDPLGTQRQPQL-VQ :  70
BoCNGC19 : SRTRPFTFRSRSVSLSNTSSTIDVFENSSTVVLSYTDPLGTQRQPQL-VQ :  70
BrCNGC27 : SRTMPFTSRSRSTSLANNSSTIDVF-NSSTVVLGYTDPLGTQRRPPL-VQ :  69
BoCNGC18 : SRTMPFTSRSRSTSLANNSSTIDVF-NSSTVVLGYTNHLGTQRRPPL-VQ :  69
BrCNGC28 : -RTRPFTSRSRSVSLSNTCSTIDGF-DSSTVVLGYTGPLRAQRRPPL-VQ :  66
BoCNGC21 : -RTRPFTSRSRSVSLSNTCSTIDGF-DSSTVVLGYTGPLRAQRRPPL-VQ :  66
BoCNGC20 : SRTRPFTSRSRSVSLANTSSIIDGF-DSSTVVLGYTGPLRTRRRPPL-VQ :  65
BoCNGC22 : SRTRSFTSRFRSTSLANTSSAIDGF-DSSNVVLGYTGPLQTYGRPAF-VQ :  69
BrCNGC23 : SRTRPFTFRSRNVSLPNTSSTSEGL-DSSTVVLRYTDPHRTQRPPPS-VQ :  69
BrCNGC24 : SQTRVFTSRTRSVPLSNPTEETG---NSKAATLGYAGSLPSQRPPLFP-- :  56
BoCNGC23 : SQTRVFTSRTRSVPLSNPTDETG---NSNAVTLGYAGSLLSQRPPLVP-- :  67
BrCNGC25 : AHTRAFNFKNRSVSLSNSTYYIDGC-DNSKVALGYTVPIRTQRRPP---- :  63
BoCNGC24 : AHTGAFNFKNRSVSLSNSTYYIDGC-DKSKVALGYTVPIRTQRRPP---- :  63
BrCNGC26 : ---RALHSRNRSISFSDSTYSSNRV-ENS------SGPRRTQSRPSPSVH :  63
BoCNGC26 : AQSRALHSRNRSVSFSDSTYSTNRV-ENS------SGPRRTQSRPSPSVH :  65
BrCNGC29 : ANTRAFNSRNRSVSFSNSTYSTNRV-DNSSVVLGYTGPLRTQRRLPPSVQ :  71
BoCNGC25 : ANTRALNSRNRSVSFSNSTYSTNRV-DNSSVVLGYTGPLRTQRRPPPSVQ :  71
BrCNGC19 : SSSVGYYSDEDYKDEEEEEEEEE-----------EEMEETEKDEEEEEPR :  55
BoCNGC15 : SSSVGCYSDEDYKDEEEEEEEEE-----------EEMEETEKDEEEEEPR :  55
BrCNGC20 : SSSIGYYSDEDYTTDEEENEEEE-----------MEELE-EAEEEEEETH :  54
BoCNGC16 : SSSIGYYSDEDYTTEEEEDEEEE-----------MEEQE-EEEEEEEETH :  54
BrCNGC21 : TTEISENNGGESSSSSDDTPVLSSGECYACTQVGVPAFHSTSCDQANAPE :  72
BoCNGC17 : TTGISENNVGESSSS-DDTPVLSSGECYACTQVGVPAFHSTSCDQANAPE :  71


                    *      120         *      140         *
BrCNGC1  : TLKTLENWRK---------------------------------------- :  36
BoCNGC3  : TLNTLENWRK---------------------------------------- :  36
BrCNGC2  : YSPPLPKTKKR--------------------------------------- :  49
BrCNGC4  : -MKTLN-TRK---------------------------------------- :   8
BrCNGC3  : KQQPLTFHSQK--------------------------------------- :  59
BoCNGC2  : KQQPLTFHSQK--------------------------------------- :  59
BrCNGC5  : KRPLSFNSHKN--------------------------------------- :  61
BoCNGC1  : KKPLSFNSHKN--------------------------------------- :  62
BrCNGC6  : R---TSYSFKE--------------------------------------- :  99
BrCNGC7  : RRTYKSYSFKE--------------------------------------- :  67
BrCNGC8  : GK----SVTRA--------------------------------------- :  47
BoCNGC8  : GK----SVTRA--------------------------------------- :  76
```

```
BrCNGC9  : GRSLGFGVYRA--------------------------------------- : 104
BoCNGC7  : GRSLGFGVYRA--------------------------------------- :  76
BrCNGC10 : GRSIGLGVSRA--------------------------------------- :  89
BoCNGC5  : GRSIGLGVSRA--------------------------------------- :  89
BrCNGC11 : GRSIGLGVSRA--------------------------------------- :  79
BoCNGC4  : GRSIGLGVSRA--------------------------------------- :  79
BrCNGC12 : GRSIGLGVSRA--------------------------------------- :  67
BoCNGC6  : GRSIGL-VSRA--------------------------------------- :  93
BrCNGC13 : GKSFKDKVLSR--------------------------------------- :  62
BoCNGC9  : GKSFKAKVLSR--------------------------------------- :  62
BrCNGC14 : RGSVAVRYGSQ--------------------------------------- :  32
BoCNGC13 : RGSVAVRYGSQ--------------------------------------- :  32
BrCNGC15 : NNDLQNQRGRS--------------------------------------- :  36
BoCNGC14 : NNDLQNQRGRS--------------------------------------- :  36
BrCNGC16 : GNRISRYTDNN--------------------------------------- :  61
BoCNGC10 : GNRISRYTDNN--------------------------------------- :  61
BrCNGC17 : LFKLPRFGGLK--------------------------------------- :  64
BoCNGC12 : LFKLPRFGGLK--------------------------------------- :  64
BrCNGC18 : IFKIPRFGRFK--------------------------------------- :  60
BoCNGC11 : IFKIPRFGRFK--------------------------------------- :  60
BrCNGC22 : MGDPISSTRNL------ELV------------------------------ :  84
BoCNGC19 : MGDPISSTRNL------ELVSKHAHPYGTTCPSDYNREAAQI-------- : 106
BrCNGC27 : MSYPLSSTRSP------EPRFALPPPSTGASYDSVGASSSQP-------- : 105
BoCNGC18 : MSDPLSSTRSP------EPRFALPPPSTGASSDSVGASSSQP-------- : 105
BrCNGC28 : MSGPLSSTRTP------EPLFLLPPPS-----DSVGISSSQPERYPSFAT : 105
BoCNGC21 : MSGPLSSTRNP------EPLFLLPPP------DSVGISSSQPERYPSFAT : 104
BoCNGC20 : MSGPLSSTRSS------EPLFLPPPPT-----STRDVSSSQPERYPSFTA : 104
BoCNGC22 : MSASLPSTLIP------EPLFLHPTPTGG-SSHSIGVSSSQPESCP-FAA : 111
BrCNGC23 : MNGPLFSTSSP------EPLILLPPPSTGGSSDPVGVSSSQPERYPSFAA : 113
BrCNGC24 : MTGPLSSSTRR---------------SSGYFGDLEEVNSSDN-------- :  83
BoCNGC23 : MTGPLSSSTRRPEPLFPRPAPPPTRRSSGYFGDLEEVNSSDN-------- : 109
BrCNGC25 : --GPLYSTPRP--ESHFPPS-IEPPDS---SS-TVDVRSEDE-------- :  96
BoCNGC24 : --GPLYSTLRP--ESLLPPS-IEPPDS---SS-TVDVRSEDE-------- :  96
BrCNGC26 : MSGPLYDTRRP-DQSFFPPSPVQPPASSLSSS-TVDIPSEEVV------- : 104
BoCNGC26 : MSGPLYDTRRPPDQSFFPPSPVQPPESSLSSSTTVDIPSEEVV------- : 108
BrCNGC29 : MSGPLYSTRRP-DQSFFPPSPVQPPDS---SS-TVDVPSEED-------- : 108
BoCNGC25 : MSSPLYSTRRP-DQSFFPPSPVQPPDSSLSSS-TVDVPSEED-------- : 111
BrCNGC19 : VRVTCGGRR----------------------------------------- :  64
BoCNGC15 : VRVTCGGRR----------------------------------------- :  64
BrCNGC20 : VGGTCGIRRR---------------------------------------- :  64
BoCNGC16 : VGVTCGIRRR---------------------------------------- :  64
BrCNGC21 : WRASAGSSLVP--------------------------------------- :  83
BoCNGC17 : WRASAGSSLVP--------------------------------------- :  82


                  160         *        180         *       200
BrCNGC1  : -------------------------------------------------- :   -
BoCNGC3  : -------------------------------------------------- :   -
BrCNGC2  : -------------------------------------------------- :   -
```

```
BrCNGC4  : -------------------------------------------------- :   -
BrCNGC3  : -------------------------------------------------- :   -
BoCNGC2  : -------------------------------------------------- :   -
BrCNGC5  : -------------------------------------------------- :   -
BoCNGC1  : -------------------------------------------------- :   -
BrCNGC6  : -------------------------------------------------- :   -
BrCNGC7  : -------------------------------------------------- :   -
BrCNGC8  : -------------------------------------------------- :   -
BoCNGC8  : -------------------------------------------------- :   -
BrCNGC9  : -------------------------------------------------- :   -
BoCNGC7  : -------------------------------------------------- :   -
BrCNGC10 : -------------------------------------------------- :   -
BoCNGC5  : -------------------------------------------------- :   -
BrCNGC11 : -------------------------------------------------- :   -
BoCNGC4  : -------------------------------------------------- :   -
BrCNGC12 : -------------------------------------------------- :   -
BoCNGC6  : -------------------------------------------------- :   -
BrCNGC13 : -------------------------------------------------- :   -
BoCNGC9  : -------------------------------------------------- :   -
BrCNGC14 : -------------------------------------------------- :   -
BoCNGC13 : -------------------------------------------------- :   -
BrCNGC15 : -------------------------------------------------- :   -
BoCNGC14 : -------------------------------------------------- :   -
BrCNGC16 : -------------------------------------------------- :   -
BoCNGC10 : -------------------------------------------------- :   -
BrCNGC17 : -------------------------------------------------- :   -
BoCNGC12 : -------------------------------------------------- :   -
BrCNGC18 : -------------------------------------------------- :   -
BoCNGC11 : -------------------------------------------------- :   -
BrCNGC22 : -------------------------------------------------- :   -
BoCNGC19 : -------------------------------PTP---------------- : 109
BrCNGC27 : -------------------------------NERNHAYSRKAAQSRTPRV : 124
BoCNGC18 : -------------------------------NERNHAYS------RTPRV : 118
BrCNGC28 : LEHKKSDDEFVLKHANLLRSGQLGMCNDPYCTTCPSYYNRKAAQIPSSRV : 155
BoCNGC21 : LEHKKSDDEFVLKHANLLRSGQLGMCNDPYCTTCPSYYNRKAAQIPSSRV : 154
BoCNGC20 : LEHKNSEEEFVLKHAHLLRSGQLGMCNDPYCTTCPSYYNRKAAQIPTSRV : 154
BoCNGC22 : LEHKNSDDELGLG------SGQLEVCNDPYCTTCPSYYNPKPAQIPPS-- : 153
BrCNGC23 : LEHDNSDDNSVLN-PHLLRSEKFGVCNDPYCTTCPSYYNRKADQVPTSRV : 162
BrCNGC24 : --------DELLKHAHRLRSGKLGMCNDPYCTTCPSNYNPKASRLPNP-T : 124
BoCNGC23 : --------DELLKHAHRLRSGKLGMCNDPYCTTCPSNYNPKASRLPNP-T : 150
BrCNGC25 : ---------SVLENANILKSGQLGMCNEPYCTTCPSYYSHQSANFHTS-K : 136
BoCNGC24 : ---------SVVKNANILTSGQLGMCNEPYCTTCPSYYSHQSANFHTS-K : 136
BrCNGC26 : --------EALLKNANLLKSGQLGMCNDPYCTTCPSYYNLQAAQFHTYGV : 146
BoCNGC26 : --------EALLKNANLLKSGQLGMCNDPYCTTCPSYYNLQAAQFHTYGV : 150
BrCNGC29 : --------EVVLKNANLLKSGQLGMCNDPYCTTCPSYYNRQAAQFHTYRV : 150
BoCNGC25 : --------EVVLKNANLLKSGQLGMCNDPYCTTCPSYYNRQAAQFHTNRV : 153
BrCNGC19 : -------------------------------------------------- :   -
BoCNGC15 : -------------------------------------------------- :   -
BrCNGC20 : -------------------------------------------------- :   -
```

```
BoCNGC16 : -------------------------------------------------- :   -
BrCNGC21 : -------------------------------------------------- :   -
BoCNGC17 : -------------------------------------------------- :   -


                    *       220         *       240         *
BrCNGC1  : -------------------------------------------------- :   -
BoCNGC3  : -------------------------------------------------- :   -
BrCNGC2  : -------------------------------------------------- :   -
BrCNGC4  : -------------------------------------------------- :   -
BrCNGC3  : -------------------------KNENKKK---IIRVMNPNDSYLQNW :  81
BoCNGC2  : -------------------------KNENKKK---IIRVMNPNDSYLQNW :  81
BrCNGC5  : -------------------------EEKRNATGTQKKNIINPQGSFLQNW :  86
BoCNGC1  : -------------------------EEKRNATGTQKKNIINPQGSFLQNW :  87
BrCNGC6  : -------------------------TVSKGIVS--THEILDPQGTFLQKW : 122
BrCNGC7  : -------------------------AVSKGIDS--SHKILDPQGPFLQRW :  90
BrCNGC8  : -------------------------VFPEDLRIS-EKKIFDPQDKTLLIW :  71
BoCNGC8  : -------------------------VFPEDLRIS-EKKIFDPQDKTLLIW : 100
BrCNGC9  : -------------------------VFPEDLKVS-EKKIFDPQDKTLLFC : 128
BoCNGC7  : -------------------------VFPEDLKVS-EKKIFDPQDKTLLFC : 100
BrCNGC10 : -------------------------VFPEDLKVS-EKKIFDPQDKFLLLC : 113
BoCNGC5  : -------------------------VFPEDLKVS-EKKIFDPQDKFLLLC : 113
BrCNGC11 : -------------------------VFPEDLEVS-EKKIFDPQDKFLLLC : 103
BoCNGC4  : -------------------------VFPEDLEVS-EKKIFDPQDKFLLLC : 103
BrCNGC12 : -------------------------VFPEDLKVS-ERKIFDPQDKFLLLC :  91
BoCNGC6  : -------------------------VFPEDLKVS-EKKIFDPQDKFLLLC : 117
BrCNGC13 : -------------------------VFTEDLGRV-KNKILDPRGQTIRKW :  86
BoCNGC9  : -------------------------VFTEDLGRV-KNKILDPRGQTIRRW :  86
BrCNGC14 : -------------------------VLPWR------HQILDPDSNIVTYW :  51
BoCNGC13 : -------------------------VLPWR------HQILDPDSSIVTYW :  51
BrCNGC15 : -------------------------VFSELG-----DTTLDPSGDLITRW :  56
BoCNGC14 : -------------------------VFSELG-----DTTLDPSGDLITRW :  56
BrCNGC16 : -------------------------KSKSSKPSW-YKTILDPGSEIVLKW :  85
BoCNGC10 : -------------------------KTKSSKLSW-YKTILDPGSEIVLKW :  85
BrCNGC17 : -------------------------VFPENFEIE-RDKILDPGGDVVLQW :  88
BoCNGC12 : -------------------------VFPENFEIE-RDKILDPGGDVVLQW :  88
BrCNGC18 : -------------------------VFPENFEIE-RDKILDPGGDVVLQW :  84
BoCNGC11 : -------------------------VFPENFEIE-RDKILDPGGDVVLQW :  84
BrCNGC22 : -------------HNARDDDAKGWARRFVTSVDKYLPKIMEPDSKEVGGW : 121
BoCNGC19 : -RVSDS-----MVHNARDDDAKGWARRFATSVDKYLPKIMEPNSKQVRGW : 153
BrCNGC27 : FATSDF-----TLHNALDDDAKGWA--------KYFSGIIYPESNYVQLW : 161
BoCNGC18 : FATSDF-----TLHNALDDDAKGWA--------KYFSGIIYPESNFVQIW : 155
BrCNGC28 : SAFFDS-----KFHNALYDDAKGWARRFATTANRYLPGIMNPHSKFVQSW : 200
BoCNGC21 : SAFFDS-----KFHNALYDDAKGWARRFATTANRYLPGIMNPHSKFVQSW : 199
BoCNGC20 : SAIFDS-----TFH-----DAKGWARRFATSINRHLPGIMNPHSKFVQSW : 194
BoCNGC22 : -----------KLHHAMCDDARGWATRFVTSINKFLTGIINPHYKFVQRW : 192
BrCNGC23 : PAIFYS-----MFHSALYEDAKARARRFATSVNRHLPGIMNPHSIFIQSW : 207
BrCNGC24 : VSAST-------FHNALYDDARSWARRFASSVNRCLPGIMNPHSKFVQIW : 167
BoCNGC23 : VSAST-------FHNALYDDARSWARRFASSVNRCLPGIMNPHSKVVQIW : 193
```

```
BrCNGC25 : VSDSR-------FHTVLYDDARGWAKRFASCVRRCVPGIMNPHSKFVQVW : 179
BoCNGC24 : VSDSR-------FHTALYDDARGWAKRFASSVRRCVPGIMNPHSKFVQVW : 179
BrCNGC26 : VSDSRTQVNVTHNRQALHDYDRGWAKLFASYVRRCVPGIINPHSKFVQMW : 196
BoCNGC26 : VSDSRTQVNATHNRQALHDYDRGWDKLFASYVRRCVPGIINPHSKFVQMW : 200
BrCNGC29 : VSDSR-------FRTALYDDARGWAKRFASSVRKMVPGIMNPHSKFVQMW : 193
BoCNGC25 : VSDSR-------FRTALYDDARGWAKRFASSVRKLIPGIMNPHSKFVQMW : 196
BrCNGC19 : -----------------NGSPGSYNKWMM------LGRILDPRSKLVQEW :  91
BoCNGC15 : -----------------NGSPGSYNKWMM------LGRILDPRSKLVQEW :  91
BrCNGC20 : -----------------NGSSSSYNKWMV------LGRILDPRSKLVQEW :  91
BoCNGC16 : -----------------NGSSSSYNKWMM------LGRILDPRSKLVQEW :  91
BrCNGC21 : ---------------IQEGSAPSPVRARIRRLKGPFGEVLDPRSKRVQRW : 118
BoCNGC17 : ---------------IQEGSAPDPVRARFRRLKGPFGEVLDPRSKRVQRW : 117
                                                 p

                    260         *        280         *         300
BrCNGC1  : --AILLVCVVALG--------------------------------VDPLF :  52
BoCNGC3  : --AILLVCVVALG--------------------------------VDPLF :  52
BrCNGC2  : --RFPIVSAVDIGGVTVARNDDDPTNNVPDSIFESNYANCYHQATVDPLF :  97
BrCNGC4  : --IVLLVCLVALA--------------------------------IDPLF :  24
BrCNGC3  : NKIFLLLCVVALA--------------------------------FDPLF :  99
BoCNGC2  : NKIFLLLCVVALA--------------------------------FDPLF :  99
BrCNGC5  : NKIFLFASVIALA--------------------------------IDPLF : 104
BoCNGC1  : NKTFLFASVIALA--------------------------------IDPLF : 105
BrCNGC6  : NKIFVLACIIAVS--------------------------------VDPLF : 140
BrCNGC7  : NKIFVLACIIAVS--------------------------------LDPLF : 108
BrCNGC8  : NRMLVISCILAVS--------------------------------VDPLF :  89
BoCNGC8  : NRMLVISCILAVS--------------------------------VDPLF : 118
BrCNGC9  : NKLFVISCILSVF--------------------------------VDPFF : 146
BoCNGC7  : NKLFVVSCILSVF--------------------------------VDPFF : 118
BrCNGC10 : NKLFVASCILAVS--------------------------------VDPLF : 131
BoCNGC5  : NKLFVASCILAVS--------------------------------VDPLF : 131
BrCNGC11 : NKLFVASCILAVS--------------------------------VDPLF : 121
BoCNGC4  : NKLFVASCILAVS--------------------------------VDPLF : 121
BrCNGC12 : NKLFVTSCILAVS--------------------------------VDPLF : 109
BoCNGC6  : NKLFVTSCILAVS--------------------------------VDPLF : 135
BrCNGC13 : NKLFLIACLVSLF--------------------------------VDPLF : 104
BoCNGC9  : NKLFLIACLVSLF--------------------------------VDPLF : 104
BrCNGC14 : NHVFLVTSILALF--------------------------------LDPFY :  69
BoCNGC13 : NHVFLVTSILALF--------------------------------LDPFY :  69
BrCNGC15 : NHIFLITCLLALF--------------------------------LDPLY :  74
BoCNGC14 : NHIFLITCLLALF--------------------------------LDPLY :  74
BrCNGC16 : NWVFIISCMLALF--------------------------------IDPLY : 103
BoCNGC10 : NWVFIISCMLALF--------------------------------IDPLY : 103
BrCNGC17 : NRVFLFWCLVALY--------------------------------VDPLF : 106
BoCNGC12 : NRVFLFWCLVALY--------------------------------VDPLF : 106
BrCNGC18 : NRVFLFWCLVALY--------------------------------VDPLF : 102
BoCNGC11 : NRVFLFWCLVALY--------------------------------VDPLF : 102
BrCNGC22 : TIFFAFSCLLSIF--------------------------------VDPLF : 139
BoCNGC19 : TIFFAFSCLLSIF--------------------------------VDPLF : 171
```

```
BrCNGC27 : TTFFVLSCLCSIF---------------------------------VDPLF : 179
BoCNGC18 : TTFFALSCLCSIF---------------------------------VDPLF : 173
BrCNGC28 : TKFFALSCLLAIF---------------------------------IDPLF : 218
BoCNGC21 : TKFFALSCLLAIF---------------------------------IDPLF : 217
BoCNGC20 : TKFFAFSCLLAIF---------------------------------IDPLF : 212
BoCNGC22 : TKLFAS--WLVIF---------------------------------IDPLF : 208
BrCNGC23 : TRLFSLSCLLSIF---------------------------------LDPLF : 225
BrCNGC24 : TKFFAVSSLLAIF---------------------------------IDPLF : 185
BoCNGC23 : TKFFAVSSLLAIF---------------------------------IDPLF : 211
BrCNGC25 : TRFLAFSCLMAIF---------------------------------IDPTF : 197
BoCNGC24 : TRFLAFSCLMAIF---------------------------------IDPTF : 197
BrCNGC26 : TRFLAFSCLLSIF---------------------------------IDPAF : 214
BoCNGC26 : TRFLAFSCLLSIF---------------------------------IDPAF : 218
BrCNGC29 : TRFLAFSCLVAIF---------------------------------IDPAF : 211
BoCNGC25 : TRFLAFSCLVAIF---------------------------------IDPAF : 214
BrCNGC19 : NRVFLLVCATGLF---------------------------------VDPLF : 109
BoCNGC15 : NRVFLLVCATGLF---------------------------------VDPLF : 109
BrCNGC20 : NKVFLLVCATGLF---------------------------------VDPLF : 109
BoCNGC16 : NKVFLLVCATGLF---------------------------------VDPLF : 109
BrCNGC21 : NRALLLARGMALA---------------------------------VDPLF : 136
BoCNGC17 : NRALLLARGMALA---------------------------------VDPLF : 135
                   c   6                                 DP15

                     *        320        *        340        *
BrCNGC1  : LFIPVIDSP---NFCFTFDKKLAAVVSAIRTFIDTFYVIHIIFNF----I :  95
BoCNGC3  : LFIPVIDSP---NFCFTFDKKLAAVVSAIRTFIDTFYVIHIIFNF----I :  95
BrCNGC2  : FFIPVIDSH---KFCFTLDKKLGVAVCVLRTLIDVFYVIHFIFHF----I : 140
BrCNGC4  : LFIPVIDSH---RLCFTYDKKLVATACVFRTLIDTFYGIHIIFHF----I :  67
BrCNGC3  : FFIPVVDPD---RFCLKLDKKLEAVACVFRTFIDAFYLVHMLFQF----N : 142
BoCNGC2  : FFIPVVDPG---RFCLKLDKKLEAVACVFRTFIDAFYVVHMLFQF----N : 142
BrCNGC5  : FYIPIVDGK---KHCLNLHSSLEIAASVLRTFVDAFYIIHIVFQF----R : 147
BoCNGC1  : FYIPIVDGK---KHCLNLHSSLEIAASVLRTFVDAFYIIHIVFQF----R : 148
BrCNGC6  : FYVPVLDKA---NNCLDVDKKMQTTASVLRSVTDIFYAIHMVFQF----R : 183
BrCNGC7  : FYVPVVDDA---KKCLGLDNKMEITASVLRSFTDIFYVIHIIFQF----R : 151
BrCNGC8  : FYLPIVDNS--GSSCIGIDTKLAVTTTTLRTILDVFYLTRMALQF----R : 133
BoCNGC8  : FYLPIVDNS--GSSCIGIDTKLAVTTTTLRTILDVFYLTRMALQF----R : 162
BrCNGC9  : FYLPVIDGE---SKCLGIDRKLAITATTFRTFIDVFYLAHMALQL----R : 189
BoCNGC7  : FYLPVINGE---SKCLGIDRKLAITATTFRTFIDVFYLAHMALQL----R : 161
BrCNGC10 : LYLPFINDK---AKCIGIDRKLAIVATTLRTVIDSFYLFHMALRF----R : 174
BoCNGC5  : LYLPFINDK---AKCIGIDRKLAIVATTLRTVIDSFYLFHMALRF----R : 174
BrCNGC11 : LFLPFINDK---AKCVGIDRKLAIVTTTLRTVIDSFYLFHMALRF----R : 164
BoCNGC4  : LFLPFINDK---AKCVGIDRKLAIVTTTLRTVIDSFYLFHMALRF----R : 164
BrCNGC12 : LYLPFINDS---GKCIGIDRRLATIATTLRTFIDVFYLFRMALQF----R : 152
BoCNGC6  : LYLPFINDS---GKCIGIDRRLATIATTLRTFIDVFYLFRMALQF----R : 178
BrCNGC13 : FFLPVMR---K-EACITIGIRLEVVLTVIRSLADAFYIAQIVIRF----R : 146
BoCNGC9  : FFLPVMR---K-EACITIGIRLEVVLTVIRSLADAFYIAQIVIRF----R : 146
BrCNGC14 : FYAPYVG---G-PACLSVDVGLAATVTFFRSVADLFHLLHIFMKF----R : 111
BoCNGC13 : FYAPYVG---G-PACLSVDVGLAATVTFFRSVADLFHLLHIFMKF----R : 111
BrCNGC15 : FYPPIVQA--G-TACMSIDIGFGILVTFFRTLADFSFLIHILLKF----K : 117
```

```
BoCNGC14 : FYLPIVQA--G-TACMSIDIGFGILVTFFRTLADFSFLIHILLKF----K : 117
BrCNGC16 : FFVPAIGGNKD-YPCAKTDTNLRILVTFFRTIADLFYLLHIFIKF----R : 148
BoCNGC10 : FFVPAIGGNKD-YPCAKTDTNLRILVTFFRTIADLFYLLHIFIKF----R : 148
BrCNGC17 : FFLSSVKNTGR-SSCMTTDLKLGIVITFFRTLADLFYVLHIVIKF----R : 151
BoCNGC12 : FFLSSVKNTGR-SSCMTTDLKLGIVVTFFRTLADLFYVLHIVIKF----R : 151
BrCNGC18 : FFLYSVKRTGR-SSCMTTDLNLGIVVTFFRTLADLFYVLHIVIKF----R : 147
BoCNGC11 : FFLYSVKRTGR-SSCMTTDLNLGIVVTFFRTLADLFYVLHIVIKF----R : 147
BrCNGC22 : FFPIQVSESGK---CIRIDEKMARVLVALRSLTDFLYFVNTLLQC----R : 182
BoCNGC19 : FFPIEVSQSGK---CIRIDEDMAIVLVALRSLTDVLYFVNILLQ------ : 212
BrCNGC27 : FYPIEIYKEER---CIKIDWWTTNVFVIVRTITDGLYALNIVLQF----R : 222
BoCNGC18 : FYPIEIYKEER---CIKIDWWTTNVFVIVRSITDGLYALNIVLQF----R : 216
BrCNGC28 : FFLILVKQNDK---CIVIDWPMAKAFVAVRSVTDILFSVNILLQF----R : 261
BoCNGC21 : FFLIFVKQNDK---CIMIDWPMAKAFVAVRSVTDILFSVNILLQF----R : 260
BoCNGC20 : FFLILVKQNNN---CIEIDWPMAHTFVAVRSVTDVLFSVNILLQF----R : 255
BoCNGC22 : LFLIKVKENDK---CIMIDWPVAKAFIAVRSVTDVLFFVNIMLQF----R : 251
BrCNGC23 : VFLTLVKQNNK---CIVIDWPMAKAFIIVRSVTDALFSVNILLQF----R : 268
BrCNGC24 : FFIILVQKNNK---CIVIDWPIATAFVIVRTLTDVIFFANMLLQ----FR : 228
BoCNGC23 : FFIILVQKNNK---CVVIDWPIATAFVIFRTLTDVIFFANMLLQAPVPFR : 258
BrCNGC25 : VFLLLIRHDNK---CIEIDWPKTTVLVSLRSMFDLIFFINILLQ----FR : 240
BoCNGC24 : VFLLLIRHDNK---CIEIDWTKTTVLVSLRSMFDLIFFINILLQ----FR : 240
BrCNGC26 : VYLLLIRDDNK---CTDIDWPKATLFLFLRSMSDFIFFINILLQ----FR : 257
BoCNGC26 : --------DNK---CTDIDWPKATIFAFLRSMSDFIFFINILLQ----FR : 253
BrCNGC29 : LFLLSIRQDNK---CIEFDWPKTKVFVSLRSMSDLIFFMNILLQ----FR : 254
BoCNGC25 : LFLLSIRQDNK---CIEIDWPKTKVFVSLRTMSDLIFFINILLQ----FR : 257
BrCNGC19 : LYTLSVN---DACMCLLVDGWLALTVTALRSMTDLLHLWNILIQFKIARW : 156
BoCNGC15 : LYTLSVN---DACMCLLVDGWLALTVTALRSVTDLLHLWNILIQFKIARR : 156
BrCNGC20 : LYTISVN---DACMCLLVDGWLALSITAVRSMTDLLHLWNIWIQFKIARR : 156
BoCNGC16 : LYTISVN---DACMCLLVDGWLALTITAVRSMTDLLHLWNIWIQFKIARR : 156
BrCNGC21 : FYALSIGRT-TGPACLYMDGAFAAVVTVVRTCLDALHLWHVWLQFRLA-- : 183
BoCNGC17 : FYALSIGRT-TGPACLYMDGAFAAVVTVVRTCLDALHLWHVWLQFRLA-- : 182
                          C   d           R3 D

                       360       *       380       *       400
BrCNGC1  : TEFIAPRS---QVSLRGELIVHSKAIRKRLF--FFQFIVDICSVIPIPQ- : 139
BoCNGC3  : TEFIAPRS---QVSLRGELIVHSKATRKRLF--FFHFIVDICSVIPIPQ- : 139
BrCNGC2  : TELVAPRS---QASLRG----NSKPIRKRLF--FFYFSVDIVSVLPIPQ- : 180
BrCNGC4  : TKHIAPRS---QVSFRGETTVYSVAISERHL--IFYFIVDIVSVLPIP-- : 110
BrCNGC3  : TGFIAPSS---RGFGRGELVQSSKKIAVRYL--KSYFIIDVLSILPIPQ- : 186
BoCNGC2  : TGFIAPSS---RGFGRGELVQSYKKIAVRYL--KSYFIIDVLSILPIPQ- : 186
BrCNGC5  : TAYVSPLS---RVFGRGELVEDPKAIALKYI--SSYFIIDVLSILPLPQ- : 191
BoCNGC1  : TAYVSPLS---RVFGRGELVEDPKAIALKYL--SSYFIIDVLSILPLPQ- : 192
BrCNGC6  : TGFIAPSS---RVFGRGVLVEDRRKIAKRYL--SSHFIIDILAVLPLP-- : 226
BrCNGC7  : TGFIAPSS---RVFGRGVLVEDTRQIAIRYL--SSHFIIDILAVLPLPQV : 196
BrCNGC8  : TAYIAPSS---RVFGRGELVIDPAKIAQRYL--TRYFIVDFLAVLPLPQ- : 177
BoCNGC8  : TAYIAPSS---RVFGRGELVIDPAKIAQRYL--TRYFIVDFLAVLPLPQ- : 206
BrCNGC9  : TAYIAPSS---RVFGRGELVIDPAQIAKRYL--QRWFIIDFLSVLPVPQ- : 233
BoCNGC7  : TAYIAPSS---RVFGRGELVIDPAQIAKRYL--QRWFIIDFLSVLPVPQ- : 205
BrCNGC10 : TAYVAPSS---RVFGRGELVIDPKQIAKRYL--RQYFIIDLLSVLPLPQ- : 218
BoCNGC5  : TAYVAPSS---RVFGRGELVIDPKQIAKRYL--QQYFIIDLLSVLPLPQ- : 218
```

```
BrCNGC11 : TAYVAPSS---RVFGRGELVIDPAQIAKRYL--QQYFIIDLLSVLPVPQ- : 208
BoCNGC4  : TAYVAPSS---RVFGRGELVIDPAQIAKRYL--QQYFIIDLLSVLPVPQ- : 208
BrCNGC12 : TAFVAPSS---RVFGRGELVIDPAQIAKRYL--QQYFIVDFLSVLPLPQ- : 196
BoCNGC6  : TAFVAPSS---RVFGRGELVIDPAQIAKRYL--QQYFIVDFLSVLPLPQ- : 222
BrCNGC13 : TAYIAPSS---RVFGRGELVIDSRKIAWRYL--NKSFWIHLVAALPLPQ- : 190
BoCNGC9  : TAYIAPSS---RVFGRGELVIDSRKIAWRYL--NKSFWIHLVAALPLPQ- : 190
BrCNGC14 : TAFVARSS---RVFGRGELVRDPREIAMKYL--KSDFIVDVAAMLPLPQ- : 155
BoCNGC13 : TAFVARSS---RVFGRGELVRDPREIAMKYL--KSDFIVDVAAMLPLPQ- : 155
BrCNGC15 : TAFVSKSS---RVFGRGELVIDRREIAIRYL--KSEFIIDLAATLPLPQ- : 161
BoCNGC14 : TAFVSKSS---RVFGRGELVMDRREIAIRYL--KSEFIIDLAATLPLPQ- : 161
BrCNGC16 : TGFIAPNSST-RVFGRGELVMDPKAIAWRYL--KSDFIIDLVATLPLPQ- : 194
BoCNGC10 : TGFIAPNSST-RVFGRGELVMDPKAIAWRYL--KSDFIIDLVATLPLPQ- : 194
BrCNGC17 : TAYVSRTS---RVFGRGELVKDPKLIARRYL--RSDFIVDLIACLPLPQ- : 195
BoCNGC12 : TAYVSRTS---RVFGRGELVKDPKLIARRYL--RSDLIVDLIACLPLPQ- : 195
BrCNGC18 : TAYVSRTS---RVFGRGELVKDPKLIARRYL--RSDFIVDLIACLPLPQ- : 191
BoCNGC11 : TAYVSRTS---RVFGRGELVKDPKLIARRYL--RSDFIVDLIACLPLPQ- : 191
BrCNGC22 : LAYTDPKS---TVVGSGQLVKGSVEIAKRYY--RGNFLLDLVIVIPLPQ- : 226
BoCNGC19 : ------------------------------------------------- :   -
BrCNGC27 : LAYVDLES---TVAGAGQLVDDPKKIASHYL--RGKFLTDFFIVLPIPQ- : 266
BoCNGC18 : LAYVDLES---TVVGAGQLVDDPKKIASHYL--RGKFLTDFFIVLPIPQ- : 260
BrCNGC28 : LAYVAPES---TVVGAGQLVAHPRKIARHYF--RGKFLLDLFIVMPLPQ- : 305
BoCNGC21 : LAYVAPES---TVVGAGQLVAHPRKIARHYF--RGKFLLDLFIVMPLPQ- : 304
BoCNGC20 : LAYVAPES---TVVGAGQLVDHPRKIASHYF--RGKFLLDLFIVMPLPQ- : 299
BoCNGC22 : LAYATLES---TVVGVGHLFDHPRKIARNYL--RVKFLLDLFIVMPLPQ- : 295
BrCNGC23 : LAYVSPES---MVVGVTWLVDHPVKIARHYF--QGNFFLDLFIVMPLPQ- : 312
BrCNGC24 : LAYVARES---TVVGAGQLV----------------------------- : 245
BoCNGC23 : LAYVARES---TVVGAGQLVDRPKKIALHYF--RGYFIVDLIIVMPFPQ- : 302
BrCNGC25 : MAYVAPES---RIVGAGQLVDHPRKIARNYL--RGKFFLDLFIVLPIPQ- : 284
BoCNGC24 : MAYVAPES---RIVGAGQLVDHPRKIARNYL--RGKFFLDLFIVLPIPQ- : 284
BrCNGC26 : LAYVAPES---RIVGAGQLVDHPRKIACNYL--RGKFFLDVVIVYPVPQ- : 301
BoCNGC26 : LAYVAPES---RIVGAGQLVDHPRKIACNYL--RGKFFLDLLIVYPVPQ- : 297
BrCNGC29 : LAYVAPES---RIVGAGQLVDHPRKIASNYL--RGKFLLDLLIVLPMPQ- : 298
BoCNGC25 : LAYVAPES---RIVGAGQLVDHPRKIASKYF--RGKFFLDLLIVVPMPQ- : 301
BrCNGC19 : WPYRGGDSDGDINKGDGTRVRM--RGAPPYVKKNG-FFFDLFVILPLPQ- : 202
BoCNGC15 : WPYPSGDSDGDINKGDGTRVRT--RVAPPYVKKNG-FFFDLFVILPLPQ- : 202
BrCNGC20 : WPYPGGDSDGDTNKGDETRLRTSRRVAPPYVKKKGTFFFDLFVILPLPQ- : 205
BoCNGC16 : WPYPGGDSDGDTNKGDETRLRTSRRVAPPYVKKKGTFFFDLFVILPLPQ- : 205
BrCNGC21 : --YVSRES---LVVGCGKLVWDPRAIASHYARSLTGFWFDVIVILPVPQ- : 227
BoCNGC17 : --YVSRES---LVVGCGKLVWDPRAIASHYARSLTGFWFDVIVILPVPQ- : 226
                   s      g g l      a  y     f  d     p pq

                      *        420        *        440       *
BrCNGC1  : ---------------------------VVVLILIH-RSD---SLVSQAIL : 158
BoCNGC3  : ---------------------------VVVLILIH-RSD---SLVSQAIL : 158
BrCNGC2  : ---------------------------VMVLTLLS-RKQK-TSLVSKEIL : 201
BrCNGC4  : ------------------------------------------------- :   -
BrCNGC3  : ---------------------------VVVLAVVP-SMSRPASLVTKELL : 208
BoCNGC2  : ---------------------------VVVLAVVP-SMGRPASLVTKELL : 208
BrCNGC5  : ---------------------------LVVLAVIP-NVDKPVSLLTKDYL : 213
```

```
BoCNGC1  : ---------------------------LVVLAVIP-NVEKPVSLLTKDYL : 214
BrCNGC6  : --------------------------QMVISIIIP-RMREPKTLHTKNML : 249
BrCNGC7  : RIEIEIEIDLVLSTCISKPLMIVCFPQVVILIIIP-HMRGSRSLNTKNLL : 245
BrCNGC8  : ---------------------------IAVWKFLH-GSKGMDVLPTKTAL : 199
BoCNGC8  : ---------------------------IAVWKFLH-GSKGMDVLPTKTAL : 228
BrCNGC9  : ---------------------------IVVWRFLQ-SSRGSDVLATKQAL : 255
BoCNGC7  : ---------------------------IVVWRFLQ-RSRGSDVLATKQAL : 227
BrCNGC10 : ---------------------------ITVWRFLY-TSKGANVLATKQAL : 240
BoCNGC5  : ---------------------------ITVWRFLY-TSKGANVLATKQAL : 240
BrCNGC11 : ---------------------------IIVWRFLY-SSRGANVLATKQAL : 230
BoCNGC4  : ---------------------------IIVWRFLY-SSRGANVLATKQAL : 230
BrCNGC12 : ---------------------------IVVWRFLY-TSTGGSVLETKQAL : 218
BoCNGC6  : ---------------------------IVVWRFLY-TSTGGSVLETKQAL : 244
BrCNGC13 : ---------------------------VLIWIVIP-NLRGSPMTNTKNTL : 212
BoCNGC9  : ---------------------------VLIWIVIP-NLRGSPMTNTKNTL : 212
BrCNGC14 : ---------------------------LVIWLVIP-AATNGTANHANSTL : 177
BoCNGC13 : ---------------------------LVIWLVIP-AATNGTANHANSTL : 177
BrCNGC15 : ---------------------------IMIWFVIPNAGEFRYAAHQNHTL : 184
BoCNGC14 : ---------------------------IMIWFVIPNAGEFRYAAHQNHTL : 184
BrCNGC16 : ---------------------------IVIWFVMP-ATRSYRFDHSNNDL : 216
BoCNGC10 : ---------------------------IVIWFVMP-ATRSYRFDHSNNDL : 216
BrCNGC17 : ---------------------------IVSWFILP-SIRSSHSDHTTNAL : 217
BoCNGC12 : ---------------------------IVSWFILP-SIRSSHSDHTTNAL : 217
BrCNGC18 : ---------------------------IVSWFILP-SIRSSHSDHTTNAL : 213
BoCNGC11 : ---------------------------IVSWFILP-SIRSSHSDHTTNAL : 213
BrCNGC22 : ---------------------------ILILWIIPHVLVIYGANTIKNLL : 249
BoCNGC19 : -------------------------------------------------- :   -
BrCNGC27 : ---------------------------ILLLWIIPQLLGTSGANNTKNYL : 289
BoCNGC18 : ---------------------------ILLLWIIPQLLGTSGANNTKNYL : 283
BrCNGC28 : ---------------------------ILILWIIPAHLGASGANYAKNLL : 328
BoCNGC21 : ---------------------------ILILWIIPAHLGASGANYAKNLL : 327
BoCNGC20 : ---------------------------ILILWIIPAHLAISGANYAKNLL : 322
BoCNGC22 : ---------------------------IWIFWILPVQLGASGDNYAKNFL : 318
BrCNGC23 : ---------------------------ILISWITPAWLGGSWENNAYSLL : 335
BrCNGC24 : ----------------------------LVLSVIPAQLAISGTNYAKNLL : 267
BoCNGC23 : ---------------------------VLVLSVIPAQLAISGTNYAKNLL : 325
BrCNGC25 : ---------------------------IMTLSILPAHLGTSTAGFERNII : 307
BoCNGC24 : ---------------------------IMTLSILPAHLGTSTAGFERNIT : 307
BrCNGC26 : ---------------------------LMILSILP------KSEFEENAI : 318
BoCNGC26 : ---------------------------IMIFSILP------KSEFEENTV : 314
BrCNGC29 : ---------------------------IMILSMLPAHLGTPRSELEKNII : 321
BoCNGC25 : ---------------------------IMILSMLPAHLGTPRSELEKNII : 324
BrCNGC19 : ---------------------------VVLWVVIPSLLKRGSVTLVVSIL : 225
BoCNGC15 : ---------------------------VVLWVVIPSLLKRGSVTLVVSIL : 225
BrCNGC20 : ---------------------------VVLWVVIPSLLKRGSVTLVVSVL : 228
BoCNGC16 : ---------------------------VVLWVVIPSLLKRGSVTLVVSVL : 228
BrCNGC21 : ---------------------------AVFWLVVPKLIREEKVKLIMTIL : 250
BoCNGC17 : ---------------------------AVFWLVVPKLIREEKVKLIMTIL : 249
```

```
                    460         *        480         *        500
BrCNGC1  : KWIILTQYIPRIIRIYPLLKEVTRASGTIAETKWVGAAFNLFLYMLHSHV : 208
BoCNGC3  : KWIILTQYVPRIIRIYPLLKEVTRASGTIAETKWVGAAFNLFLYMLHSHV : 208
BrCNGC2  : KWAMFCQSIPRSIRIYPIYKNGTKLYGRVAVTKWVGAALNLFFYLLPSHV : 251
BrCNGC4  : ------------------------------------------------QV : 112
BrCNGC3  : KWAIFCQYVPRIARIYPLFKEVTRTSGLVTETAWAGAALNLFLYMLASHV : 258
BoCNGC2  : KWVIFCQYVPRIARIYPLFKEVTRTSGLVTETAWAGAALNLFLYMLASHV : 258
BrCNGC5  : ITVIFAQYIPRILRIYPLYSEVTRTSGIVTETAWAGAAWNLSLYMLASHV : 263
BoCNGC1  : ITVIFAQYIPRILRIYPLYSEVTRTSGIVTETAWAGAAWNLSLYMLASHV : 264
BrCNGC6  : KFIVFFQYIPRFMRIYPLYKQVTRTSGILTETAWAGAAFNLFLYMLASHV : 299
BrCNGC7  : KFIVFFQYIPRFIRIYPLYKEVTRTSGILTETAWAGAAFNLFLYMLASHV : 295
BrCNGC8  : LNIVITQYIPRFVRFIPLTSELKKTAGAFAEGAWAGAAYYLLWYMLASHI : 249
BoCNGC8  : LNIVITQYIPRFVRFIPLTSELKKTAGAFAEGAWAGAAYYLLWYMLASHI : 278
BrCNGC9  : LFIVLVQYIPRFLRVLPLTSELKRTAGVFAETAWAGAAYYLMLYMLASHI : 305
BoCNGC7  : LFIVLVQYIPRFLRVLPLTSELKRTAGVFAETAWAGAAYYLMLYMLASHI : 277
BrCNGC10 : RYIVLVQYIPRFVRMYPLSSELKRTAGVFAETAWAGAAYYLLLYMLASHI : 290
BoCNGC5  : RYIVLVQYIPRFLRMYPLSSELKRTAGVFAETAWAGAAYYLLLYMLASHI : 290
BrCNGC11 : RYIVLVQYIPRFLRMYPLSSELKRTAGVFAETAWAGAAYYLLLYMLASHI : 280
BoCNGC4  : RYIVLVQYIPRFLRMYPLSSELKRTAGVFAETAWAGAAYYLLLYMLASHI : 280
BrCNGC12 : RSIILVQYIPRFIRIYPLSKELKRTAGVFAETAWAGAAYYLLLYMLASHI : 268
BoCNGC6  : RSIILVQYIPRFFRIYPLSKELKRTAGVFAETAWAGAAYYLLLYMLASHI : 294
BrCNGC13 : RFIIIFQYVPRMFLIFPLSRQIIKATGVVTETAWAGAAYNLMLYMLASHV : 262
BoCNGC9  : RFIIIFQYVPRMFLIFPLSRQIIKATGVVTETAWAGAAYNLMLYMLASHV : 262
BrCNGC14 : ALIVLVQYIPRSFIIFPLNQRIIKTTGFIAKTAWAGAAYNLLLYILASHV : 227
BoCNGC13 : ALIVLVQYIPRSFIIFPLNQRIIKTTGFIAKTAWAGAAYNLLLYILASHV : 227
BrCNGC15 : SLVVLIQYVPRILVMLPLNRRIIKATGVAAKTAWSGAAYNLVLYLLVSHV : 234
BoCNGC14 : SLVVLIQYVPRILVMLPLNRRIIKATGVAAKTAWSGAAYNLVLYLLVSHV : 234
BrCNGC16 : ALIVLLQYIPRFYLIIPLSSQIVKATGVVTKTAWAGAAYNLLLYMLASHV : 266
BoCNGC10 : ALIVLLQYIPRFYLIIPLSSQIVKATGVVTKTAWAGAAYNLLLYMLASHV : 266
BrCNGC17 : VLIVLVQYIPRLYLIFPLSAEIIKATGVVTTTAWAGAAYNLLQYMLASHI : 267
BoCNGC12 : VLIVLVQYIPRLYLIFPLSAEIIKATGVVTTTAWAGAAYNLLQYMLASHI : 267
BrCNGC18 : VLIVLVQYIPRLYLIFPLSAEIIKATGVVTTTAWAGAAYNLLQYMLASHI : 263
BoCNGC11 : VLIVLVQYIPRLYLIFPLSAEIIKATGVVTTTAWAGAAYNLLQYMLASHI : 263
BrCNGC22 : CTAVVVQCIPKLHRLFHLLARKR-TTGFIFESAWASFFINLFTYILAGHI : 298
BoCNGC19 : -------------------------------------------------- :   -
BrCNGC27 : RAAILVQYIPKLRRLFPLLAGQT-PRGFRFDSALAKFFINLLTFMLAGHV : 338
BoCNGC18 : RAAILVQYIPKLRRLFPLLAGQT-PRGFRFDSALAKFFINLLTFMLAGHV : 332
BrCNGC28 : RAAVLFQYIPKLYRLLPLLAGQT-PTGFIFESAWANFVINLLTFMLAGHV : 377
BoCNGC21 : RAAVLFQYIPKLYRLLPLLAGQT-PTGFIFESAWANFVINLLTFMLAGHV : 376
BoCNGC20 : RAAVLFQYIPKLYRLLPLLAGQT-PTGFIFESAWANFFINLLTFMLAGHV : 371
BoCNGC22 : TAAVLFQYIPKLCRLLPLLAGKT-PTVFLFESACS-FVTNILTFMLVGHV : 366
BrCNGC23 : QAAVLLQYTLKLYRLLPLLAGKT-PIGFILESSSK-FAINFLTFMLAGHV : 383
BrCNGC24 : RTGILVQYIPKLYRLLPLLAGQT-PTGLIFESAWANFVINLLTFMLAGHV : 316
BoCNGC23 : RAGILVQYIPKLYRLLPLLAGQT-PTGLIFESAWANFVINLLTFMLAGHV : 374
BrCNGC25 : RSTFIVQYIPKLYRLLPLLAGQT-PTGFIFESAWASFVINLLTFMLAGHA : 356
BoCNGC24 : RSIFIVQYIPKLYRLLPLLAGQT-PTGFIFESAWASFVINLLTFMLAGHA : 356
BrCNGC26 : IGIFLFQYIPKLYRLLPLIAGQT-PTGFIFESAWSNFIINLLTFILAGHA : 367
BoCNGC26 : IALFLFQYIPKLYRLLPLIAGQT-PTGFIFESAWSNFIINLLTFILAGHA : 363
```

```
BrCNGC29 : RIVFLFQYIPKLYRLLPLLAGQT-PTGFIFESAWANFIINLLTFILAGHA : 370
BoCNGC25 : RIVFLFQYIPKLYRLLPLLAGQT-PTGFIFESAWANFIINLLTFILAGHA : 373
BrCNGC19 : LLTFLFQYLPKIYHSIRHLRQNATLSGYIFGTVWWGFALNMVAYFVAAHA : 275
BoCNGC15 : LLTFLFQYLPKIYHSIRHLRRNATLSGYIFGTVWWGIALNMVAYFVAAHA : 275
BrCNGC20 : LVTFLFQYLPKIYHSVRHLRQNATLSGYIFGTVWWGIALNMIAYFVAAHA : 278
BoCNGC16 : LVTFLFQYLPKIYHSVRHLRQNATLSGYIFGTVWWGIALNMIAYFVAAHA : 278
BrCNGC21 : LLIFLFQFLPKIYHCICLMRRMQKVTGYIFGTIWWGFALNLIAYFIASHV : 300
BoCNGC17 : LLIFLFQFLPKIYHCICLMRRMQKVTGYIFGTIWWGFALNLIAYFIASHV : 299
                q p     p         g     w           a h

                   *          520         *         540          *
BrCNGC1  : FGAFWYVSSVEKKNKCWRLECAKI------SGCNLR--HQYCARGRE-N- : 248
BoCNGC3  : FGAFWYVSSVEKKNKCWRLECAKI------FGCNLR--YQYCARGRQ-N- : 248
BrCNGC2  : IGAIWYLSAVEKKETCWREACAKI------DECDLT--NLLCARGAGGD- : 292
BrCNGC4  : FGAFWYLSAIEKKNRCWSDACAKS------SMCNLTNLDLYCVRGGG-D- : 154
BrCNGC3  : FGSFWYLISIERKDRCWRETCAKI------EGCVHG--NLYCSGGED--- : 297
BoCNGC2  : FGSFWYLISIERKDRCWRETCAKI------KGCIHA--YLYCSGGED--- : 297
BrCNGC5  : FGALWYLISVEREDRCWREACGKR------QGCELR--DLYCDGNNN-V- : 303
BoCNGC1  : FGALWYLISVEREDRCWREACEKR------QGCELR--FLYCDGNNN-V- : 304
BrCNGC6  : FGAFWYLFSIERKTVCWKQAWNRAG----RSKCDMR--SLYCAREHY-G- : 341
BrCNGC7  : FGAFWYLFSIERETVCWKQACNRN-----RNICDIT--SLYCDHKAA-G- : 336
BrCNGC8  : TGAFWYMLSVERNDTCWRFACKVQP----DPKLCVQ--ILYCGTKFVSSR : 293
BoCNGC8  : TGAFWYMLSVERNDTCWRFACKVQP----DPKLCVQ--ILYCGTKFVSSR : 322
BrCNGC9  : VGAFWYLLALERNDACWQEACSDAG----KKICTTG--FLYCGNQNMDG- : 348
BoCNGC7  : VGAFWYLLALERNDACWQEACSDAG----KKICTTG--FLYCGNQNMDG- : 320
BrCNGC10 : VGALWYLLALERNNDCWSKACVKK------DNCTRN--FLFCGNQNMEG- : 331
BoCNGC5  : VGALWYLLALERNNDCWSKACVKK------DNCTRN--FLFCGNQNMEG- : 331
BrCNGC11 : VGALWYLLALERNNDCWSKACKDN------DNCTRN--FLFCGNQNMKG- : 321
BoCNGC4  : VGALWYLLALERNNDCWSKACKDN------DNCTRN--FLFCGNQNMKG- : 321
BrCNGC12 : VGALWYLLALERVNGCWKKACLVDG-----QNCTRN--FLFCGNENMDG- : 310
BoCNGC6  : VGALWYLLALERVNGCWKRACLLDG-----QNCTRN--YLFCGNENMDG- : 336
BrCNGC13 : LGACWYLLAVERQEACWRHACNIEK-----PICQYR--FFECR-RLEDP- : 303
BoCNGC9  : LGACWYLLAVERQEACWRHACNIEK-----TICQYR--FFECR-RLEDP- : 303
BrCNGC14 : LGAMWYLSSIGRQFSCWSKVCEKDH-ALRVLDCLPS--FLDCK-SLEQP- : 272
BoCNGC13 : LGAMWYLSSIGRQFSCWSKVCEKDH-ALRVLDCLPS--FLDCK-SLQQP- : 272
BrCNGC15 : LGSVWYVLSIQRQHECWRRECIKEMNATHSPSCNLL--FLDCG-SLRDP- : 280
BoCNGC14 : LGSVWYVLSIQRQHECWRRECIKEMNATHSPSCNLL--FLDCG-SLRDP- : 280
BrCNGC16 : LGAAWYILSFDRYTSCWKTRCNKEH---GGVNCYLY--YLDCDSPLYDA- : 310
BoCNGC10 : LGAAWYILSFDRYTSCWKTRCNKEH---GGVNCYLY--YLDCDSPLYDA- : 310
BrCNGC17 : LGAAWYLLSIERQATCWKAECHNEL---GPIRCVTD--FFDCG-TVNRE- : 310
BoCNGC12 : LGAAWYLLSIERQATCWKAECHNEL---GPIRCVTD--FFDCG-TVNRE- : 310
BrCNGC18 : LGAAWYLLSIQRQATCWKAECHKEF---APLECVTD--FFDCG-TLHRP- : 306
BoCNGC11 : LGAAWYLLSIQRQATCWKAECHKEF---APLECVTD--FFDCG-TLHRP- : 306
BrCNGC22 : VGSCWYLFGLQRVNRCLRETCGDHM---ESKCHGVCKELIDCGLRLKEK- : 344
BoCNGC19 : -----------RVNRCLRETCEDHM---ELKCHGVCKELIDCGLHLKEK- : 247
BrCNGC27 : IGSCWYLLGLQRVNQCLRDACGN--------SSFECKQLIDCG------- : 373
BoCNGC18 : IGSCWYLLGLQRVNQCLRDACGN--------SSFECKQLIDCG------- : 367
BrCNGC28 : VGSCWYLFGLQRVNQCLRDACGN--------SDHECRNLIDCG------- : 412
BoCNGC21 : VGSCWYLFGLQRVNQCLRDACGN--------SDHECRNLIDCG------- : 411
```

```
BoCNGC20 : VGSCWYLFGLQRVNQCLRDACGN--------TDRPCRELIDC-------- : 405
BoCNGC22 : VGSCWYLFGLQRVNHCLRDACGN--------SHQECRELIYCG------- : 401
BrCNGC23 : VGSCWYLFGLQRVNQCLRDACGN--------TDRACRELIDCG------- : 418
BrCNGC24 : VGSCWYLFGLQRVNQCLRNAC-G-------HSGRECRGLIDCGHG----- : 353
BoCNGC23 : VGSCWYLFGLQRVNQCLRNAC-G-------HSGRECRGLIDCGHG----- : 411
BrCNGC25 : VGSCWYFSGIERVKKCLLYAAWN-------NSVDERRNLIDCARG----- : 394
BoCNGC24 : VGSCWYFSGIERVKKCLLYAAWN-------NSVDERRNLIDCARG----- : 394
BrCNGC26 : VGSFWYIAGLQRVNKCLLQAG-N-------FTMDERRYLIDCTSG----- : 404
BoCNGC26 : VGSFWYIAGLQRVKKCLLQAG-N-------FTMDERRYIIDCTSG----- : 400
BrCNGC29 : IGSFWYIAGLQRVKKCLLHAG-N-------YTMDERRNLIDCAHG----- : 407
BoCNGC25 : IGSFWYIAGLQRVKKCLLHAG-N-------YTMDERRNLIDCARG----- : 410
BrCNGC19 : AGACWYLLGVQRSAKCLKEQCENTMG--CDLRMLSCKEPVYYGTTEMVL- : 322
BoCNGC15 : AGACWYLLGVQRSAKCLKEQCENTMG--CDLRMLSCKEPVYYGTTEMVL- : 322
BrCNGC20 : AGACWYLLGVQRSAKCLTEQCESTMG--CDLRMLSCKEPVYYGTTEMVL- : 325
BoCNGC16 : AGACWYLLGVQRSAKCLKEQCESTMG--CDLRMLSCKEPVYYGTTEMVL- : 325
BrCNGC21 : AGGCWYVLAIQRVASCIRQQCMRTAN--CNL-SLSCQEEVCYQFVSPSS- : 346
BoCNGC17 : AGGCWYVLAIQRVASCIRQQCMRTAN--CNL-SLSCQEEVCYQFVSPTS- : 345
             g  wy     4   C    c                    c
```

```
                    560         *         580         *         600
BrCNGC1  : -------NGRYLNTTCPLIDPDQIIG-STVFNFGMYTDALRSGIVESKPR : 290
BoCNGC3  : -------NGRYLNTTCPLIDPDQIIG-STVFNFGMYTDALRSGIVESKPR : 290
BrCNGC2  : -------NSRFLNTSCPLIDPEQITN-STVLNFGIYTDALKSGVVET--R : 332
BrCNGC4  : -------NSHFLKISCPLIDPGEITN-STVFNFGMYIDALKSGVVES--R : 194
BrCNGC3  : -------NSQYLIGSCPLMDPEEIKN-STVFNFGIFAEALQSGVVES--M : 337
BoCNGC2  : -------NSQYLIGSCPLMDPEEIKN-STVFNFGIFAEALQSGVVES--M : 337
BrCNGC5  : -------INDYLTTSCPFINPDDITN-STTFNFGIFTDALKSGIVKS--D : 343
BoCNGC1  : -------INDYLTTSCPFINPDDITN-STTFNFGIFTDALKSGIVES--D : 344
BrCNGC6  : -------NNTFLNGSCPVLKP----N-ATCFEFGIFLGALESGVVES--H : 377
BrCNGC7  : -------GNAFLNASCPVQTP----N-ATLFDFGIFLNALQSGVVES--Q : 372
BrCNGC8  : ETEWIKTVPELLKSNCSAKADD------AKFNYGIYGQAISSGIVSS--T : 335
BoCNGC8  : ETEWIKTVPELLKSNCSAKADD------AKFNYGIYGQAISSGIVSS--T : 364
BrCNGC9  : YDVWNKTKEAVLESRCRADLDD---P-NPPFDFGIYTQALSSGIVSS--Q : 392
BoCNGC7  : YDVWNKTKESVLQSKCRAELDD---P-NPPFDFGIYTQALSSGIVSS--Q : 364
BrCNGC10 : YAAWYTAKSSVLQEMCPVNVTEG--E-EPPFDFGIYSRALSSGIVSS--K : 376
BoCNGC5  : YAAWYTAKSSVLQEKCPVNVTEG--E-EPPFDFGIYSRALSSGIVSS--K : 376
BrCNGC11 : YDAWDDVKDPFLQLRCPVNVTEG--E-EPPFDFGIYLRALSSGIVSS--K : 366
BoCNGC4  : YDAWDDVKDPFLQLRCPVNVTDG--E-EPPFDFGIYLRALSSGIVSS--K : 366
BrCNGC12 : YAAWNTIKESVLQKSCPVNVTDG--D-NPPFDFGIYLRALSSGIVSS--E : 355
BoCNGC6  : YAAWNTIKESVLQKSCPVNVTDG--D-NPPFDFGIYLRALSSGIVSS--E : 381
BrCNGC13 : ------QRNSWFEWSNITTICKPGTR---FYEFGIYGDAVTSTVTSS--- : 341
BoCNGC9  : ------QRNSWFEWSNITTICKPGTR---FYEFGIYGDAVTSTVTSS--- : 341
BrCNGC14 : ------ERQYWQNVTQVLSHCDATSS-TTNFKFGMFAEAFTTQVATT--- : 312
BoCNGC13 : ------ERQYWQNVTQVLSHCDATSS-TTNFKFGMFAEAFTTQVATT--- : 312
BrCNGC15 : ------GRQAWMRITRVLSNCDARNDDDQHFQFGMFGDAFTNDVTSS--- : 321
BoCNGC14 : ------GRQAWMRITRVLSNCDARNDDDQHFQFGMFGDAFTNDVTSS--- : 321
BrCNGC16 : ------RQQQWANVTNVFKLCDAR-K--GEFKYGMFENAITKKVVSS--- : 348
BoCNGC10 : ------RQQQWANVTNVFKLCDAR-K--GEFKYGMFENAITKKVVSS--- : 348
BrCNGC17 : ------DRNNWQNVTVVFSNCDPSNK--IRFTFGIFADALTKNVVSS--- : 349
```

```
BoCNGC12 : ------DRNNWQNVTVVFSNCDPSNK--IRFTFGIFADALTKNVVSS--- : 349
BrCNGC18 : ------DRNNWQNITVVFSNCDPSND--IKFTFGIFADALTKNVVSS--- : 345
BoCNGC11 : ------DRNNWQNITVVFSNCDPSND--IKFTFGIFADALTKNVVSS--- : 345
BrCNGC22 : LIDCERRNTTVTQAVLNWNVTAAACFQENGFPYGIYWKAVNLTHDSN--- : 391
BoCNGC19 : LIDCERRNTTVTQAVLNWNVTAAACFQENGFRYGIYWKAVNLTHDTN--- : 294
BrCNGC27 : -----RENRTEVLHAWKINVSANACFQEDGFDYGIYLKAVNLTSNRTR-- : 416
BoCNGC18 : -----RENRTEVLHAWKINVSANACFQEDGFDYGIYLKAVNLTSNCTR-- : 410
BrCNGC28 : -----RGESSEAFAAWKGNASASACFQEGGFPYGIYMKAVNLTNHTS--- : 454
BoCNGC21 : -----RGDSSEAFAAWKGNASASACFQDGGFPYGIYMKAVNLTSHTS--- : 453
BoCNGC20 : -----HGETSALSAAWKDNAGASACFQEDGFPYGIYLKAVNLTNHNS--- : 447
BoCNGC22 : -----HGNS--HVAAWKDNASAIACFQEDGFPYGIYLKAVNLTNHSS--- : 441
BrCNGC23 : -----RGSSDVVLAALKYNTSASACFQENGFPYGIYLKAVNLTNQSS--- : 460
BrCNGC24 : ----NSNISASLRAIWRNSASANACFQEDGFSYGIYLKMVNLTTHTS--- : 396
BoCNGC23 : ----NSNISASLRAIWRNSASANACFQEDGFSYGIYLKMVNLTTHTS--- : 454
BrCNGC25 : ----NIYASASLRARWRDSDSVNACFQESGFSYGIYAKAVNLTSHSS--- : 437
BoCNGC24 : ----NMYASASLRALWRDSDSVNACFQESGFSYGIYAKAVNLTSHSS--- : 437
BrCNGC26 : ----ESYLR----ALWRDSESVNACFQDGGFSFGIYLNAANLTTQRN--- : 443
BoCNGC26 : ----DSNLR----ALWRDSESVNACFQESGFSFGIYLNAANLTTEPN--- : 439
BrCNGC29 : ----VNYARESQIALWRDSESVNACFQEGGFSFGIYLKAVNLTTQPN--- : 450
BoCNGC25 : ----VNYARESQIALWRESESVNACFQEGGFSFGIYLKAVNLTTQPN--- : 453
BrCNGC19 : ----DRARLAWARNHQ---ARSVCLDIDTDYTYGAYQWTIQLVSNES--- : 362
BoCNGC15 : ----DRARLAWARNHQ---ARSVCLDSDTNYTYGAYQWTIQLVSNES--- : 362
BrCNGC20 : ----DRARLAWAQNNQ---ARSICLDINTNYTYGAYKWTIQLVSNES--- : 365
BoCNGC16 : ----DRARLAWAQNNQ---ARSICLDINTNYTYGAYKWTIQLVSNES--- : 365
BrCNGC21 : ----TIGFPCVSGNLTSVVKKPMCLDSDGPFRYGIYGWALPVISSNS--- : 389
BoCNGC17 : ----TIGFPCLSGNLTSVVKKPMCLDSNGPFRYGIYGWALPVISSNS--- : 388
                                            5G 5  a

                    *        620       *       640         *
BrCNGC1  : DFPRKFFYCFW--------------------------------------- : 301
BoCNGC3  : DFPRKFFYCFW--------------------------------------- : 301
BrCNGC2  : DFPRKLLYCFW--------------------------------------- : 343
BrCNGC4  : DFPRKFFYCFW--------------------------------------- : 205
BrCNGC3  : NFPKKFFYCFW--------------------------------------- : 348
BoCNGC2  : DFPKKFFYCFW--------------------------------------- : 348
BrCNGC5  : DFWKKFFYCFW--------------------------------------- : 354
BoCNGC1  : DFWKKFFYCFW--------------------------------------- : 355
BrCNGC6  : DFPQKFFYCFW--------------------------------------- : 388
BrCNGC7  : DFPQKFFYCFW--------------------------------------- : 383
BrCNGC8  : TFFSKFCYCLW--------------------------------------- : 346
BoCNGC8  : TFFSKFCYCLW--------------------------------------- : 375
BrCNGC9  : KFITKYCYCLW--------------------------------------- : 403
BoCNGC7  : KFITKYCYCLW--------------------------------------- : 375
BrCNGC10 : KFVSKYFFCLW--------------------------------------- : 387
BoCNGC5  : KFVSKYFFCLW--------------------------------------- : 387
BrCNGC11 : KFVSKYFFCLW--------------------------------------- : 377
BoCNGC4  : KFVSKYFFCLW--------------------------------------- : 377
BrCNGC12 : SFVAKYFFCLW--------------------------------------- : 366
BoCNGC6  : SFVAKYFFCLW--------------------------------------- : 392
```

```
BrCNGC13 : NFISKYFYCLW--------------------------------------- : 352
BoCNGC9  : NFINKYFYCLW--------------------------------------- : 352
BrCNGC14 : DFVSKYLYCLW--------------------------------------- : 323
BoCNGC13 : DFVSN--------------------------------------------- : 317
BrCNGC15 : PFFDKYFYCLW--------------------------------------- : 332
BoCNGC14 : PFFDKYFYCLW--------------------------------------- : 332
BrCNGC16 : NFNERYFYCLW--------------------------------------- : 359
BoCNGC10 : NFNERYFYCLW--------------------------------------- : 359
BrCNGC17 : PFLEKYLYCLW--------------------------------------- : 360
BoCNGC12 : PFLEKYLYCLW--------------------------------------- : 360
BrCNGC18 : PFLEKYLYCLW--------------------------------------- : 356
BoCNGC11 : PFLEKYLYCLW--------------------------------------- : 356
BrCNGC22 : -LPKKYIYSLF--------------------------------------- : 401
BoCNGC19 : -LPEKYIYSLF--------------------------------------- : 304
BrCNGC27 : -WYRRYSYSLF--------------------------------------- : 426
BoCNGC18 : -WYRRYSYSLF--------------------------------------- : 420
BrCNGC28 : -LFTRYSYSLF--------------------------------------- : 464
BoCNGC21 : -LFTRYSYSLF--------------------------------------- : 463
BoCNGC20 : -LFTRYSYSLF--------------------------------------- : 457
BoCNGC22 : -LFTRYIYSLL--------------------------------------- : 451
BrCNGC23 : -LITIHKIHLLSFLGFSANLIRMFYGFDIKSALVGICGSLTLAYCQLSIV : 509
BrCNGC24 : -LLTRYSYSLL--------------------------------------- : 406
BoCNGC23 : -LLTRYSYSLL--------------------------------------- : 464
BrCNGC25 : -IFTRFSYSLF--------------------------------------- : 447
BoCNGC24 : -IFTRFSYSLF--------------------------------------- : 447
BrCNGC26 : -IFTRFSFSLF--------------------------------------- : 453
BoCNGC26 : -IFTRFSFSLF--------------------------------------- : 449
BrCNGC29 : -IFTRFSYSLF--------------------------------------- : 460
BoCNGC25 : -IFTRFSYSLF--------------------------------------- : 463
BrCNGC19 : -RLEKVLFPIF--------------------------------------- : 372
BoCNGC15 : -RLEKILFPIF--------------------------------------- : 372
BrCNGC20 : -RLEKILFPIF--------------------------------------- : 375
BoCNGC16 : -RLEKILFPIF--------------------------------------- : 375
BrCNGC21 : -LAVKILYPIF--------------------------------------- : 399
BoCNGC17 : -LAVKILYPIF--------------------------------------- : 398


                  660         *       680         *       700
BrCNGC1  : -WGLRNISALGQNLKTSNSVGDIVFALIICVSGLLLFAVLIGNIQKYLQS : 350
BoCNGC3  : -WGLRNISALGQNLKTSNSVGDIVFALIICVSGLLLFAVLIGNIQKYLQS : 350
BrCNGC2  : -WGLRNISALGQNLKTSNSAGEVFFAIIICVSGVLLFAGLIGNVQKYLQS : 392
BrCNGC4  : -WGLRNLSALGQNLETSNSVGEIFFAITICVSGLLLFSVLIGNVQKYLQS : 254
BrCNGC3  : -WGLRNLSAVGQNLKTSDFEGEIIFAIIICISGLVLFALLIGNMQKYLQS : 397
BoCNGC2  : -WGLRNLSALGQNLKTSAFEGEIIFAVIICISGLVLFALLIGNMQKYLQS : 397
BrCNGC5  : -WGLRNLSALGQNLNTSKFVGEIIFAVLICISGLVLFALLIGNMQKYLES : 403
BoCNGC1  : -WGLRNLSALGQNLNTSKFVGEIIFAVLICISGLVLFALLIGNMQKYLES : 404
BrCNGC6  : -WGLQNLSSLGQNLKTSTYIWENCFAVFISISGLVLFSFLIGNMQTYLQS : 437
BrCNGC7  : -WGLQNLSSLGQNLKTSTYIWEICFAVFISISGLVLFSFLIGNMQTYLQS : 432
BrCNGC8  : -WGLQNLSTLGQGLQTSTFPGEVLFSIAIAIAGLLLFALLIGNMQTYLQS : 395
```

```
BoCNGC8  : -WGLQNLSTLGQGLQTSTFPGEVLFSIAIAIAGLLLFALLIGNMQTYLQS : 424
BrCNGC9  : -WGLQNLSTLGQGLETSTYPLEIMFSITLAISGLILFALLIGNMQTYLQS : 452
BoCNGC7  : -WGLQNLSTLGQGLETSTYPLEIMFSITLAISGLILFALLIGNMQTYLQS : 424
BrCNGC10 : -WGLQNLSTLGQGLETSTYPGEVIFSIALAIAGLLLFALLIGNMQTYLQS : 436
BoCNGC5  : -WGLQNLSTLGQGLETSTYPGEVIFSIALAIAGLLLFALLIGNMQTYLQS : 436
BrCNGC11 : -WGLQNLSTLGQGLETSTYPGEVIFSIVLAIAGLLLFALLIGNMQTYLQS : 426
BoCNGC4  : -WGLQNLSTLGQGLETSTYPGEVIFSIVLAIAGLLLFALLIGNMQTYLQS : 426
BrCNGC12 : -WGLQNLSTLGQGLETSTYPGEIIFSIALAVAGLLLFALLIGNMQTYLQS : 415
BoCNGC6  : -WGLQNLSTLGQGLETSTYPGEIIFSIALAVAGLLLFALLIGNMQTYLQS : 441
BrCNGC13 : -WGLKNLSSLGQNLSTSTYVGEIIFAVVMATLGLVLFALLIGNMQTYLQS : 401
BoCNGC9  : -WGLKNLSSLGQNLSTSTYVGEIIFAVVMATLGLVLFALLIGNMQTYLQS : 401
BrCNGC14 : -WGLRNLSSYGQNITTSVYLGETLFCITICIFGLILFTLLIGNMQTSLQS : 372
BoCNGC13 : --------SYGQNITTSVYLGETLFCITICIFGLILFTLLIGNMQTSLQS : 359
BrCNGC15 : -WGLRNLSSYGQSLAASTLSSETLFSCFICVAGLVFFSHLIGNVQNYLQS : 381
BoCNGC14 : -WGLRNLSSYGQSLAASTLSSETLFSCFICVAGLVFFSHLIGNVQNYLQS : 381
BrCNGC16 : -WGLQQLSSYGQNLSTTTFIGETTFAVLIAIFGLVLFAHLIGNMQTYLQS : 408
BoCNGC10 : -WGLQQLSSYGQNLSTTTFIGETTFAVLIAIFGLVLFAHLIGNMQTYLQS : 408
BrCNGC17 : -FGLQQLSSYGQNLDTSTSVLETMFAILVAIFGLVLFALLIGNMQTYLQS : 409
BoCNGC12 : -FGLQQLSSYGQNLDTSTSVLETMFAILVAIFGLVLFALLIGNMQTYLQS : 409
BrCNGC18 : -FGLQNLSSYGQNLDTSTSVLETMFAILVAIFGLVLFALLIGNMQTYLQS : 405
BoCNGC11 : -FGLQNLSSYGQNLDTSTSVLETMFAILVAIFGLVLFALLIGNMQTYLQS : 405
BrCNGC22 : -WGFQQISTLAGNLVPTDFVWEVLFTMAIIGLGLFLFALLIGNMQNFLQS : 450
BoCNGC19 : -WGFQQISTLGGNQVPSYFIWEVLFTMAIIGLGLFLFALLIGNMQNFLQS : 353
BrCNGC27 : -WGFQQISTLAGNQVPSYFFGEVLFTMCIIGLGLFLFALLIGNMQNFLQS : 475
BoCNGC18 : -WGFQQISTLAGNQVPSYFFGEVLFTMCIIGLGLFLFALLIGNMQNFLQS : 469
BrCNGC28 : -WGFQQISTLAGNQVPSYFLGEVFFTMGIIGLGLLLFALLIGNMQNFLQA : 513
BoCNGC21 : -WGFQQISTLAGNQVPSYFLGEVFFTMGIIGLGLLLFALLIGNMQNFLQA : 512
BoCNGC20 : -WGFQQISTLAGNLIPSYFLGEVFFTMGIIGLGLLLFALLIGNMQNFLQS : 506
BoCNGC22 : -WGFQQISTLAGNQVPSYFLGEASFTMVIIALGLLLFPLLIGNMQNFLQA : 500
BrCNGC23 : YYSPKQISTLAGNQVPSYFIWEVLFTMGIIGLGLLLFAFLIGNMQNFLQT : 559
BrCNGC24 : -WGFQVMVYSN--------------------------------------- : 416
BoCNGC23 : -WGFQQISTLAGNQVPSYFFPEVVFTMGIIGLGLLLFALLIGNMQNFLQS : 513
BrCNGC25 : -WGFQQISTLAGNLSPSYSVGEVFFTMGITGLGLLLFARLIGNMHNFLQA : 496
BoCNGC24 : -WGFQQISTLAGNLSPSYSVGEVFFTMGITGLGLLLFARLIGNMHNFLQA : 496
BrCNGC26 : -WGFQQISTLAGNQTPSYSVGEVYFTMGIIGLGLLLFARLIGNMHNFLQA : 502
BoCNGC26 : -WGFQQISTLAGNQTPSYSLGEVYFTMGIIGLGLLLFARLIGNMHNFLQA : 498
BrCNGC29 : -WGFQQISTLAGNQTPTYSVGEVYFTMGIIGLGLLLFARLIGNMHNFLQA : 509
BoCNGC25 : -WGFQQISTLAGNQTPTYSVGEVYFTMGIIGLGLLLFARLIGNMHNFLQA : 512
BrCNGC19 : -WGLMTLSTFGN-LESTTEWSEVVFNIIVLTSGLLLVTMLIGNIKVFLHA : 420
BoCNGC15 : -WGLMTLSTFGN-LESTTEWSEVVFNIIVLTSGLLLVTMLIGNIKVFLHA : 420
BrCNGC20 : -WGLMTLSTFGN-LESTTEWSEVVFNIIVLTSGLLLVTMLIGNIKVFLHA : 423
BoCNGC16 : -WGLMTLSTFGN-LESTTEWSEVVFNIIVLTSGLLLVTMLIGNIKVFLHA : 423
BrCNGC21 : -WGLMTLSTFGNDLEPTSNWLEVVFSIVMVLSGLLLFTLLIGNIQVFLHA : 448
BoCNGC17 : -WGLMTLSTFGNDLEPTSNWLEVVFSIVMVLSGLLLFTLLIGNIQVFLHA : 447
             g    s             e  f       g  lf  lign q  l

                    *       720         *       740         *
BrCNGC1  : TT-----------------------IRLDEMEEKKRDTEKWMSNRMLPEY : 377
BoCNGC3  : TT-----------------------IRLDEMEEKKRDTEKWMSNRMLPEY : 377
```

```
BrCNGC2  : TS-----------------------IRVDEWEAKKRDTEQWMPSEDLPDD : 419
BrCNGC4  : TI-----------------------VRINEMEEKRRDTEKWMSYRMLPES : 281
BrCNGC3  : TT-----------------------VRIEEMRVKRRDAEQWMSHRMLPDD : 424
BoCNGC2  : TT-----------------------VRVEEMRVKRRDAEQWMSHRMLPDD : 424
BrCNGC5  : TT-----------------------VREEEMRVRKRDAEQWMAHRMLPED : 430
BoCNGC1  : TT-----------------------VREEEMRVRKRDAEQWMAHRMLPED : 431
BrCNGC6  : TT-----------------------TRLEEMRVKRRDAEQWMSHRLLPDN : 464
BrCNGC7  : TT-----------------------TRLEEMRVKRRDAEQWMAHRLLPES : 459
BrCNGC8  : LT-----------------------VRLEEMRIKRRDSEQWMHHRSLPQN : 422
BoCNGC8  : LT-----------------------VRLEEMRIKRRDSEQWMHHRSLPQN : 451
BrCNGC9  : LT-----------------------IRLEEMRVKRRDSEQWMHHRMLPQD : 479
BoCNGC7  : LT-----------------------IRLEEMRVKRRDSEQWMHHRMLPQD : 451
BrCNGC10 : LT-----------------------IRLEEMRVKRRDSEQWMHHRMLPPE : 463
BoCNGC5  : LT-----------------------IRLEEMRVKRRDSEQWMHHRMLPPE : 463
BrCNGC11 : LT-----------------------IRLEEMRVKRRDSEQWMHHRMLPPE : 453
BoCNGC4  : LT-----------------------IRLEEMRVKRRDSEQWMHHRMLPPE : 453
BrCNGC12 : LT-----------------------IRLEEMRVKRRDSEQWMHHRMLPPD : 442
BoCNGC6  : LT-----------------------IRLEEMRVKRRDSEQWMHHRMLPPD : 468
BrCNGC13 : TT-----------------------MRLEEWRIRRTDTEQWMHHRQLPPE : 428
BoCNGC9  : TT-----------------------MRLEEWRIRRTDTEQWMHHRQLPPE : 428
BrCNGC14 : MS-----------------------VRVEEWRIKRRDTEEWMRHRQLPPE : 399
BoCNGC13 : MS-----------------------VRVEEWRVKRRDTEEWMRHRQLPPE : 386
BrCNGC15 : TT-----------------------ARLDEWRVRRRDTEEWMRHRQLPQE : 408
BoCNGC14 : TT-----------------------ARLDEWRVRRRDTEEWMRHRQLPQE : 408
BrCNGC16 : LT-----------------------VRLEEWRLKKRDTEEWMRHRQLPED : 435
BoCNGC10 : LT-----------------------VRLEEWRLKKRDTEEWMRHRQLPED : 435
BrCNGC17 : IT-----------------------VRLEEWRLKRRDTEEWMGHRQLPQN : 436
BoCNGC12 : IT-----------------------VRLEEWRLKRRDTEEWMGHRQLPQN : 436
BrCNGC18 : IT-----------------------VRLEEWRLKRRDTEEWMGHRQLPQD : 432
BoCNGC11 : IT-----------------------VRLEEWRLKRRDTEEWMGHRQLPQD : 432
BrCNGC22 : LG-----------------------RRNMEMTLRRRDVNNWMSHRQFPDD : 477
BoCNGC19 : LG-----------------------RRNMEMTLRRRDVNKWMSHRQFPEV : 380
BrCNGC27 : LG-----------------------RRDTEMTVRRRDVEQWMSHRRFPKD : 502
BoCNGC18 : LG-----------------------RRDTEMTVRRRDVEQWMSHRRFPKD : 496
BrCNGC28 : LG-----------------------RRNLEMTLRRRDVEQWMSHRRLPEG : 540
BoCNGC21 : LG-----------------------RRNLEMTLRRRDVEQWMSHRRLPEG : 539
BoCNGC20 : LG-----------------------RRNLEMTLRRRDVEQWMRHRRLPGD : 533
BoCNGC22 : LG-----------------------GRSLEMS-----------HKRFLEG : 516
BrCNGC23 : LG-----------------------QRNLEMTLRQRDAEQWMSHRRFPEG : 586
BrCNGC24 : ------------------------NVWSLEMTLRRRDVEQWMSHRRLPEG : 442
BoCNGC23 : LGKRMIIFGGWKHKLHWLPSKTGSDRRSLEMTLRRRDVEQWMSHRRLPEG : 563
BrCNGC25 : L-----------------------DRRRMEMMLRRRDVEQWMSHRLLPED : 523
BoCNGC24 : L-----------------------DRRRMEMMLRRRDVEQWMSHRLLPED : 523
BrCNGC26 : L-----------------------DRRRTEMMLRQRDVEQWMSHRLLPDD : 529
BoCNGC26 : L-----------------------DRRRTEMMLRQRDVEQWMSHRLLPDD : 525
BrCNGC29 : L-----------------------DRRRMEMMLRRRDVEQWMSHRLLPED : 536
BoCNGC25 : L-----------------------DRRRMEMMLRRRDVEQWMSHRLLPED : 539
BrCNGC19 : TT-----------------------SKKQAMHLKMRNIEWWMKKRHLPLG : 447
BoCNGC15 : TT-----------------------SKKQAMHLKMRNIEWWMKKRHLPFG : 447
```

```
BrCNGC20 : TT-----------------------SKKQAMHLKMRNIEWWMKKRQLPLG : 450
BoCNGC16 : TT-----------------------SKKQAMHLKMRNIEWWMKKRQLPLG : 450
BrCNGC21 : VM-----------------------AKKRKMQIRCRDMEWWMKRRQLPSR : 475
BoCNGC17 : VM-----------------------AKKRKMQIRCRDMEWWMKRRQLPSR : 474
                                        e     r  e wm h  lp

                  760         *       780         *       800
BrCNGC1  : LKERIRRYENYKWRKTRGIEEEALLHSLPKDLRLETKRHLYLTLLNSVPW : 427
BoCNGC3  : LKERIRRYENYKWRKTRGIEEEALLHSLPKDLRLETKRHLYLTLLNSVPW : 427
BrCNGC2  : LEKCI------EWEKNSSIEREAHLRSLPKDLRVEAKRNLYLYSLENVPW : 463
BrCNGC4  : LKERIRKSEDHKWRETRGTKEESFLRGLPKNLRLEIERQIYQKLLKHMPW : 331
BrCNGC3  : LRKRVREYEQYKWQETRGVEEEALLSSLPKDLRKEIKRHLCLNLLKKVPW : 474
BoCNGC2  : LRKRVRKYEQYKWQETRGVEEEVLLSSLPKDLRKDIKRHLCLNMLKTVPW : 474
BrCNGC5  : LRKRIRRYEQYKWQETRGVEEENLLRNLPKDLRRDIKRHFCLDLLKKVPL : 480
BoCNGC1  : LRKRIRRYEQYKWQETRGVEEENLLRNLPNDLRRDIKRHFCLDLLKKVPL : 481
BrCNGC6  : LRKRIRRYEQYKWQETRGVNEENLLSNLPKDLRRDIKRHLCLALVMRVPM : 514
BrCNGC7  : LRKRIRRYEQYKWQETRGVDEENLLSNLPKDLRRDIKRHLCLALLMRVPM : 509
BrCNGC8  : LRERVRRYDQYKWLETRGVDEENIVQSLPKDLRRDIKRHLCLNLVRRVPL : 472
BoCNGC8  : LRERVRRYDQYKWLETRGVDEENIVQSLPKDLRRDIKRHLCLNLVRRVPL : 501
BrCNGC9  : LRQRVRRYDQYKWLETRGVDEEYLVQNLPKDLRRDIKRHLCLALVRRVPL : 529
BoCNGC7  : LRQRVRRYDQYKWLETRGVDEEYLVQNLPKDLRRDIKRHLCLALVRRVPL : 501
BrCNGC10 : LRERVRRYDQYKWLETRGVDEENIVSNLPKDLRRDIKRHLCLALVRRVPL : 513
BoCNGC5  : LRERVRRYDQYKWLETRGVDEENIVSNLPKDLRRDIKRHLCLALVRRVPL : 513
BrCNGC11 : LRERVRRYDQYKWLETRGVDEENLVSNLPKDLRRDIKRHLCLALVRRVPL : 503
BoCNGC4  : LRERVRRYDQYKWLETRGVDEENLVSNLPKDLRRDIKRHLCLALVRRVPL : 503
BrCNGC12 : LRERVRRYDQYKWLETKGVDEENLVQNLPKDLRRDIKRHLCLALVRRVPL : 492
BoCNGC6  : LRERVRRYDQYKWLETKGVDEENLVQNLPKDLRRDIKRHLCLALVRRVPL : 518
BrCNGC13 : LRQAVRKYDQYKWLATRGVDEEALLISLPLDLRRDIKRHLCFDLVRRVPL : 478
BoCNGC9  : LRQAVRKYDQYKWLATRGVDEEALLISLPLDLRRDIKRHLCFDLVRRVPL : 478
BrCNGC14 : LQERVRRFVQYKWLATRGVDEESILQSLPTDLRREIQRHLCLALVRRVPF : 449
BoCNGC13 : LQERVRRFVQYKWLATRGVDEESILQSLPTDLRREIQRHLCLALVRRVPF : 436
BrCNGC15 : LQERVRRFVQYKWLTTRGVDEEAILRALPLDLRRQIQRHLCLALVRRVPF : 458
BoCNGC14 : LQERVRRFVQYKWLTTRGVDEEAILRALPLDLRRQIQRHLCLALVRRVPF : 458
BrCNGC16 : LRDRVRRYEQYKWLAARGVDEEVLLQSLPTDLRRDIQRHLCLDLVRRVPF : 485
BoCNGC10 : LRDRVRRYEQYKWLAARGVDEEVLLQSLPTDLRRDIQRHLCLDLVRRVPF : 485
BrCNGC17 : LRERVRRFVQYKWLATRGVDEETILHSLPADLRRDIQRHLCLDLVRRVPL : 486
BoCNGC12 : LRERVRRFVQYKWLATRGVDEETILHSLPADLRRDIQRHLCLDLVRRVPL : 486
BrCNGC18 : LRERVRRFVQYKWLATRGVDEETILHSLPADLRRDIQRHLCLDLVRRVPL : 482
BoCNGC11 : LRERVRRFVQYKWLATRGVDEETILHSLPADLRRDIQRHLCLDLVRRVPL : 482
BrCNGC22 : IRTRVKNAERLNWSSTRGVNEELLFENMSDDLQRDIRRHLFA-FLKKVRI : 526
BoCNGC19 : IRERVRDAERLNWSATRGVHEELLFENMPDDLQRDIRRHLFA-FLKKVRI : 429
BrCNGC27 : IRKRVREVERLNWNATRGVNEELLFENMPDDLQRDIRRHLFA-FLKKVRI : 551
BoCNGC18 : IRKRVREVERLNWNATRGVNEELLFENMPDDLQRDIRRHLFA-FLKKVRI : 545
BrCNGC28 : IRKRVREAERFNWAATRGVNEELLFENMPDDLQRDIRRHLFI-FLKKVRI : 589
BoCNGC21 : IRKRVREAERFNWAATRGVNEELLFENMPDDLQRDIRRHLFI-FLKKVRI : 588
BoCNGC20 : IRKRVREAERFNWAATRGVNEELLFKNMPDDLQREIRRHLFI-FLKKVRI : 582
BoCNGC22 : IRGRVRLAEWVNWAATRGFNEKIVFENMPNDLQRDIRRHLFI-FLKKVRV : 565
BrCNGC23 : IRKRVREVERFHWAARRGVDEELLFNNMPDDLQRDIRRHLFK-FLNKVRI : 635
BrCNGC24 : IRKRVREAERFNWAATRGVNEELLFENMPDDLQGDIRRHLFI-FLKKVRI : 491
```

```
BoCNGC23 : IRKRVREAERFNWAATRGVNEELLFENMPDDLQGDIRRHLFI-FLKKVRI : 612
BrCNGC25 : IRKRVREAERFNWAATRGVNEEFLFENMPEDLLRDIKRHLFK-FLKKVRI : 572
BoCNGC24 : IRKRVREAERFNWAATRGVNEELLFENMPEDLRRDIKRHLFK-FLKKVRI : 572
BrCNGC26 : IRKRVREAERFNWAATRGVKEELIFENMPDDIQRDIKRHLFK-FLKKVWI : 578
BoCNGC26 : IRKRVREAERFNWAATRGVKEELIFENMPDDIQRDIKRHLFK-FLKKVWI : 574
BrCNGC29 : IRKRVREAERFNWAATRGVNEELLFENMPDDLQRDIKRHLFK-FLKKVRI : 585
BoCNGC25 : IRKRVREAERFNWAATRGVNEELLFENMPDDLQRDIKRHLFK-FLKKVRI : 588
BrCNGC19 : FRQRVRNYERQRWAAMRGVDECEMVQNLPEGLRRDIKYHLCLDLVRQVPL : 497
BoCNGC15 : LRQRVRNYERQRWAAMRGVDECEMVQNLLEGLRRDIKYHLCLDLVRQVPL : 497
BrCNGC20 : YRQRVRNYERQRWAAMRGVDECEMVQNLPEGLRRDIKYHLCLDLVRQVPL : 500
BoCNGC16 : YRQRVRNYERQRWAAMRGVDECEMVQNLPEGLRRDIKYHLCLDLVRQVPL : 500
BrCNGC21 : LRQRVRRFERQRWTALGGEDELELIQDLPPGLRRDIKRYLCVDLINNVPL : 525
BoCNGC17 : LRQRVRRFERQRWTALGGEDELELIHDLPPGLRRDIKRYLCVDLINKVPL : 524
               r6       W  t g  ee     6p  6  di rh     6  6

                    *       820        *       840        *
BrCNGC1  : LNMMDDSWLLEALCDRVKSVFYSANSYIVKEGDPVAEMLIITKGSLKSMI : 477
BoCNGC3  : LNMMDDSWLLEALCDRVKSVFYSANSYIVKEGDPVAEMLIITKGSLKSMI : 477
BrCNGC2  : ISFIDDDWLLNEIYDRVKPVFYSQKSYILGEGDPVKEMLIVTYGELESMT : 513
BrCNGC4  : FEDMDD-RLLDSVCARLKTVIYTEDSYIVDEGEQIENMLFINRG---TVI : 377
BrCNGC3  : FKAMDD-RLLDALCARLNTVLYTENSYIVREGEPVEDMVFIMRGKLISTT : 523
BoCNGC2  : FKAMDD-RLLDALCARLKPALYTENSYIVREGEPLEDMVFIMRGKLTSTT : 523
BrCNGC5  : FEIMDE-QLLDAVCDKLRPVLYTENSYAIREGDPVEEMLFVMRGKLMSAT : 529
BoCNGC1  : FEIMDE-QLLDAVCDKLRPVLYTENSYAIREGDPVEEMLFVMRGKLMSAT : 530
BrCNGC6  : FEQMDE-QLLDALCDRLQPVLYTEESYIVREGDPVDEMLFIMRGKLLTMT : 563
BrCNGC7  : FEKMDE-QLLDALCDRLQPVLYTEESYIVREGDPVDEMLFIMRGKLLTMT : 558
BrCNGC8  : FANMDE-RLLDAICERLKPSLYTESTYIVREGDPVNEMLFIIRGRLESVT : 521
BoCNGC8  : FANMDE-RLLDAICERLKPSLYTESTYIVREGDPVNEMLFIIRGRLESVT : 550
BrCNGC9  : FESMDD-KLLDAICMRLKPCLFTESTYLVREGDPVDEMLFIIRGRLESVT : 578
BoCNGC7  : FESMDE-KLLDAICMRLKPCLFTESTYLVREGDPVDEMLFIIRGRLESVT : 550
BrCNGC10 : FDNMDE-RLLDAICMRLKPCLYTEKSFLVREGDPVNEMLFIIRGRLECVT : 562
BoCNGC5  : FENMDE-RLLDAICMRLKPCLYTEKSFLVREGDPVNEMLFIIRGRLESVT : 562
BrCNGC11 : FENMDE-RLLDAICERLKPCLYTEKSFLVREGDPVNEMLFIIRGRLESVT : 552
BoCNGC4  : FENMDE-RLLDAICERLKPCLYTEKSFLVREGDPVNEMLFIIRGRLESVT : 552
BrCNGC12 : FENMEE-RLLDAICERLKPCLYTEKSYLVREGDPVNEMLFIIRGRLESVT : 541
BoCNGC6  : FENMEE-RLLDAICERLKPCLYTEKSYLVREGDPVNEMLFIIRGRLESVT : 567
BrCNGC13 : FDQMDE-RMLDAISERLKPALFTEGTFLVREGDPVNEMLFIIRGHLDSYT : 527
BoCNGC9  : FDQMDE-RMLDAISERLKPALCTEGTFLVREGDPVNEMLFIIRGHLDSYT : 527
BrCNGC14 : FSQMDD-QLLDAICGCLVSSLSTAGTYIFREGDPVDEMLFVIRGQIESST : 498
BoCNGC13 : FSQMDD-QLLDAICGCLVSSLSTAGTYIFREGDPVDEMLFVIRGQIESST : 485
BrCNGC15 : FAQMDD-QLIDAICERLVPSLNTKDTYVTREGDPVNEMLFIIRGQMESST : 507
BoCNGC14 : FAQMDD-QLIDAICERLVPSLNTKDTYVTREGDPVNEMLFIIRGQMESST : 507
BrCNGC16 : FSQMDD-QLLDAICERLVSSLCTEGTYLVREGDLITEMLFIIRGRLESST : 534
BoCNGC10 : FSQMDD-QLLDAICERLVSSLCTEGTYLVREGDLITEMLFIIRGRLESST : 534
BrCNGC17 : FAQMDD-QLLDAICERLVSSLSTQGNYIVREGDPVTEMLFIIRGKLESST : 535
BoCNGC12 : FAQMDD-QLLDAICERLVSSLSTQGNYIVREGDPVTEMLFIIRGKLESST : 535
BrCNGC18 : FAQMDD-QLLDAICERLVSSLSTQGNYIVREGDPVTEMLFIIRGKLDSST : 531
BoCNGC11 : FAQMDD-QLLDAICERLVSSLSTQGNYIVREGDPVTEMLFIIRGKLDSST : 531
BrCNGC22 : FSKMDE-PILDAIRERLKHKTYLNGSMVLHSGDLVKKMVIIVRGQMESTR : 575
```

```
BoCNGC19 : FSKMDD-SILDAIRERLKHKTYVNGSMVLHRGDLVKKMVIIVRGTMESIG : 478
BrCNGC27 : FSEIDE-SILDAMRARLKQRTYLKNNKVLHRGGVVKKMVFILRGKMESIG : 600
BoCNGC18 : FSEIDE-SILDAMRARLKQRTYLKNNKVLHRGGVVKKMVFILRGKMESIG : 594
BrCNGC28 : FSLMDE-SILDAIRERLKQRTYISSSTVLHRGGLVEKMVFIVRGEMESIG : 638
BoCNGC21 : FSLMDE-SILDAIRERLKQRTYISSSTVLHRGGLVEKMVFIVRGEMESIG : 637
BoCNGC20 : FSVMDE-SILDAIRERLKQRTYIRSSMVLHRGGLVEKMVFIVRGEIESVG : 631
BoCNGC22 : FSKMDE-LILDAIRERLTYRRYISSSTVLHRGGVVEKMVFIVRGEMESFG : 614
BrCNGC23 : FTLMDE-SILNAIRERLKHITYISSSVVFSAGDVIEKIVFIVRGEMESIG : 684
BrCNGC24 : FSLMDE-SILDSIRERLRQRTYISGSTVLHRWGPVEKIVFVVRGEMESTG : 540
BoCNGC23 : FSLMDE-SILDSIRERLRQRTYISGSTVLHRWGLVEKIVFIVRGEMESIG : 661
BrCNGC25 : FSLMDE-SILDSIRERLKQRTYISSSTVLHRRGLVEKMVFIVRGEMESIG : 621
BoCNGC24 : FSLMDE-SILDSIRERLKQRTYISSSTVLHRRGLVEKMVFIVRGGMESIG : 621
BrCNGC26 : FSLMDE-LILDAIRERLKQRRYIRSSTVLRRKGLVEKMVFIVRGERESIG : 627
BoCNGC26 : FSLMDE-LILDAIRERLKQRRYIRSSTVLRRKGLVEKMVFIVRGEMESIG : 623
BrCNGC29 : FSLMDE-SILDAIRERLKQRTYIRSSTVLHRKGLVEKMVFIVRGEMESIG : 634
BoCNGC25 : FSLMDE-SILDAIRERLKQRTYIRSSTVLHRKGLVEKMVFIVRGEMESIG : 637
BrCNGC19 : FQHMDD-LVLENICDRVKSLIFTKGETIQKEGDAVQRMLFVVRGHLQSSQ : 546
BoCNGC15 : FQHMDD-LVLENICDRVKSLIFTKGETIQKEGDAVQRMLFVVRGHLQSSQ : 546
BrCNGC20 : FQHMDD-LVLENICDRVKSLIFTKGETIQKEGDAVQRMLFVVRGHLQSSQ : 549
BoCNGC16 : FQHMDD-LVLENICDRVKSLIFTKGETIQKEGDAVQRMLFVVRGHLQSSQ : 549
BrCNGC21 : FRGMDD-LILDNICDRAKPRVYSKDEKIIREGDPVQRMIFIMRGRVKRNQ : 574
BoCNGC17 : FRGMDD-LILDNICDRAKPRVYSKDEKIIREGDPVQRMIFIMRGRVKRNQ : 573
           f  6d   66  6                  g  6  66f6  G

                  860         *       880         *       900
BrCNGC1  : GSSDITGYYDSSY-LQAGDICGDLLFWVLDP----------HSSSSLPTS : 516
BoCNGC3  : GFSDITGYYDSSY-LQAGDICGDLLFWVLDP----------HSSSSLPTS : 516
BrCNGC2  : ESFETSSYSDIQIRLMKGDVWEDLLFWALDP----------HTSPSLPIS : 553
BrCNGC4  : VTEKKTTFFRSVG----EFCGEELLSWALLD----------PHSSCVPIS : 413
BrCNGC3  : TYGGQTGFFNIAH-LEAGDFCGDLLTWALDP----------NTS-HLPIS : 561
BoCNGC2  : TYGGKSGFFNSVS-LGVGQFCGDLLTWALDP----------NTS-HFPIS : 561
BrCNGC5  : TNGGRTGFFNAVYLKASDFCGEDLLTWALDP----------QSSSHFPIS : 569
BoCNGC1  : TNGGRTGFFNAVYLNASDFCGEDLLTWALDP----------QSSSHFPIS : 570
BrCNGC6  : TNGGRTGFFNSEHLGAGDFCGEELLTWALDP----------HTSTNLPIS : 603
BrCNGC7  : TNGGRTGFFNSEHLGAGDFCGEELLTWALDP----------HTSSNLPIS : 598
BrCNGC8  : TDGGRSGFFNRGLLKEGDFCGEELLTWALDP----------KAGSNLPSS : 561
BoCNGC8  : TDGGRSGFFNRGLLKEGDFCGEELLTWALDP----------KAGSNLPSS : 590
BrCNGC9  : TDGGRSGFFNYSLLKEGEFCGEELLTWALDP----------KSGVNLPSS : 618
BoCNGC7  : TDGGRSGFFNYSLLKEGEFCGEELLTWALDP----------KSGVNLPSS : 590
BrCNGC10 : TDGGRSGFYKRSLLKEGDFCGDELLTWALDP----------KSGSNLPSS : 602
BoCNGC5  : TDGGRSGFYNRSLLKEGDFCGDELLTWALDP----------KSGSNLPSS : 602
BrCNGC11 : TDGGRSGFYNRSLLKEGDFCGDELLTWALDP----------KSGSNLPSS : 592
BoCNGC4  : TDGGRSGFYNRSLLKEGDFCGDELLTWALDP----------KSGSNLPSS : 592
BrCNGC12 : TDGGRSGFFNRSLLKEGDFCGEELLTWALDP----------KSGSNLPSS : 581
BoCNGC6  : TDGGRSGFFNRSLLKEGDFCGEELLTWALDP----------KSGSNLPSS : 607
BrCNGC13 : TNGGRTGFFNSCLIGPGDFCGEELLTWALDP----------RPVVILPSS : 567
BoCNGC9  : TNGGRTGFFNSCLIGPGDFCGEELLTWALDP----------RPVVILPSS : 567
BrCNGC14 : TNGGRSGFFNSTTLRPGDFCGEELLTWALMP----------NSTLNFPSS : 538
BoCNGC13 : TNGGRSGFFNSTTLRPGDFCGEELLTWALMP----------NSTLNFPSS : 525
```

```
BrCNGC15 : TDGGRSGFFNSITLRPGDFCGEELLTWALMP----------NINQNLPLS : 547
BoCNGC14 : TDGGRSGFFNSITLRPGDFCGEELLTWALMP----------NINQNLPLS : 547
BrCNGC16 : TNGGRTGFFNSIILRPGDFCGEELLSWALLP----------KSTLNLPSS : 574
BoCNGC10 : TNGGRTGFFNSIILRPGDFCGEELLSWALLP----------KSTLNLPSS : 574
BrCNGC17 : TNGGRTGFFNSITLKPGEFCGEELLAWALLP----------KSKVNLPSS : 575
BoCNGC12 : TNGGRTGFFNSITLKPGEFCGEELLVWALLP----------KSKVNLPSS : 575
BrCNGC18 : TNGGRTGFFNSITLKPGEFCGEELLAWALLP----------KSKVNLPSS : 571
BoCNGC11 : TNGGRTGFFNSITLKPGEFCGEELLAWALLP----------KSKVNLPSS : 571
BrCNGC22 : EDG------SCTRLSKRDVYGEELLTWY-ERSSLNPDGTRIRMPSKGLLS : 618
BoCNGC19 : EDG------YRTVLSEEFVCGEELLTWCQERSSVNPDGTMIRMPSKGLIS : 522
BrCNGC27 : EDG------YRTLLSEGDVCGEELLTWCQERSSVNPDGTIIRMPSKGLLS : 644
BoCNGC18 : EDG------YRILLSEGDVCGEELLTWCQERSSVNPDGTMIRMPSKGLLS : 638
BrCNGC28 : EDG------SVLPLSEGDVCGEELLTWCLERSSVNPDGTRIRIPSKGLLS : 682
BoCNGC21 : EDG------SVLPLSEGDVCGEELLTWCLERSSVNPDGTRIRIPSKGLLS : 681
BoCNGC20 : EDG------YVLPLSEGDVCGEELLTWCLERSSVNPDGTRIRMPSKGLLS : 675
BoCNGC22 : EDG------YVLPLSEGDVCGEELLAWCLGRSSVNPDGTRIRMPSKGLVS : 658
BrCNGC23 : KDG------SVISLSEGDVFGEELLSWCLERAASNTDGTRIWIKRKGLLS : 728
BrCNGC24 : EDG------SVLPLSEGDVCGEELLTWCLERSAVNLDG---RMLSKGLLS : 581
BoCNGC23 : EDG------SVLPLSEGDVCGEELLTWCLERSAVNLDG---RMLSKGLLS : 702
BrCNGC25 : QDG------SVLLLSEGDVCGEELLTWCLERSSVNPDGRSIKLPLKGLVS : 665
BoCNGC24 : QDG------SVLLLSEGDVFGEELLTWCLERSSVNPDGRSIKLPLKGLVS : 665
BrCNGC26 : EDG------SVLPLSEGDVCGEELLTWCLERSSVDPDGTRIKMPTKGLVS : 671
BoCNGC26 : EDG------SVLPLSQGDVCGEELLTWCLERSSVDPDGTRIKMPTKGLVS : 667
BrCNGC29 : EDG------SVLPLSEGDVCGEELLTWCLERSSVNPDGTRIKMPTKGLVS : 678
BoCNGC25 : EDD------SVLPLSEGDVCGEELLTWCLERSSVNPDGTRIKMPTKGLVS : 681
BrCNGC19 : LLR--DGVKSCCMLGPGNFSGDELLSWCLRR----------PFVERLPPS : 584
BoCNGC15 : LLR--DGVKSCCMLGPGNFSGDELLSWCLRR----------PFVERLPPS : 584
BrCNGC20 : LLR--DGVRSCCMLGPGNFSGDELLSWCLRR----------PFVERLPPS : 587
BoCNGC16 : LLR--DGVRSCCMLGPGNFSGDELLSWCLRR----------PFVERLPPS : 587
BrCNGC21 : SLS--KGVVATSTLEPGGYLGDELLSWCLRR----------PFIDRLPPS : 612
BoCNGC17 : SLS--KGVVATSTLEPGGYLGDELLSWCLRR----------PFIDRLPPS : 611
               g             g   g eLL W l                    S

                    *       920         *       940         *
BrCNGC1  : DRSVLTLTDVEGFILLHDDLKFVASHFNRFHSSRLRHMF------YSAHW : 560
BoCNGC3  : DRSVLTLTDVEGFILLHDDLKFVASHFNRSHSSRLRHMFR----FYSAHW : 562
BrCNGC2  : NGTVTTLTYVEGLTLSADDLKCHS-----------------------KSW : 580
BrCNGC4  : SMIIKARTEVEGFVLSADDLKFVAAQYCRLHRKKARHISRQEFQLHSRKW : 463
BrCNGC3  : TRTVQAETEVEGFVLSAEDLKFFSTQYRRLHSKQLRHTC------YSVQW : 605
BoCNGC2  : TSTVQAQTEVEGFVLSADDLKFVATQYRRINSKQLRHMFR----YHSVQW : 607
BrCNGC5  : PRTVQALTEVEAFALAAEDLKLVASQFRRLHSKQLQHTFR----FYSVQW : 615
BoCNGC1  : PRTVQALTEVEAFALAAEDLKLVASQFRRLHSKQLQHTFR----FYSVQW : 616
BrCNGC6  : TRTVQALVEVEAFALKADNLKFVASQFRRLHSKQLRHTFR----FYSQQW : 649
BrCNGC7  : TRTVRALVEVEAFALKADDLKFVASQFRRLHSKQLRHTFR----FYSQQW : 644
BrCNGC8  : TRTVKALTEVEAFALEAEELKFVASQFRRLHSRQVQQTFR----FYSQQW : 607
BoCNGC8  : TRTVKALTEVEAFALEAEELKFVASQFRRLHSRQVQQTFR----FYSQQW : 636
BrCNGC9  : TRTVKALTEVEAFALASEELKFVASQFRRLHSRQVQHTFR----FYSHQW : 664
BoCNGC7  : TRTVKALTEVEAFALASEELKFVASQFRRLHSRQVQHTFR----FYSHQW : 636
BrCNGC10 : TRTVKALTEVEAFALIADELKFVASQFRRLHSRQVQHTFR----FYSQQW : 648
```

```
BoCNGC5  : TRTVKALTEVEAFALIADELKFVASQFRRLHSRQVQHTFR----FYSQQW : 648
BrCNGC11 : TRTVKALTEVEAFALIADELKFVASQFRRLHSRQVQHTFR----FYSQQW : 638
BoCNGC4  : TRTVKALTEVEAFALIADELKFVASQFRRLHSRQVQHTFR----FYSQQW : 638
BrCNGC12 : TRTAKALTEVEAFALIADELKFVASQFRRLHSRQVQHTFR----FYSQQW : 627
BoCNGC6  : TRTAKALTEVEAFALIADELKFVASQFRRLHSRQVQHTFR----FYSQQW : 653
BrCNGC13 : TRTVKAIYEVEAFALRADDLKFVATQFRRLHSKQLKHKFR----FYSHQW : 613
BoCNGC9  : TRTVKAIYEVEAFALRADDLKFVATQFRRLHSKQLKHKFR----FYSHQW : 613
BrCNGC14 : TRSVRALSEVEAFALSAEDLKFVAHQFKRLQSKKLQHAFR----YYSHQW : 584
BoCNGC13 : TRSVRALSEVEAFALSAEDLKFVAHQFKRLQSKKLQHAFR----YYSHQW : 571
BrCNGC15 : TRTVRTLSEVEAFALRAEDLKFVANQFRRLHSKKLQHAFR----YYSHQW : 593
BoCNGC14 : TRTVRTLSEVEAFALRAEDLKFVANQFRRLHSKKLQHAFR----YYSHQW : 593
BrCNGC16 : TRTVRALVEVEAFALRAEDLKFVANQFRRLHSKKLQHTFR----FYSPHG : 620
BoCNGC10 : TRTVRALVEVEAFALRAEDLKFVANQFRRLHSKKLQHTFR----FYSHHW : 620
BrCNGC17 : TRTVRALEEVEAFALQAEDLKFVANQFRRLHSKKLQHTFR----YYSHQW : 621
BoCNGC12 : TRTVRALEEVEAFALQAEDLKFVANQFRRLHSKKLQHTFR----YYSHQW : 621
BrCNGC18 : TRTVRALEEVEAFALQAEDLKFVANQFRRLHSKKLQHTFR----YYSHQW : 617
BoCNGC11 : TRTVRALEEVEAFALQAEDLKFVANQFRRLHSKKLQHTFR----YYSHQW : 617
BrCNGC22 : NRDVKCVTNVEAFSLSVADLDDVMSLFPN--------------------- : 647
BoCNGC19 : SRAVWCVTNVEAFSLSVADLEDIMSLFPN--------------------- : 551
BrCNGC27 : SRDVWCVTNVEAFSLSVADLEDITSLFPN--------------------- : 673
BoCNGC18 : SRDVWCVTNVEAFSLSVADLEDITSLFPN--------------------- : 667
BrCNGC28 : YRNVKCVTNVEAFSLSVADLEDVTSLFSRFLRNPRVQGAI---RYESPYW : 729
BoCNGC21 : YRNVRCVTNVEAFSLSVADLEDVTSLFSRFLRNPRVQGAI---RYESPYW : 728
BoCNGC20 : NRNVRCVTNVEAFSLSVADLEDVTSLFSRFLRSNRVQGAI---RYESPYW : 722
BoCNGC22 : NRNVRCVTDVEAFSLSVADLEDITSLFLRFLRTHREQGAI---R--SPHW : 703
BrCNGC23 : YRSVRCVTNVDAFSLSVADLDAS--------------------------- : 751
BrCNGC24 : SRNVKCVTNVEAFSLSAADLEDVTSLFSRFLRSHRVQGAI---RYESPYC : 628
BoCNGC23 : SRNVKCVTNVEAFSLSAADLEDVTSLL--FLRSHRVQGAI---RYESPYC : 747
BrCNGC25 : NRSVRCVTNVEAFSLSVADLEDVTSLFSRFLRSHRVLGAI---RYESPYW : 712
BoCNGC24 : NRSVRCVTNVEAFSLSVADLEDVTSLFSRFLRSHRVLGAI---RYESPYW : 712
BrCNGC26 : NRSVRCVTNVEAFSLSVADLEDVTSLFSRFLRSNRVQGAI---RYESPYW : 718
BoCNGC26 : NRSVRCVTNVEAFSLSVADLEDVTSLFSRFLRSNRVQGAI---RYESPYW : 714
BrCNGC29 : NRNVRCVTNVEAFSLSVADLEDVTSLFSRFLRSHRVQGAI---RYESPYW : 725
BoCNGC25 : NRNVRCVTNVEAFSLSVADLEDVTSLFSRFLRSHRVQGAI---RYESPYW : 728
BrCNGC19 : SSTLVTLETTEAFGLDAQDVKYVTQHFRYTFVNEKVKRSAR---YYSPGW : 631
BoCNGC15 : SSTLVTLETTEAFGLDAQDVKYVTQHFRYTFVNEKVKRSAR---YYSPGW : 631
BrCNGC20 : TSTLVTLETTEAFGLDAEDVKYVTQHFRYTFVNEKVKRSAR---YYSPGW : 634
BoCNGC16 : TSTLVTLETTEAFGLDAEDVKYVTQHFRYTFVNEKVKRSAR---YYSSGW : 634
BrCNGC21 : SATFVCLDNIEAFSLGSEDLRYITDHFRYKFANERLKRTAR---YYSSNW : 659
BoCNGC17 : SATFVCLDSIEAFSLGSEDLRYITDHFRYKFANERLKRTAR---YYSSNW : 658
             r        eaf L    6                           s  w

                   960         *         980         *      1000
BrCNGC1  : RLWAACFIQAAWREHYKRKLSRILHAKR---------------------- : 588
BoCNGC3  : RLWAACFIQAAWREHCKRKLSRILHAKR---------------------- : 590
BrCNGC2  : RSWAAFYIQAAWKAHCRRKASKILPAIK---------------------- : 608
BrCNGC4  : RNWAACFIQAAWTEYCRRKLSKDLGEEE---------------------- : 491
BrCNGC3  : QTWAACFIQAAWKRYCRRKLSRVLREEE---------------------- : 633
BoCNGC2  : RTWAACFIQAAWKRYCRKKLSRALREEE---------------------- : 635
```

```
BrCNGC5  : RTWGASFIQAAWRRHCRRRLARSLTEEE---------------------- : 643
BoCNGC1  : RTWGASFIQAAWRRHCRRRLARSLTQEE---------------------- : 644
BrCNGC6  : RTWAACFIQAAWRRHVKKRLEESLREEE---------------------- : 677
BrCNGC7  : RTWAACFIQAAWRRHVKKKMEESLKEEE---------------------- : 672
BrCNGC8  : RTWASSFIQAAWRRHSRRKNAELRRIEEEEDEMGYEDEYDD--------- : 648
BoCNGC8  : RTWASSFIQAAWRRHSRRKNAELRRIEEEEDEMGYEDEYDD--------- : 677
BrCNGC9  : RTWAACFIQAAWRRYCKRKKMEEAEAEA---------------------- : 692
BoCNGC7  : RTWAACFIQAAWRRYCKRKKMEEAEAEA---------------------- : 664
BrCNGC10 : RTWAACFIQAAWRRYTKRKKLEQLRKEE---------------------- : 676
BoCNGC5  : RTWAACFIQAAWRRYTKRKKLEQLRKEE---------------------- : 676
BrCNGC11 : RTWAACFIQAAWRRYTKRKKLEELRKEE---------------------- : 666
BoCNGC4  : RTWAACFIQAAWRRYTKRKKLEELRKEE---------------------- : 666
BrCNGC12 : RTWAAIFIQAAWRRYVKKKKLEQLKKEE---------------------- : 655
BoCNGC6  : RTWAAIFIQAAWRRYVKKKKLEQLKKEE---------------------- : 681
BrCNGC13 : RTWAACFIQAAWRRHKKRKYATELRVKEEFQCMFETAS------------ : 651
BoCNGC9  : RTWAACFIQAAWRRHKKRKYATELRVKEEFQCMFETAS------------ : 651
BrCNGC14 : RAWGACFVQSAWRRYKRRKLAKELSLHESSGYYYRDETGYNEEGDEENYY : 634
BoCNGC13 : RAWGACFVQSAWRRYKRRKLAKELSLHESSGYYYRDETGYNEEGDEENYY : 621
BrCNGC15 : RAWGTGFIQAAWRRYMKRKLAMELARQEEGDDYYYDD-------DDDDQY : 636
BoCNGC14 : RAWGTGFIQAAWRRYMKRKLAMELARQEEGDDYYYDD-------DDDDQY : 636
BrCNGC16 : RTWAACFIQAAWRRYKRRAMENHLTAVESKQSDEDDEEEE---VVVRKVV : 667
BoCNGC10 : RTWAACFIQAAWRRYKRRAMENHLTAVESKQSDEE-EEEE---VVVGKVV : 666
BrCNGC17 : RTWAACFVQVAWRRYKRRMLAKSLSLAESYSSYEEEEALAA--AAAEEIM : 669
BoCNGC12 : RTWAACFVQVAWRRYKRRMLAKSLSLAESYSSYEEEEALAT--AAAEEIM : 669
BrCNGC18 : RTWAACFVQVAWRRYKRRMVAKSLSLAESFSSYEEEEAVA---VAAEEIM : 664
BoCNGC11 : RTWAACFVQVAWRRYKRRMVAKSLSLAESFSSYEEEEAVA---VAAEEIM : 664
BrCNGC22 : --------------------LEISKES----------------------- : 654
BoCNGC19 : --------------------LEIPKEP----------------------- : 558
BrCNGC27 : --------------------LEIPKEP----------------------- : 680
BoCNGC18 : --------------------LEIPKEP----------------------- : 674
BrCNGC28 : RLRAARQIQVAWRYRRRRLQRLYTAQSSYSL------------------- : 760
BoCNGC21 : RLRAARQIQVAWRYRRRRLQRLYTAQSSYSL------------------- : 759
BoCNGC20 : RLRAAMQIQVAWRYRRRRLQRLYTDQSSYSL------------------- : 753
BoCNGC22 : RLRAARQIQVPWRHRRRRLQRFYSAQSSYSL------------------- : 734
BrCNGC23 : --------------------RDCSRDS----------------------- : 758
BrCNGC24 : RLRAATQIQVAWRYRKRRLQKLSTAQKKQYSSELIQKDMAKT-------- : 670
BoCNGC23 : RLRAATQIQVAWRYRKRQLRKLSTAQKKQYSLELIKKDMAKT-------- : 789
BrCNGC25 : RLRAATQIQVAWRYRRRRLHRLYTAQSTSRR------------------- : 743
BoCNGC24 : RLRAATQIQVAWRYRRRRLHRLYTAQSTSRR------------------- : 743
BrCNGC26 : RLRAAMQIQVAWRYRRKRLERK--KQNGDIDE------------------ : 748
BoCNGC26 : RLRAAMQIQVAWRYRKKRLERR--KQNGEMDE------------------ : 744
BrCNGC29 : RLRAAMQIQVAWRYRKKRLERLLLQDNRVMKEMSDVRIGYRLSWSGVVEG : 775
BoCNGC25 : RLRAAMQIQVAWRYRKKRLERLLLQDNRVMKEMSDVPIGYRLSWSGVVEG : 778
BrCNGC19 : RTWAAVAVQLAWRRYKHRLTLTSLSFIR---------------------- : 659
BoCNGC15 : RTWAAVAVQLAWRRYKHRFTLTSLSFIR---------------------- : 659
BrCNGC20 : RTWAAVAVQLAWRRYKHRLTLTSLSFIR---------------------- : 662
BoCNGC16 : RTWAAVAIQLAWRRYKHRLTLTSLSFIR---------------------- : 662
BrCNGC21 : RTWAAVNIQMSWRRYRKRTCGGVGGSMS---------------------- : 687
BoCNGC17 : RTWAAVNIQMSWRRYRKRTC--VGGSMS---------------------- : 684
```

```
           r  aa   q aw

                   *      1020           *      1040          *
BrCNGC1  : ---DYNH--IPQG-----TQLNIGAALYVSRFVSKALRNRQKNAANC--- : 625
BoCNGC3  : ---DYNH--IPQG-----PQLNLGAALYVSRFVSKALRNRQKNAANC--- : 627
BrCNGC2  : ---DEQQ--ILQD-----TQRNLGATLYASRFVSKALRNRHVDSAEC--- : 645
BrCNGC4  : ---AKLHSTIQKDDSAG-NTHNLGGTAFASRLASTVLRNLRVNASRK--- : 534
BrCNGC3  : ---ERLQNTLQTTDDSGGNKLNLGAAIYASRFASHALRNVRANAAARS-- : 678
BoCNGC2  : ---ERLENTLQT-DDSGGNKLNLGAAIYASRFASHALRNVRANAAARN-- : 679
BrCNGC5  : ---DRFRIAVAKRERRAASSPSLVATLYASRFASNALRNLRQHNNNTL-- : 688
BoCNGC1  : ---DQFRIAVAKRERRAASSSSLVATLYASRFASNALRNLRQHN--TL-- : 687
BrCNGC6  : ---NRLQDALAKQACG--SSPSFGATMYASRFAANILRTIRRSGSVRK-- : 720
BrCNGC7  : ---NRLQDALAKEACG--SSPSLGATMYASRFAANILRTIRRSGSVRK-- : 715
BrCNGC8  : -DDAEEEDERTPVFTRTESSSRLRSTIFASRFAANALKGHRLRSTE---- : 693
BoCNGC8  : -DDAEEEDERTPVFTRTESSSRLRSTIFASRFAANALKGHRLRSTE---- : 722
BrCNGC9  : --VPMST---------TGSSSSMGAAFLVTKFAASALRTIHRNRNTR--- : 728
BoCNGC7  : --VPMST---------TGSSSSMGAAFLVTKFAASALRTIHRNRNTR--- : 700
BrCNGC10 : --EEEEEESAARLIAGGS-PYSIRATFLASKFAANALRSVHKNRIRKS-- : 721
BoCNGC5  : --EEEEE-TAARLIAGGS-PYSIRATFLASKFAANALRSVHKNRIRKS-- : 720
BrCNGC11 : --EMEEESSTARLIAGGSSPFSIRATFLASRFAANALRGVRKNRTAKL-- : 712
BoCNGC4  : --EMEEESSAARLIAGGSSPYSIRATFLASRFAANALRGVRKNRTAKL-- : 712
BrCNGC12 : --EEE----------GEGHVASIRATFLASKFAANALRKVHQNRIA---- : 689
BoCNGC6  : --EEEEE-------EGEGPVASIRATFLASKFAANALRKVHQNRIA---- : 718
BrCNGC13 : ----------------------MVRLNSGKFTR----------------- : 662
BoCNGC9  : ----------------------MVRLNSGKFTR----------------- : 662
BrCNGC14 : GSDDDDFEGERLSVDNTNNSQNLGATMLASKFAANTRRGTNQKASSSTSA : 684
BoCNGC13 : GSDDDDFEGERLSVDNTNNSQNLGATMLASKFAANTRRGTNQKASSSSST : 671
BrCNGC15 : GGED-MPESSNNVDDNSSNSQNLSATILASKFAANTKRGVLGNQRGSS-- : 683
BoCNGC14 : GGED-MPESSNNVDDNSSNNQNLSATILASKFAANTKRGVLGNQRGSS-- : 683
BrCNGC16 : EEEEEGVGSSPK------TKMNIGVMVLASRFAANTRRGV---------- : 701
BoCNGC10 : EEEEEGVGPSPN------TKMNIGVMVLASRFAANTRRGV---------- : 700
BrCNGC17 : SQQEERQSSTPSRHHTSIGKPHFAATVLASRFAKNTRR------------ : 707
BoCNGC12 : SQQEERQSSTPSRHHTSIEKPHFAATVLASRFAKNTRR------------ : 707
BrCNGC18 : SQQGERQSSNPSRHSTSIGKPHFAATILASRFAKNTRK------------ : 702
BoCNGC11 : SQQGERQSSNPSRHSTSIGKPHFAATILASRFAKNTRK------------ : 702
BrCNGC22 : -------------------------------------------------- :   -
BoCNGC19 : -------------------------------------------------- :   -
BrCNGC27 : -------------------------------------------------- :   -
BoCNGC18 : -------------------------------------------------- :   -
BrCNGC28 : -------------------------------------------------- :   -
BoCNGC21 : -------------------------------------------------- :   -
BoCNGC20 : -------------------------------------------------- :   -
BoCNGC22 : -------------------------------------------------- :   -
BrCNGC23 : -------------------------------------------------- :   -
BrCNGC24 : -------------------------------------------------- :   -
BoCNGC23 : -------------------------------------------------- :   -
BrCNGC25 : -------------------------------------------------- :   -
BoCNGC24 : -------------------------------------------------- :   -
BrCNGC26 : -------------------------------------------------- :   -
```

```
BoCNGC26 : -------------------------------------------------- :   -
BrCNGC29 : SGKQLDTRGSS--------------------------------------- : 786
BoCNGC25 : SGK----------------------------------------------- : 781
BrCNGC19 : -----PRRPLSRCASLGEDKLRLYTAILTS-------------------- : 684
BoCNGC15 : -----PRRPLSRCASLGEDKLRLYTAILTS-------------------- : 684
BrCNGC20 : -----PRRPLSRCASLGEDKLRLYTAILTS-------------------- : 687
BoCNGC16 : -----PRRPLSRCASLGEDKLRLYTAILTS-------------------- : 687
BrCNGC21 : -----PVSEHS-VEGNSERRLLQYAAMFMS-------------------- : 711
BoCNGC17 : -----PVSEHS-VEGNSERRLLQYAAMFMS-------------------- : 708
```

```
                 1060         *       1080         *
BrCNGC1  : --SISPHMLPPI--PHKPADPEFSKN-------------- : 647
BoCNGC3  : --SISPHMLPPI--PHKPADPEFSKN-------------- : 649
BrCNGC2  : --SSFPEMLP-----DKPADPEFSKKEA------------ : 666
BrCNGC4  : --DRVRQISLP----EKPVDPKFPMDEI------------ : 556
BrCNGC3  : --SMLPHMLSLL--PQKPADPEFPMDET------------ : 702
BoCNGC2  : --SMLPHMLSLL--PQKPADPEFPMDET------------ : 703
BrCNGC5  : --PLLP---------PKPSEPDFSVDDD------------ : 705
BoCNGC1  : --PLLP---------PKPSEPDFGVDDD------------ : 704
BrCNGC6  : --PRMLERMPPMLLLQKPAEPDFNSDDYMRIVPIYKNRSL : 758
BrCNGC7  : --PRMPERM----LLQKPAEPDFNSDDYCI---------- : 739
BrCNGC8  : -------SSKRLLNLQKPPEPDFDAE-------------- : 712
BoCNGC8  : -------SSKRLLNLQKPPEPDFDAE-------------- : 741
BrCNGC9  : --------IRELVKLQKPPEPDFTAEDAD----------- : 749
BoCNGC7  : --------IRELVKLQKPPEPDFTAEDAD----------- : 721
BrCNGC10 : --NLAPPSTKELVKFQKPPEPDFSADC------------- : 746
BoCNGC5  : --NLAPPSTKELVKFQKPPEPDFSADC------------- : 745
BrCNGC11 : --LALSQPTKELLKVQKPPEPDFSADC------------- : 737
BoCNGC4  : --LALSQPTKELLKVQKPPEPDFSADC------------- : 737
BrCNGC12 : -----AKSTKELVIFQKPSEPDFSADDP------------ : 712
BoCNGC6  : -----AKSTKELVIFQKPSEPDFSADDP------------ : 741
BrCNGC13 : ----SGSDSGMVSSIQKPVEPDFSSE-------------- : 684
BoCNGC9  : ----SGSDSGMVSSIQKPVEPDFSSE-------------- : 684
BrCNGC14 : GKKDGSSNSLKMPQLFKPDEPDFSMDKEDV---------- : 714
BoCNGC13 : GKKDGSSNSLKMPQLFKPDEPDFSMDKEDV---------- : 701
BrCNGC15 : -RIDPDDPTLKMPKMFKPEDPGFF---------------- : 706
BoCNGC14 : -RIDPDDPTLKMPKMFKPEDPGFF---------------- : 706
BrCNGC16 : --AAQRVKDVEMPRFKKPEEPDFSAEPDD----------- : 728
BoCNGC10 : --AAQRVKDVEMPRFKKPEEPDFSAEPDD----------- : 727
BrCNGC17 : --ASRKMKDVDVPMLPKPEEPDFSVDAD------------ : 733
BoCNGC12 : --ASRKIKDVDVPMLPKPEEPDFSVDAD------------ : 733
BrCNGC18 : --TAHKLKDVEVPMLPKPDEPDFSVDGD------------ : 728
BoCNGC11 : --TAHKLKDVEVPMLPKPDEPDFSVDGD------------ : 728
BrCNGC22 : ---------------------------------------- :   -
BoCNGC19 : ---------------------------------------- :   -
BrCNGC27 : ---------------------------------------- :   -
BoCNGC18 : ---------------------------------------- :   -
BrCNGC28 : ---------------------------------------- :   -
```

```
BoCNGC21 : ---------------------------------------- :   -
BoCNGC20 : ---------------------------------------- :   -
BoCNGC22 : ---------------------------------------- :   -
BrCNGC23 : ---------------------------------------- :   -
BrCNGC24 : ---------------------------------------- :   -
BoCNGC23 : ---------------------------------------- :   -
BrCNGC25 : ---------------------------------------- :   -
BoCNGC24 : ---------------------------------------- :   -
BrCNGC26 : ---------------------------------------- :   -
BoCNGC26 : ---------------------------------------- :   -
BrCNGC29 : ---------------------------------------- :   -
BoCNGC25 : ---------------------------------------- :   -
BrCNGC19 : ---------------PKPNPDDFDDY-------------- : 695
BoCNGC15 : ---------------PKPNPDDFDDY-------------- : 695
BrCNGC20 : ---------------PKPNPDDFDDY-------------- : 698
BoCNGC16 : ---------------PKPNPDDFDDY-------------- : 698
BrCNGC21 : ---------------IRPH-DHLE---------------- : 719
BoCNGC17 : ---------------IRPH-DHLE---------------- : 716
```

Figure S4. Phylogenetic relationship between *CNGCs* from *B. rapa* L. and *A. thaliana*

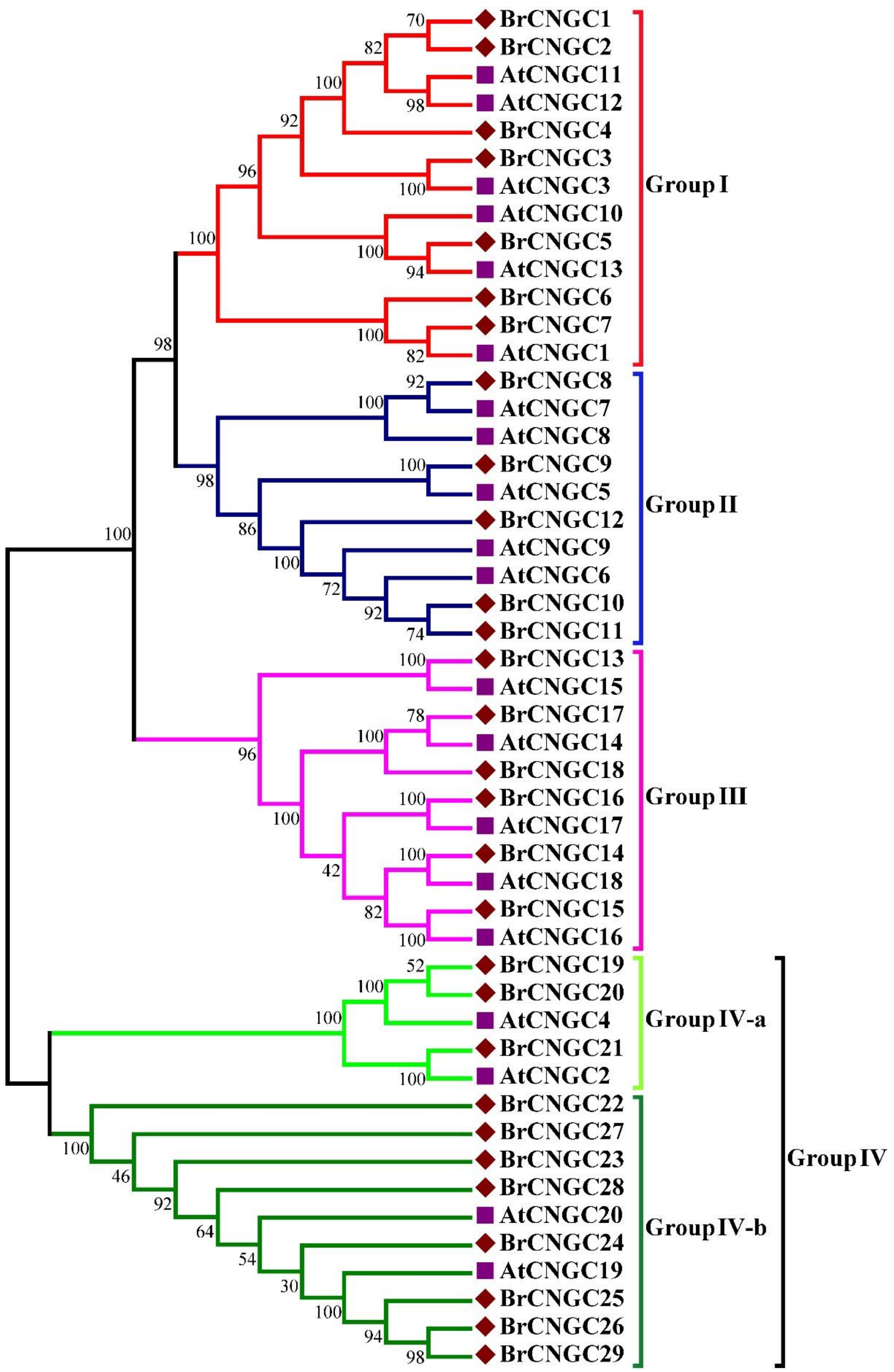

Figure S5. Phylogenetic relationship of CNGCs between five *Brassica* species. The evolutionary history was inferred using the Neighbor-Joining method in MEGA. The optimal tree with the sum of branch length = 7.85138788 is shown. The evolutionary distances were computed using the JTT matrix-based method and are in the units of the number of amino acid substitutions per site. The analysis involved 194 amino acid sequences. All positions containing gaps and missing data were eliminated. There were a total of 148 positions in the final dataset. Each *Brassica* specie is represented with colored diamonds, while AtCNGCs are indicated with black triangles. *B. rapa* L. = BrCNGCs, *B. napus* = BnCNGCs, *B. nigra* = BniCNGCs, *B. oleracea* = BoCNGCs.

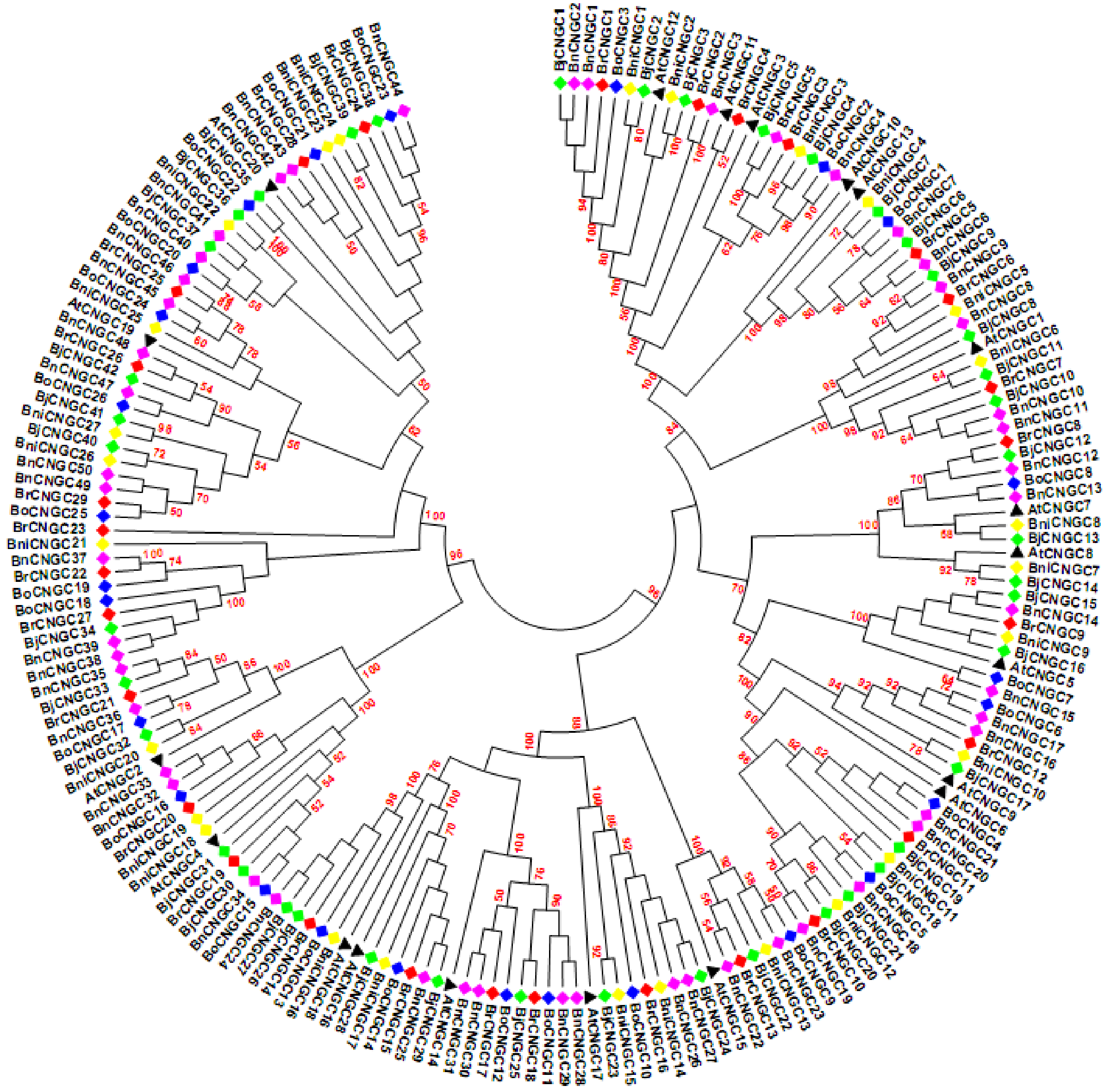

Figure S6. Gene structure showing exon-intron organization of *A. thaliana CNGC* genes

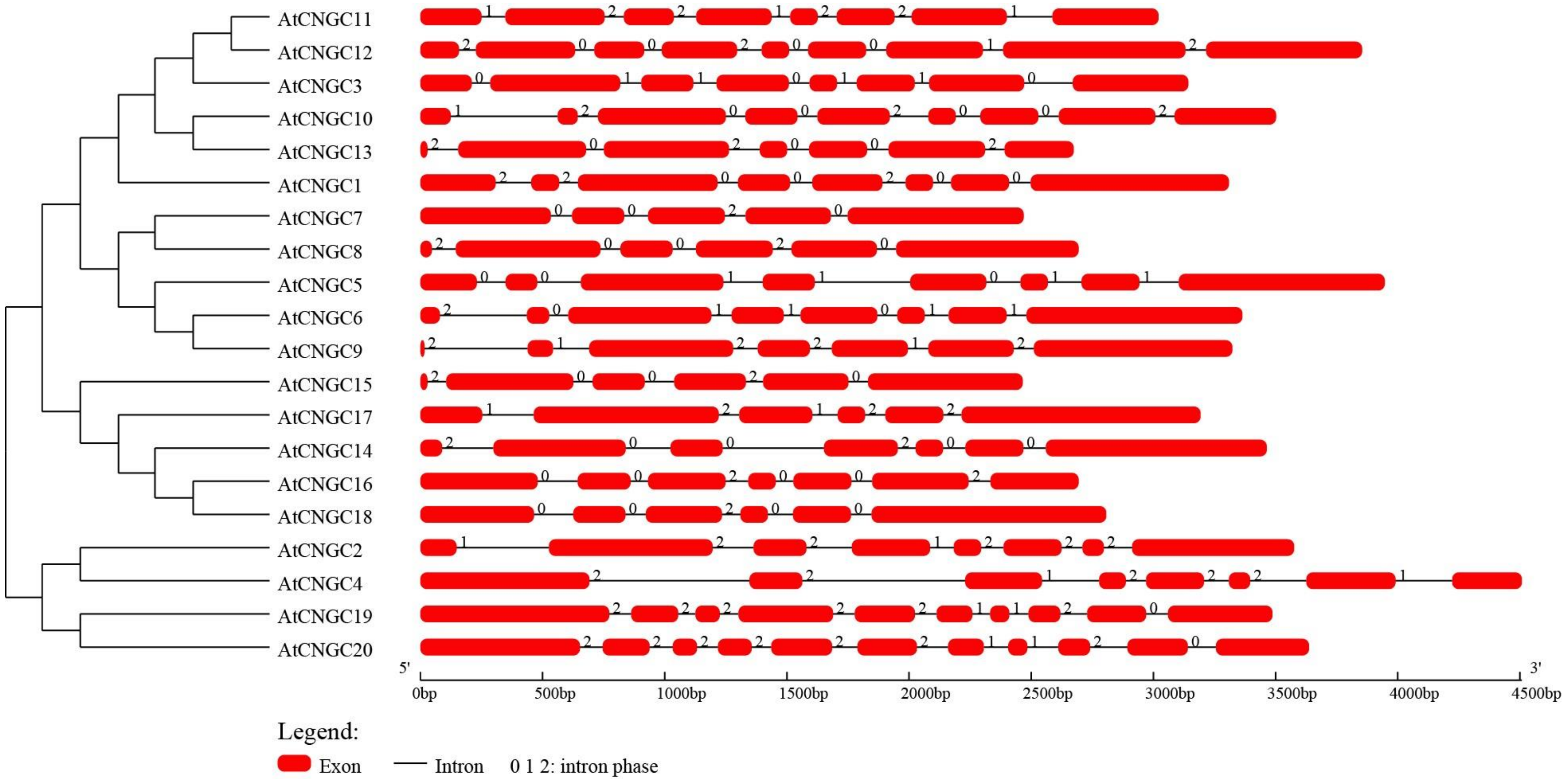

Figure S7. Gene structure showing exon-intron organization of *B. oleracea CNGC* genes

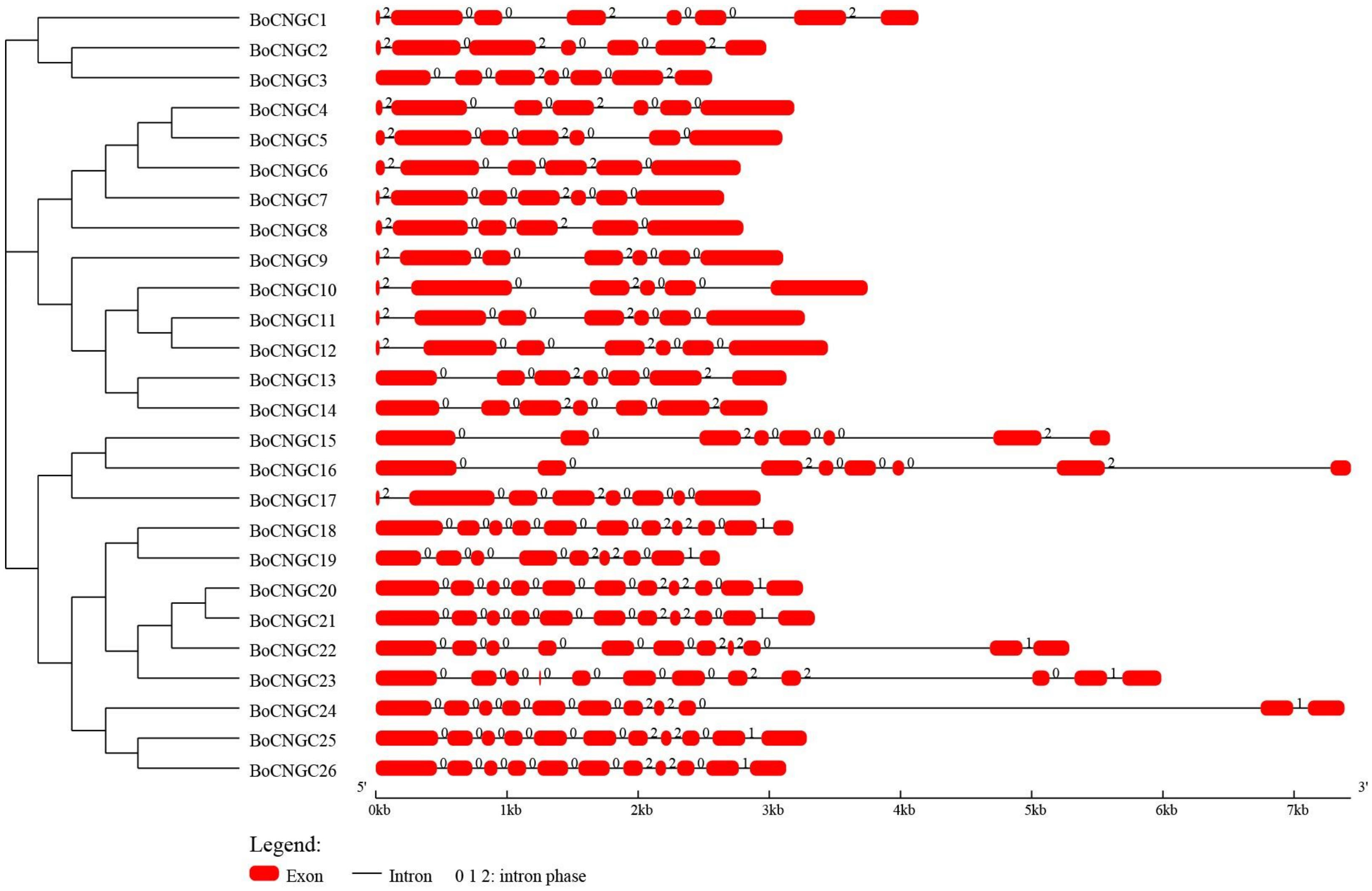

Figure S8. The logos of functionally annotated conserved motifs. The details of these motifs can be found in S7 Table

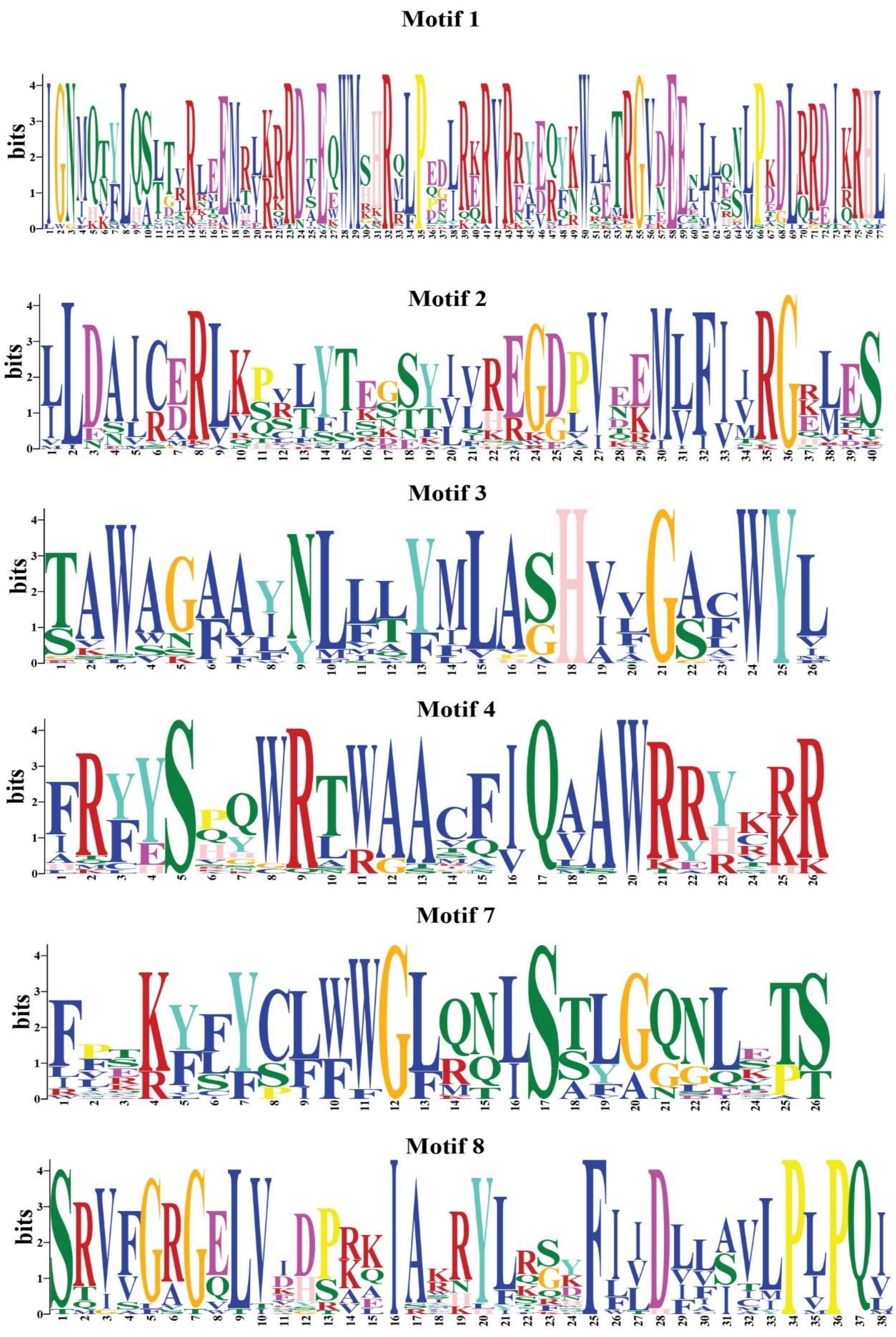

Figure S9. Distribution of positively charged and negatively charged amino acid residues in *BrCNGCs*

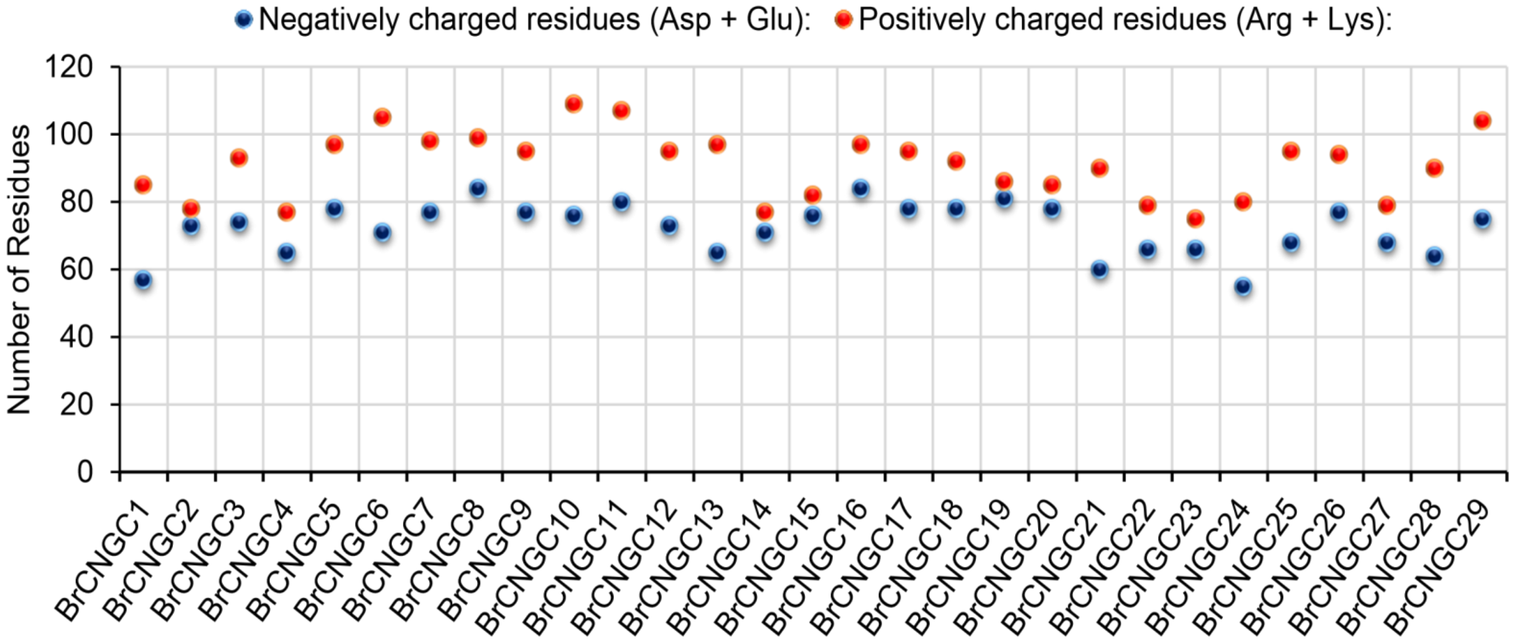

Figure S10. Atomic composition of BrCNGCs

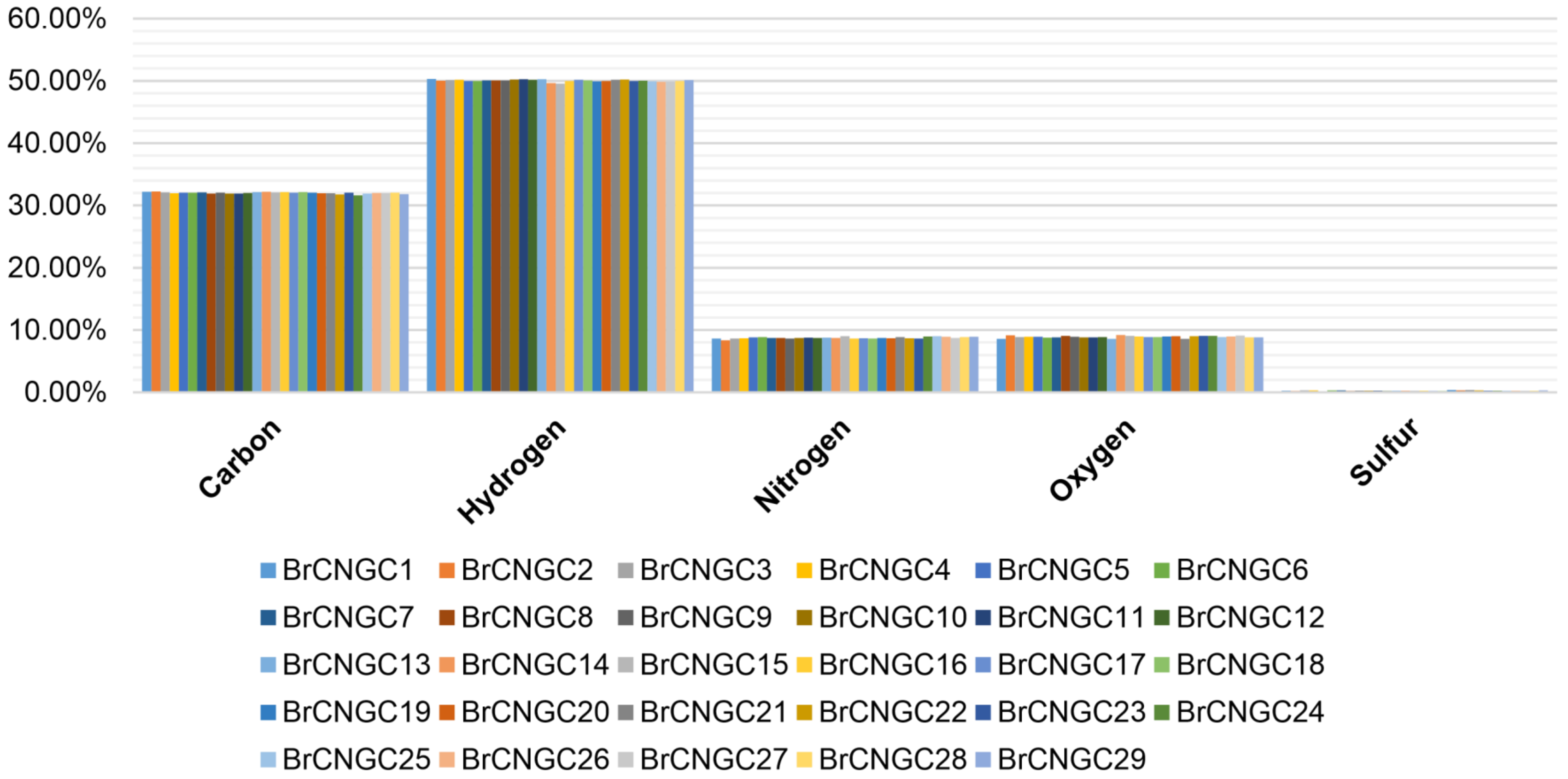

Figure S11. Composition of BrCNGC proteins showing the distribution of 20 amino acids in their protein sequences

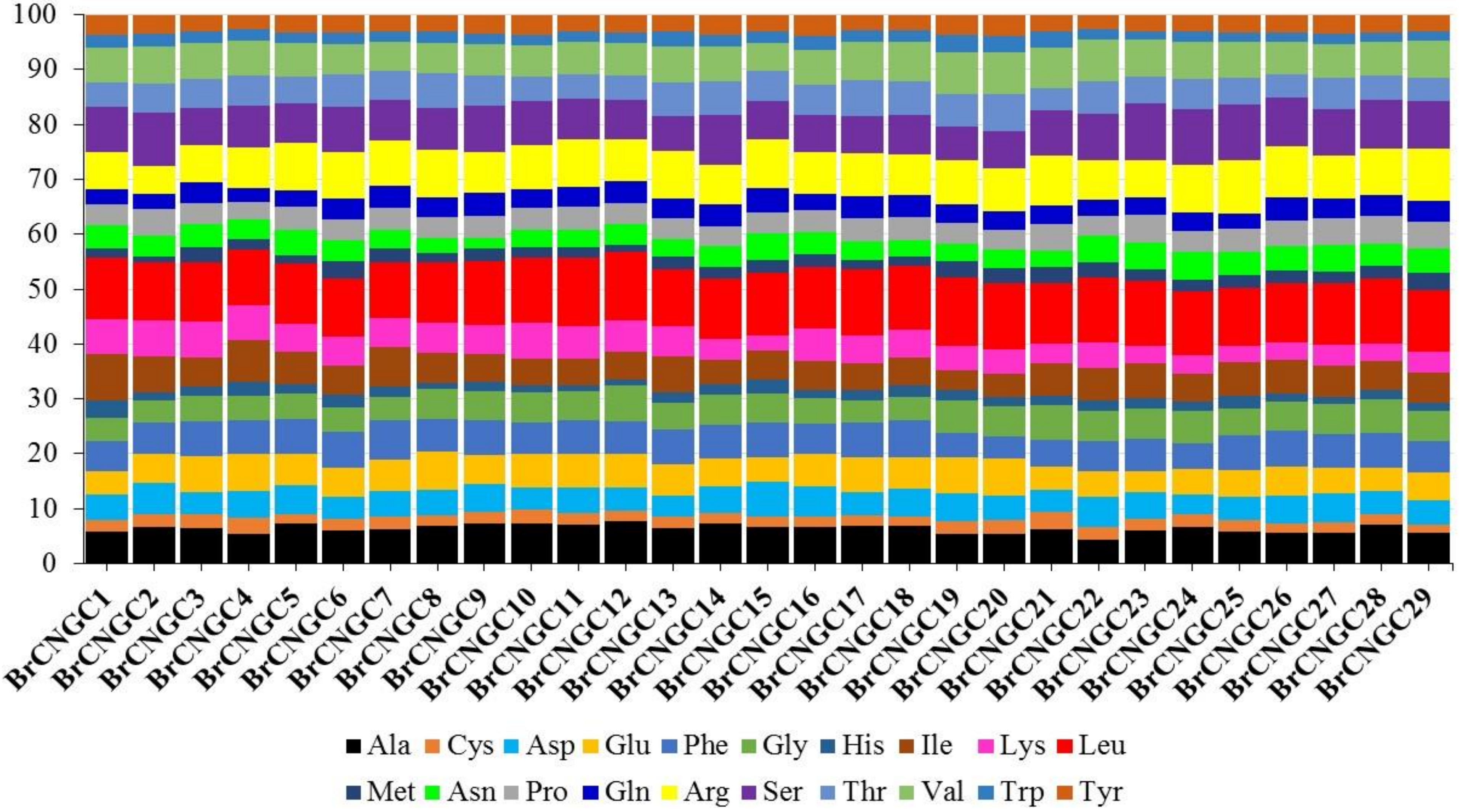

Figure S12. Functional association network of BrCNGC proteins. Each association is represented by different colors, where proteins jointly contribute to a shared function.

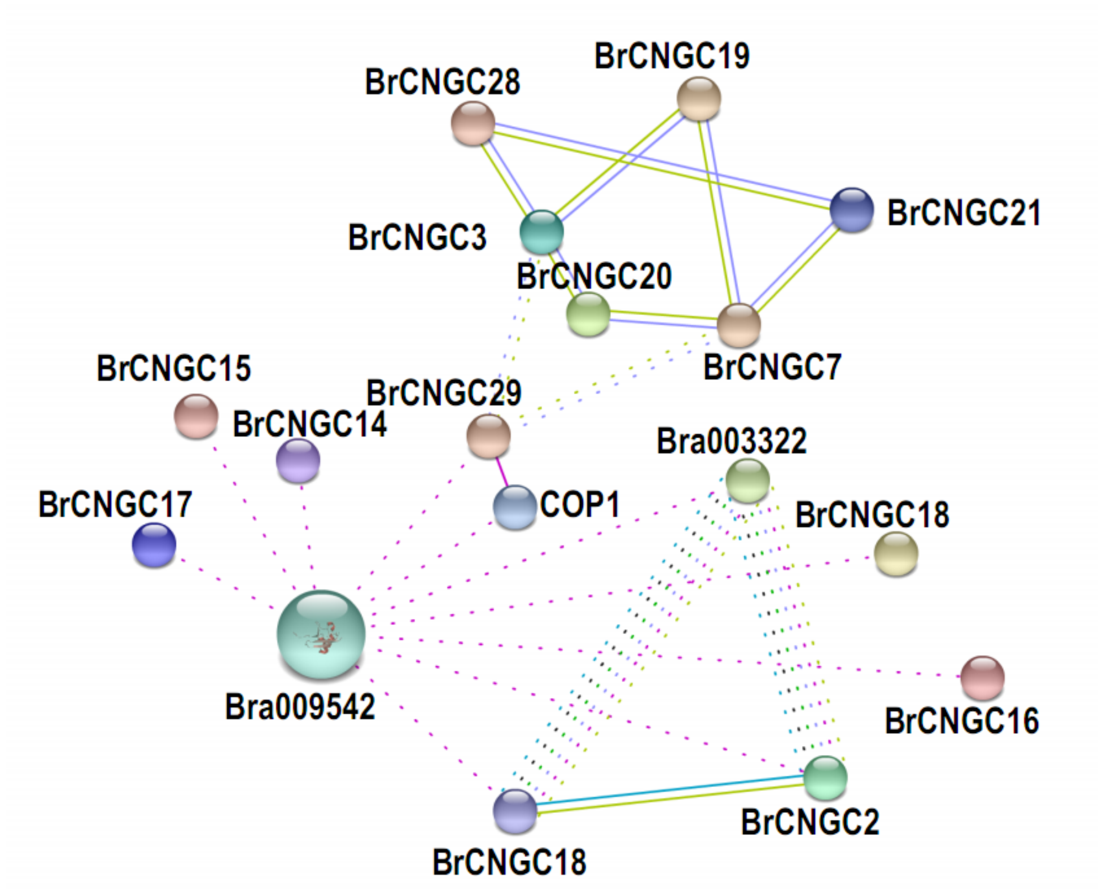

Supplement: Supplementary file 1 — Supplementary Information 1. [file 41598_2021_3712_MOESM1_ESM.pdf]
